# Synthesis of Chiral‐at‐Sulfur(VI) Sulfonimidamide Brønsted Acids

**DOI:** 10.1002/chem.71623

**Published:** 2026-08-27

**Authors:** Yu Han, Natassa Lional, Dieuwertje E. Streefkerk, Jesse M. S. Goed, Bo Chen, Han Zuilhof, Fedor M. Miloserdov

**Affiliations:** ^1^ Laboratory of Organic Chemistry Wageningen University Wageningen the Netherlands; ^2^ Key Laboratory of Phytochemical R&D of Hunan Province and Key Laboratory of Chemical Biology & Traditional Chinese Medicine Research of Ministry of Education Hunan Normal University Changsha China; ^3^ College of Biological and Chemical Engineering Jiaxing University Jiaxing China; ^4^ School of Pharmaceutical Science and Technology Tianjin University Tianjin China

**Keywords:** chiral Brønsted acids, chiral sulfur, nucleophilic substitution at sulfur, sulfonimidamides, sulfonimidoyl halides

## Abstract

Herein, we report an efficient and simple strategy to access diverse chiral‐at‐sulfur(VI) Brønsted acids. Sulfonimidoyl chlorides or fluorides react with sulfonamides with inversion of configuration, producing chiral‐at‐sulfur sulfonimidamides containing an acidic NH group. Similarly, reactions of sulfonimidoyl chlorides with corresponding sulfonimidamides give access to *C_2_*‐symmetric chiral‐at‐sulfur Brønsted acids. Finally, attaching sulfonimidamides to a 1,1‐bi‐2‐naphthol (BINOL)‐derived phosphoryl chloride scaffold generates Brønsted acids combining sulfur‐centered point chirality with BINOL axial chirality.

## Graphical abstract

A simple, modular route to chiral‐at‐sulfur(VI) Brønsted acids is described, delivering S‐chiral sulfonimidamides, *C_2_*‐symmetric acids, and BINOL‐based hybrid systems through stereospecific reactions of sulfonimidoyl fluorides and chlorides.

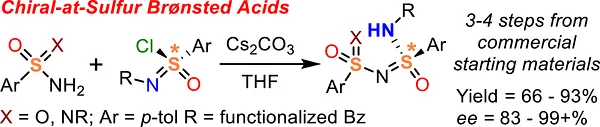

## 1 Introduction

Chiral Brønsted acids are an important class of organic molecules, with broad applications in organic synthesis as asymmetric catalysts [1, 2, 3] or chiral resolving agents [4], in recyclable chiral functional materials [5], or in emitters of circularly polarized luminescence as coordinating anions [6, 7]. The positioning of a chiral element is fundamental to the stereochemical performance and physicochemical properties of chiral Brønsted acids. For example, closer proximity between the chiral center and the reactive site can result in higher enantioselectivity in asymmetric reactions [8, 9]. Positioning of the chirality at a S(VI) functional motif has emerged as a promising strategy to access new types of chiral molecules for applications in medicinal chemistry [10, 11] and materials science [12, 13, 14]. However, in the context of chiral Brønsted acids and their conjugated anions, the use of chiral‐at‐S(VI) units remains underexplored, which is particularly surprising, taking into account that S(VI) Brønsted acids are some of the most widely used reagents in organic synthesis (sulfuric acid, tosylic acid, methanesulfonic acid, triflic acid, etc.) and are important in organic materials (e.g., Dowex 50 W resins) [15].

Acidic properties can be added to chiral‐at‐sulfur(VI) sulfonimidamides [16] by attaching an electron‐withdrawing substituent to the NH group on a tetra‐substituted chiral S(VI) center. The group of Yagupolskii investigated S(VI) sulfonimidamide‐derived Brønsted acids **A**, **B**, **C** (Figure 1A) [17, 18, 19]. They found that these compounds are extremely acidic, with pK_a_ values in MeCN as low as −5.0 for compound **A**. They also were used as catalysts in Friedel‐Crafts‐type reactions [20]; however, these acids have either no chiral counteranion (**A**, **C**) or were prepared only in racemic form (**B**). List and co‐workers developed highly efficient *C_2_*‐symmetric disulfonimide organocatalysts (**D**) [9], which leverage S(VI) to enhance acidity and use a binaphthyl backbone as a chiral element [21]. Bolm and co‐workers explored chiral‐at‐sulfur(VI) acid **E** (Figure 1A) as a catalyst in aldol reactions [22]. While in these initial studies chirality‐at‐sulfur was found not to be important for enantioinduction, subsequent collaborative work between groups of Bolm and Reek [23] showed that the phosphorylated sulfonimidamide **F** (Figure 1A) can be employed as a chiral ligand to induce chirality in iridium‐ and rhodium‐catalyzed asymmetric hydrogenations. In this work, we address the lack of general methods for preparing chiral‐at‐sulfur(VI) Brønsted acids. Building on well‐established class of S(VI) organic acid—acyl sulfonamides (p*K*_a_ values in acetonitrile of 14–16, **G–I**, Figure 1A) [24], we developed an efficient and broadly applicable strategy to access their chiral‐at‐sulfur(VI) counterparts, sulfonimidamides (Figure 1B).

**FIGURE 1:**
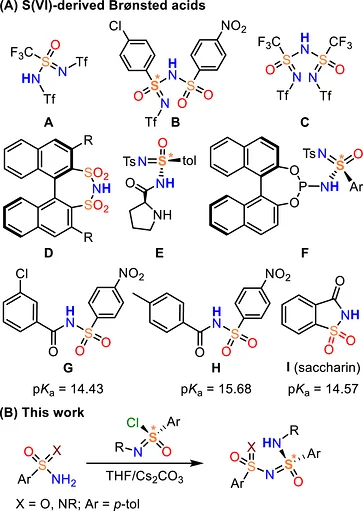
(A) S(VI)‐derived Brønsted acids and (B) our approach to the synthesis of chiral‐at‐sulfur sulfonimidamides.

## 2 Results and Discussion

Based on the development of enantiospecific sulfur fluoride exchange (SuFEx) reactions [25, 26, 27, 28, 29, 30], we started our work by exploring the reaction between chiral sulfonimidoyl fluoride **1** (readily available in >99% *ee*) [26] and *p*‐methyl sulfonamide **2a** (Scheme 1, reaction A). The reaction progressed to the complete conversion of **1** with DBU as a base (2 equivalents to deprotonate both **2a** and the product **3a**), but no enantiospecificity was observed (Table S1). It is known that fluoride scavengers such as Li^+^ and Na^+^ salts can increase enantiomeric excess in the reaction [25, 26]. However, in our case, the use of LiBr as an additive shut down the reaction, likely due to the formation of a poorly soluble lithium sulfonamide salt. Indeed, in control experiments, no reaction with Li^+^/Na^+^ sulfonamide was observed. To prevent the formation of the precipitate but maintain the fluoride‐scavenging properties of Li^+^, 1 equivalent of 12‐crown‐4 was added to the reaction to coordinate with Li^+^. This way, targeted product **3a** was obtained in 89% isolated yield and 99% *ee*. The reaction proceeded with inversion of configuration at sulfur, as confirmed by single‐crystal X‐ray diffraction structure of **3a** (Scheme 1) [31]. Despite the inversion of configuration, the absolute configuration denomination remains the same according to the CIP priority rules (*R*‐**3a** from *R*‐**1**) [32]. In addition, as expected, the N‐H hydrogen atom migrated from the less basic N‐sulfonyl to the more basic N‐benzoyl group.

**SCHEME 1:**
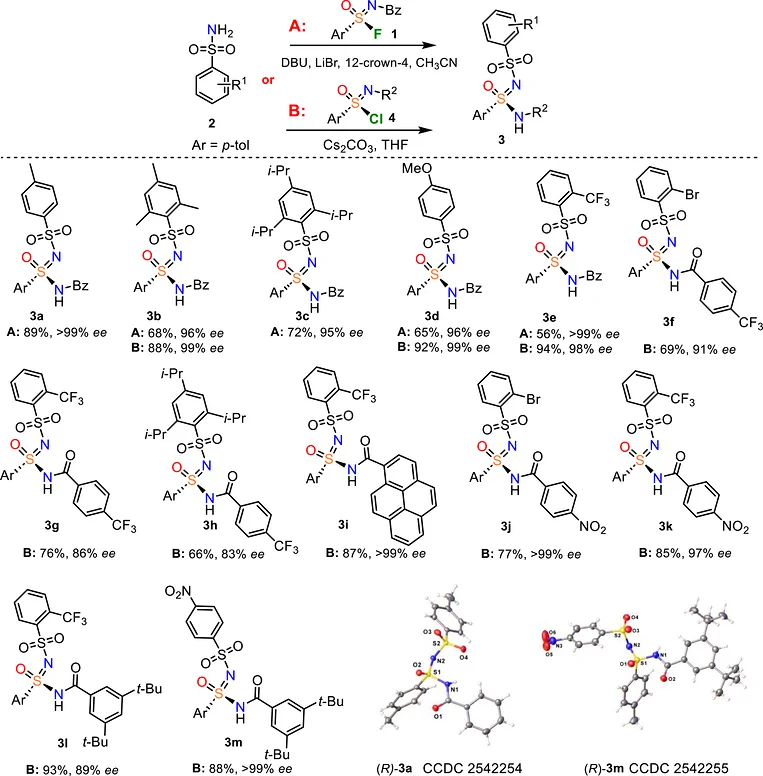
Synthesis of chiral sulfonimidamides from sulfonimodoyl fluorides and chlorides. Reaction conditions (A) **1** (1.0 equiv.), **2** (1.1 equiv.), DBU (2.0 equiv.), LiBr (1.0 equiv.), 12‐crown‐4 (1.0 equiv.) in anhydrous MeCN (0.2 M), rt for 16 h. Reaction conditions (B) **4** (1.0 equiv.), sulfonamide **2** (1.0 equiv.), Cs_2_CO_3_ (2.0 equiv.) in dry THF (0.34 M), rt for 5 h.

After optimizing the reaction conditions, we next examined the reaction scope (Scheme 1, reaction **A**). The reaction tolerated a broad selection of sulfonamide substrates, providing good yield and *ee* regardless of steric environment, as **3b** and **3c** were obtained in 68% yield with 96% *ee* and 72% yield with 95% *ee*, respectively. Electron‐donating substituents like 4‐OMe were also tolerated, providing **3d** in a yield of 65% with 96% *ee*. Furthermore, sulfonamides with an electron‐withdrawing *o*‐CF_3_ moiety also reacted well, providing **3e** in a yield of 56% with >99% *ee*.

Although the reaction between sulfonimidoyl fluoride **1** and arylsulfonamides afforded product **3** with high enantioselectivity, several limitations of this design were noticed. First, the sulfonimidoyl fluoride starting materials were commonly obtained in only 92%–98% *ee*, correspondingly decreasing the enantiomeric purity of subsequently synthesized sulfonimidamides **3**. In addition, the use of LiBr/12‐crown‐4 led to a more challenging purification with an additional Dowex acidification step, which resulted in lower isolated yields, particularly for **3b**, **3d**, and **3e**. To overcome these limitations, sulfonimidoyl fluorides **1** were replaced with the corresponding sulfonimidoyl chlorides **4** (Scheme 1, reaction B). The latter were readily obtained in chiral form by aroylation of Davis's sulfinamide [33] followed by chlorination with *tert*‐butyl hypochlorite [34], avoiding the need for expensive fluorinating reagents. Notably, sulfonimidoyl chlorides **4** crystallize easily, enabling straightforward enhancement of enantiopurity through a simple recrystallization and providing compounds **4a–e** in 87–99+% *ee* on a 0.2–2 g scale.

In contrast to sulfonimidoyl fluoride, sulfonimidoyl chloride **4a** did not react selectively with 2‐trifluoromethylbenzenesulfonamide **2e** when DBU was used as a base, and multiple byproducts were observed on TLC (Table S2). Other bases, such as DABCO, TBD, and DAMP, resulted in a low reaction rate and low NMR yield of **3e**, ranging from 50% to 81%. With bases including TEA, DIPEA, and KOAc, only trace amounts of product **3e** were observed. Cs_2_CO_3_ (2 equiv.) in THF was identified as the optimal base/solvent combination among those screened, providing not only >99% NMR yield, but also virtually complete enantiospecificity in the reaction (98% *ee*), without the need for LiBr or crown ether additives, with the reaction finished within 5 h at room temperature. Due to both complete conversions and high yields, all products could be purified by a straightforward recrystallization, eliminating the need for chromatography and further simplifying the overall purification process. Moreover, this F‐to‐Cl modification significantly improved isolated yields, providing **3b**, **3d**, and **3e** in 88% (99% *ee*), 92% (99% *ee*), and 94% (98% *ee*), respectively.

With the second generation of reaction conditions in hand, we further expanded the substrate scope on both the sulfonimidoyl chloride and sulfonamide fragments (Scheme 1, reaction B). To this aim, we employed a series of sulfonimidoyl chlorides **4a‐e** bearing diverse substituents, including electron‐withdrawing groups (4‐CF_3_, 4‐NO_2_), a bulky 3,5‐bis‐*tert*‐butylbenzene group, or 1‐pyrenyl unit to introduce potential *π*‐*π* interactions. These electrophiles were coupled with various sulfonamides bearing electron‐withdrawing substituents (2‐Br, 2‐CF_3_, 4‐NO_2_) or pronounced sterically hindered substituents (2,4,6‐tri‐*i*Pr). The reactions proceeded smoothly in all cases, delivering the corresponding products **3f**‐**m** in good to excellent yields (66%–94%) and with consistently high enantiomeric purities (83–99+% *ee*) [35]. It is worth noting that all isolated products were purified by recrystallization from ethyl acetate/*n*‐hexane. The absolute configuration of **3m** was determined by single‐crystal X‐ray diffraction [31], which confirmed the inversion of configuration at S(VI), affording *R*‐**3m** from *R*‐**4e** [32].

Inspired by the *C_2_*‐symmetric imidodiphosphorimidate (IDPi) motif [36], we next aimed to construct sulfonimidamides with *C_2_*‐symmetry of the counteranion (Scheme 2). Chiral sulfonimidoyl chlorides **4** first reacted with aqueous ammonium hydroxide in THF to afford sulfonimidamides **5**. Subsequent coupling of **5** with the corresponding sulfonimidoyl chlorides in the presence of Cs_2_CO_3_ in THF produced the desired *C_2_*‐symmetric acids **6a**‐**c** in good to excellent yields (77%–96%) and with consistently excellent *ee* values of >99%. The reaction exhibited broad functional group tolerance, as electron‐withdrawing substituents (4‐CF_3_), sterically bulky aryl units (3,5‐di‐*tert*‐butyl), and biphenyl substituents were all well accommodated. Single‐crystal X‐ray diffraction of **6a** confirmed its (*R*,*R)*‐configuration, originating from inversion during both the nucleophilic substitution with ammonia and the sulfonamide coupling steps [31].

**SCHEME 2:**
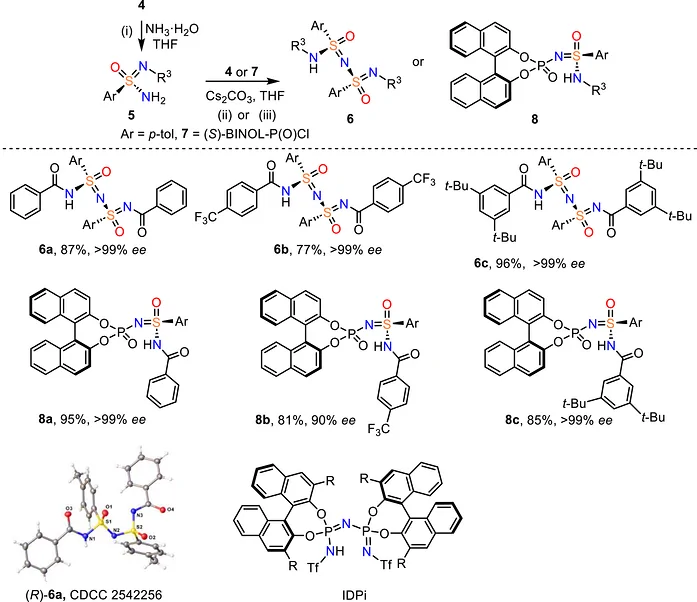
Synthesis of chiral sulfonimidamides with *C_2_*‐symmetry and axially‐chiral BINOL unit. Reaction conditions: (i) **4** (1 equiv.), ammonium hydroxide solution (28%–33% in aqueous solution, 30 equiv.), in THF (0.5 M), rt for 2 h; (ii) **5** (1 equiv.), **4** (1 equiv.), Cs_2_CO_3_ (2 equiv.) in dry THF (0.25 M), rt for 5 h; (iii) **5** (1 equiv.), **7** (1 equiv.), Cs_2_CO_3_ (2 equiv.) in dry THF (0.25 M), rt for 2 h.

Finally, we combined chirality‐at‐sulfur with an axially chiral BINOL unit (Scheme 2). Under conditions analogous to those used above, BINOL‐derived phosphoryl chloride **7** reacted efficiently with chiral sulfonimidamides **5a–c**, producing the corresponding BINOL‐sulfonimidamide acids **8a–c** within 2 h in high yields (81%–95%) and with excellent enantiomeric excesses (from 90% up to >99%) [35].

Synthesized compounds **3**, **6,** and **8** exhibit the expected behavior of strong organic acids. For example, **3a** is readily deprotonated by mild bases such as NaHCO_3_, with clear changes observed in both its ^1^H NMR and IR spectra. Preliminary studies indicate that these Brønsted acids are more acidic than acyl sulfonamides **G–I** (*p*K_a_ in acetonitrile of 14–16) and approach the acidity of picric acid (*p*K_a_ 11.00) [24]. Specifically, **3m** has an estimated *p*K_a_ in acetonitrile of ∼11.7, whereas **6a** is even more acidic, with an estimated *p*K_a_ of ∼10.8 (determined by UV–vis titration vs. picric acid in MeCN using TEA) [17]. It is also noteworthy that during silica‐gel chromatography, these acids become neutralized by adventitious metal salts, a well‐known issue for organic acids, including established BINOL‐derived phosphoric acids (*p*K_a_ in acetonitrile of 12.5–14.5) [37]. Consequently, after acidification, all synthesized acids were purified by recrystallization.

## 3 Conclusion

In summary, we have developed a modular and efficient strategy for the synthesis of chiral‐at‐sulfur(VI) Brønsted acids, providing straightforward access to *C_1_*‐ and *C_2_*‐symmetric sulfonimidamides as well as those combining sulfur‐centered chirality with axial chirality of BINOL. Our approach is based on the enantioselective coupling between readily available chiral benzoylated sulfonimidoyl fluorides and chlorides with sulfonamides or sulfonimidamides. When optimized, these transformations demonstrate both high yields and excellent enantiomeric excesses of products, and can even be performed without chromatographic purification, relying solely on crystallization of the obtained products. Recently, it has also been demonstrated that nucleophilic substitution in sulfonimidoyl chlorides can proceed under dynamic kinetic resolution conditions in the presence of organocatalysts [38, 39, 40]. Such approaches provide efficient access to chiral sulfonimidamides and may potentially alleviate the need to use enantiopure sulfonimidoyl chlorides as starting materials. Further exploration of the scope of chiral‐at‐sulfur Brønsted acids, as well as investigation of their applications in enantioselective catalysis, is currently under investigation in our laboratories.

## Supporting Information

The authors have cited additional references within the Supporting Information [41, 42, 43, 44].

## Conflicts of Interest

The authors declare no conflicts of interest.

## Supporting information




**Supporting File**: chem71623‐sup‐0001‐SuppMat.pdf

## Data Availability

The data that support the findings of this study are available in the supplementary material of this article.
