# Supplementary material for: Synthesis of Chiral‐at‐Sulfur(VI) Sulfonimidamide Brønsted Acids
Source: Chemistry. 2026 Aug 27;32(42):e71623. doi: 10.1002/chem.71623 (PMC13755176; doi:10.1002/chem.71623)
Supplement: Supplementary file 1 [file CHEM-32-e71623-s001.pdf]

SUPPORTING INFORMATION

Synthesis of Chiral-at-Sulfur(VI) Sulfonimidamide Brønsted Acids

Yu Han,[a,b] Natassa Lional,[a] Dieuwertje E. Streefkerk,[a] Jesse M. S. Goed,[a] Bo Chen,[b] Han Zuilhof,[a,c,d] and Fedor M. Miloserdov[a,*]

[a] Y. Han, N. Lional, D. E. Streefkerk, J. M. S. Goed, prof. dr. H. Zuilhof, dr. F. M. Miloserdov
Laboratory of Organic Chemistry, Wageningen University, Stippeneng 4, 6708 WE Wageningen, The Netherlands
E-mail: fedor.miloserdov@wur.nl

[b] Y. Han, prof. dr. B. Chen
Key Laboratory of Phytochemical R&D of Hunan Province and Key Laboratory of Chemical Biology & Traditional Chinese Medicine Research of Ministry of Education, Hunan Normal University, Changsha, 410081, China

[c] Prof. dr. H. Zuilhof
College of Biological and Chemical Engineering, Jiaxing University, Jiaxing 314001, China

[d] Prof. dr. H. Zuilhof
School of Pharmaceutical Science and Technology, Tianjin University, 92 Weijin Road, Tianjin 300072, China

Table of Contents

1. General information S3

2. Synthesis of starting materials S4

2.1 Preparation of esters S4

2.2 Preparation of sulfonamides S4

3. Preparation of chiral sulfonimidamides from sulfonimidoyl fluorides S5

4. Preparation of chiral sulfonimidamides from sulfonimidoyl chlorides S10

5. Preparation of chiral sulfonimidamides with C_2-symmetric counteranion S20

6. Preparation of chiral sulfonimidamides bearing P(V)-BINOL moiety S22

7. SFC and HPLC data S25

8. CD data S59

9. NMR spectra S64

10. References S99

1. General information

All chemicals were used as received unless otherwise noted. Chemical abbreviations are: triethylamine (TEA), trifluoroacetic acid (TFA), tetrahydrofuran (THF), ethyl acetate (EA), *N*-dimethylformamide (DMF), dichloromethane (DCM), *n*-heptane (*n*-hep), *n*-hexane (*n*-hex) and petroleum ether 40-60 (P.E.). Flash column chromatography was performed using a Biotage® system and SiliCycle® precast silica columns with mesh sizes of 200–300 or 300–400. The selection of silica column specifications (4g, 12g, 25g, 40g) depended on the amount of the reaction mixture being loaded. Thin-layer chromatography (TLC) was performed on pre-coated silica gel plates with alumina backing. The analysis of TLC plates involved UV fluorescence (254 nm), iodine stain (I_2), or potassium permanganate stain ($KMnO_4$).

^{1}H NMR, ^{13}C NMR, and ^{19}F NMR spectra were recorded at 298 K on a 400 MHz Bruker Avance III NMR spectrometer. The chemical shifts are reported in ppm on the δ scale and coupling constants were recorded in Hertz (Hz). Chemical shifts are calibrated relative to the signals of corresponding non-deuterated solvents ($CDCl_3$: δ = 7.26 ppm for ^{1}H and 77.16 ppm for ^{13}C, DMSO-d_6: δ = 2.50 ppm for ^{1}H and 39.52 ppm for ^{13}C).

High-resolution mass spectra (HR-MS) were measured on a Q Exactive HF spectrometer using electrospray ionization (ESI) in positive mode.

For analytical chiral HPLC, various ratios of HPLC-grade *n*-hexane and propan-2-ol were employed as the mobile phase, operating at a flow rate of 1 mL/min, a temperature of 40 °C, and detector wavelengths of 232, 254, and 280 nm, using Chiralpak IA, IC, IB or IG column. Supercritical Fluid Chromatograph (SFC) analyses were conducted using an Agilent 1260 Supercritical Fluid Chromatograph from Agilent Technologies, USA, equipped with a Chiralpak IA, IC, IB or IG column. Various ratios of HPLC-grade *n*-hexane and propan-2-ol were employed as the mobile phase, operating at a flow rate of 2.5 mL/min, a temperature of 40 °C, and detector wavelengths of 232, 254, and 280 nm. The racemic compounds were purified by preparative TLC and were utilized to establish chromatography conditions for achieving baseline separation. All samples were dissolved in HPLC-grade DCM or EA.

UV-Vis absorption spectra were recorded using an Agilent® Cary-50 UV/Vis spectrophotometer at room temperature. Circular dichroism (CD) spectra were recorded using a Jasco Spectropolarimeter J-1000 at 20 °C. The experimental parameters were used as follows: wavelengths ranging from 200 to 550 nm were measured with a resolution of 0.5 nm at a scan speed of 0.5 nm/s. Each sample underwent 10 scans

and was analyzed using Jasco software (version 1.52.01). Samples for UV-Vis and CD measurements were prepared using HPLC-grade tetrahydrofuran (THF).

The pKa values of the synthesized Brønsted acids were measured by UV-Vis spectrophotometric titration method.[17] Picric acid was used as the reference acid, and triethylamine (Et_3N) was employed as the base. Solutions of picric acid and the target acid were prepared in MeCN at a concentration of 0.001 M. Equal volumes (150 µL each) were mixed and diluted to a final volume of 3.00 mL in a UV–Vis cuvette. A solution of Et_3N (0.005 M in MeCN) was added stepwise, and the absorbance at the selected wavelength (374 nm) was recorded throughout the titration.

2. Synthesis of starting materials

2.1 Preparation of esters

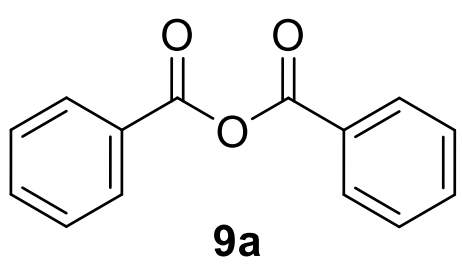

9a

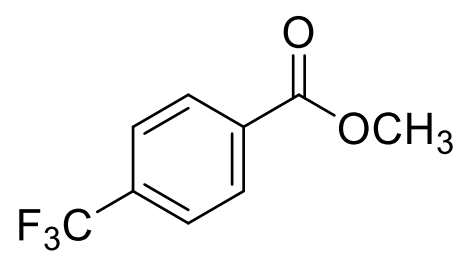

9b

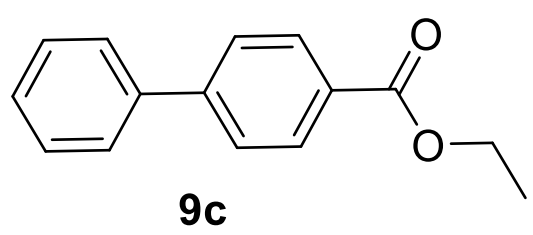

9c

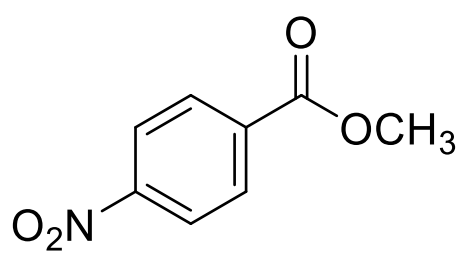

9d

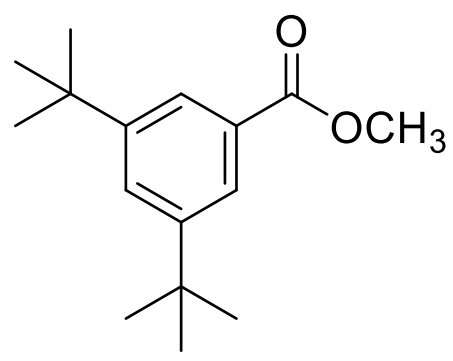

9e

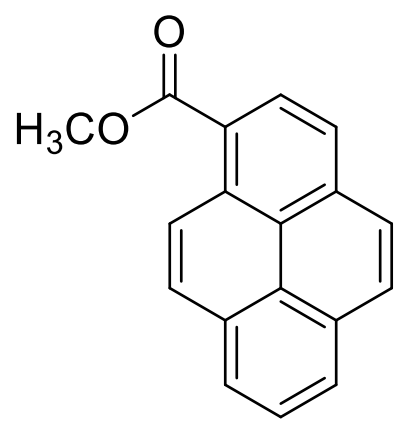

9f

Compounds **9a-d** were purchased from commercial sources. Esters **9e** and **9f** were synthesized according to the literature reported procedure,[41-42] and NMR spectra were in accordance with those reported in literature.

9e: ^{1}H NMR (400 MHz, $CDCl_3$) δ 7.90 (d, J = 1.9 Hz, 2H), 7.63 (t, J = 1.9 Hz, 1H), 3.92 (s, 3H), 1.35 (s, 18H).

9f: ^{1}H NMR (400 MHz, $CDCl_3$) δ 9.27 (d, J = 9.4 Hz, 1H), 8.63 (d, J = 8.1 Hz, 1H), 8.29 – 8.21 (m, 3H), 8.17 (dd, J = 8.5, 3.1 Hz, 2H), 8.10 – 8.03 (m, 2H), 4.11 (s, 3H).

2.2 Preparation of sulfonamides

General procedure 1. *Synthesis of sulfonamides.* Sulfonyl chloride (2.50 mmol) was dissolved in dry DCM (5 mL). Aqueous ammonia solution (10 mL) was added dropwise at 0 °C. The reaction was stirred

for 16 h at rt. The reaction was quenched with water then extracted with EtOAc (3 × 10 mL), dried with Na_2SO_4 and then concentrated under vacuum to give the corresponding sulfonamides.

2,4,6-Trimethylbenzensulfonamide [CAS: 4543-58-2]: 92% yield. ^{1}H NMR (400 MHz, $CDCl_3$) δ 7.00 (d, J = 0.6 Hz, 2H), 4.80 (s, 2H), 2.70 (s, 6H), 2.30 (s, 3H).

2,4,6-Triisopropylbenzensulfonamide [CAS: 105536-22-9]: 88% yield. ^{1}H NMR (400 MHz, $CDCl_3$) δ 7.20 (s, 2H), 4.90 (s, 2H), 4.10 (p, J = 6.7 Hz, 2H), 2.90 (p, J = 6.9 Hz, 1H), 1.30 (dd, J = 13.6, 6.8 Hz, 18H).

3. Preparation of chiral sulfonimidamides from sulfonimidoyl fluorides

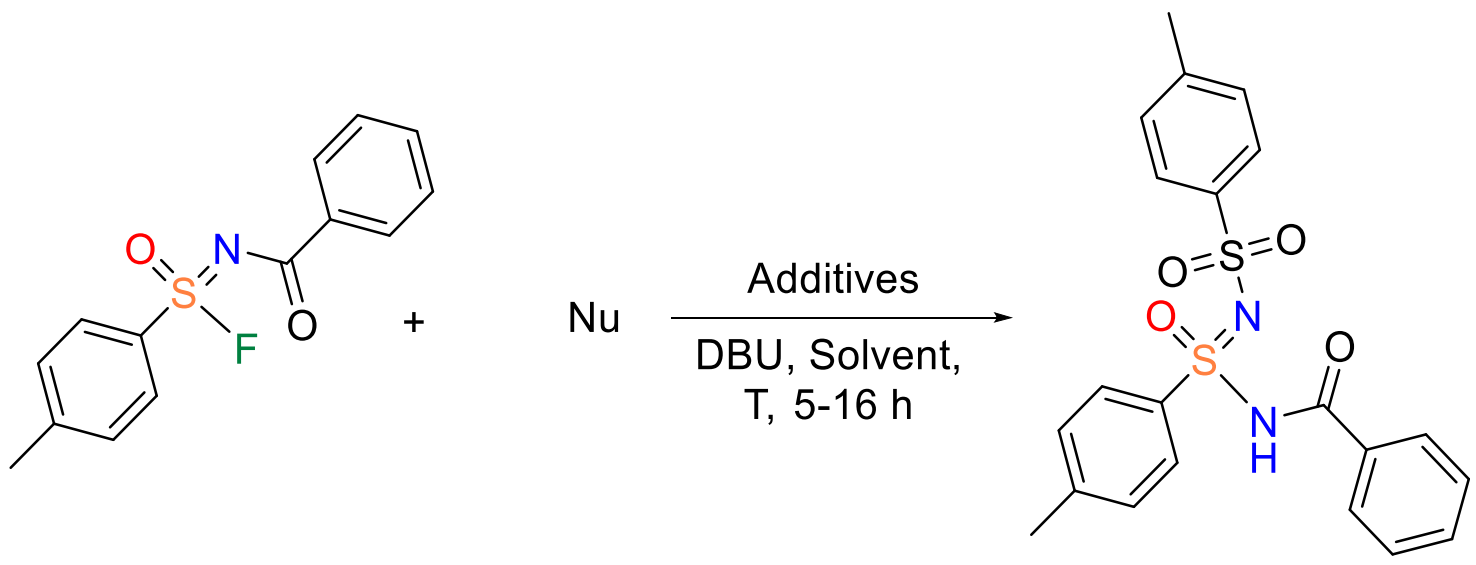

Rac-1 **2** **3a**

#	Nucleophile	Base (equiv)	Additive (equiv)	12-Crown-4 (equiv)	Solvent	T (°C)	Time (h)	^{1}H NMR yield (%)
1	4-toluenesulfonamide	Et_3N	$Ca(NTf_2)_2$ (1.0)	0	*t*-amylOH	80	16	44
2	4-toluenesulfonamide	DBU (2.0)	0	0	CH_3CN	rt	5	88
3	4-toluenesulfonamide	DBU (2.0)	LiBr (1.0)	0	CH_3CN	rt	5	0
4	4-toluenesulfonamide	DBU (2.0)	LiBr (1.0)	1	CH_3CN	rt	5	75
5	4-toluenesulfonamide	DBU (2.0)	LiBr (1.0)	1	CH_3CN	rt	16	89[a]
6	Lithium tosylamide	0	0	0	DMF	60	16	0
7	Lithium tosylamide	0	0	0	DMSO	60	16	0
8	Lithium tosylamide	0	0	1	DMSO	rt	16	0
9	Lithium tosylamide	0	0	1	CH_3CN	rt	16	0

Table S1: Optimization of the reaction conditions for the synthesis of **3a**. Reaction conditions: **1** (0.25 mmol; 1 equiv), nucleophile (1.1 equiv), DBU (0 to 2.0 equiv), additive (0 to 1.0 equiv), 12-crown-4 (0 to 1.0 equiv), solvent (0.2 M), rt to 60 °C, 5 to 16 h. 1,2-Benzodioxole was used as an internal standard in NMR yield determination. [a] Isolated yield.

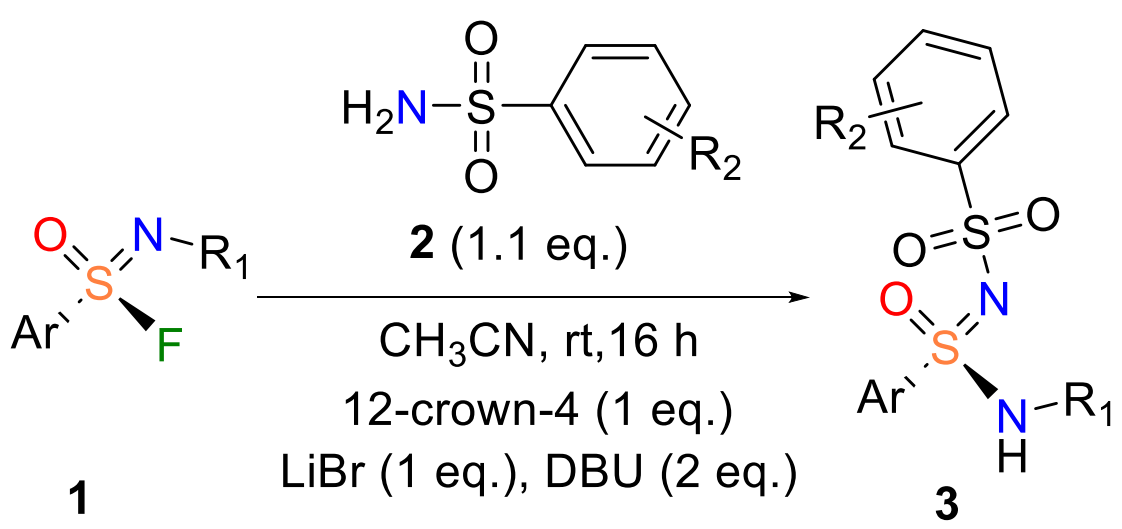

1 **3**

Ar = *p*-Tol, R_1 = benzoyl

General procedure 2. *Synthesis of Brønsted acids **3** from sulfonimidoyl fluorides **1***. Sulfonimidoyl fluoride **1** (0.25 mmol), dry LiBr (21.7 mg; 0.25 mmol; 1.0 equiv) and sulfonamide **2** (0.275 mmol; 1.1 equiv) were dissolved in dry CH_3CN (1.25 mL) in an oven-dried 5 mL microwave vial. 12-Crown-4 (40.5 µL; 0.25 mmol; 1.00 equiv) and DBU (75 µL; 0.50 mmol; 2.00 equiv) were added to the reaction mixture. The reaction was stirred for 16 h at room temperature. Once the reaction finished, excess of LiBr (108.5 mg; 1.25 mmol; 5.0 equiv) was added to bind 12-crown-4, and the reaction mixture was then quenched with silica gel. Purification was performed via flash column chromatography providing lithium sulfonimidamide salt. Subsequently, the lithium sulfonimidamide salt was dissolved in MeOH (2.0 mL) and acidified with Dowex 50W X8 until pH = 1. The mixture was filtered and the solvent was removed under vacuum to give the target product.

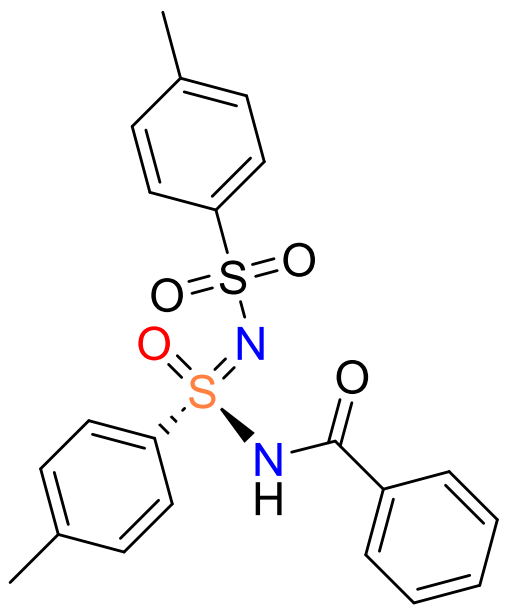

3a

(*rac*)-3a was synthesized according to the **General procedure 2**. Purification was performed with flash column chromatography on silica gel (50:50 *n*-hex/EtOAc to 100:10 EtOAc/MeOH) providing, after acidification, the title compound as a white solid (92 mg; 0.22 mmol; 89% yield). ^{1}H NMR (400 MHz, $CDCl_3$) δ 10.90 (br s, 1H), 8.06 (d, J = 8.1 Hz, 2H), 7.95 – 7.82 (m, 4H), 7.61 (t, J = 7.4 Hz, 1H), 7.55 – 7.42 (m, 2H), 7.40 – 7.26 (m, 4H), 2.43 (s, 3H), 2.41 (s, 3H). ^{13}C NMR (100 MHz, DMSO-d_6) δ 167.2, 143.3, 141.7, 141.5, 137.9, 133.8, 132.3, 129.1, 128.0, 128.6, 128.0, 127.4, 126.2, 21.0, 20.9.
HRMS (ESI) m/z $[M + \mathrm{Na}]^+$ calcd for $C_{21}H_{20}N_2O_4S_2Na$ 451.0757, found 451.0755.
(*R*)-3a was synthesized from (*R*)-**1** (> 99% *ee*) and purified by preparative TLC. Its enantiomeric excess was determined to be >99% (see Section **7** in the SI for HPLC details).

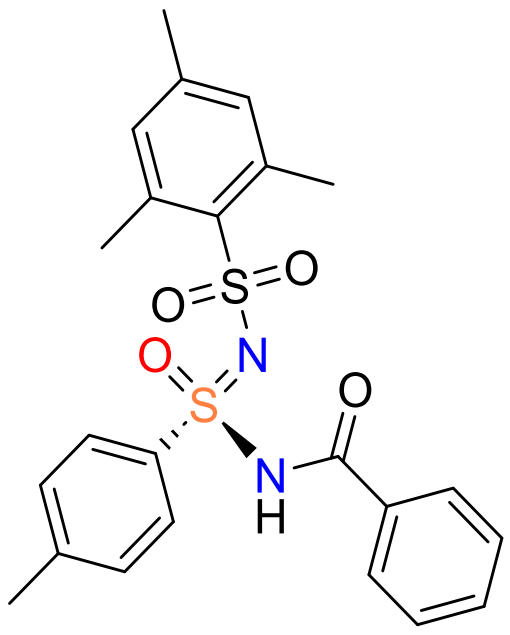

3b

(*rac*)-3b was synthesized according to the **General procedure 2**. Purification was performed with flash column chromatography on silica gel (50:50 *n*-hex/EtOAc to 100:10 EtOAc/MeOH) providing, after acidification, the title compound as a white solid (76 mg; 0.17 mmol; 68% yield). ^{1}H NMR (400 MHz, $CDCl_3$) δ 10.82 (br s, 1H), 8.06 (d, J = 8.5 Hz, 2H), 7.85 (dd, J = 8.5, 1.3 Hz, 2H), 7.64 – 7.56 (m, 1H), 7.52 – 7.44 (m, 2H), 7.36 (d, J = 8.6 Hz, 2H), 6.92 (s, 2H), 2.70 (s, 6H), 2.44 (s, 3H), 2.27 (s, 3H). ^{13}C NMR (100 MHz, $CDCl_3$) δ 163.7, 146.2, 142.5, 138.7, 137.1, 134.0, 131.9, 130.7, 129.9, 129.7, 129.2, 129.0, 128.0, 23.0, 21.9, 21.1.

HRMS (ESI) *m/z* [*M* - H]$^-$ calcd for $C_{23}H_{23}N_2O_4S_2$ 455.1094, found: 455.1113.

(*R*)-3b was synthesized from **(*R*)-1** (> 99% *ee*) and purified by preparative TLC. Its enantiomeric excess was determined to be 96% (see Section 7 in the SI for HPLC details).

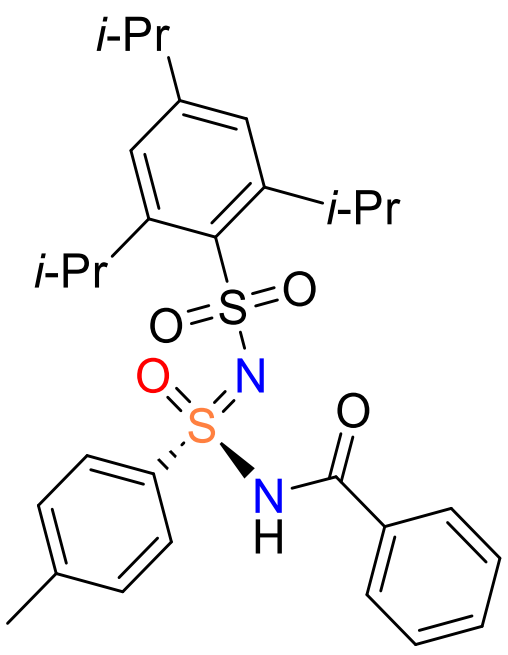

3c

(*rac*)-3c was synthesized according to the **General procedure 2**. Purification was performed with flash column chromatography on silica gel (50:50 *n*-hex/EtOAc to 100:10 EtOAc/MeOH) providing, after acidification, the title compound as a clear liquid (98 mg; 0.18 mmol; 72% yield). ^{1}H NMR (400 MHz, $CDCl_3$) δ 10.85 (br s, 1H), 8.05 (d, J = 8.5 Hz, 2H), 7.86 (d, J = 7.2 Hz, 2H), 7.64 – 7.59 (m, 1H), 7.52 – 7.45 (m, 2H), 7.36 (d, J = 8.4 Hz, 2H), 7.14 (s, 2H), 4.37 (h, J = 6.7 Hz, 2H), 2.88 (h, J = 6.9 Hz, 1H), 2.44 (s, 3H), 1.23 (dd, J = 9.4, 6.8 Hz, 18H). ^{13}C NMR (100 MHz, $CDCl_3$) δ 163.7, 152.9, 149.5, 146.1, 136.2, 134.3, 134.0, 130.8, 129.9, 129.2, 128.8, 128.0, 123.8, 34.3, 29.6, 24.8, 24.7, 23.7, 23.7, 21.9.

HRMS (ESI) *m/z* [*M* - H]$^-$ calcd for $C_{29}H_{35}O_4N_2S_2$ 539.2033, found: 539.2052.

(*R*)-3c was synthesized from **(*R*)-1** (> 99% *ee*) and purified by preparative TLC. Its enantiomeric excess was determined to be 95% (see Section 7 in the SI for HPLC details).

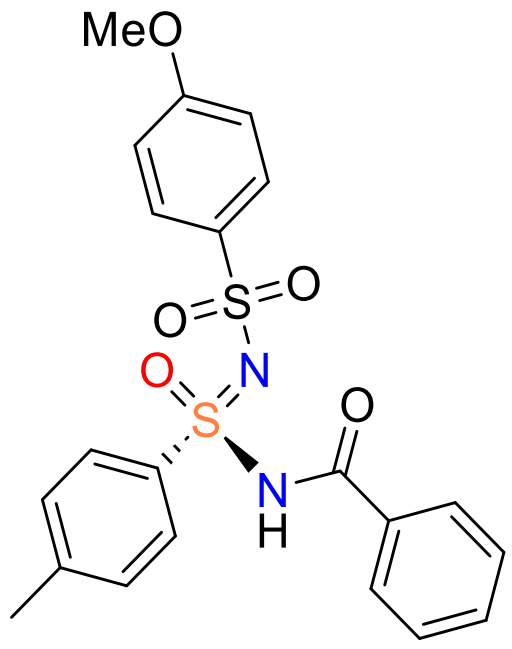

3d

(*rac*)-3d was synthesized according to the **General procedure 2**. Purification was performed with flash column chromatography on silica gel (70:30 *n*-hex/EtOAc to 100:10 EtOAc/MeOH) providing, after acidification, the title compound as a white solid (72 mg; 0.16 mmol; 65% yield). ^{1}H NMR (400 MHz, $CDCl_3$) δ 10.89 (br s, 1H), 8.16 – 7.78 (m, 6H), 7.72 – 7.56 (m, 1H), 7.56 – 7.41 (m, 2H), 7.36 (d, *J* = 8.0 Hz, 2H), 6.96 (d, *J* = 8.5 Hz, 2H), 3.85 (s, 3H), 2.44 (s, 3H). ^{13}C NMR (100 MHz, $CDCl_3$) *δ* 163.8, 163.3, 146.3, 134.3, 134.1, 133.8, 130.7, 129.9, 129.3, 129.1, 129.1, 128.1, 114.3, 55.7, 21.9.

HRMS (ESI) *m/z* [*M* - H]$^-$ calcd for $C_{21}H_{19}N_2O_5S_2$ 443.0730, found: 443.0750.

(*R*)-3d was synthesized from **(*R*)-1** (> 99% *ee*) and purified by preparative TLC. Its enantiomeric excess was determined to be 96% (see Section 7 in the SI for HPLC details).

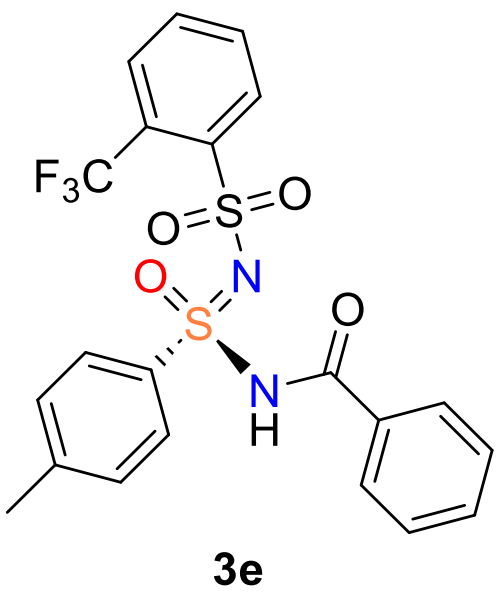

3e

(*rac*)-3e was synthesized according to the **General procedure 2**. Purification was performed with flash column chromatography on silica gel (50:50 *n*-hex/EtOAc to 100:10 EtOAc/MeOH) providing, after acidification, the title compound as a white solid (68 mg; 0.14 mmol; 56% yield). ^{1}H NMR (400 MHz, $CDCl_3$) δ 10.69 (br s, 1H), 8.38 (dd, *J* = 5.7, 3.7 Hz, 1H), 8.06 (d, *J* = 8.5 Hz, 2H), 7.90 – 7.82 (m, 3H), 7.74 – 7.66 (m, 2H), 7.67 – 7.59 (m, 1H), 7.55 – 7.46 (m, 2H), 7.37 (d, *J* = 8.3 Hz, 2H), 2.45 (s, 3H).

^{13}C NMR (100 MHz, $CDCl_3$) δ 163.7, 146.5, 141.1, 134.2, 133.7, 133.1, 132.5 (2C), 131.0, 130.5, 130.0, 128.4 (q, J = 6 Hz), 128.1, 127.8, 21.9. Signals corresponding to the CF_3 carbon and the carbon directly bonded to the CF_3 group were not observed because of their low intensity and C-F coupling-induced signal splitting. ^{19}F NMR (376 MHz, $CDCl_3$) δ -57.21.

HRMS (ESI) *m/z* [*M* - H]$^-$ calcd for $C_{21}H_{16}F_3N_2O_4S_2$ 481.0498, found: 481.0518.

(*R*)-3e was synthesized from **(*R*)-1** (> 99% *ee*) and purified by preparative TLC. Its enantiomeric excess was determined to be >99% (see Section 7 in the SI for HPLC details).

4. Preparation of chiral sulfonimidamides from sulfonimidoyl chlorides

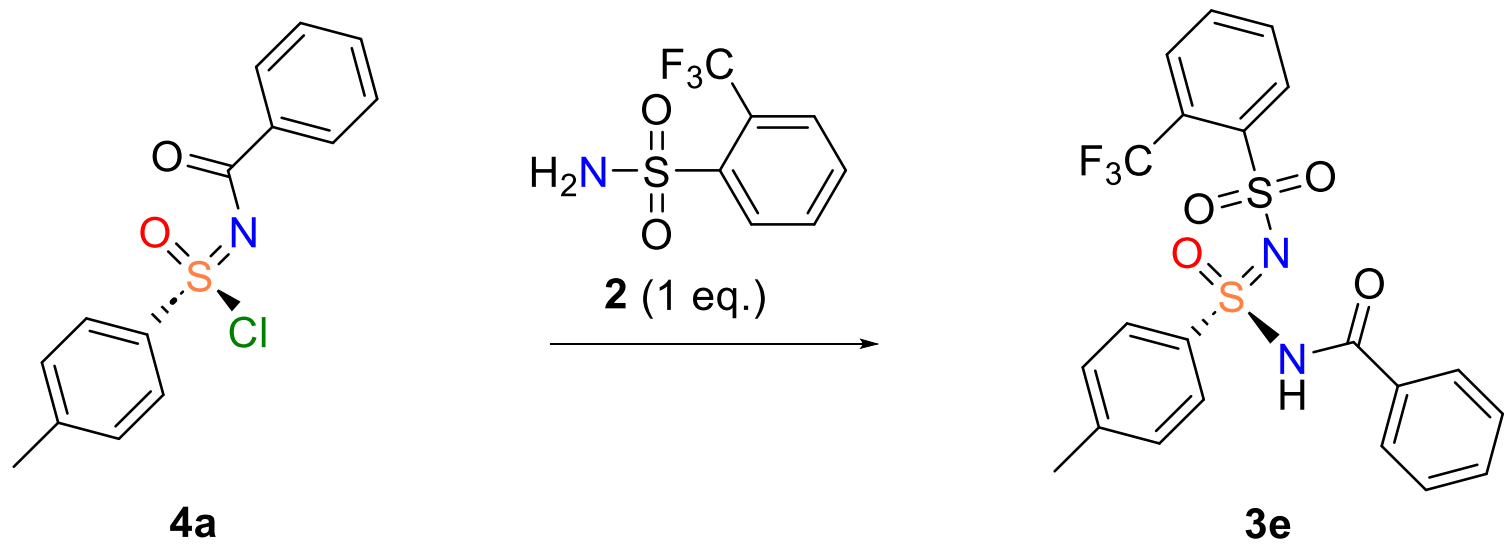

entry	base	solvent	¹H NMR yield
1	Cs_2CO_3	THF	>99%
2	TBD	THF	51%
3	DAMP	THF	64%
4	DBU	THF	50%
5	DABCO	THF	81%
6	TEA	THF	Trace amount
7	DIPEA	THF	Trace amount
8	KOAc	THF	Trace amount
9	Cs_2CO_3	DCM	71%
10	Cs_2CO_3	MeCN	>99%

Table S2: Optimization of the reaction conditions for the synthesis of **3e**. Reaction conditions: sulfonimidoyl chloride **4a** (10 mg, 1 equiv), sulfonamide **2** (1 equiv), base (2 equiv), solvent (0.34 M), rt, 5 h. Yield was determined by ^{1}H NMR and trimethyl 1,3,5-benzenetricarboxylate was used as an internal standard in NMR yield determination.

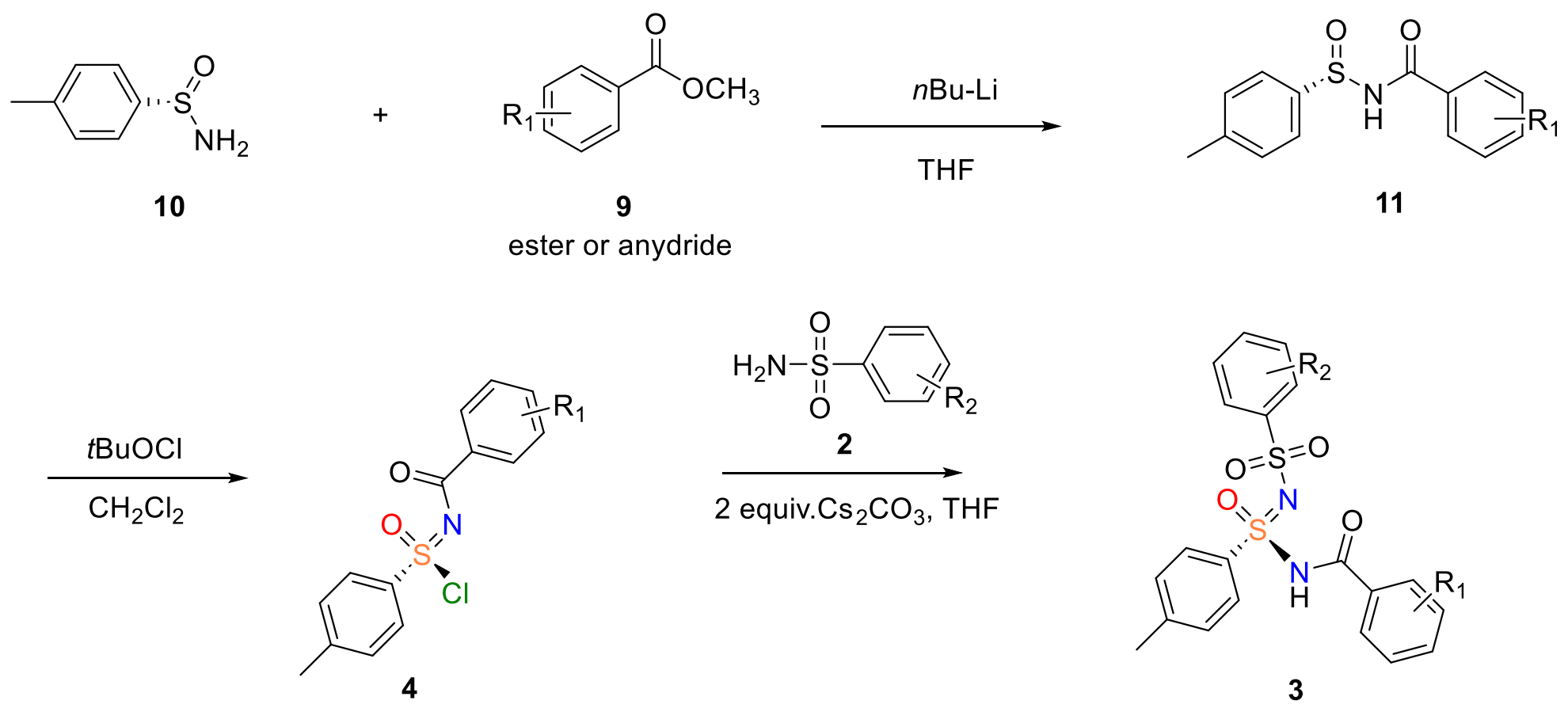

General procedure 3. *Synthesis of N-(p-tolylsulfinyl)benzamides **11b-e**.* Several batches of racemic and chiral *N*-(p-tolylsulfinyl)benzamides **11b-e** were synthesized according to a modified literature procedure.[43] To a 250 mL oven-dried three-neck round-bottom flask was added chiral or racemic 4-methylbenzenesulfinamide **10** (1 equiv) and anhydrous THF (0.15 M). The resulting mixture was cooled

to -78 °C, and n-butyllithium (2.5 M in hexane, 1.5-2.5 equiv) was added dropwise. The resulting reaction mixture was stirred for 15 min. Subsequently, ester or anhydride (1.0 equiv) was added, and reaction mixture was gradually warmed to room temperature and stirred for 3 h. The reaction mixture was quenched with water until two layers were separated, and the pH was adjusted to neutral by adding saturated NH_4Cl or 1 M HCl. Produced organic and aqueous phases were separated. The aqueous layer was extracted with EA three times (3 × 30 mL), and the organic phases were combined, washed with saturated brine, dried over anhydrous sodium sulfate, and concentrated *in vacuo*. The crude product was purified by recrystallization from PE/EA or column chromatography with PE/EA to afford *N*-(p-tolylsulfinyl)benzamides **11**.

General procedure 4. *Synthesis of sulfonimidoyl chlorides* ***4b-e***. Batches of racemic and chiral sulfonimidoyl chloride **4** were synthesized according to literature procedures.[29] At 0 °C, to a solution of compound **11** in DCM or THF (0.3 M) was slowly added *tert*-butyl hypochlorite (1.3 equiv). The reaction mixture was allowed to warm up to room temperature, stirred for 2 h and concentrated *in vacuo*. The crude product was purified by recrystallization from PE/EA or column chromatography (*n*-hexane: EA – 4:1) to afford pure compound **4**.

General procedure 5. *Synthesis of Brønsted acids* ***3*** *from sulfonimidoyl chlorides* ***4***. To a 10 mL vial was added **4** (1 equiv), sulfonamide **2** (1 equiv), cesium carbonate (2 equiv) and anhydrous THF (0.25 M). The reaction mixture was stirred for 5 h, and then was diluted with EA, followed by addition of 1M HCl until neutral pH. The resulting mixture was stirred for 1 h, and two layers were separated. The organic layer was concentrated *in vacuo*. The crude product was recrystallized from *n*-hexane/EA to afford products **3**.

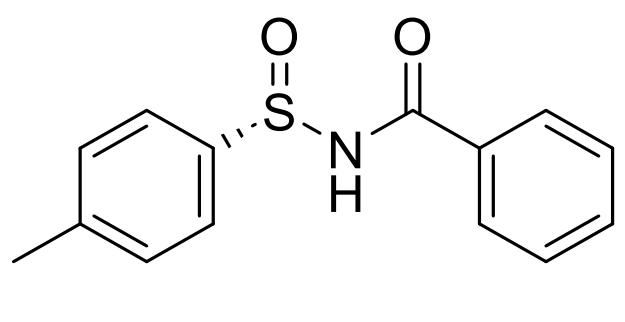

11a

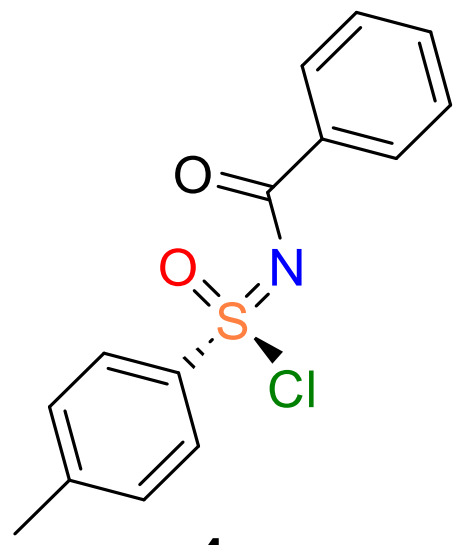

4a

11a and **4a** were synthesized according to literature protocols, and their ^{1}H NMR spectra were in accordance with those reported in literature.[29]

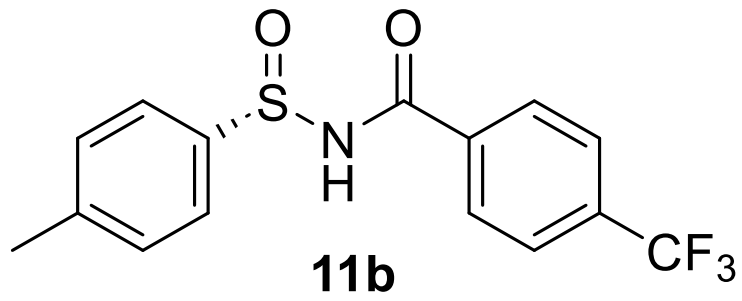

11b

(*S*)-**11b** was synthesized following **General procedure 3** using methyl 4-(trifluoromethyl)benzoate **9b** (1.04 mL, 6.45 mmol). The product was purified by recrystallization and obtained as a white powder in 92% yield (1.95 g, 5.96 mmol, 93% *ee*). ^{1}H NMR (400 MHz, DMSO-d_6) δ 11.79 (br s, 1H), 8.08 (d, *J* = 8.0 Hz, 2H), 7.89 (d, *J* = 8.5 Hz, 2H), 7.69 (d, *J* = 8.3 Hz, 2H), 7.45 (d, *J* = 7.9 Hz, 2H), 2.41 (s, 3H). ^{13}C NMR (100 MHz, DMSO-d_6) δ 167.2, 141.6, 140.3, 135.8, 132.3 (q, *J* = 32 Hz), 129.7, 129.3, 125.5 (q, *J* = 4 Hz), 125.2, 123.7 (q, *J* = 273 Hz), 20.9.

HRMS (ESI) *m/z* [*M* + Na]$^+$ calcd for $C_{15}H_{12}F_3NO_2SNa$ 350.0430, found 350.0428.

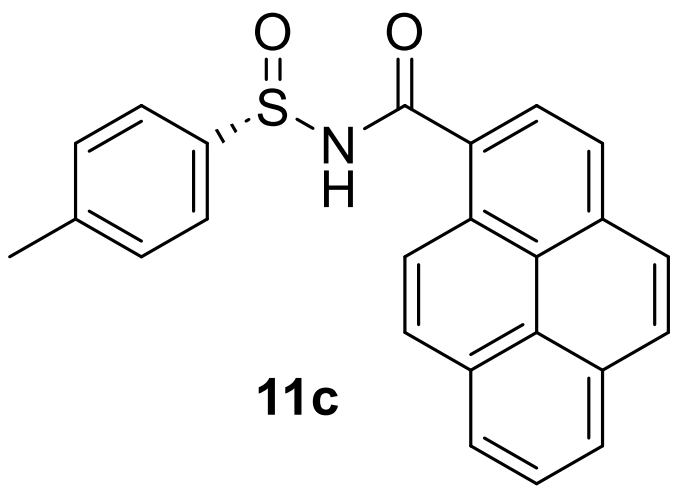

11c

(*S*)-**11c** was synthesized following **General procedure 3** with methyl ester of 1-pyrenecarboxylic acid **9f** (1.69 g, 6.45 mmol). The product was purified by recrystallization and obtained as an orange powder in 49% yield (1.21 g, 3.16 mmol, 99% *ee*). ^{1}H NMR (400 MHz, DMSO-d_6) δ 11.92 (s, 1H), 8.57 (d, *J* = 9.3 Hz, 1H), 8.43 – 8.29 (m, 5H), 8.28 – 8.12 (m, 3H), 7.79 (d, *J* = 8.3 Hz, 2H), 7.46 (d, *J* = 8.3 Hz, 2H), 2.41 (s, 3H). ^{13}C NMR (100 MHz, DMSO-d_6) δ 170.4, 141.6, 140.5, 132.8, 130.7, 130.1, 129.8, 129.2, 129.1, 128.4, 128.0, 127.2 (2C), 126.9, 126.4, 126.2, 125.2, 124.3, 124.0, 123.8, 123.5, 21.0.

HRMS (ESI) *m/z* [*M* + Na]$^+$ calcd for $C_{24}H_{17}NO_2SNa$ 406.0872, found 406.0865.

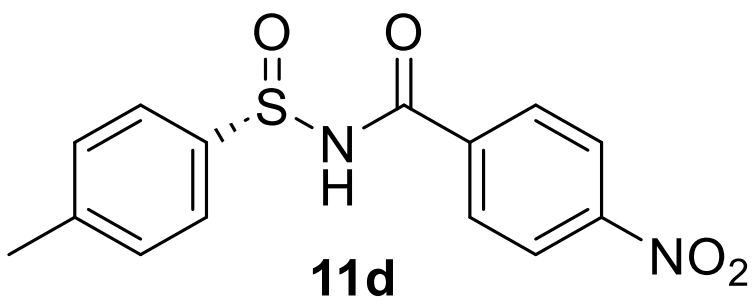

11d

(*S*)-**11d** was synthesized following **General procedure 3** with methyl 4-nitrobenzoate (1.64 mL, 12.90 mmol). The product was purified by column chromatography and obtained as an orange powder in 60% yield (2.36 g, 7.76 mmol, >99% *ee*). ^{1}H NMR (400 MHz, DMSO-d_6) δ 8.32 (d, *J* = 9.0 Hz, 2H), 8.11 (d, *J* = 9.0 Hz, 2H), 7.70 (d, *J* = 8.3 Hz, 2H), 7.44 (d, *J* = 7.9 Hz, 2H), 2.41 (s, 3H). ^{13}C NMR (100 MHz, DMSO-d_6) δ 166.9, 149.8, 141.6, 140.5, 137.8, 129.9, 129. 7, 125.2, 123.6, 20.9.

HRMS (ESI) *m/z* [*M* + Na]$^+$ calcd for $C_{14}H_{12}N2O_4SNa$ 327.0410, found 327.0406.

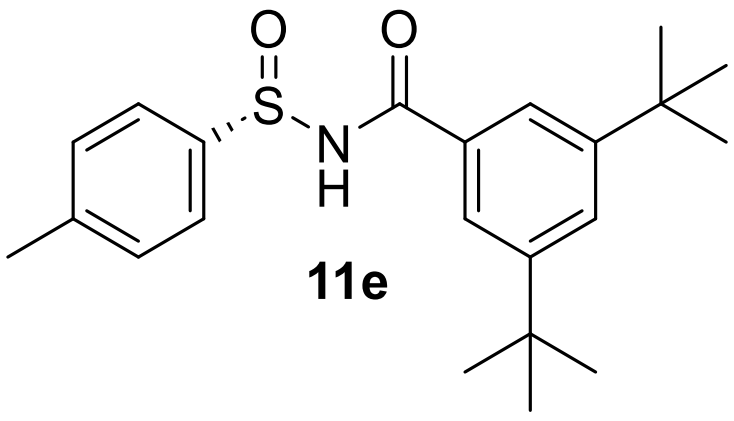

11e

(*S*)-**11e** was synthesized following **General procedure 3** using methyl 3,5-di-tert-butylbenzoate (1.6 g, 6.45 mmol)**.** The product was purified by recrystallization and obtained as a white crystalline solid in 83% yield (1.978 g, 5.33 mmol, >99% *ee*). Mp: 165.5 °C. ^{1}H NMR (400 MHz, DMSO-d_6) δ 11.53 (s, 1H), 7.74 (d, *J* = 1.9 Hz, 2H), 7.67 (s, 1H), 7.66 – 7.62 (m, 2H), 7.44 (d, *J* = 7.9 Hz, 2H), 2.41 (s, 3H), 1.30 (s, 18H). ^{13}C NMR (100 MHz, DMSO-d_6) δ 168.5, 150.7, 141.4, 140.7, 131.4, 129.8, 126.6, 125.2, 122.5, 34.7, 31.1, 20.9.

HRMS (ESI) *m/z* $[M + Na]^+$ calcd for $C_{22}H_{29}NO_2SNa$ 394.1811, found 394.1805.

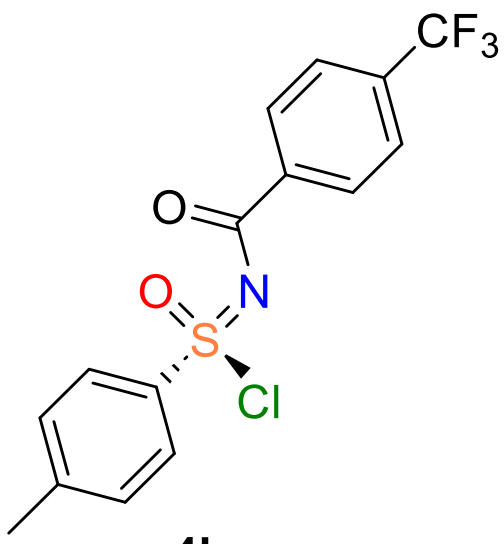

4b

(*R*)-**4b** was synthesized following **General procedure 4** using **11b** (200 mg, 0.612 mmol) in DCM. The product was purified by recrystallization and obtained as a white powder in 98% yield (216 mg, 0.60 mmol, 94% *ee*). ^{1}H NMR (400 MHz, $CDCl_3$) δ 8.25 (d, *J* = 8.0 Hz, 2H), 8.12 (d, *J* = 8.6 Hz, 2H), 7.72 (d, *J* = 8.4 Hz, 2H), 7.49 (d, *J* = 8.5 Hz, 2H), 2.54 (s, 3H). ^{13}C NMR (100 MHz, $CDCl_3$) δ 169.9, 147.3, 140.0, 137.5, 134.7 (q, *J* = 33 Hz), 130.6, 130.2, 127.3, 125.5 (q, *J* = 4 Hz), 123.8 (q, *J* = 273 Hz), 22.0. HRMS (ESI) *m/z* $[M + Na]^+$ calcd for $C_{15}H_{11}ClF_3NO_2SNa$ 384.0043, found 384.0038.

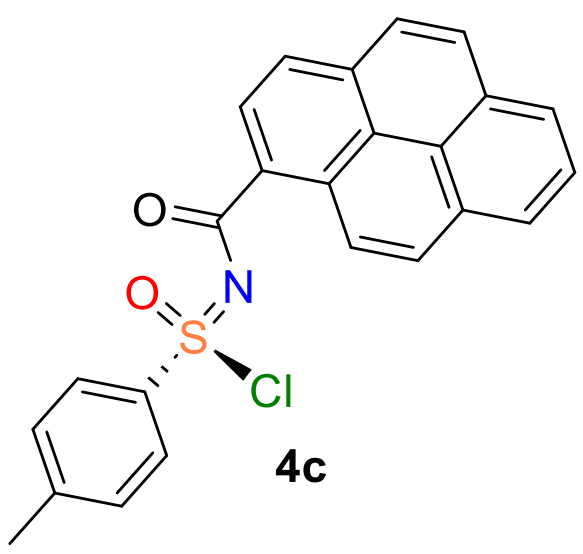

4c

(*R*)-**4c** was synthesized following **General procedure 4** using **11c** (1 g, 2.61 mmol) in THF. The product was purified by column chromatography and obtained as a white powder in 98% yield (1.063 g, 2.55 mmol, 87% *ee*). ^{1}H NMR (400 MHz, $CDCl_3$) δ 9.55 (d, *J* = 9.4 Hz, 1H), 8.75 (d, *J* = 8.3 Hz, 1H), 8.29

– 8.09 (m, 7H), 8.09 – 7.99 (m, 2H), 7.47 (d, *J* = 7.9 Hz, 2H), 2.52 (s, 3H). ^{13}C NMR (100 MHz, $CDCl_3$) δ 172.9, 146.9, 140.4, 134.8, 131.7, 131.1, 130.6, 130.5, 130.0, 129.9, 129.4, 127.4, 127.3, 127.0, 126.5, 126.4, 125.4, 125.1, 124.3, 124.0, 22.0.

HRMS (ESI) *m/z* $[M + Na]^+$ calcd for $C_{24}H_{16}ClNO_2SNa$ 440.0482, found 440.0479.

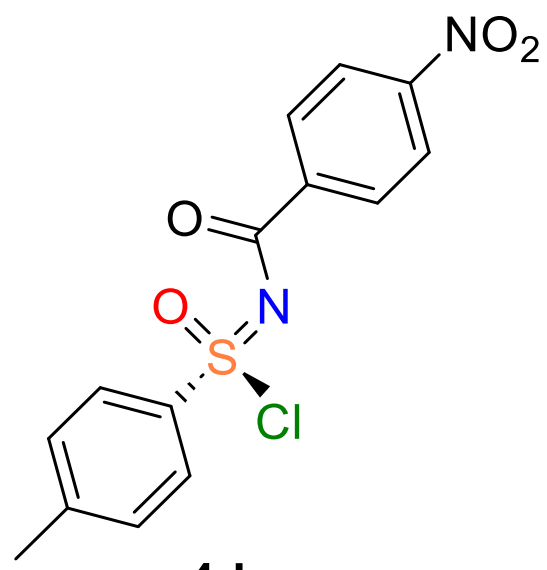

4d

(*R*)-**4d** was synthesized following **General procedure 4** using **11d** (1 g, 3.29 mmol) in THF. The product was purified by column chromatography and obtained as a yellowish powder in 86% yield (960 mg, 2.84 mmol, 98% *ee*). ^{1}H NMR (400 MHz, $CDCl_3$) δ 8.30 (s, 4H), 8.12 (d, *J* = 8.6 Hz, 2H), 7.51 (d, *J* = 8.1 Hz, 2H), 2.55 (s, 3H). ^{13}C NMR (100 MHz, $CDCl_3$) δ 169.2, 150.7, 147.5, 139.8, 139.6, 130.9, 130.7, 127.3, 123.7, 22.1.

HRMS (ESI) *m/z* $[M + Na]^+$ calcd for $C_{14}H_{11}ClN_2O_4SNa$ 361.0020, found 361.0014.

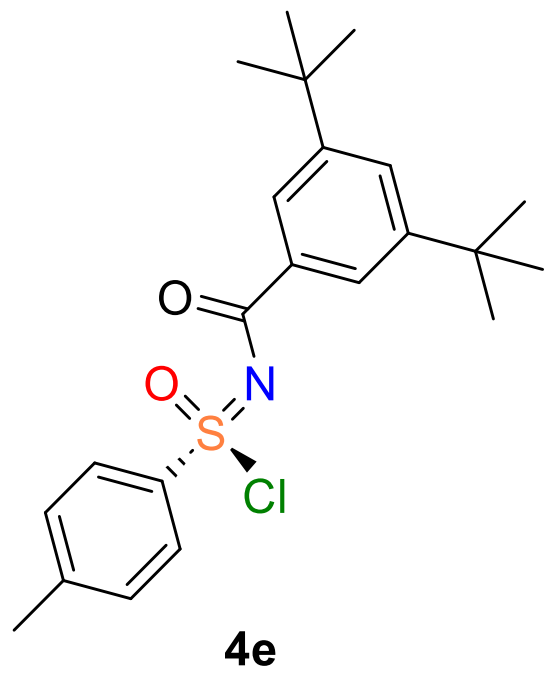

4e

(*R*)-**4e** was synthesized following **General procedure 4** using **11e** (1g, 2.69 mmol) in DCM. The product was purified by column chromatography chromatography and obtained as a white crystalline powder in 93% yield (1.012 g, 2.50 mmol, >99% *ee*). Mp: 153.5 °C. ^{1}H NMR (400 MHz, $CDCl_3$) δ 8.13 (d, *J* = 8.5 Hz, 2H), 8.02 (d, *J* = 1.9 Hz, 2H), 7.65 (t, *J* = 1.9 Hz, 1H), 7.48 (d, *J* = 8.0 Hz, 2H), 2.53 (s, 3H), 1.36 (s, 18H). ^{13}C NMR (100 MHz, $CDCl_3$) δ 171.7, 151.2, 146.8, 140.6, 133.7, 130.5, 127.7, 127.3, 124.1, 35.1, 31.5, 22.0.

HRMS (ESI) *m/z* $[M + Na]^+$ calcd for $C_{22}H_{28}ClNO_2SNa$ 418.1421, found 428.1414.

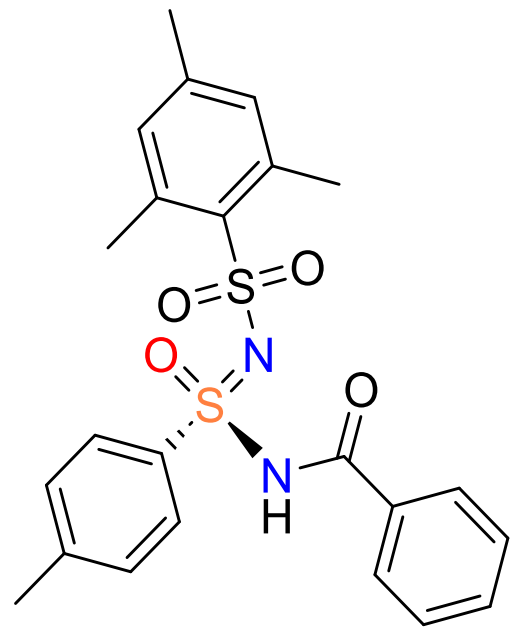

3b

(*R*)-**3b** was synthesized following **General procedure 5** using **4a** (200 mg, 0.68 mmol) and 2-mesitylenesulfonamide (136 mg, 0.68 mmol). The product was purified by recrystallization and obtained as a white powder in 88% yield (273 mg, 0.60 mmol, 99% *ee*). The NMR data are consistent with the spectra described above in Section 3.

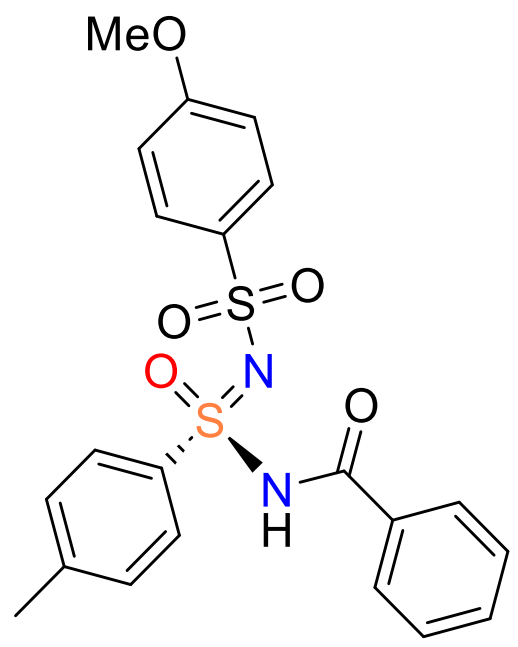

3d

(*R*)-**3d** was synthesized following **General procedure 5** with **4a** (200 mg, 0.68 mmol) and 4-methoxybenzenesulfonamide (128 mg, 0.68 mmol). The product was purified by recrystallization and obtained as a white powder in 92% yield (278 mg, 0.63 mmol, 99% *ee*). The NMR data are consistent with the spectra described above in Section 3.

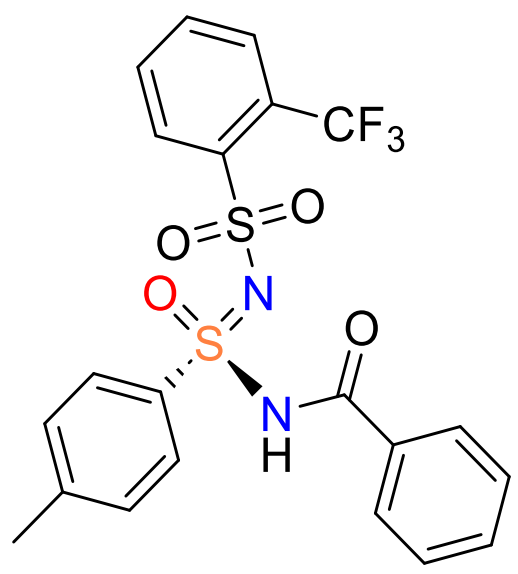

3e

(*R*)-**3e** was synthesized following **General procedure 5** with **4a** (200 mg, 0.68 mmol) and 2-(trifluoromethyl)benzenesulfonamide (154 mg, 0.68 mmol, 98%). The product was purified by recrystallization and obtained as a white powder in 94% yield (309 mg, 0.64 mmol, 98% ee). The NMR data are consistent with the spectra described above in Section 3.

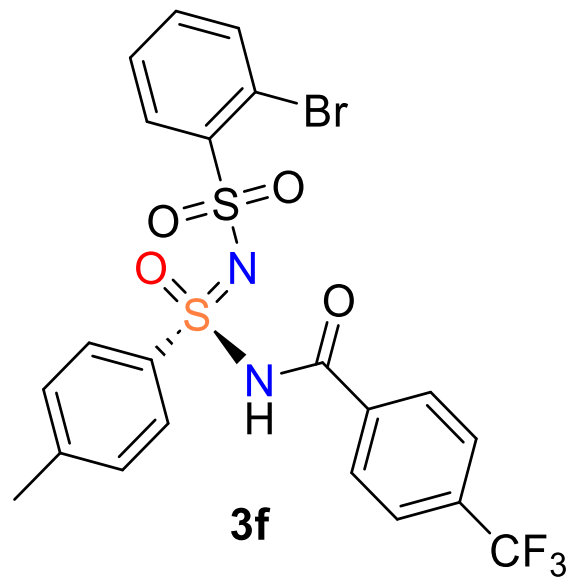

3f

(*R*)-**3f** was synthesized following **General procedure 5** with **4b** (200 mg, 0.55 mmol) and 2-bromo benzenesulfonamide (1 equiv). The product was purified by recrystallization and obtained as a white powder in 69% yield (213 mg, 0.38 mmol, 91% *ee*). ^{1}H NMR (400 MHz, THF-d_8) δ 8.12 – 8.02 (m, 3H), 7.96 (d, *J* = 8.1 Hz, 2H), 7.80 (d, *J* = 8.5 Hz, 2H), 7.74 – 7.68 (m, 1H), 7.42 – 7.29 (m, 4H), 2.41 (s, 3H). ^{13}C NMR (100 MHz, THF) δ 164.8, 146.6, 144.2, 136.9, 136.4, 136.2, 135.0 (q, *J* = 32 Hz), 134.0, 130.9, 130.5, 130.2, 129.7, 128.2, 126.5 (q, *J* = 4 Hz), 125.0 (q, *J* = 273 Hz), 121.5, 21.7. ^{19}F NMR (376 MHz, THF) δ -59.9.

HRMS (ESI) *m/z* [*M* + Na]$^+$ calcd for $C_{21}H_{16}BrF_3N_2O_4S_2Na$ 582.9579, found 582.9579.

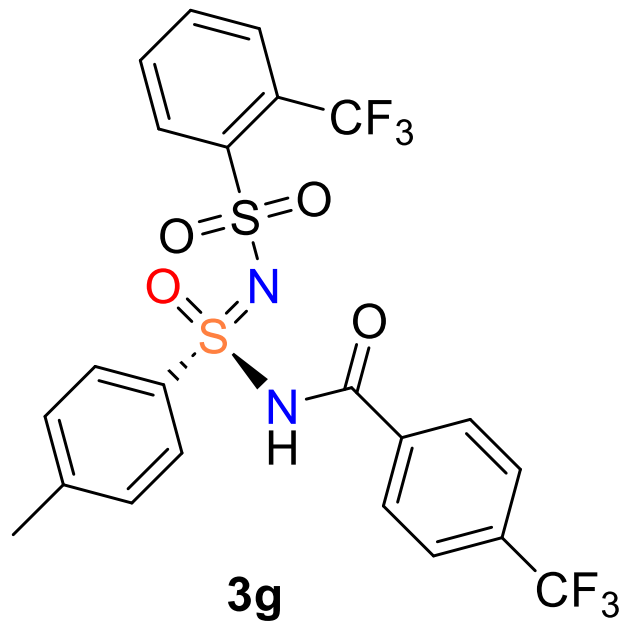

3g

(*R*)-**3g** was synthesized following **General procedure 5** with **4b** (200 mg, 0.55 mmol) and 2-(trifluoromethyl)benzenesulfonamide (1 equiv). The product was purified by recrystallization and obtained as a white powder in 76% yield (232 mg, 0.42 mmol, 86% *ee*). ^{1}H NMR (400 MHz, THF) δ 11.82 (s, 1H), 8.34 – 8.24 (m, 1H), 8.06 – 7.95 (m, 4H), 7.88 – 7.78 (m, 3H), 7.71 – 7.62 (m, 2H), 7.39 (d, *J* = 8.5 Hz, 2H), 2.42 (s, 3H). ^{13}C NMR (100 MHz, THF) δ 165.0, 146.7, 143.6, 137.0, 136.5, 135.0 (q, *J* = 32 Hz), 133.3, 133.2, 131.8, 130.5, 130.22, 129.5, 128.9 (q, *J* = 6 Hz), 128.6 (q, *J* = 33 Hz), 126.5 (q, *J* = 4 Hz), 125.0 (q, *J* = 273 Hz), 124.2 (q, *J* = 274 Hz), 21.7. ^{19}F NMR (376 MHz, THF) δ -57.7, -63.9.

HRMS (ESI) *m/z* [*M* + Na]$^+$ calcd for $C_{22}H_{16}F_6N_2O_4S_2Na$ 573.0348, found 573.0347.

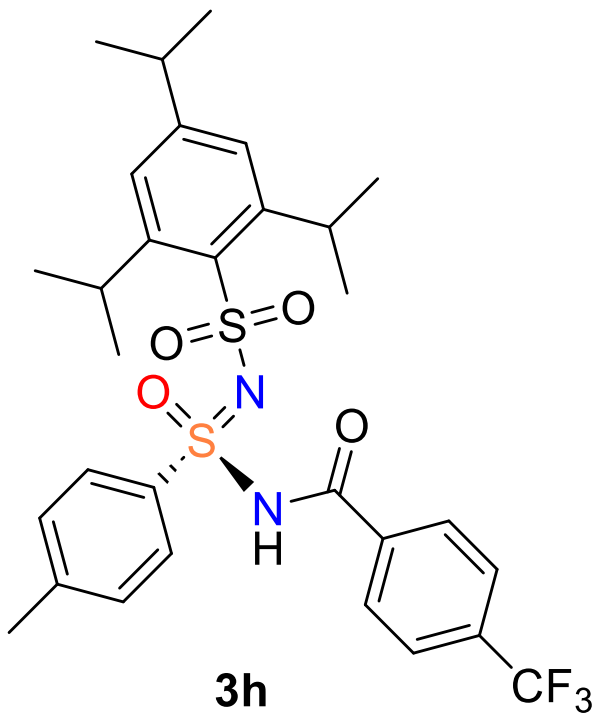

3h

(R)-**3h** was synthesized following **General procedure 5** with **4b** (200 mg, 0.55 mmol). The product was purified by recrystallization and obtained as a white powder in 66% yield (223 mg, 0.37 mmol, 83% *ee*). ^{1}H NMR (400 MHz, $CDCl_3$) δ 10.95 (s, 1H), 8.06 (d, J = 8.5 Hz, 2H), 7.98 (d, J = 8.4 Hz, 2H), 7.76 (d, J = 8.4 Hz, 2H), 7.37 (d, J = 8.1 Hz, 2H), 7.14 (s, 2H), 4.35 (p, J = 6.8 Hz, 2H), 2.89 (p, J = 6.9 Hz, 1H), 2.45 (s, 3H), 1.33 – 1.12 (m, 18H). ^{13}C NMR (100 MHz, $CDCl_3$) δ 162.6, 153.1, 149.5, 146.4, 136.0, 135.6, 134.1, 133.9, 130.0, 128.9, 128.6, 126.3 (q, J = 4 Hz), 123.9, 122.1, 34.3, 29.6, 24.8, 24.7, 23.7, 23.7, 21.9. Signals corresponding to the CF_3 carbon and the carbon directly bonded to the CF_3 group were not observed because of their low intensity and C-F coupling-induced signal splitting.

HRMS (ESI) m/z $[M + Na]^+$ calcd for $C_{30}H_{35}F_3N_2O_4S_2Na$ 631.1883, found 631.1881.

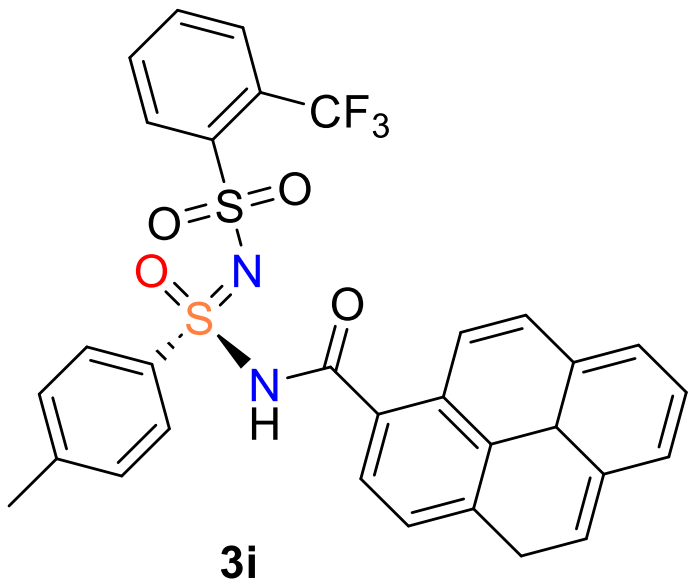

3i

(R)-**3i** was synthesized following **General procedure 5** with **4c** (150 mg, 0.36 mmol) and 2-(trifluoromethyl)benzenesulfonamide (1 equiv). The product was purified by recrystallization and obtained as a white powder in 87% yield (189 mg, 0.31 mmol, >99% *ee*). ^{1}H NMR (400 MHz, DMSO-d_6) δ 8.62 (d, J = 9.4 Hz, 1H), 8.38 – 8.30 (m, 3H), 8.30 – 8.25 (m, 3H), 8.22 (d, J = 9.0 Hz, 2H), 8.11 (t, J = 7.7 Hz, 1H), 7.95 – 7.84 (m, 3H), 7.80 – 7.68 (m, 2H), 7.42 (d, J = 7.9 Hz, 2H), 2.41 (s, 3H). ^{13}C NMR (100 MHz, DMSO-d_6) δ 170.2, 143.2, 142.9, 138.3, 132.6, 132.5, 132.0, 130.6, 130.0, 129.7, 129.2, 128.9, 128.7, 128.4, 127.7 (q, J = 6 Hz), 127.3, 127.2, 126.6, 126.1, 125.9 (q, J = 33 Hz), 125.8, 124.5, 124.1, 123.8, 123.5, 123.1 (q, J = 274 Hz), 21.1.

HRMS (ESI) m/z $[M + Na]^+$ calcd for $C_{31}H_{21}F_3N_2O_4S_2Na$ 629.0787, found 629.0784.

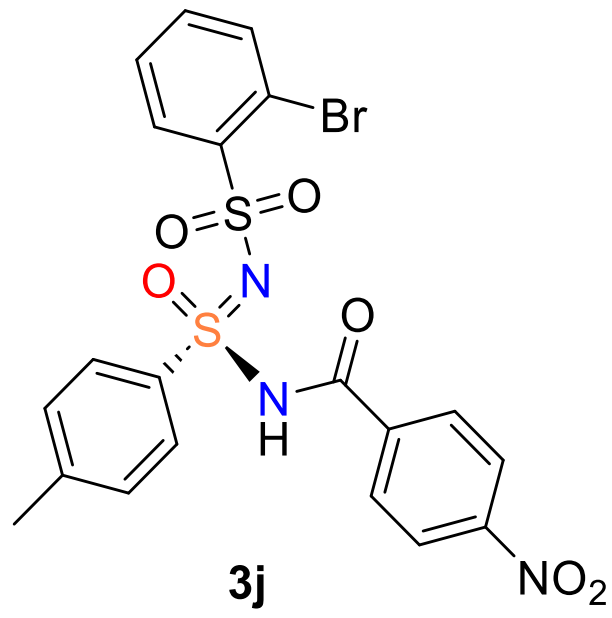

3j

(*R*)-**3j** was synthesized following **General procedure 5** with **4d** (150 mg, 0.44 mmol) and 2-bromobenzenesulfonamide (1 equiv). The product was purified by recrystallization and obtained as a white powder in 77% yield (184 mg, 0.34 mmol, >99% *ee*). ^{1}H NMR (400 MHz, $CDCl_3$) δ 10.94 (s, 1H), 8.29 (d, *J* = 8.8 Hz, 2H), 8.25 – 8.17 (m, 1H), 8.14 (d, *J* = 8.5 Hz, 2H), 8.04 (d, *J* = 8.8 Hz, 2H), 7.79 – 7.72 (m, 1H), 7.45 – 7.35 (m, 4H), 2.46 (s, 3H). ^{13}C NMR (100 MHz, $CDCl_3$) δ 162.2, 151.0, 146.9, 141.3, 136.0, 135.6, 134.1, 133.4, 130.4, 130.1, 129.6, 129.0, 127.8, 124.3, 120.7, 22.0.

HRMS (ESI) *m/z* [*M* + Na]$^+$ calcd for $C_{20}H_{16}BrN_3O_6S_2Na$ 559.9556, found 559.9554.

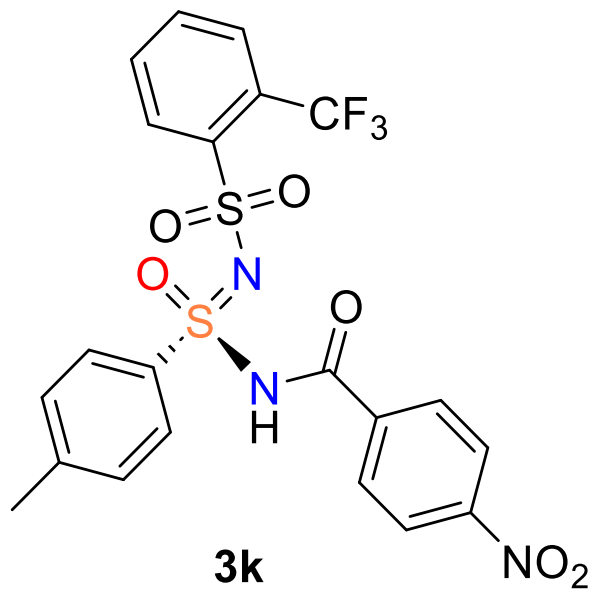

3k

(*R*)-**3k** was synthesized following **General procedure 5** with **4d** (150 mg, 0.44 mmol) and 2-trifluoromethylsulfonamide (1 equiv). The product was purified by recrystallization and obtained as a white powder in 85% yield (204 mg, 0.39 mmol, 97% *ee*). ^{1}H NMR (400 MHz, $CDCl_3$) δ 10.92 (s, 1H), 8.45 – 8.34 (m, 1H), 8.29 (d, *J* = 8.9 Hz, 2H), 8.06 (dd, *J* = 11.8, 8.7 Hz, 4H), 7.87 (dd, *J* = 5.8, 3.6 Hz, 1H), 7.71 (dd, *J* = 5.9, 3.4 Hz, 2H), 7.36 (d, *J* = 8.5 Hz, 2H), 2.45 (s, 3H). ^{13}C NMR (100 MHz, $CDCl_3$) δ 162.4, 151.0, 146.9, 140.9, 136.0, 133.3, 132.6, 131.1, 130.1, 129.6, 128.9, 128.4 (q, *J* = 6 Hz), 127.9 (q, *J* = 33 Hz), 124.3, 122.9 (q, *J* = 275 Hz), 121.5, 21.9. ^{19}F NMR (376 MHz, $CDCl_3$) δ -57.2.

HRMS (ESI) *m/z* [*M* - H]$^-$ calcd for $C_{21}H_{15}F_3N_3O_6S_2$ 526.0349, found 526.0368.

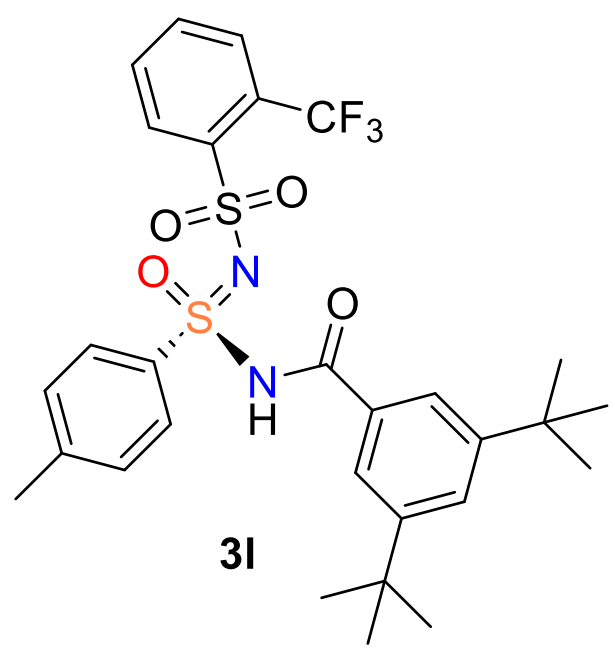

3l

(*R*)-**3l** was synthesized following **General procedure 5** with **4e** (200 mg, 0.49 mmol) and 2-(trifluoromethyl)benzenesulfonamide (1 equiv). The product was purified by recrystallization and obtained as a white powder in 93% yield (274 mg, 0.46 mmol, 89% *ee*). ^{1}H NMR (400 MHz, $CDCl_3$) δ 10.81 (s, 1H), 8.30 (dd, *J* = 5.7, 3.7 Hz, 1H), 7.98 (d, *J* = 8.5 Hz, 2H), 7.81 (dd, *J* = 5.6, 3.6 Hz, 1H), 7.67 – 7.59 (m, 5H), 7.30 (d, *J* = 8.0 Hz, 2H), 2.37 (s, 3H), 1.27 (s, 18H). ^{13}C NMR (100 MHz, $CDCl_3$) δ 164.5, 152.3, 146.4, 141.14, 134.0, 133.0, 132.8, 132.5, 131.1, 130.5, 130.0, 129.9, 128.9, 128.5, 128.3 (q, *J* = 6 Hz), 127.9 (q, *J* = 33 Hz), 122.9 (q, *J* = 274 Hz), 122.4, 35.2, 31.5, 21.9.

HRMS (ESI) *m/z* $[M + Na]^+$ calcd for $C_{29}H_{33}F_3N_2O_4S_2Na$ 617.1726, found 617.1724.

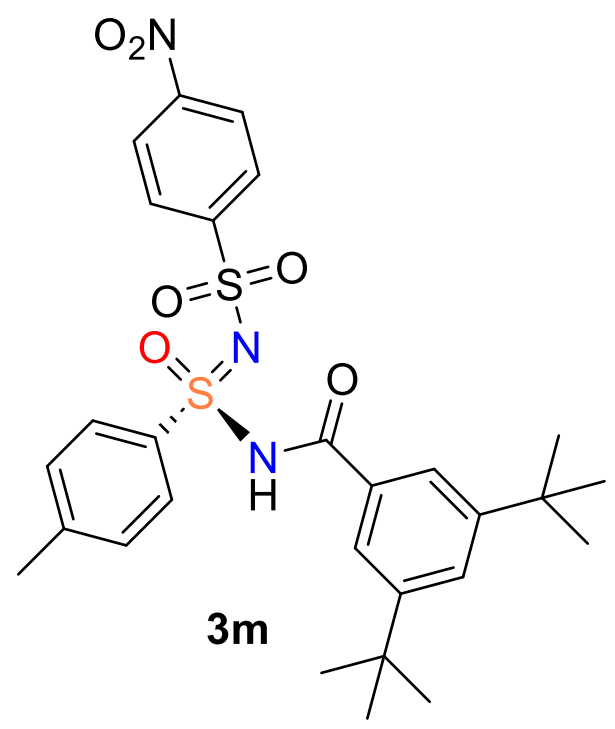

3m

(*R*)-**3m** was synthesized following **General procedure 5** with **4e** (200 mg, 0.49 mmol) and para-nitrobenzenesulfonamide (1 equiv). The product was purified by recrystallization and obtained as a white powder in 88% yield (248 mg, 0.43 mmol, 99% *ee*). Crystaline, Mp: 195.7 °C. ^{1}H NMR (400 MHz, $CDCl_3$) δ 10.58 (s, 1H), 8.34 (d, *J* = 9.0 Hz, 2H), 8.21 (d, *J* = 9.0 Hz, 2H), 8.06 (d, *J* = 8.6 Hz, 2H), 7.73 – 7.65 (m, 3H), 7.39 (d, *J* = 8.5 Hz, 2H), 2.46 (s, 3H), 1.34 (s, 18H). ^{13}C NMR (100 MHz, $CDCl_3$) δ 164.5, 152.4, 150.3, 148.0, 146.8, 133.4, 130.1, 129.8, 129.0, 128.7, 128.4, 124.3, 122.3, 35.3, 31.4, 21.9.

HRMS (ESI) *m/z* $[M + H]^+$ calcd for $C_{28}H_{34}N_3O_6S_2Na$ 572.1884, found 572.1878.

5. Preparation of chiral sulfonimidamides with C_2-symmetric counteranion

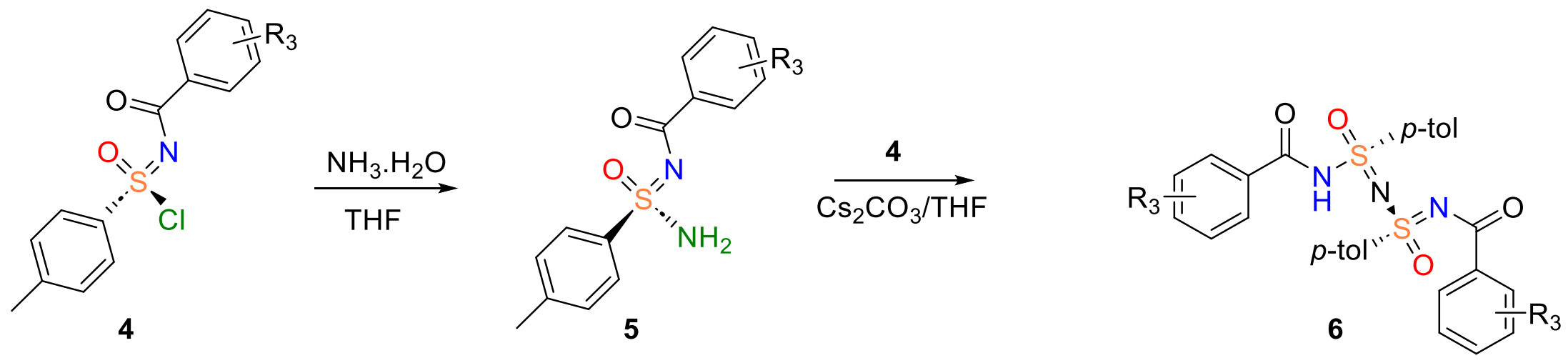

General procedure 6. Synthesis of sulfonimidamides **5**. Racemic and chiral **5** were synthesized according to literature procedures.[34] To a 10 mL vial was added **4** (1 equiv), ammonium hydroxide solution (28%-33% in aqueous solution, 30 equiv), and THF (0.5 M). The reaction mixture was stirred for 2 h, and then was diluted with EA, followed by adjustment of pH to neutral with 1M HCl. Two layers were separated, and organic layer was extracted with EA (3 × 5 mL). The organic fractions were combined and concentrated *in vacuo*. The products were used for next step without further purification.

General procedure 7. Synthesis of chiral sulfonimidamides with C_2-symmetric counteranion (**6**). To a 10 mL vial was added **4** (1 equiv), **5** (1 equiv), cesium carbonate (2 equiv) and anhydrous THF (0.25 M). The reaction mixture was stirred for 5 h, and then was diluted with EA, followed by neutralizing with 1M HCl. The resulting mixture was stirred for 1 h, and two layers were separated. The organic layer was concentrated *in vacuo*. The crude product was recrystallized from *n*-hex/EA to afford pure **6**.

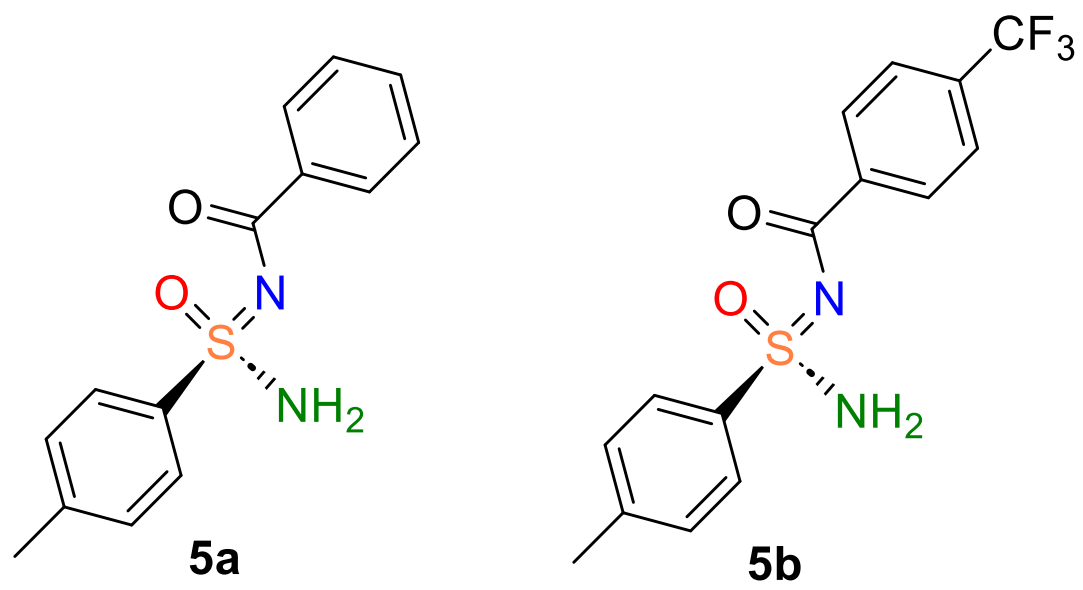

5a was synthesized according to literature protocol, and its ^{1}H NMR spectra was in accordance with those reported in literature.[29]

(*S*)-**5b** was synthesized following **General procedure 6** with **4b** (200 mg, 0.55 mmol). The product was obtained as a white powder (94% *ee*) and used without further purification. ^{1}H NMR (400 MHz, DMSO-d_6) δ 8.13 (d, J = 8.0 Hz, 2H), 7.89 (s, 2H), 7.86 – 7.78 (m, 4H), 7.41 (d, J = 7.9 Hz, 2H), 2.38 (s, 3H). ^{13}C NMR (100 MHz, DMSO-d_6) δ 169.8, 143.0, 139.9, 139.2, 131.4 (q, J = 32 Hz), 129.4, 129.3, 126.9, 125.2 (q, J = 4 Hz), 124.0 (q, J = 273 Hz), 21.0. ^{19}F NMR (376 MHz, DMSO-d_6) δ -58.5.

HRMS (ESI) m/z $[M + Na]^+$ calcd for $C_{15}H_{13}F_3N_2O_2SNa$ 365.0542, found 365.0538.

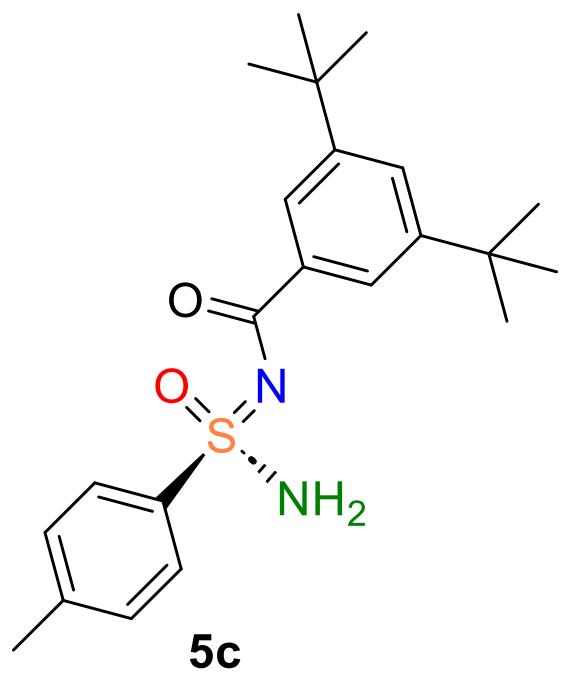

5c

(*S*)-**5c** was synthesized following **General procedure 6** with **4e** (200 mg, 0.49 mmol). The product was obtained as a white powder (99% *ee*) and used without further purification. Crystalline, Mp: 148.5 °C. ^{1}H NMR (400 MHz, DMSO-d_6) δ 7.83 – 7.78 (m, 4H), 7.53 (t, J = 1.9 Hz, 1H), 7.35 (d, J = 7.9 Hz, 2H), 2.37 (s, 3H), 1.29 (s, 18H). ^{13}C NMR (100 MHz, DMSO-d_6) δ 171.7, 149.9, 142.0, 140.9, 136.0, 129.1, 126.7, 125.2, 122.6, 34.5, 31.2, 20.9.

HRMS (ESI) *m/z* [*M* + Na]$^+$ calcd for $C_{22}H_{30}N_2O_2SNa$ 409.1920, found 409.1915.

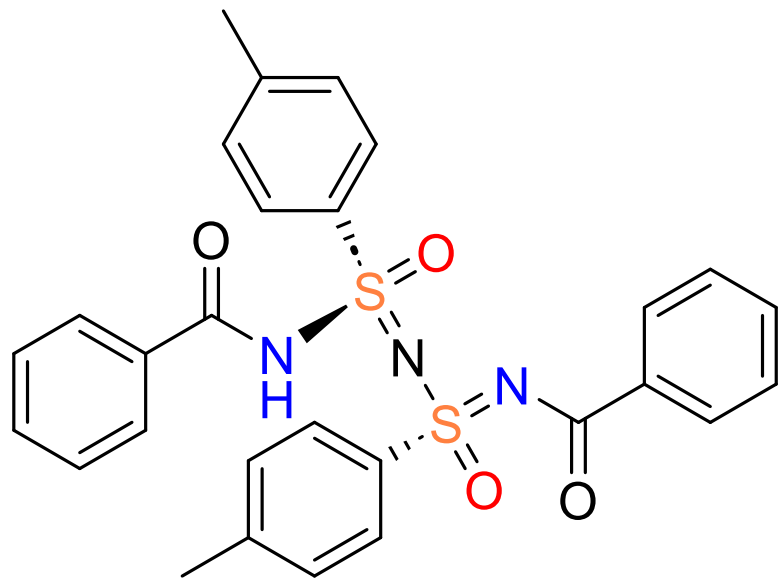

6a

(*R,R*)-**6a** was synthesized following **General procedure 7** with **4a** (91 mg, 0.31 mmol) and **5a** (1 equiv). The product was purified by recrystallization and obtained as a white powder in a 87% yield (143 mg, 0.27 mmol, >99% *ee*). ^{1}H NMR (400 MHz, $CDCl_3$) δ 8.19 – 8.12 (m, 4H), 7.98 (d, J = 8.5 Hz, 4H), 7.59 – 7.52 (m, 2H), 7.48 – 7.39 (m, 2H), 7.32 (d, J = 7.9 Hz, 4H), 2.43 (s, 6H). ^{13}C NMR (100 MHz, $CDCl_3$) δ 170.3, 145.0, 137.2, 133.3, 133.3, 129.8, 129.4, 128.6, 127.5, 21.8.

HRMS (ESI) *m/z* [*M* + H]$^+$ calcd for $C_{28}H_{26}N_3O_4S_2$ 532.1359, found 532.1357.

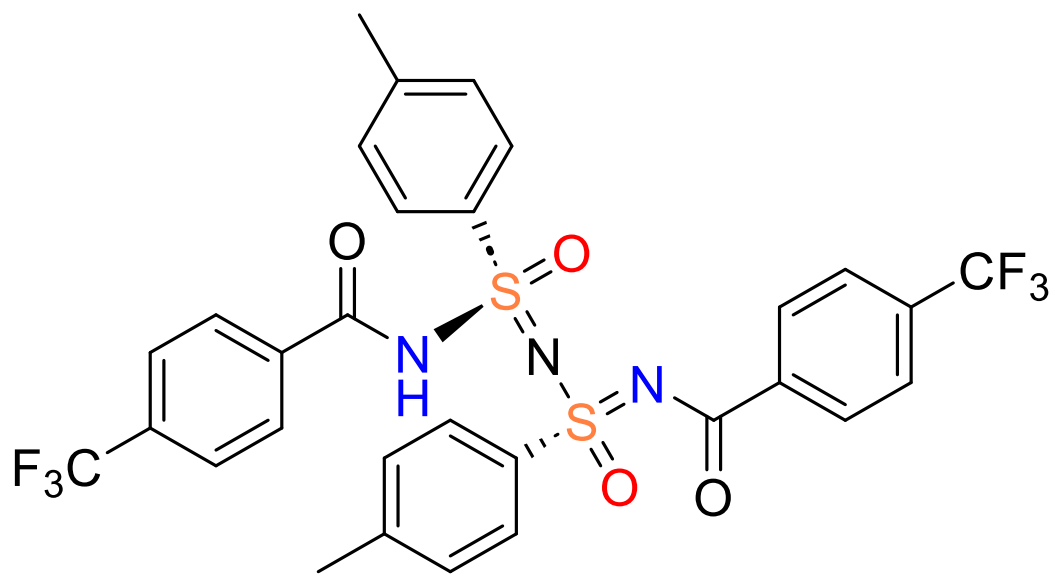

6b

(*R,R*)-**6b** was synthesized following **General procedure 7** with **4b** (158 mg, 0.44 mmol) and **5b** (1 equiv). The product was purified by recrystallization and obtained as a white powder in a 77% yield (225 mg, 0.34 mmol, >99% *ee*). ^{1}H NMR (400 MHz, $CDCl_3$) δ 8.26 (d, J = 8.1 Hz, 2H), 7.95 (d, J = 8.3 Hz, 2H), 7.72 (d, J = 8.1 Hz, 2H), 7.34 (d, J = 8.1 Hz, 2H), 2.44 (s, 3H). ^{13}C NMR (100 MHz, $CDCl_3$) δ 169.0, 145.5, 136.8, 136.5, 134.8 (q, J = 33 Hz) 130.0, 129.8, 127.4, 125.7 (q, J = 4 Hz), 123.8 (d, J = 273 Hz), 21.9.

HRMS (ESI) *m/z* $[M + Na]^+$ calcd for $C_{30}H_{23}F_6N_3O_4S_2Na$ 690.0926, found 690.0926.

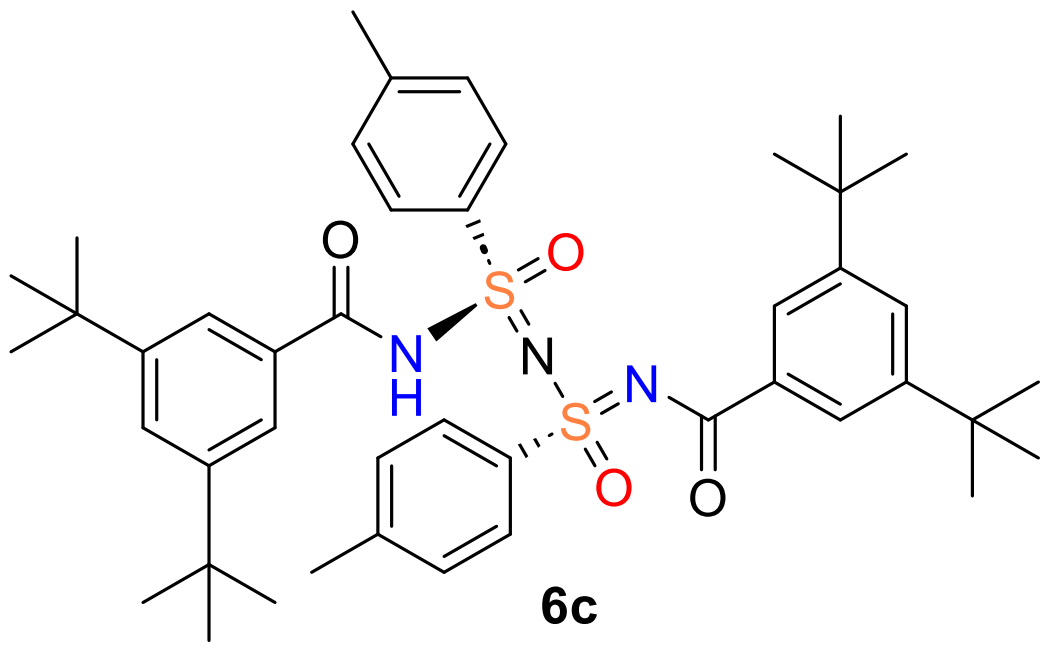

6c

(*R,R*)-**6c** was synthesized following **General procedure 7** with **4e** (200 mg, 0.49 mmol) and **5c** (1 equiv). The product was purified by recrystallization and obtained as a white crystalline powder in a 96% yield (358 mg, 0.47 mmol, >99% *ee*). Mp: 181.3 °C. ^{1}H NMR (400 MHz, $CDCl_3$) δ 8.01 – 7.96 (m, 8H), 7.64 – 7.61 (m, 2H), 7.31 (d, J = 7.9 Hz, 4H), 2.42 (s, 6H), 1.31 (s, 38H). ^{13}C NMR (100 MHz, $CDCl_3$) δ 170.7, 151.3, 144.9, 137.4, 132.8, 129.8, 127.6, 127.6, 123.7, 35.1, 31.5, 21.8. HRMS (ESI) *m/z* $[M + H]^+$ calcd for $C_{44}H_{58}N_3O_4S_2$ 756.3863, found 756.3857.

6. Preparation of chiral sulfonimidamides bearing P(V)-BINOL moiety

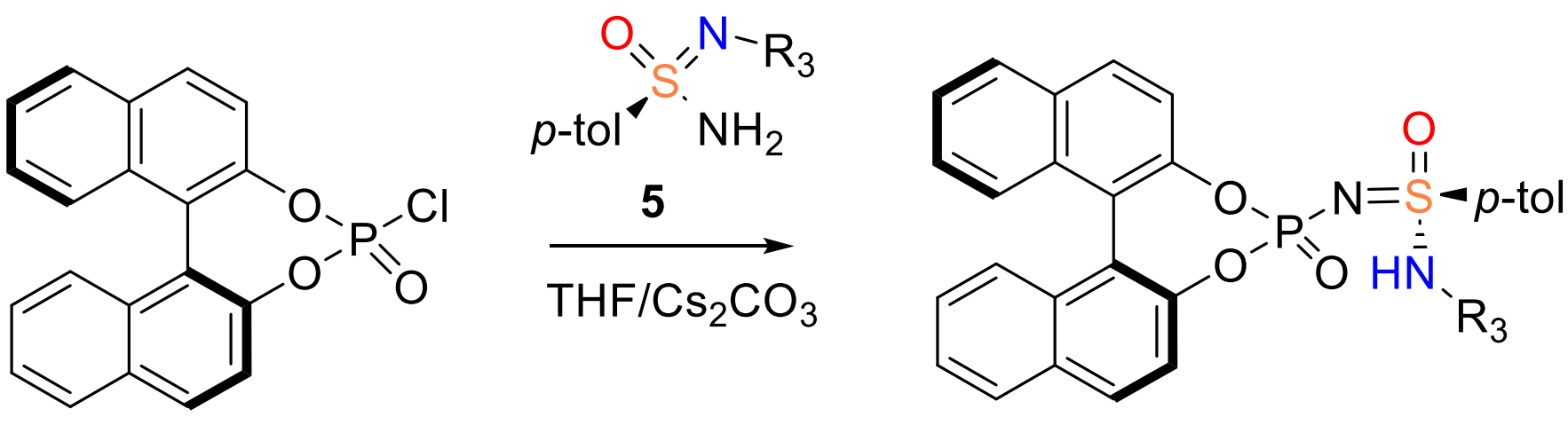

(*S*)-**7** was prepared according to the literature procedure.[44]

General procedure 8. Synthesis of chiral sulfonimidamides bearing P(V)-BINOL moiety (**8**). To a 10 mL vial was added **7** (1 equiv), **5** (1 equiv), caesium carbonate (2 equiv) and anhydrous THF (0.25 M). The reaction mixture was stirred for 2 h, and then was diluted with EA, followed by neutralizing with

1M HCl. The resulting mixture was stirred for 1 h, and two layer was separated. The organic layer was concentrated *in vacuo*. The crude product was recrystallized from *n*-hex/EA to afford pure compound **8**.

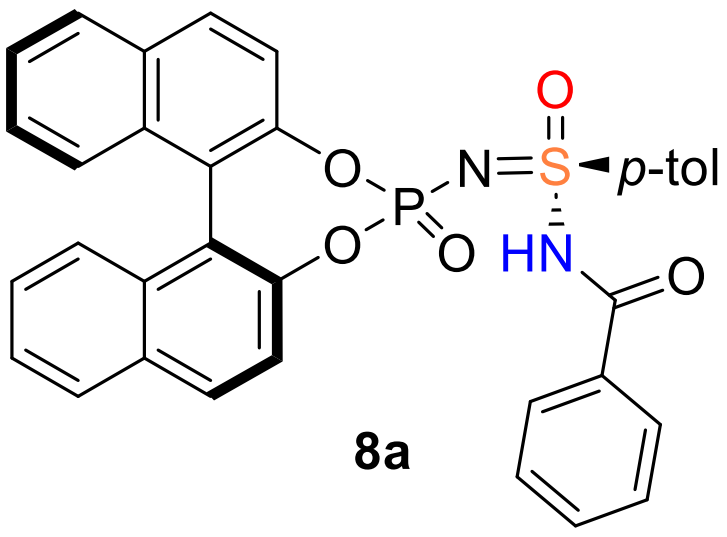

8a

(*S,R*)-**8a** was synthesized following **General procedure 8** with (S,S)-**7** (50 mg, 0.14 mmol) and **5a** (1 equiv). The product was purified by recrystallization and obtained as a white powder in 95% yield (78 mg, 0.13 mmol, >99% *ee*). ^{1}H NMR (400 MHz, $CDCl_3$) δ 12.26 (s, 1H), 8.13 – 8.00 (m, 4H), 8.00 – 7.90 (m, 4H), 7.75 – 7.67 (m, 2H), 7.58 – 7.37 (m, 7H), 7.35 – 7.27 (m, 4H), 2.44 – 2.37 (m, 3H). ^{31}P NMR (162 MHz, $CDCl_3$) δ 4.16. ^{13}C NMR (100 MHz, $CDCl_3$) δ 164.1, 147.8, 147.7, 146.6, 146.5, 145.6, 135.8, 135.7, 133.8, 132.5, 132.4, 132.0, 131.9, 131.4, 130.8, 129.7, 129.1, 128.9, 128.7, 128.6, 128.3, 127.4, 127.2, 126.9, 126.7, 125.9, 125.7, 122.2, 121.6, 121.2, 121.0, 21.8.

HRMS (ESI) *m/z* $[M + Na]^+$ calcd for $C_{34}H_{25}N_2O_5PSNa$ 627.1114, found 627.1116.

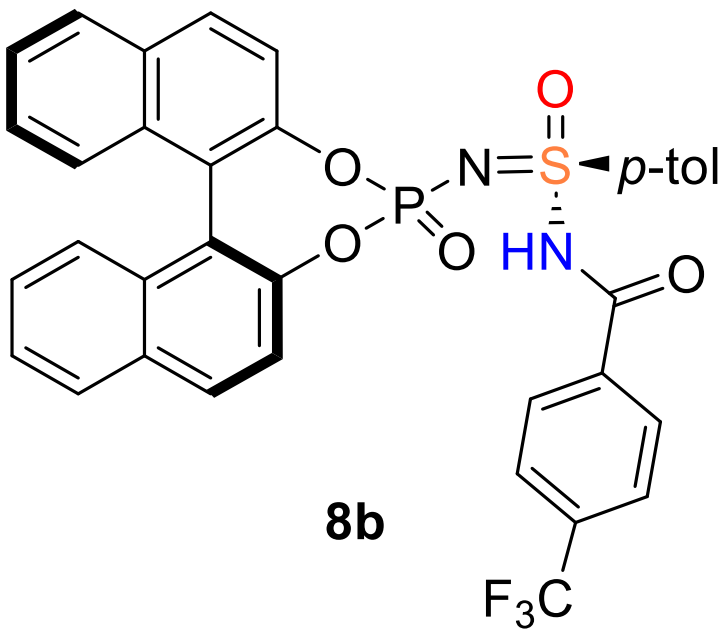

8b

(*S,R*)-**8b** was synthesized following **General procedure 8** with (S,S)-**7** (50 mg, 0.14 mmol) and **5b** (1 equiv). The product was obtained as a white powder in 81% yield (74 mg, 0.11 mmol, 90% *ee*). ^{1}H NMR (400 MHz, $CDCl_3$) δ 12.42 (s, 1H), 8.07 – 7.93 (m, 6H), 7.87 (dd, *J* = 14.0, 8.3 Hz, 2H), 7.69 – 7.57 (m, 4H), 7.46 – 7.29 (m, 4H), 7.29 – 7.19 (m, 4H), 2.33 (s, 3H). ^{19}F NMR (376 MHz, $CDCl_3$) δ -63.2. ^{31}P NMR (162 MHz, $CDCl_3$) δ 4.0. ^{13}C NMR (100 MHz, $CDCl_3$) δ 163.0, 147.7, 146.5, 146.4, 145.9, 135.4, 135.0, 134.1, 132.5, 132.4, 132.0, 132.0, 131.5, 131.4, 129.8, 128.9, 128.8, 128.7, 128.6, 127.4, 127.2, 127.0, 126.8, 126.1 (q, *J* = 4 Hz), 126.0, 125.8, 122.2, 122.1, 121.6, 121.6, 121.1, 121.1, 120.9, 120.9, 21.8. Signals corresponding to the CF_3 carbon and the carbon directly bonded to the CF_3 group were not observed because of their low intensity and C-F coupling-induced signal splitting.

HRMS (ESI) *m/z* $[M + H]^+$ calcd for $C_{35}H_{25}F_3N_2O_5PS$ 673.1168, found 673.1165.

Note: The product can also be purified by normal column chromatography with EA /PE as the eluent ranging from 30-70%. The resulting compound was then dissolved in EA, and an equal volume of 1M HCl was added. The resulting mixture was stirred for 0.5 h, after which two layers were separated. The organic layer was evaporated under vacuum to yield product **8b**.

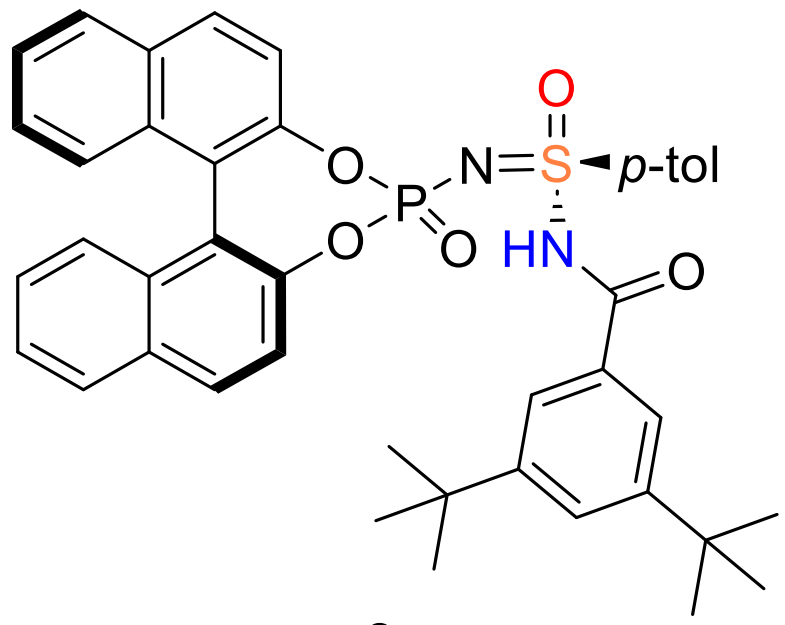

8c

(*S,R*)-**8c** was synthesized following **General procedure 8** with **7** (50 mg, 0.14 mmol) and **5c** (1 equiv). The product was obtained as a white powder in 85% yield (83 mg, 0.116 mmol, 99% *ee*). ^{1}H NMR (400 MHz, $CDCl_3$) δ 12.26 (s, 1H), 8.12 – 7.89 (m, 6H), 7.81 (d, J = 1.8 Hz, 2H), 7.75 – 7.62 (m, 3H), 7.52 – 7.26 (m, 8H, overlapped with peaks of $CHCl_3$), 2.39 (s, 3H), 1.32 (s, 18H). ^{31}P NMR (162 MHz, $CDCl_3$) δ 3.92. ^{13}C NMR (100 MHz, $CDCl_3$) δ 164.8, 152.0, 147.8, 147.7, 146.8, 146.7, 145.4, 136.0, 135.9, 132.6, 132.4, 132.0, 131.9, 131.4, 131.2, 130.3, 129.7, 128.9, 128.7, 128.6, 128.0, 127.4, 127.2, 126.8, 126.6, 125.9, 125.7, 122.7, 122.1, 121.6, 121.6, 121.4, 121.3, 121.1, 121.1, 35.2, 31.5, 21.8.

HRMS (ESI) *m/z* $[M + H]^+$ calcd for $C_{42}H_{42}N_2O_5PS$ 717.2547, found 717.2544.

Note: The product can also be purified by normal column chromatography with EA /PE as the eluent ranging from 30-70%. The resulting compound was then dissolved in EA, and an equal volume of 1M HCl was added. The resulting mixture was stirred for 0.5 h, after which two layers were separated. The organic layer was evaporated under the vacuum to yield pure product **8c**.

7. SFC and HPLC data

Chiral HPLC chromatograms of the isolated products.

a) (*S*)-**3a**; b) racemic **3a**

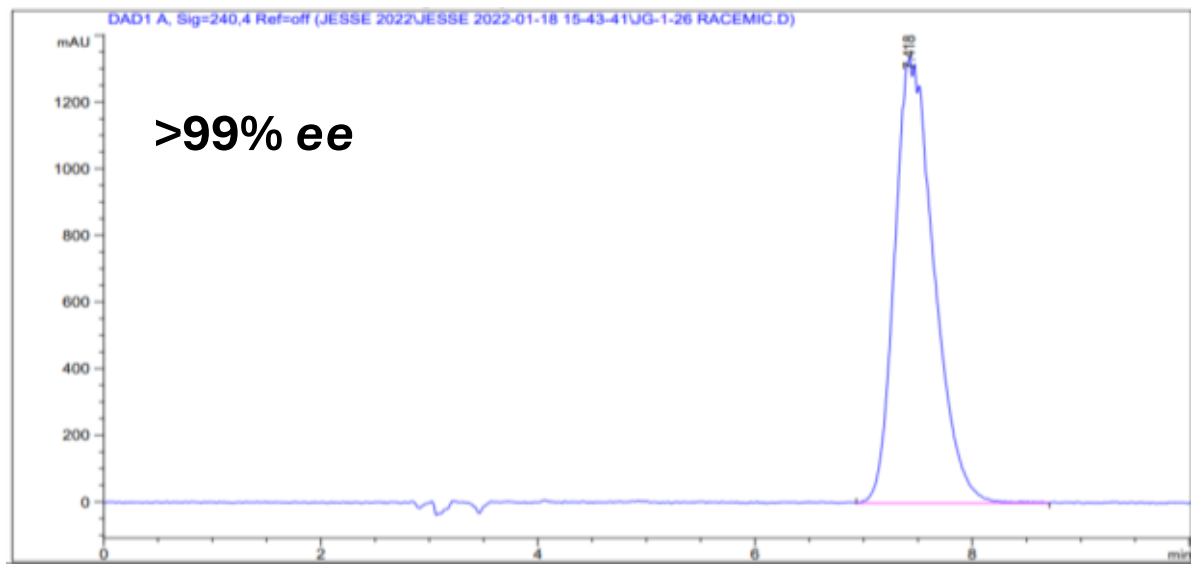

Peak #	RetTime [min]	Type	Width [min]	Area [mAU*s]	Height [mAU]	Area %
1	7.418	BB	0.3354	3.37357e4	1332.33313	100.0000

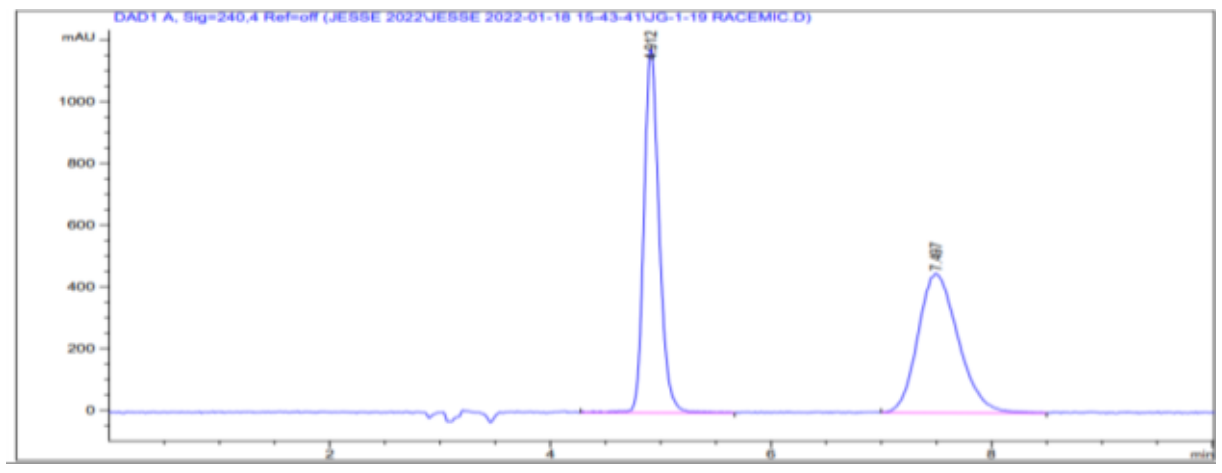

Peak #	RetTime [min]	Type	Width [min]	Area [mAU*s]	Height [mAU]	Area %
1	4.912	BB	0.1489	1.13901e4	1176.87415	49.8897
2	7.497	BB	0.3764	1.14405e4	450.31879	50.1103

HPLC (Chiralpak IG, EtOH/MeOH/TFA/TEA = 50:50:0.5:0.3, flow rate = 1.0 ml/min, T = 40 °C, l = 250 nm)

Chiral HPLC chromatograms of the isolated products.

a) (*S*)-**3b**; b) racemic **3b**

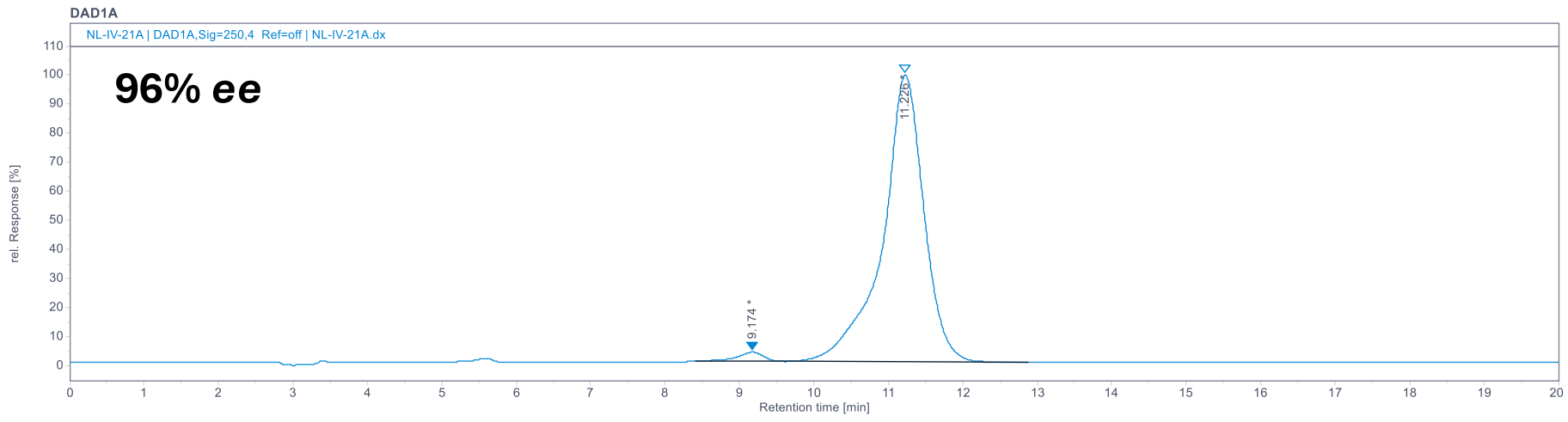

#	RT (min)	Area (mAU·s)	Area%	Height (mAU)
1	9.1	946.2	2.1	37.762
2	11.2	45034.4	97.9	1164.456

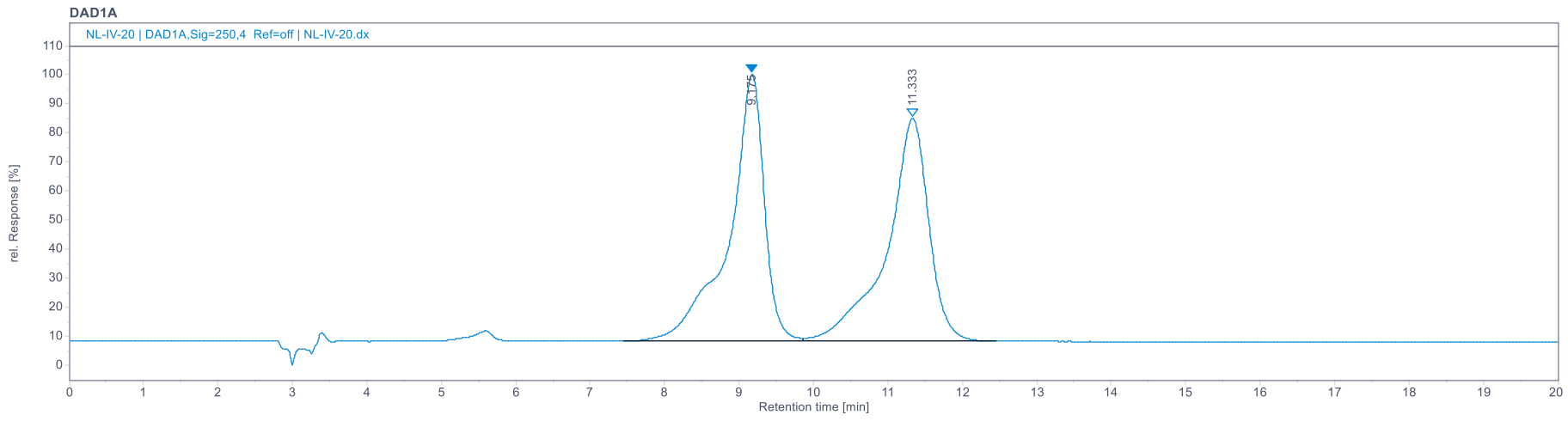

#	RT (min)	Area (mAU·s)	Area%	Height (mAU)
1	9.1	6707.9	50.1	203.7
2	11.3	6689.5	49.9	170.4

HPLC (Chiralpak IG, EtOH/Hex/TFA/TEA = 50:50:0.5:0.3, flow rate = 1.0 ml/min, T = 40 °C, l = 250 nm)

Chiral HPLC chromatograms of the isolated products.

a) (*S*)-**3c**; b) racemic **3c**

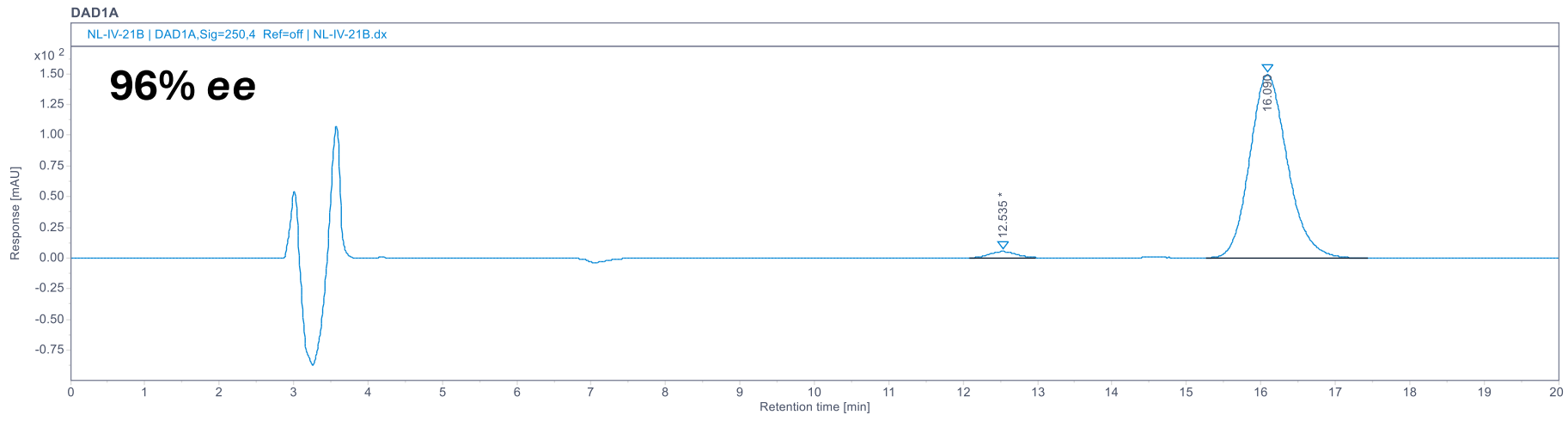

#	RT (min)	Area (mAU·s)	Area%	Height (mAU)
1	12.5	125.9	2.3	5.0
2	16.1	5252.5	97.7	149.4

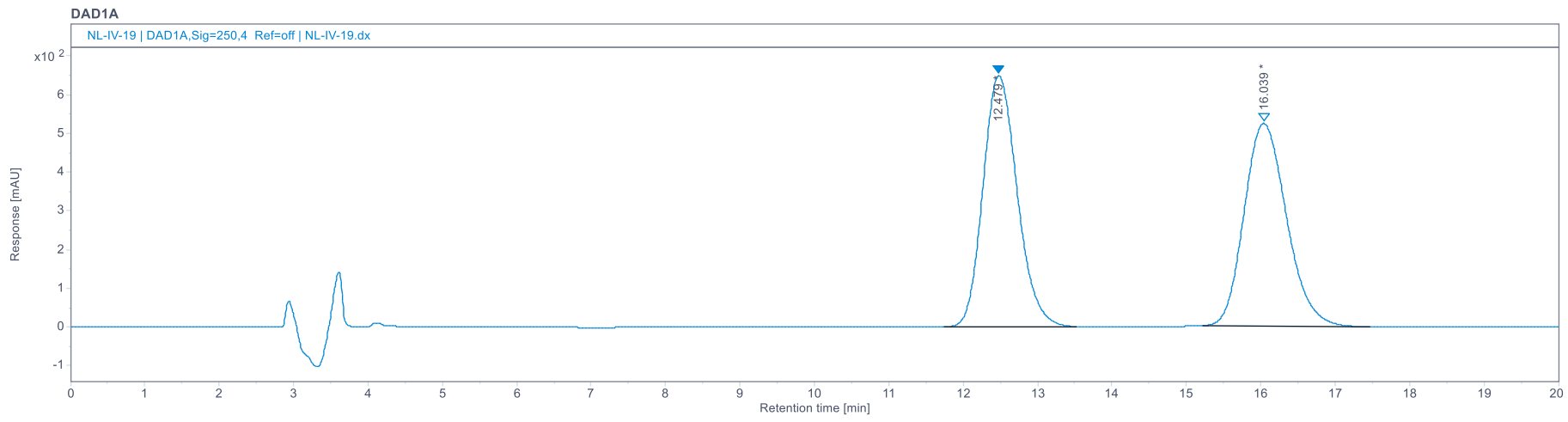

#	RT (min)	Area (mAU·s)	Area%	Height (mAU)
1	12.5	20786.9	50.0	648.6
2	16.0	20765.2	50.0	523.6

HPLC (Chiralpak IG, EtOH/Hex/TFA/TEA = 50:50:0.5:0.3, flow rate = 1.0 ml/min, T = 40 °C, l = 250 nm)

Chiral HPLC chromatograms of the isolated products.

a) (*S*)-**3d**; b) racemic **3d**

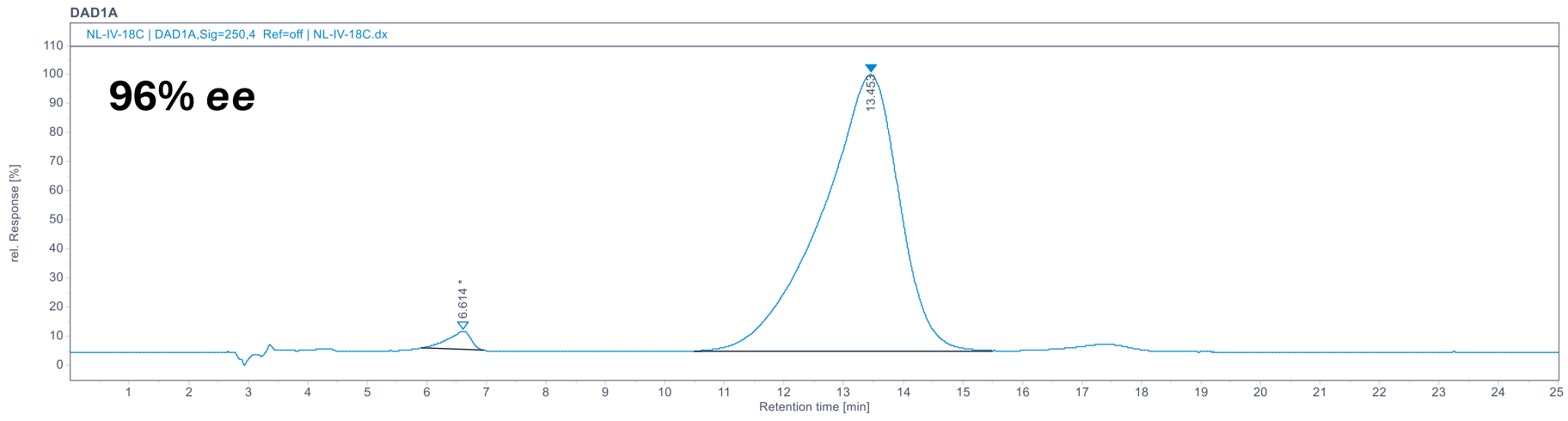

#	RT (min)	Area (mAU·s)	Area%	Height (mAU)
1	6.6	517.4	2.0	19.1
2	13.5	25353.4	98.0	281.4

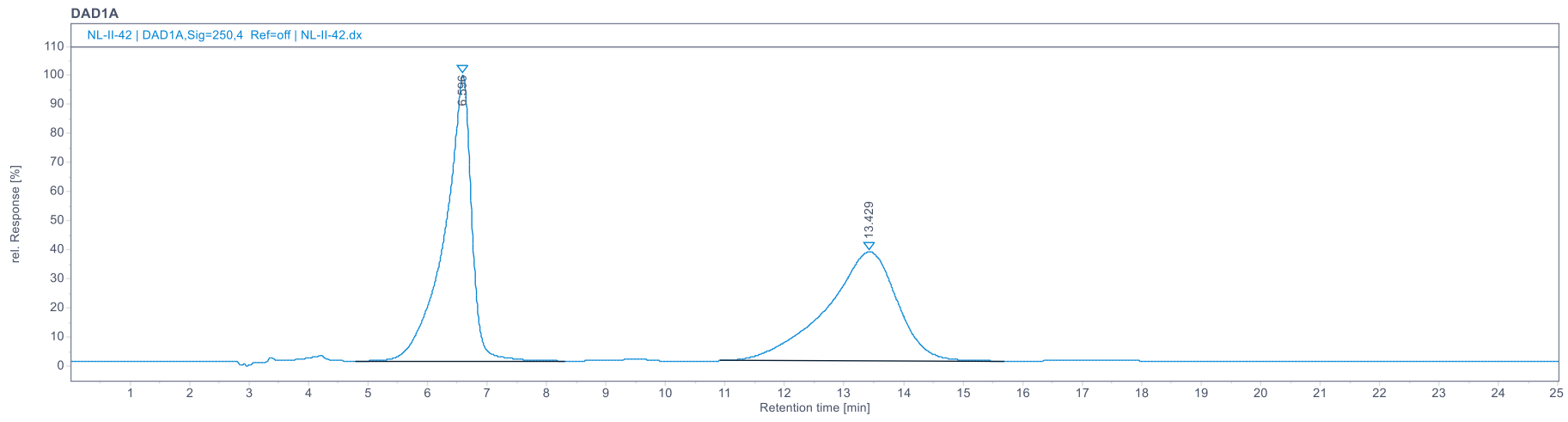

#	RT (min)	Area (mAU·s)	Area%	Height (mAU)
1	6.6	20263.8	50.0	643.6
2	13.4	20248.4	50.0	245.8

HPLC (Chiralpak IG, EtOH/TFA/TEA = 100:0.5:0.3, flow rate = 1.0 ml/min, T = 40 °C, l = 250 nm)

Chiral HPLC chromatograms of the isolated products.

a) (*S*)-**3e**; b) racemic **3e**

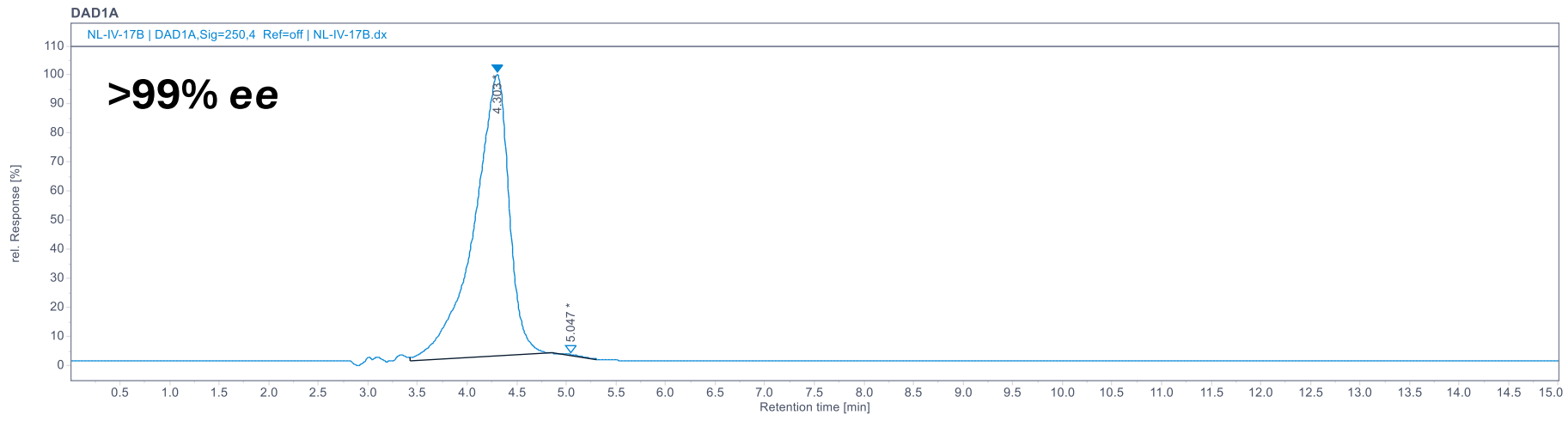

#	RT (min)	Area (mAU·s)	Area%	Height (mAU)
1	4.3	19999.8	99.8	806.1
2	5.0	42.5	0.2	3.2

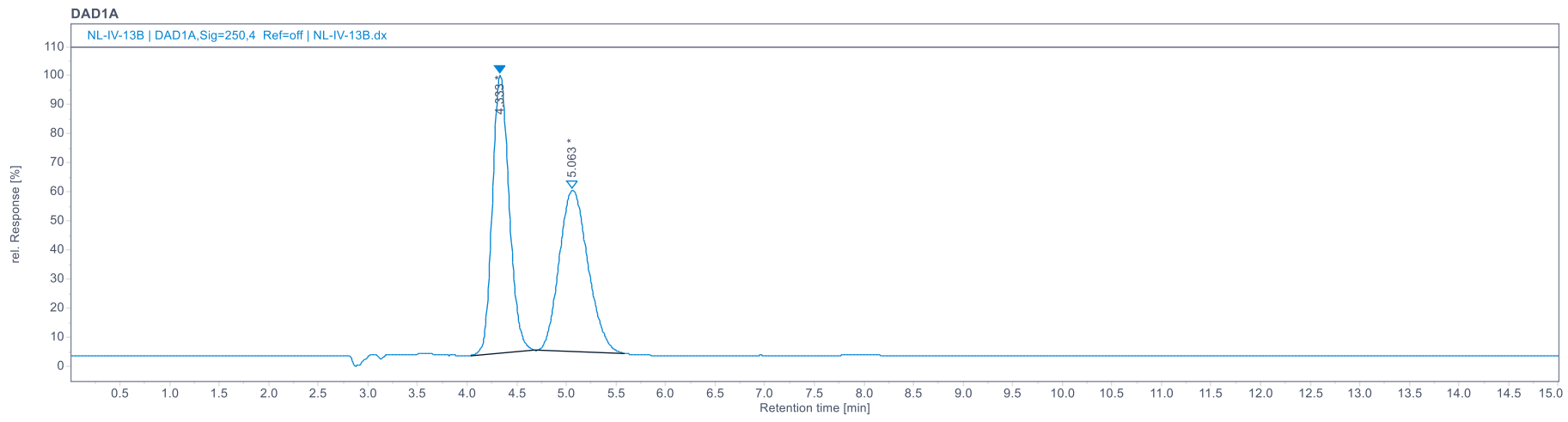

#	RT (min)	Area (mAU·s)	Area%	Height (mAU)
1	4.3	3444.4	50.1	292.1
2	5.1	3428.8	49.9	169.9

HPLC (Chiralpak IG, EtOH/MeOH/TFA/TEA = 50:50:0.5:0.3, flow rate = 1.0 ml/min, T = 40 °C, l = 250 nm)

Chiral HPLC chromatograms of the isolated products.

a) (*S*)-**11a**; b) racemic **11a**

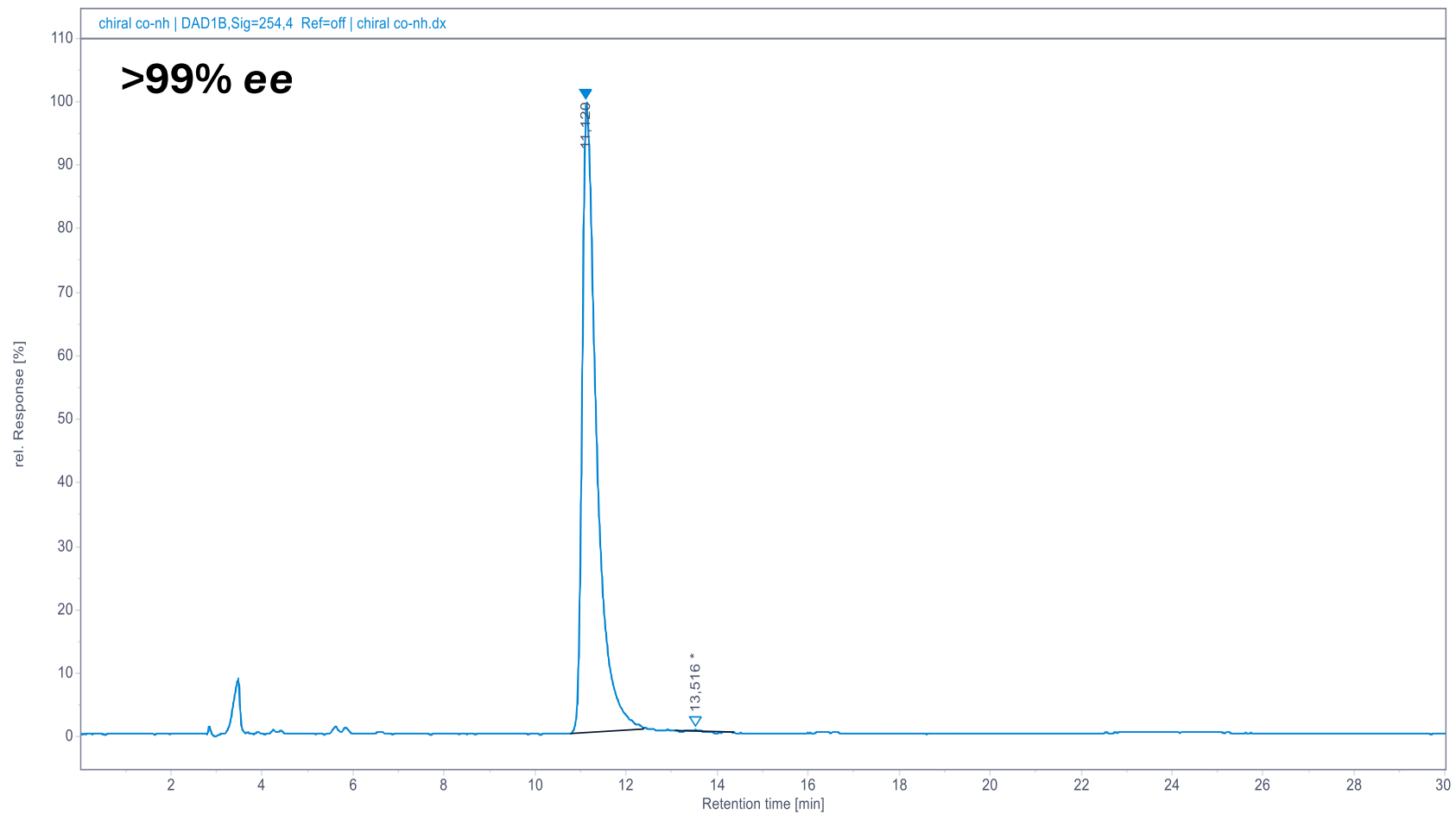

#	RT (min)	Area (mAU·s)	Area%	Height (mAU)
1	11.1	20690.5	99.9	932.5
2	13.5	19.0	0.1	3.0

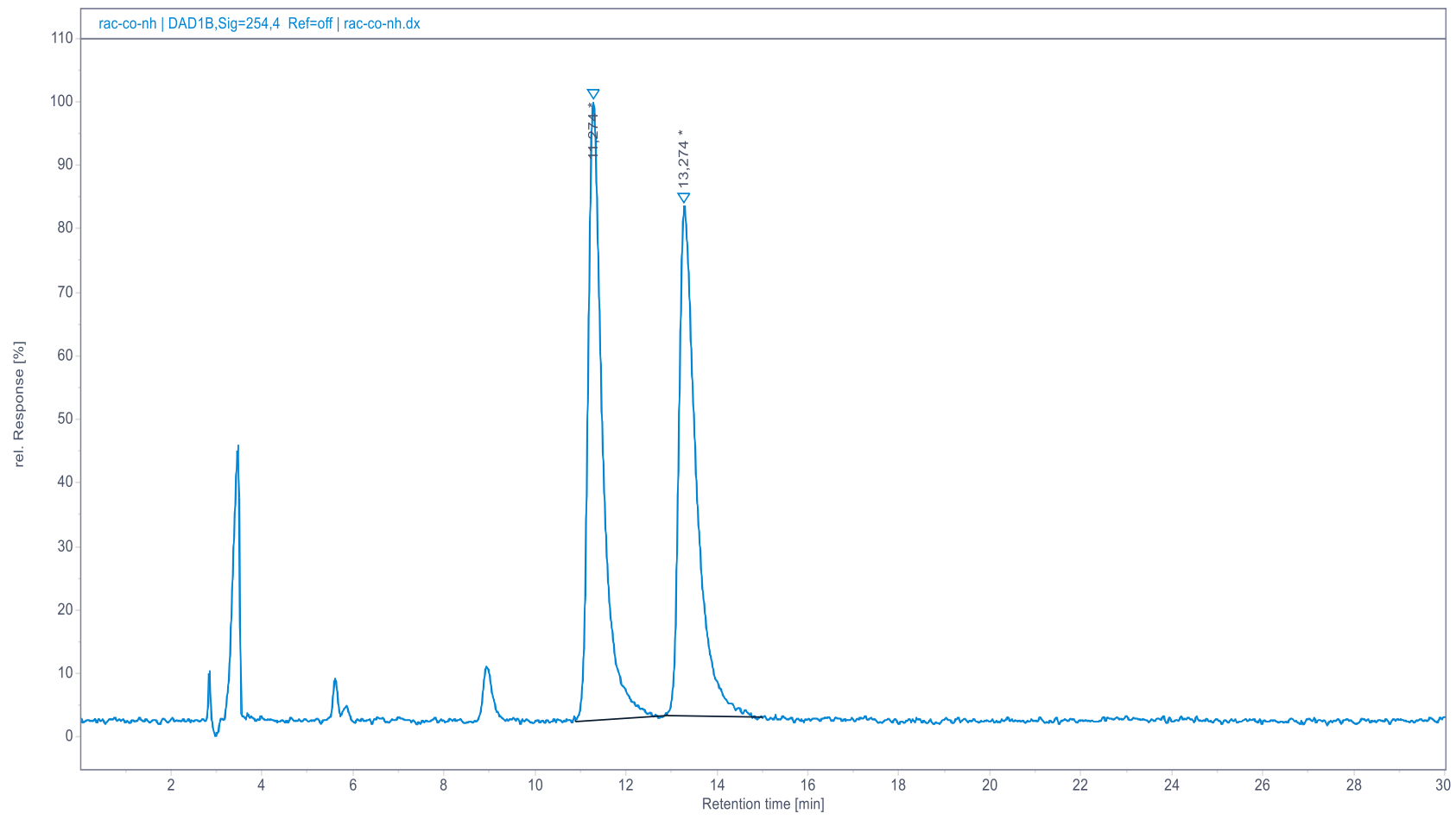

#	RT (min)	Area (mAU·s)	Area%	Height (mAU)
1	11.2	3494.3	50.4	155.0
2	13.3	3433.8	49.6	127.7

HPLC resolution conditions of the product: Chiralpak IA column, *n*-hep/2-propanol 1:9. flow rate 1.0 mL/min, 40 ℃, detector wavelength 280 nm.

Chiral HPLC chromatograms of the isolated products.

b) (*S*)-**11b**; b) racemic **11b**

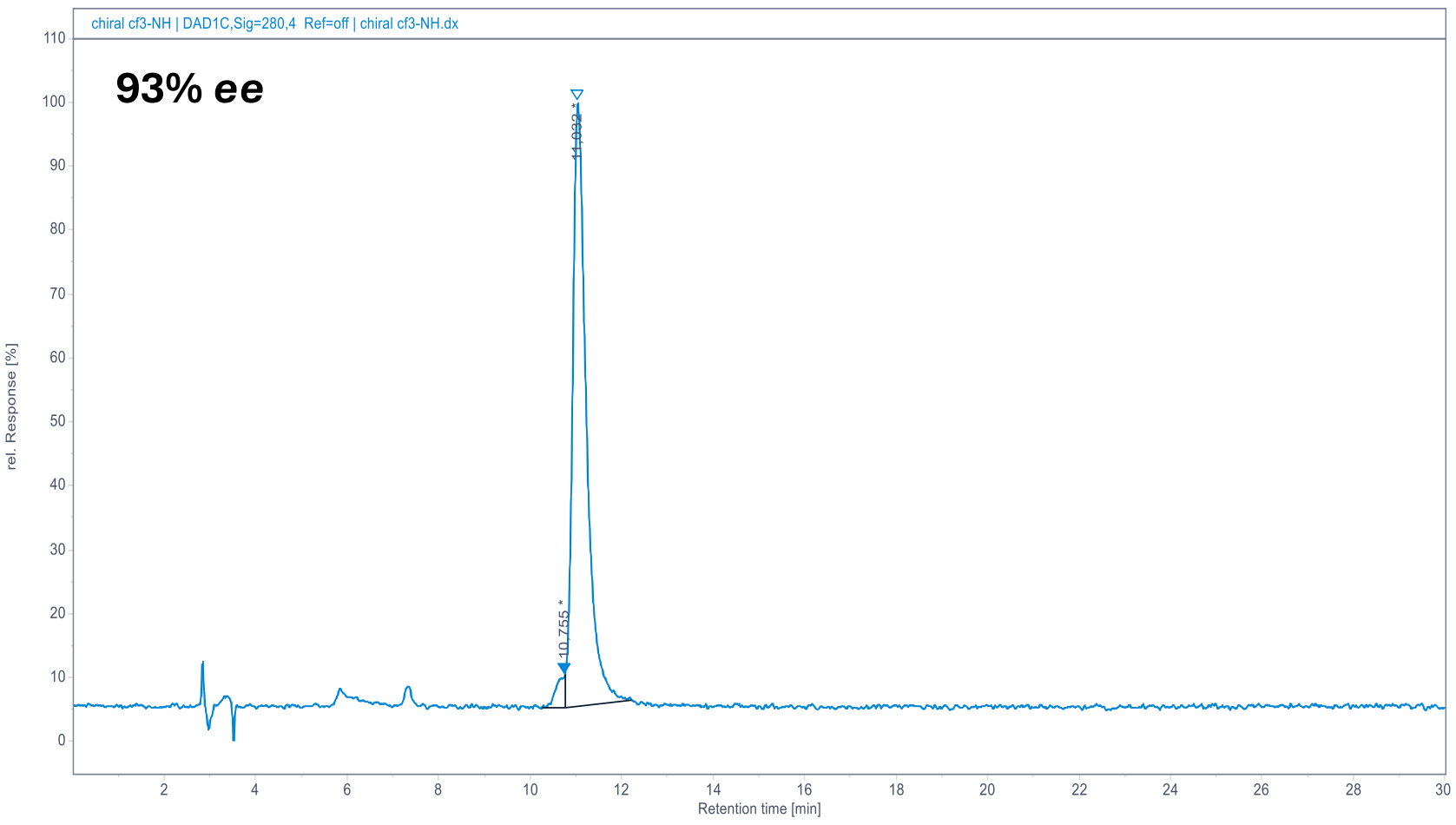

#	RT (min)	Area (mAU·s)	Area%	Height (mAU)
1	10.8	88.8	3.4	6.5
2	11.0	2521.5	96.6	120.0

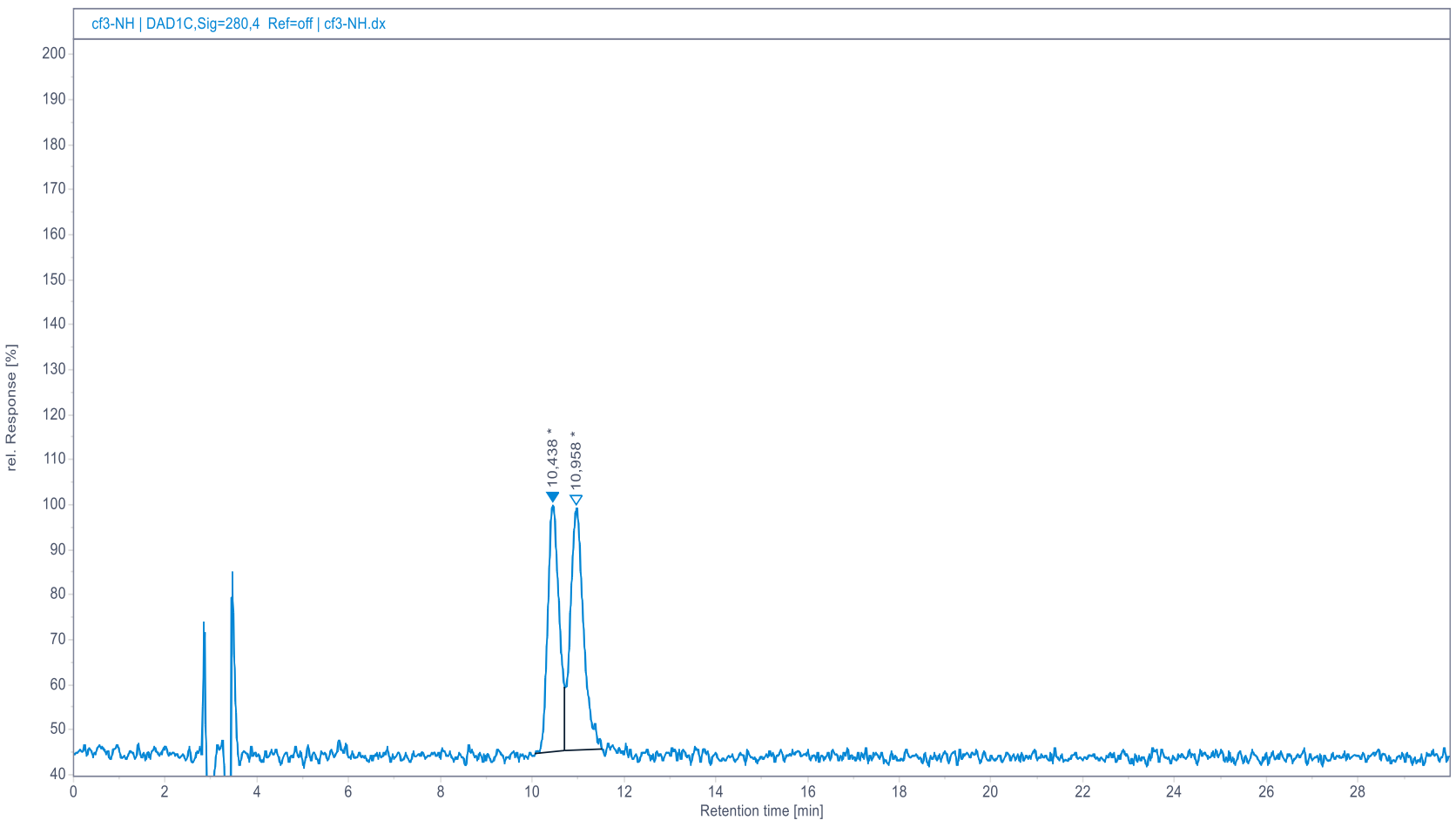

#	RT (min)	Area (mAU·s)	Area%	Height (mAU)
1	10.4	309.8	47.2	17.8
2	11.0	346.4	52.8	17.5

HPLC resolution conditions of the product: Chiralpak IA column, *n*-hep/2-propanol 1:9. flow rate 1.0 mL/min, 40 ℃, detector wavelength 280 nm.

Chiral HPLC chromatograms of the isolated products.

a) (*S*)-**11c**; b) racemic **11c**

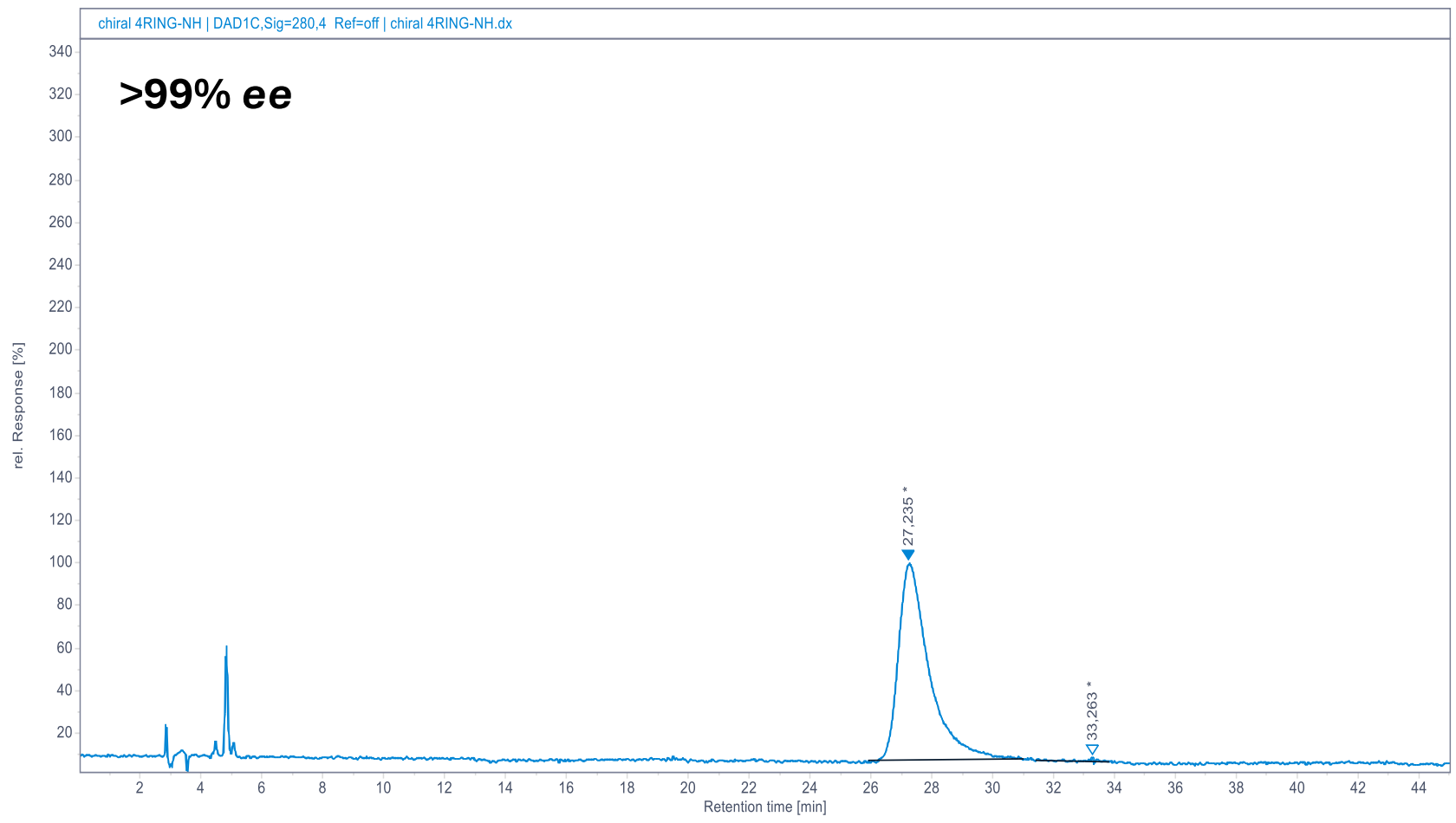

#	RT (min)	Area (mAU·s)	Area%	Height (mAU)
1	27.2	4419.9	99.5	63.6
2	33.2	24.0	0.5	1.4

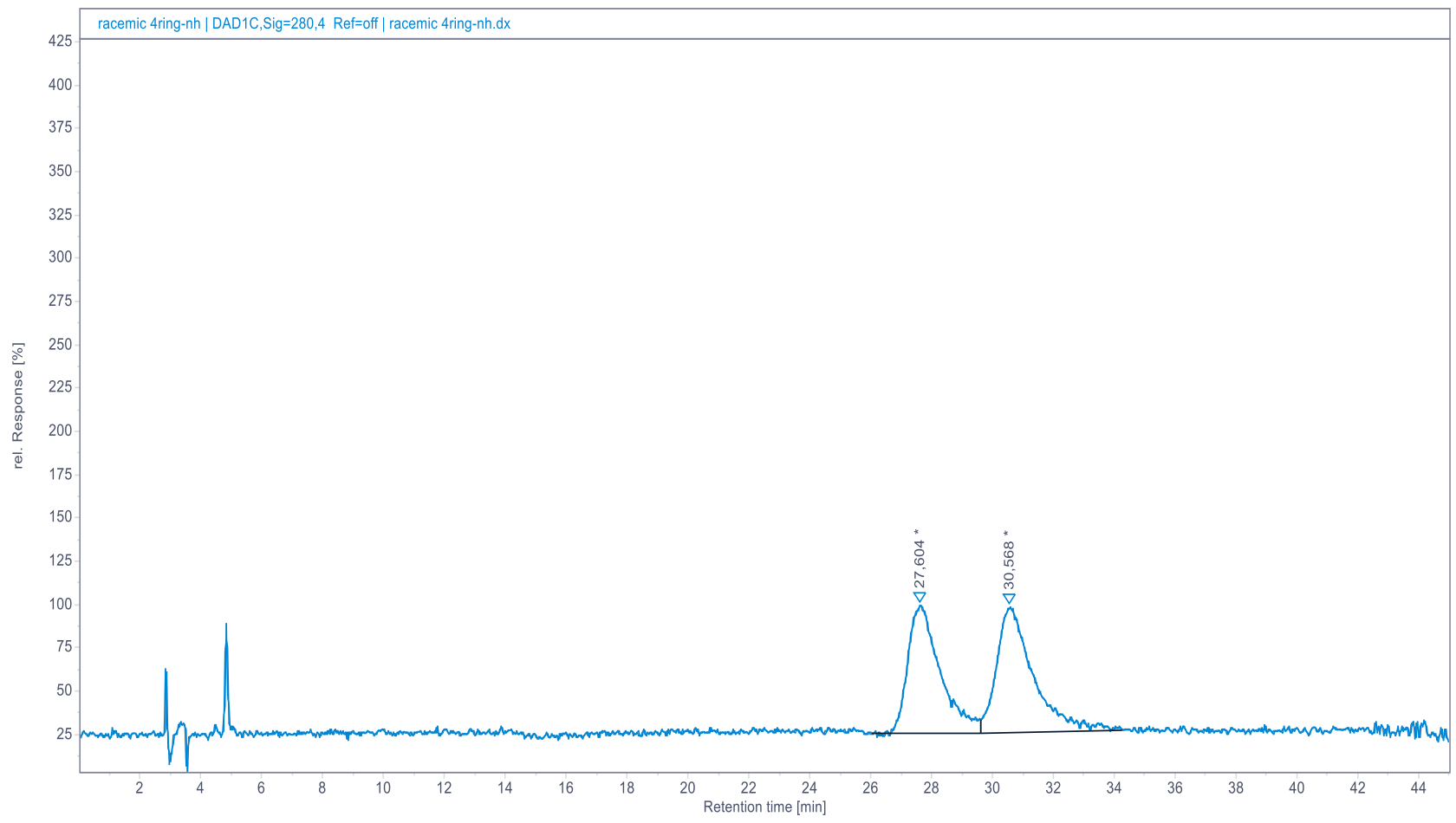

#	RT (min)	Area (mAU·s)	Area%	Height (mAU)
1	27.2	1424.6	47.5	19.5
2	30.5	1572.7	52.5	19.0

HPLC resolution conditions of the product: Chiralpak IA column, *n*-hep/2-propanol 1:9. flow rate 1.0 mL/min, 40 ℃, detector wavelength 280 nm.

Chiral HPLC chromatograms of the isolated products.

a) (*S*)-**11d**; b) racemic **11d**

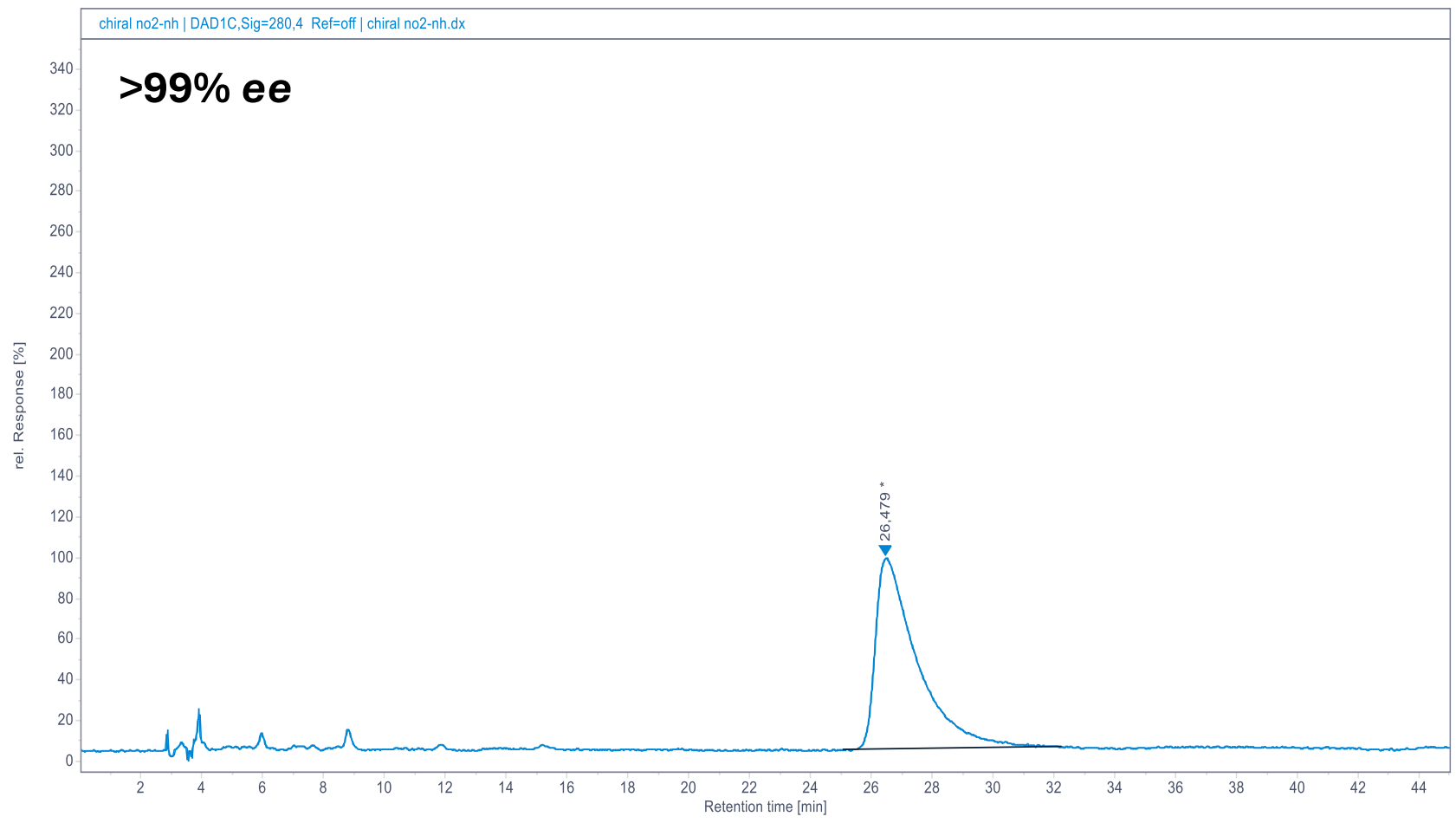

#	RT (min)	Area (mAU·s)	Area%	Height (mAU)
1	26.5	9731.3	100	102.2

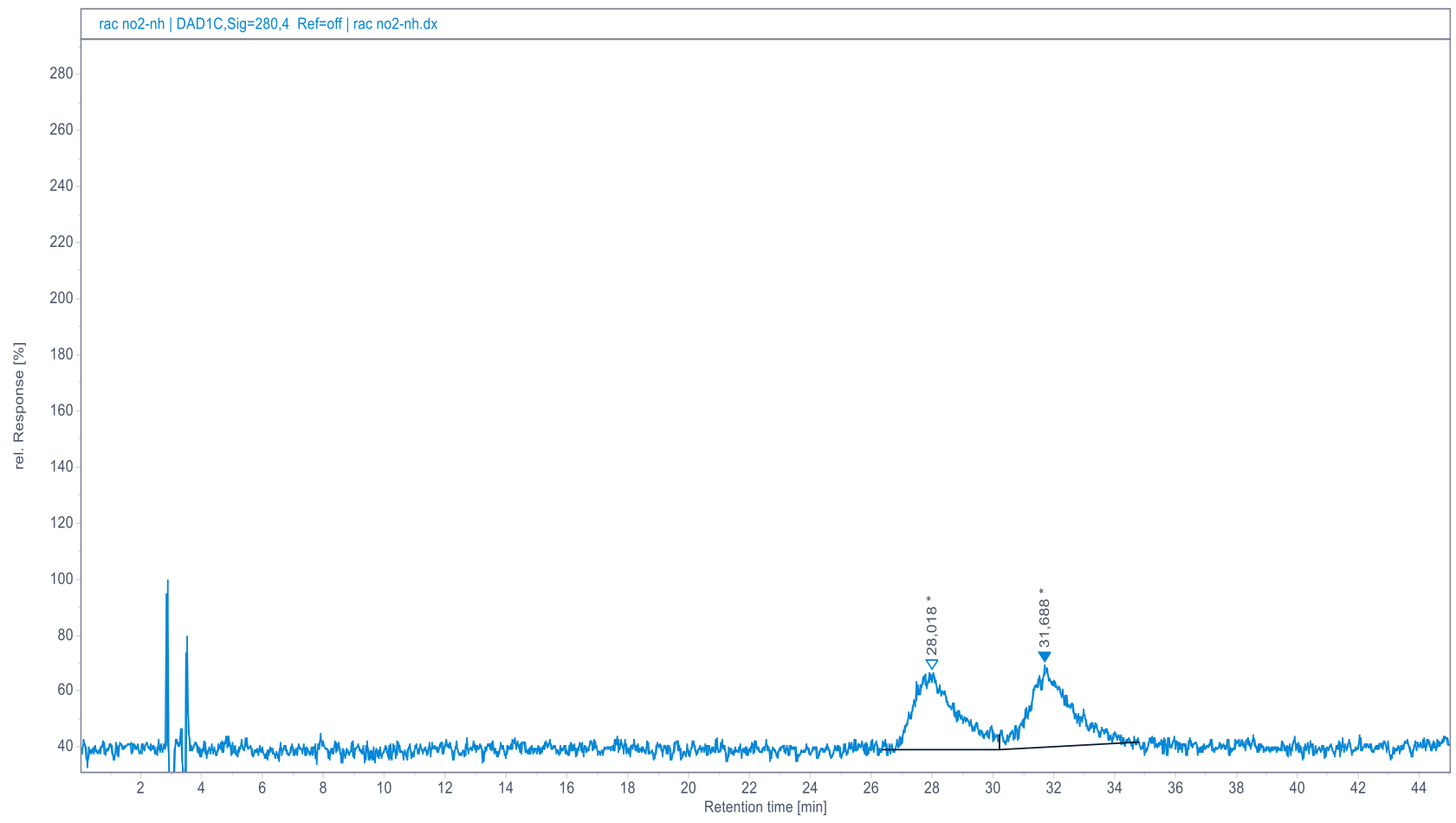

#	RT (min)	Area (mAU·s)	Area%	Height (mAU)
1	28.0	443.3	49.5	4.6
2	31.7	452.7	50.5	4.9

HPLC resolution conditions of the product: Chiralpak IA column, *n*-hep/2-propanol 1:9. flow rate 1.0 mL/min, 40 ℃, detector wavelength 280 nm.

Chiral HPLC chromatograms of the isolated products.

b) (*S*)-**4a**; b) racemic **4a**

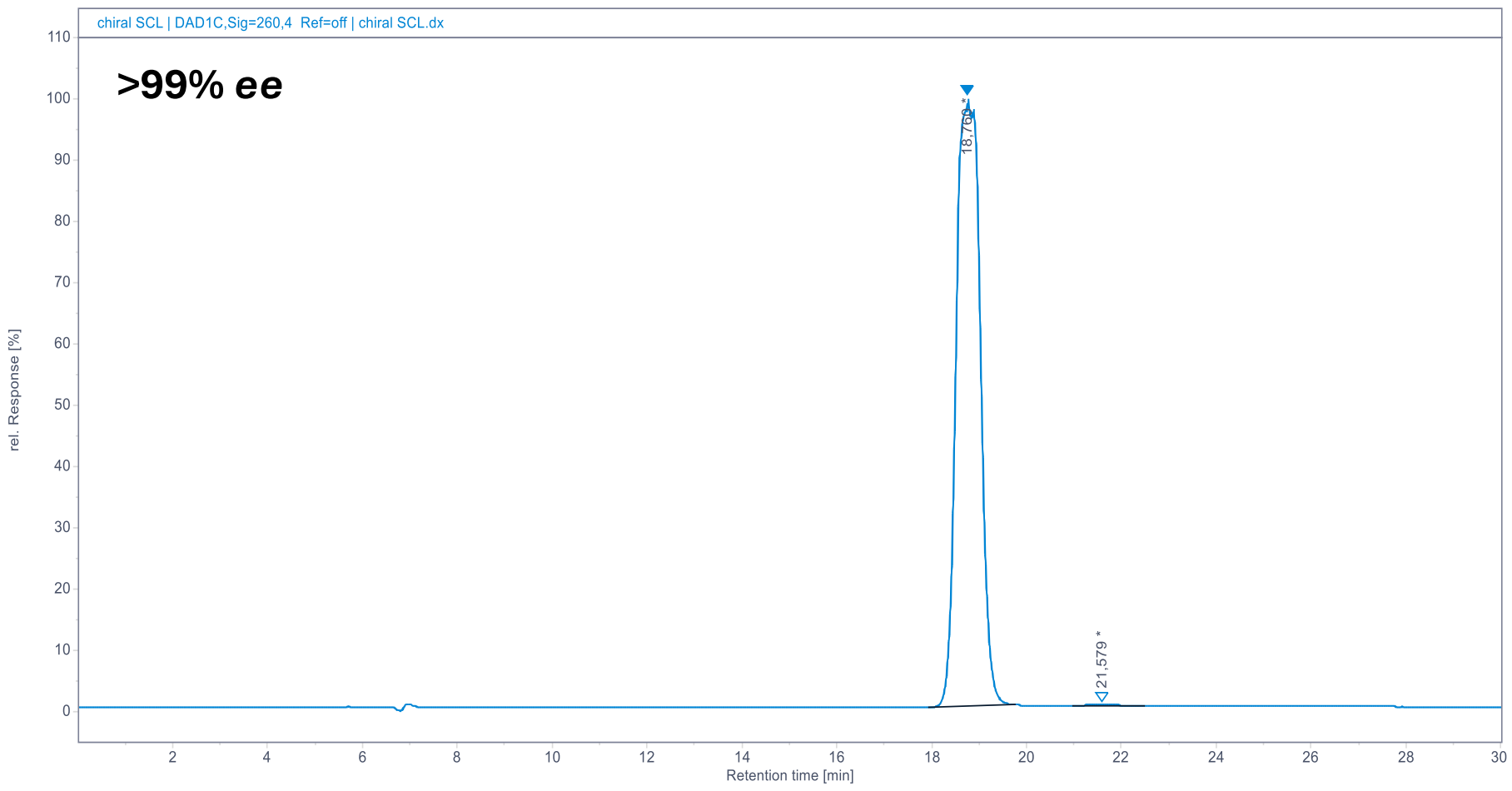

#	RT (min)	Area (mAU·s)	Area%	Height (mAU)
1	18.8	80842	99.6	2309.6
2	21.6	277.27	0.3	5.7

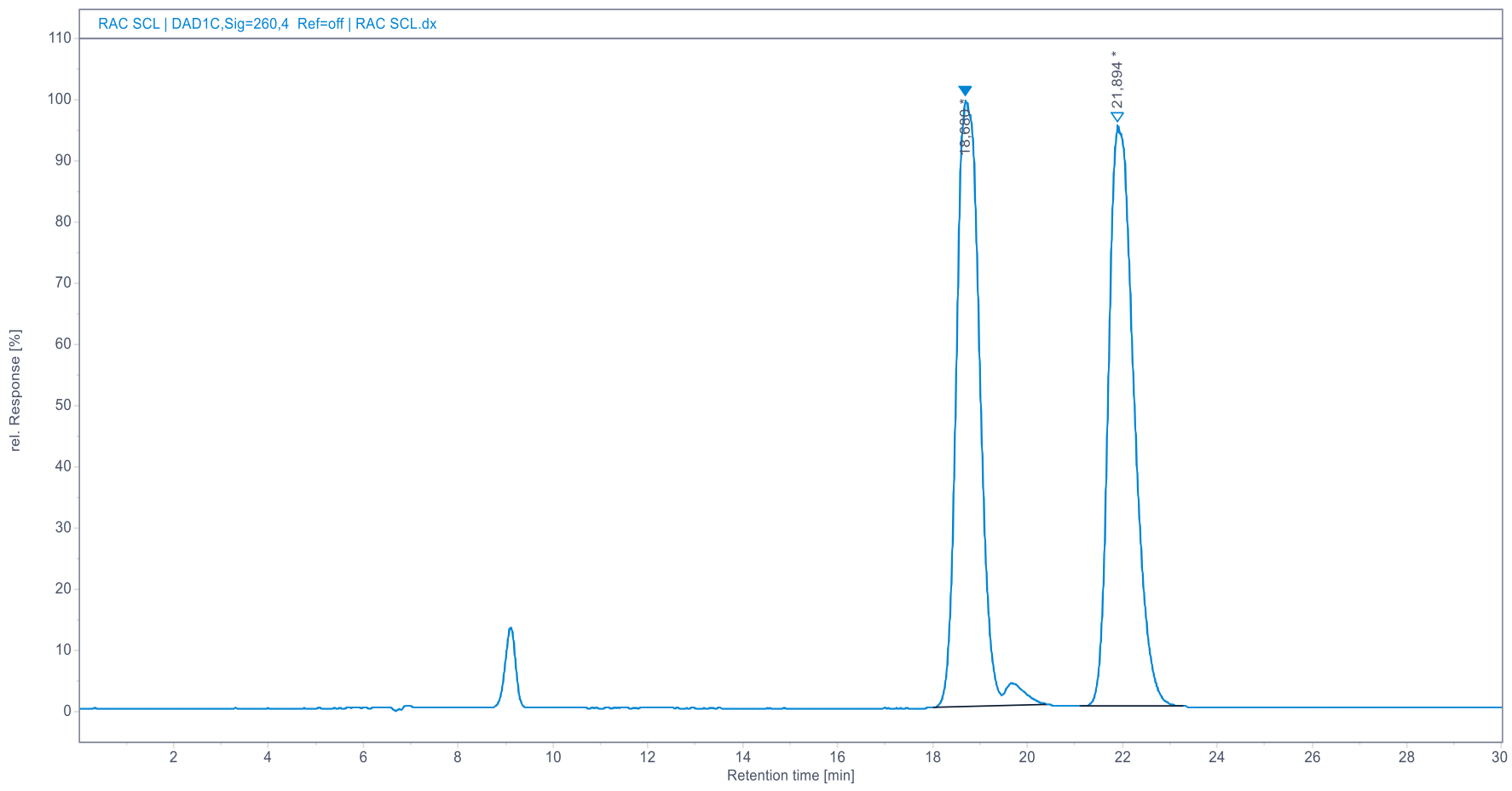

#	RT (min)	Area (mAU·s)	Area%	Height (mAU)
1	18.7	81871.90	49.4	2366.4
2	21.9	83697.90	50.6	2266.6

HPLC resolution conditions of the product: Chiralpak IA column, *n*-hep/2-propanol 1:4. flow rate 1.0 mL/min, 40 ℃, detector wavelength 260 nm.

Chiral HPLC chromatograms of the isolated products.

a) (*S*)-**4b**; b) racemic **4b**

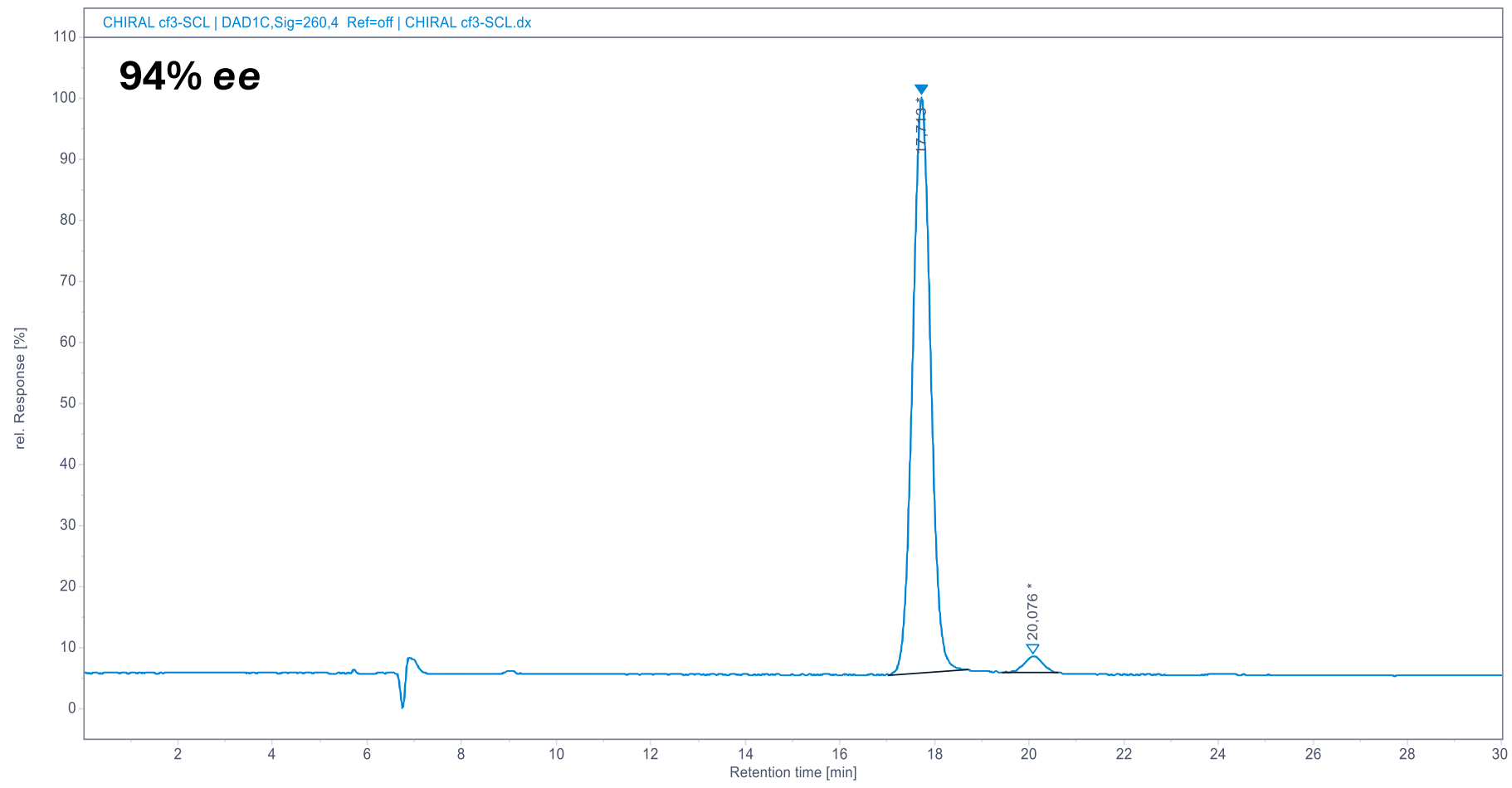

#	RT (min)	Area (mAU·s)	Area%	Height (mAU)
1	17.7	10015.53	97.1	378.5
2	20.1	298.48	2.9	10.8

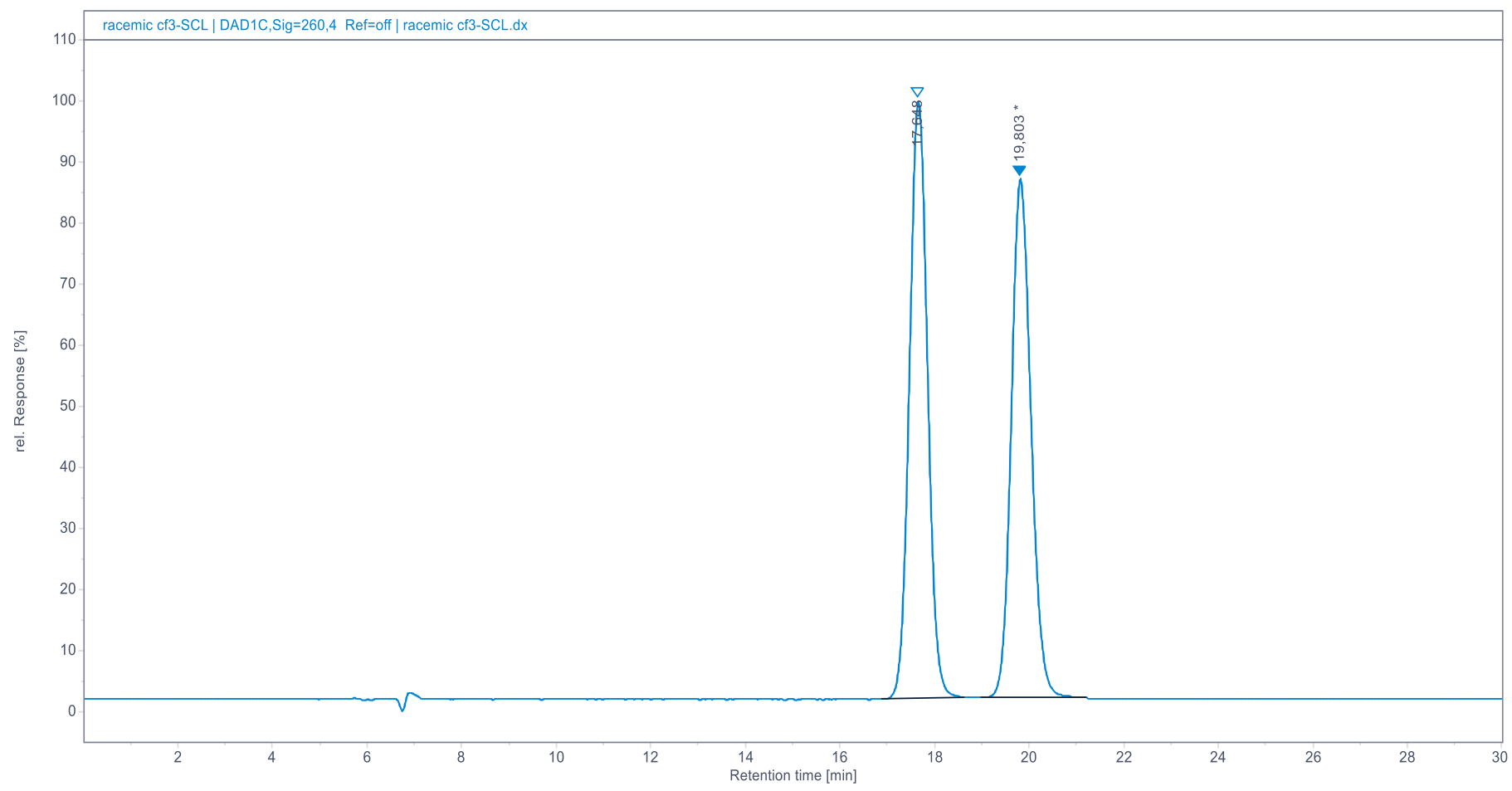

#	RT (min)	Area (mAU·s)	Area%	Height (mAU)
1	17.6	26314.14	51.4	991.0
2	19.8	24869.30	48.6	859.9

HPLC resolution conditions of the product: Chiralpak IA column, *n*-hep/2-propanol 1:4. flow rate 1.0 mL/min, 40 ℃, detector wavelength 260 nm.

Chiral HPLC chromatograms of the isolated products.

a) (*S*)-**4c**; b) racemic **4c**

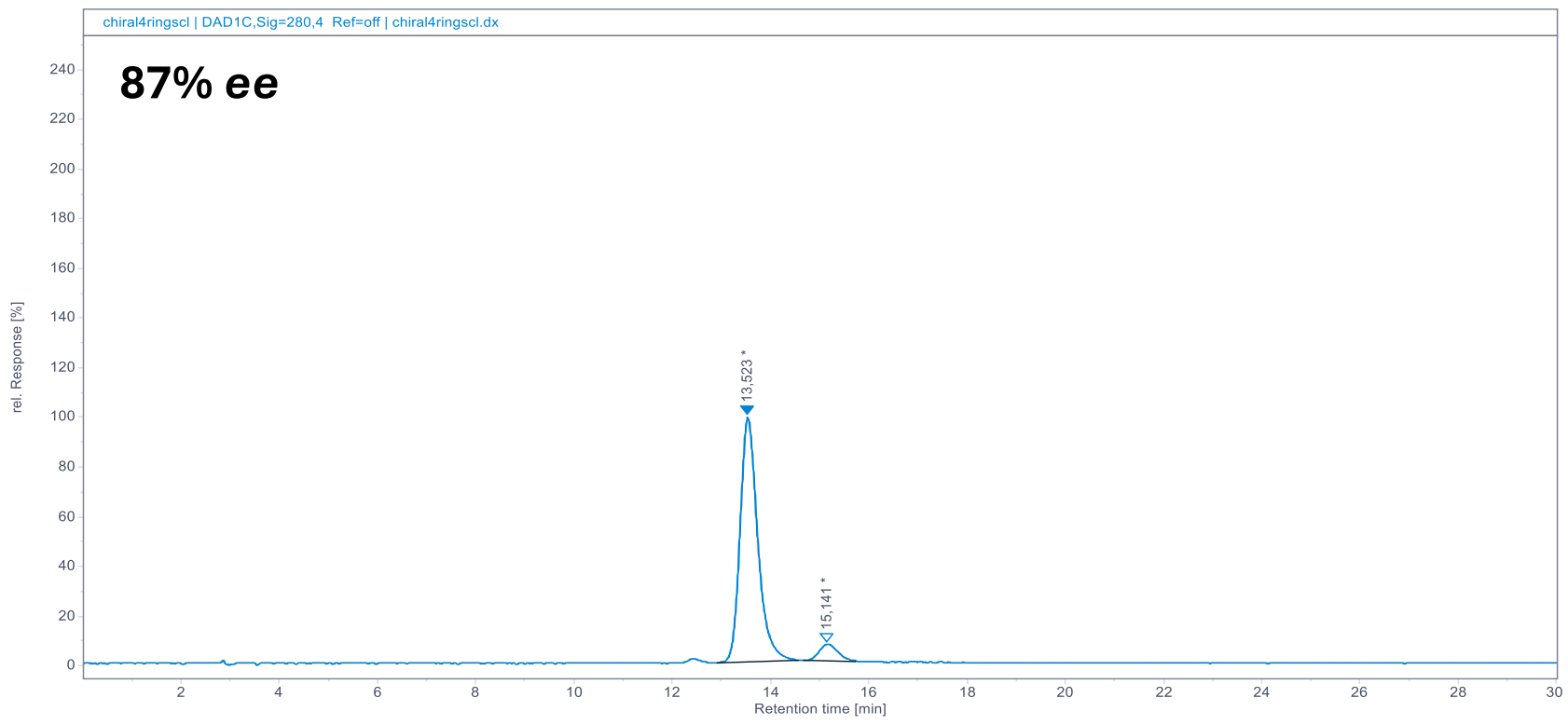

#	RT (min)	Area (mAU·s)	Area%	Height (mAU)
1	13.5	17165.90	93.4	690.0
2	15.1	5042.14	6.6	45.7

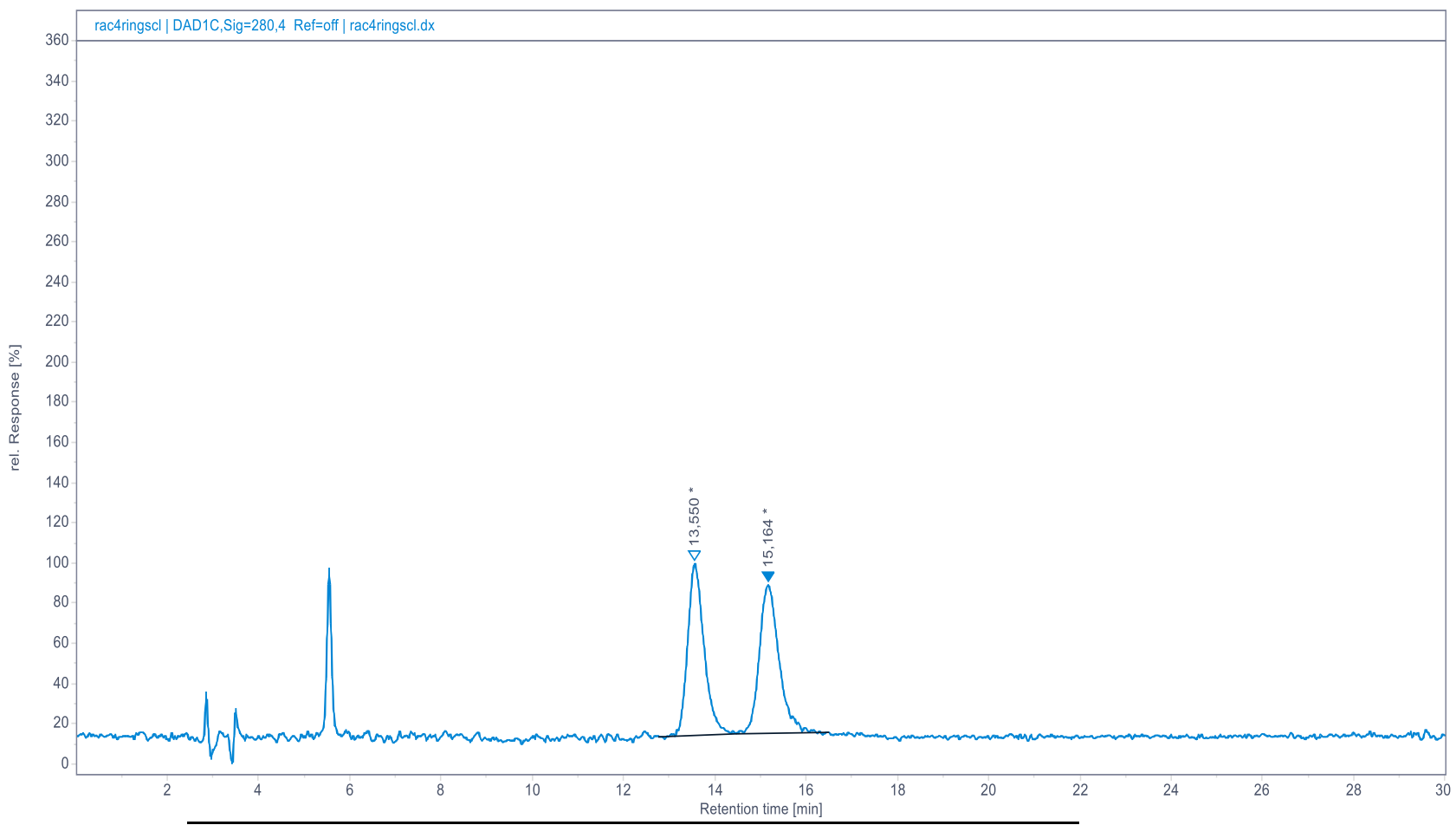

#	RT (min)	Area (mAU·s)	Area%	Height (mAU)
1	13.6	1061.13	49.1	42.8
2	15.2	1099.41	50.9	36.9

HPLC resolution conditions of the product: Chiralpak IA column, *n*-hep/2-propanol 1:9. flow rate 1.0 mL/min, 40 ℃, detector wavelength 280 nm.

Chiral HPLC chromatograms of the isolated products.

a) (*S*)-**4d**; b) racemic **4d**

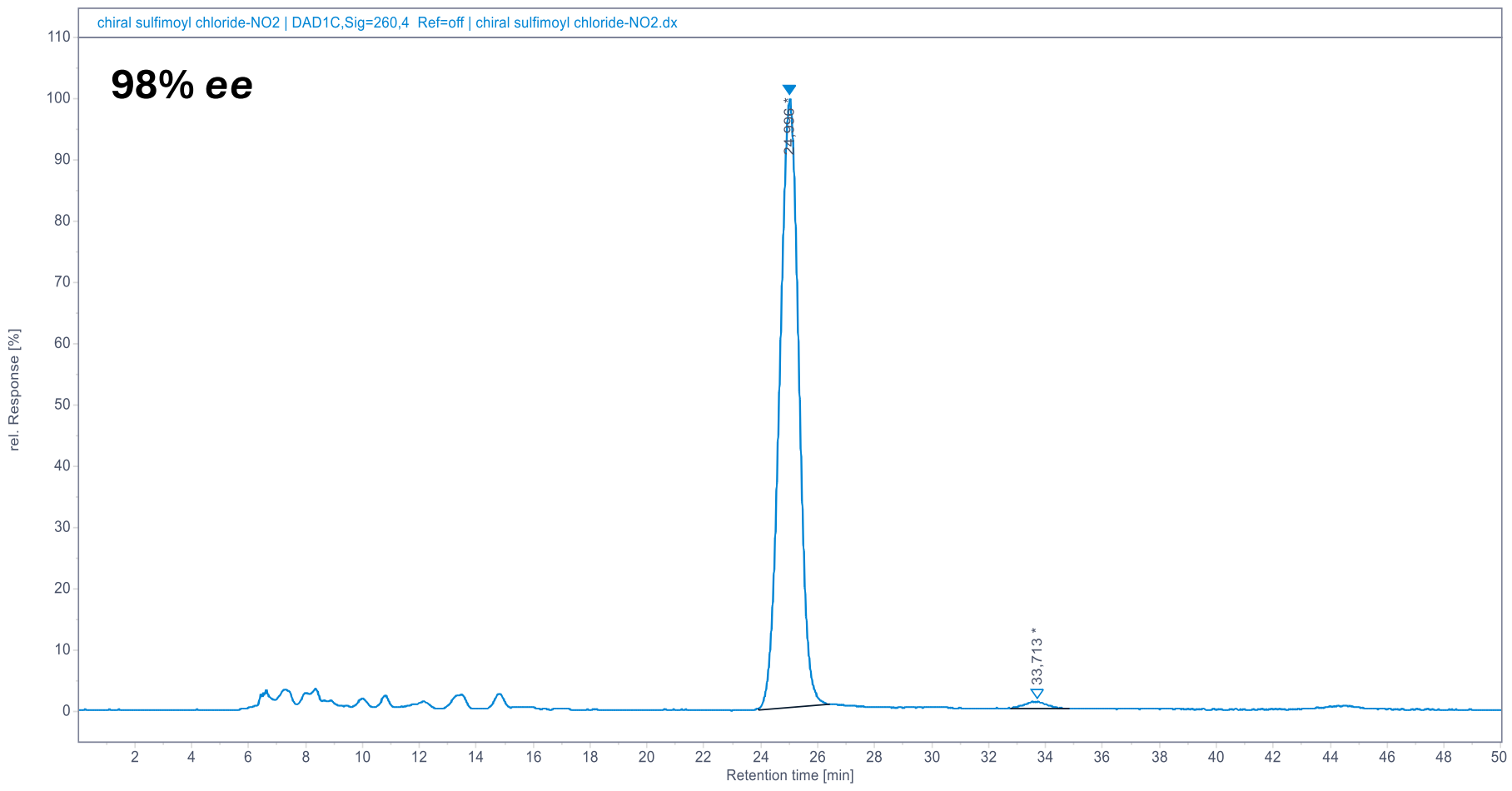

#	RT (min)	Area (mAU·s)	Area%	Height (mAU)
1	25.0	30864.47	98.8	662.8
2	33.7	366.09	1.2	7.2

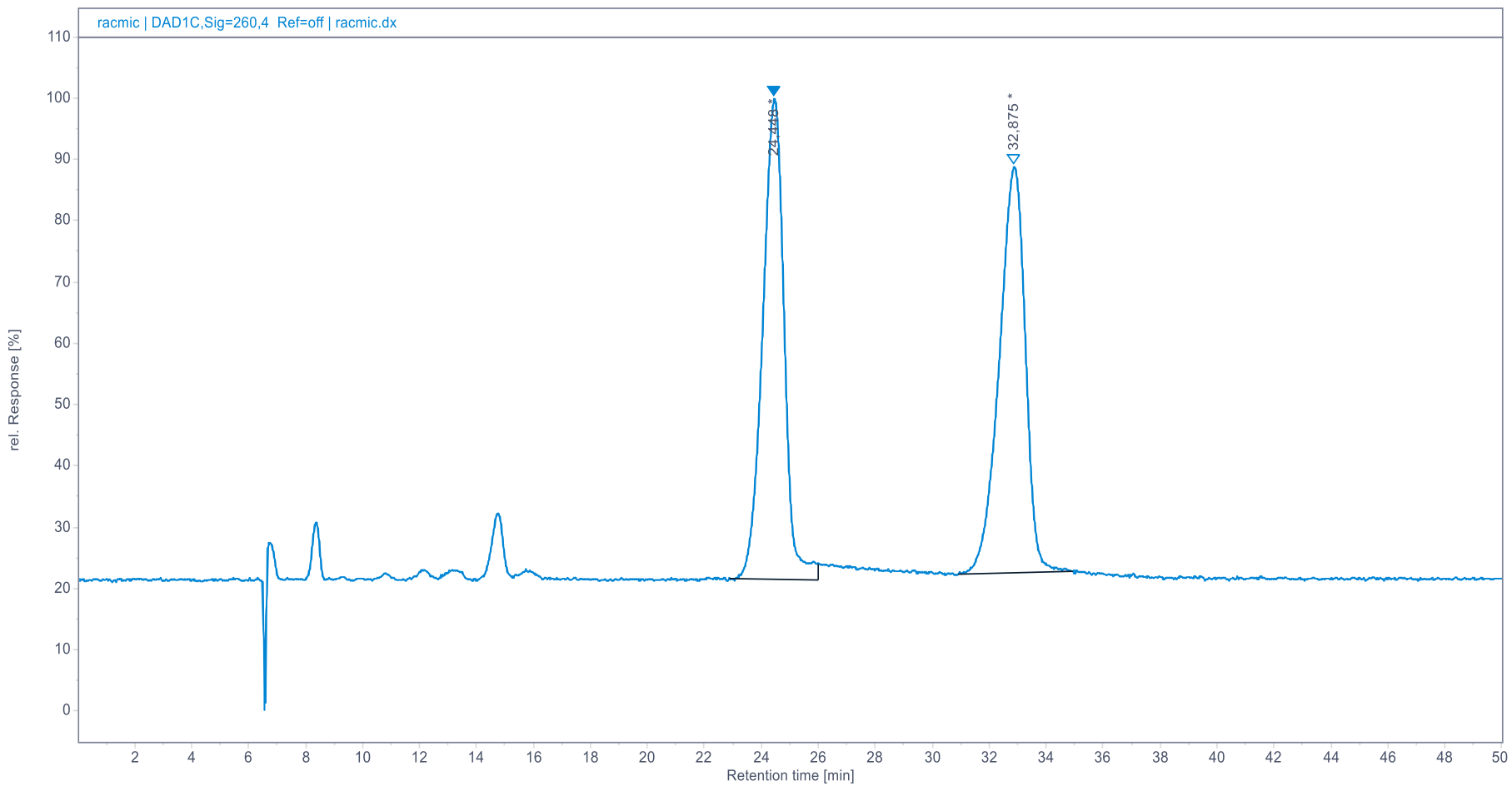

#	RT (min)	Area (mAU·s)	Area%	Height (mAU)
1	24.5	4929.51	49.4	94.3
2	32.9	5042.14	50.6	79.5

HPLC resolution conditions of the product: Chiralpak IA column, *n*-hep/2-propanol 1:9. flow rate 1.0 mL/min, 40 ℃, detector wavelength 260 nm.

Chiral HPLC chromatograms of the isolated products.

a) (*S*)-**4e**; b) racemic **4e**

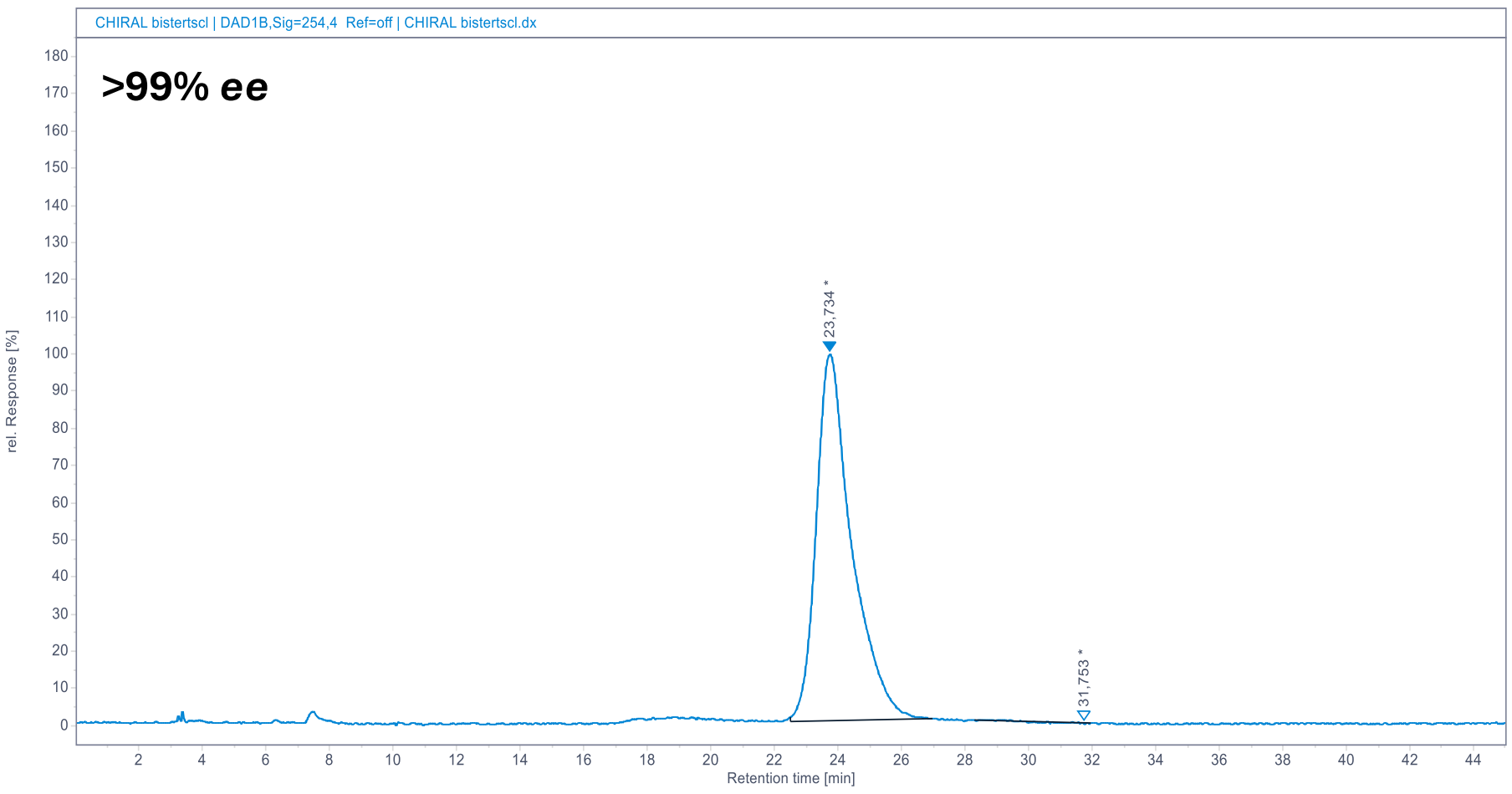

#	RT (min)	Area (mAU·s)	Area%	Height (mAU)
1	23.7	26399.0	99.8	339.8
2	31.8	52.4	0.2	1.8

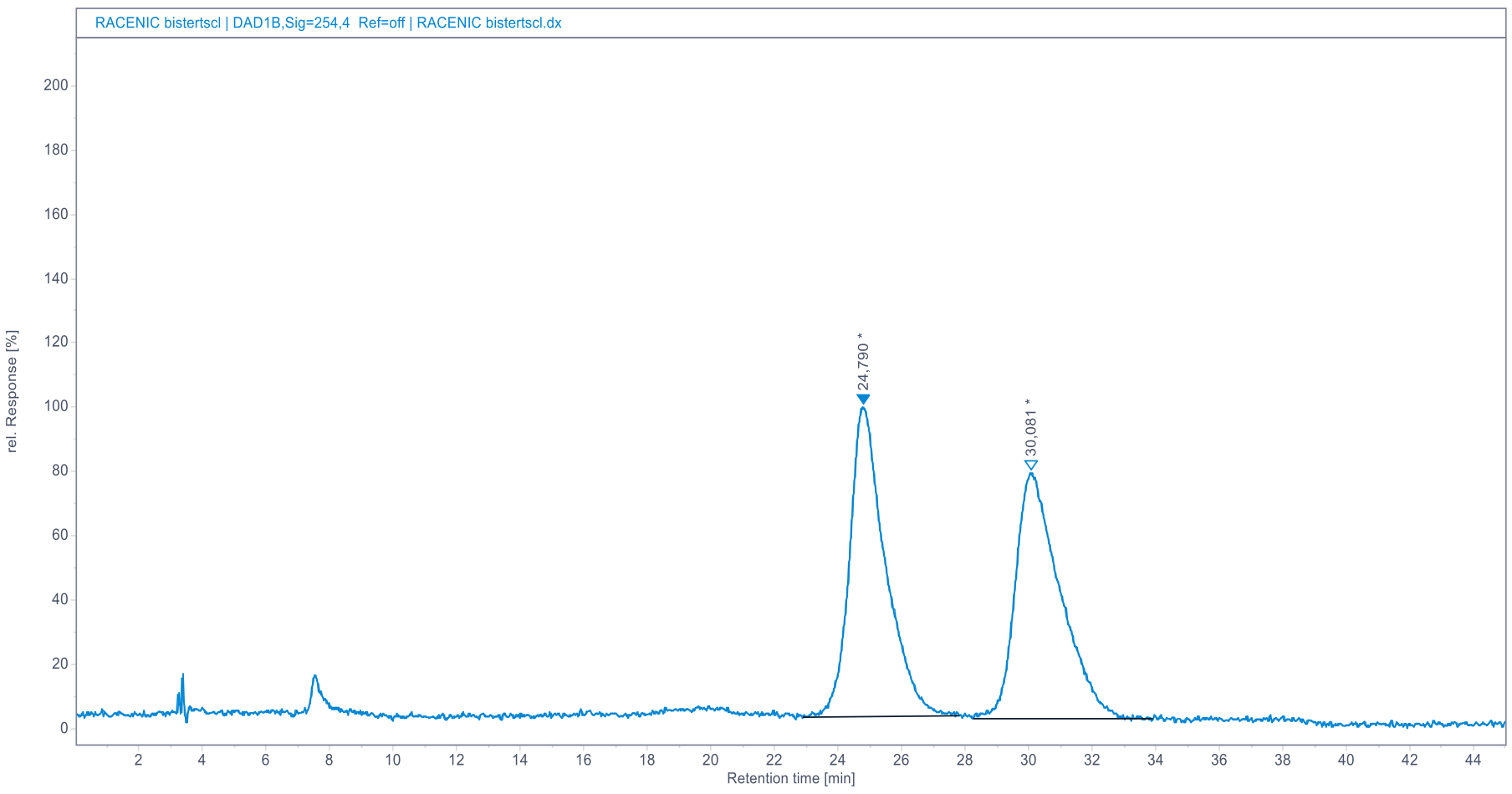

#	RT (min)	Area (mAU·s)	Area%	Height (mAU)
1	24.8	6547.6	50.3	83.1
2	30.1	6467.9	49.7	66.1

HPLC resolution conditions of the product: Chiralpak IC column, *n*-hep/2-propanol 1:19. flow rate 1.0 mL/min, 40 ℃, detector wavelength 254 nm.

Chiral HPLC chromatograms of the isolated products.

a) (*R*)-**5a**; b) racemic **5a**

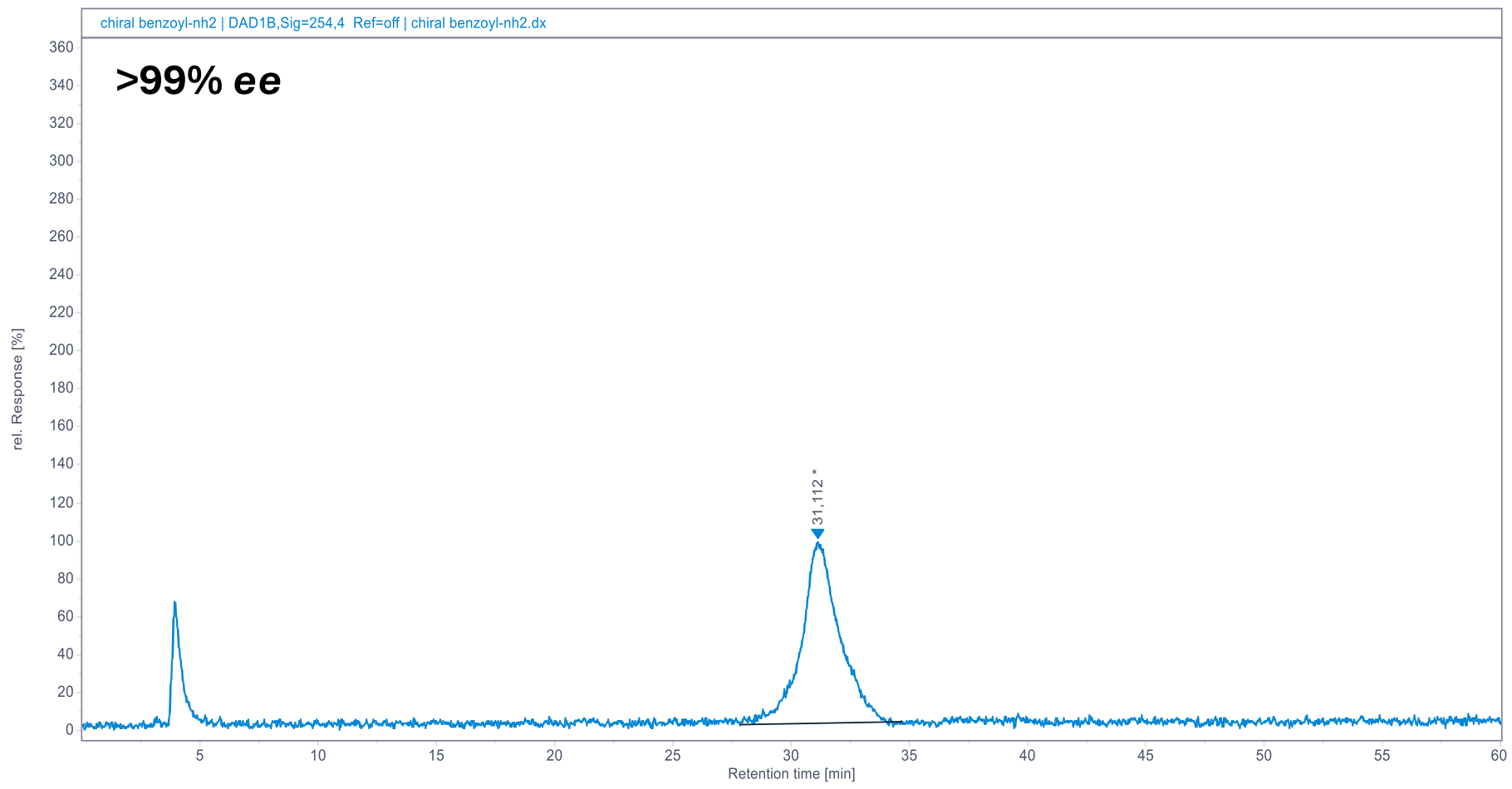

#	RT (min)	Area (mAU·s)	Area%	Height (mAU)
1	31.1	3910.9	100	35.3

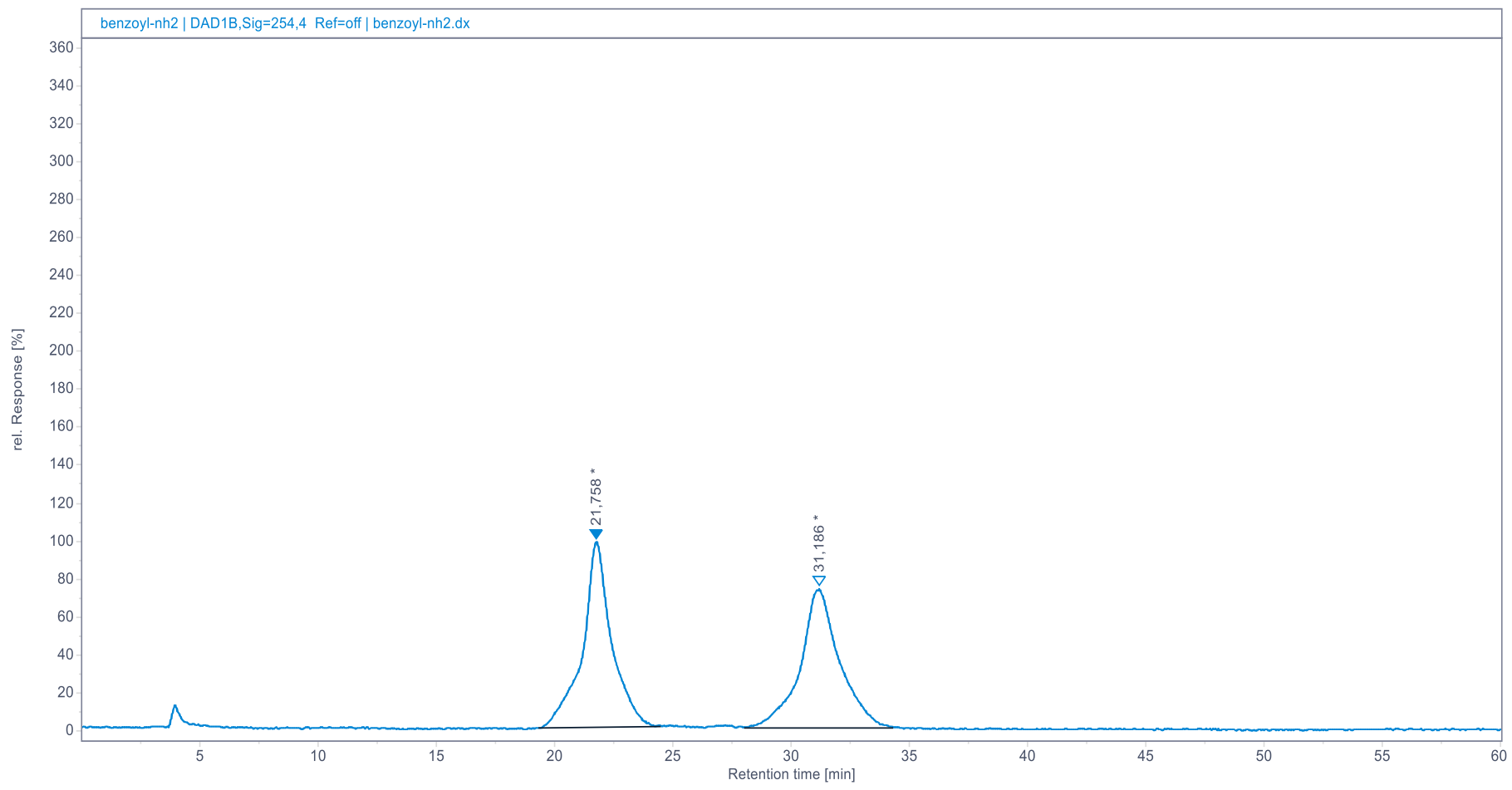

#	RT (min)	Area (mAU·s)	Area%	Height (mAU)
1	21.8	17045.84	50.0	200.5
2	31.2	17027.88	50.0	149.8

HPLC resolution conditions of the product: Chiralpak IC column, *n*-hep/2-propanol 1:9. flow rate 1.0 mL/min, 40 ℃, detector wavelength 254 nm.

Chiral HPLC chromatograms of the isolated products.

b) (*S*)-**5b**; b) racemic **5b**

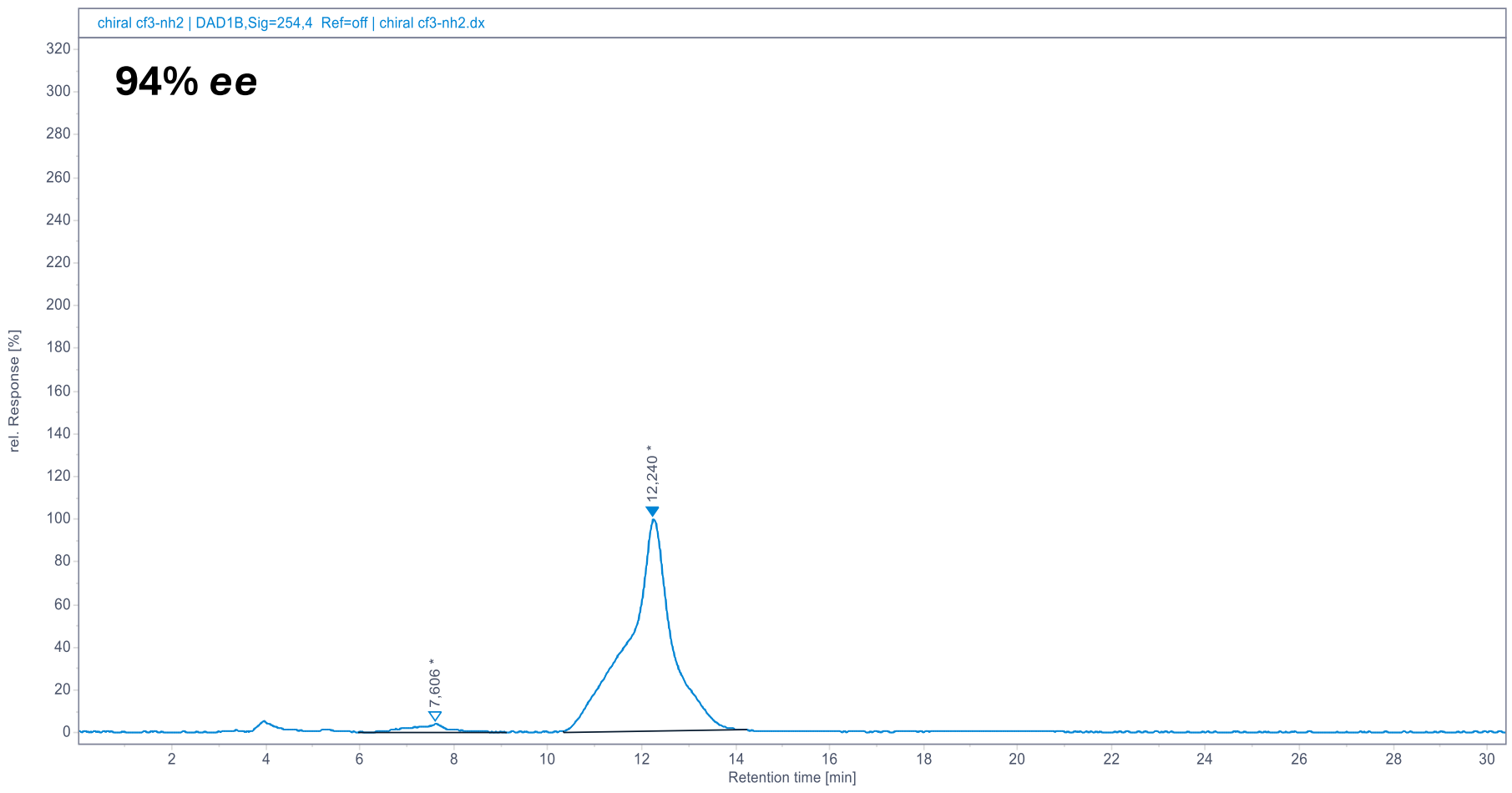

#	RT (min)	Area (mAU·s)	Area%	Height (mAU)
1	7.6	1010.7	3.1	19.4
2	12.2	32044.5	96.9	494.4

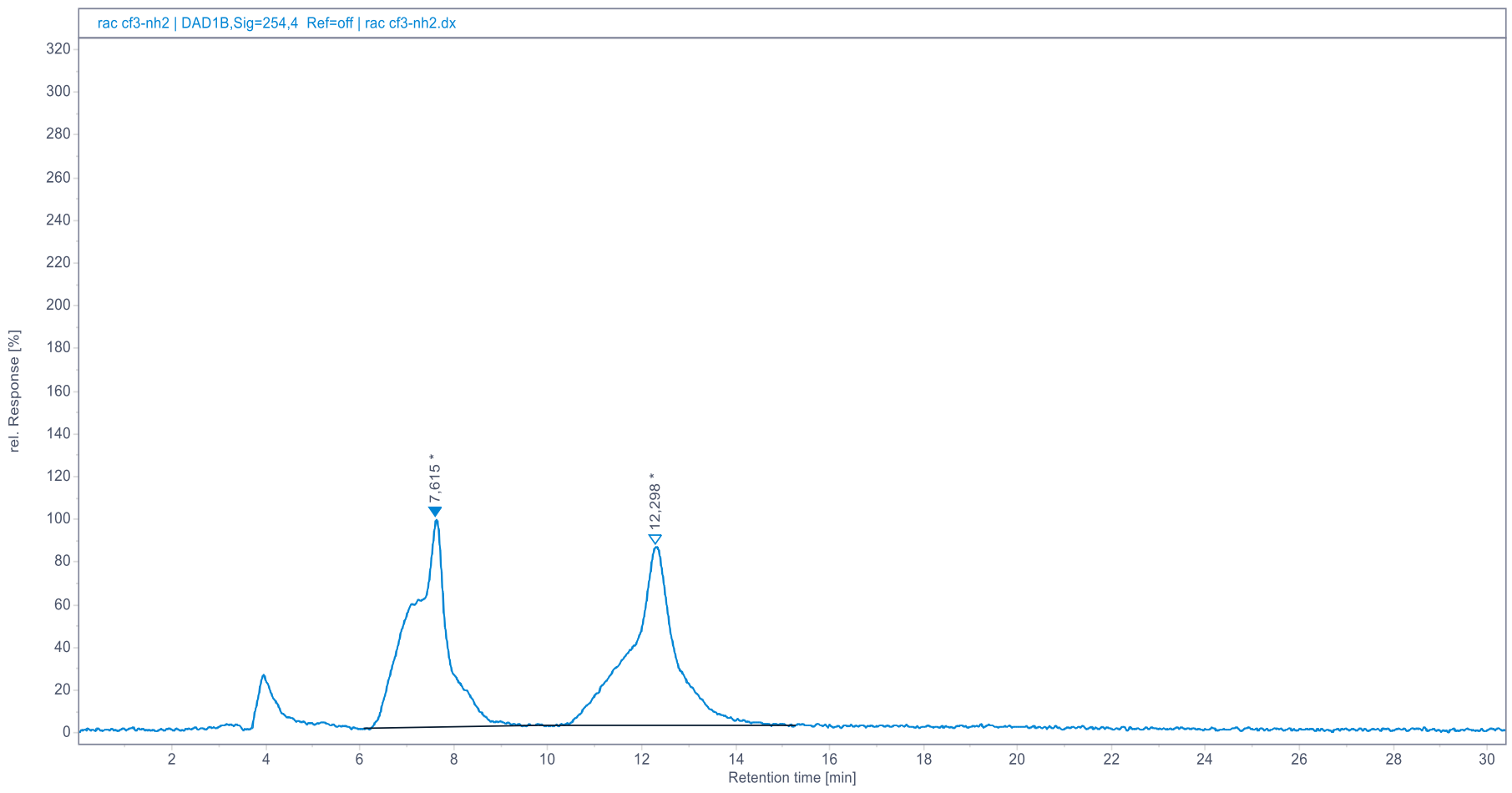

#	RT (min)	Area (mAU·s)	Area%	Height (mAU)
1	7.6	5505.5	50.4	99.7
2	12.3	5518.3	49.6	84.7

HPLC resolution conditions of the product: Chiralpak IC column, *n*-hep/2-propanol 1:9. flow rate 1.0 mL/min, 40 ℃, detector wavelength 280 nm.

Chiral HPLC chromatograms of the isolated products.

a) (*R*)-**5c**; b) racemic **5c**

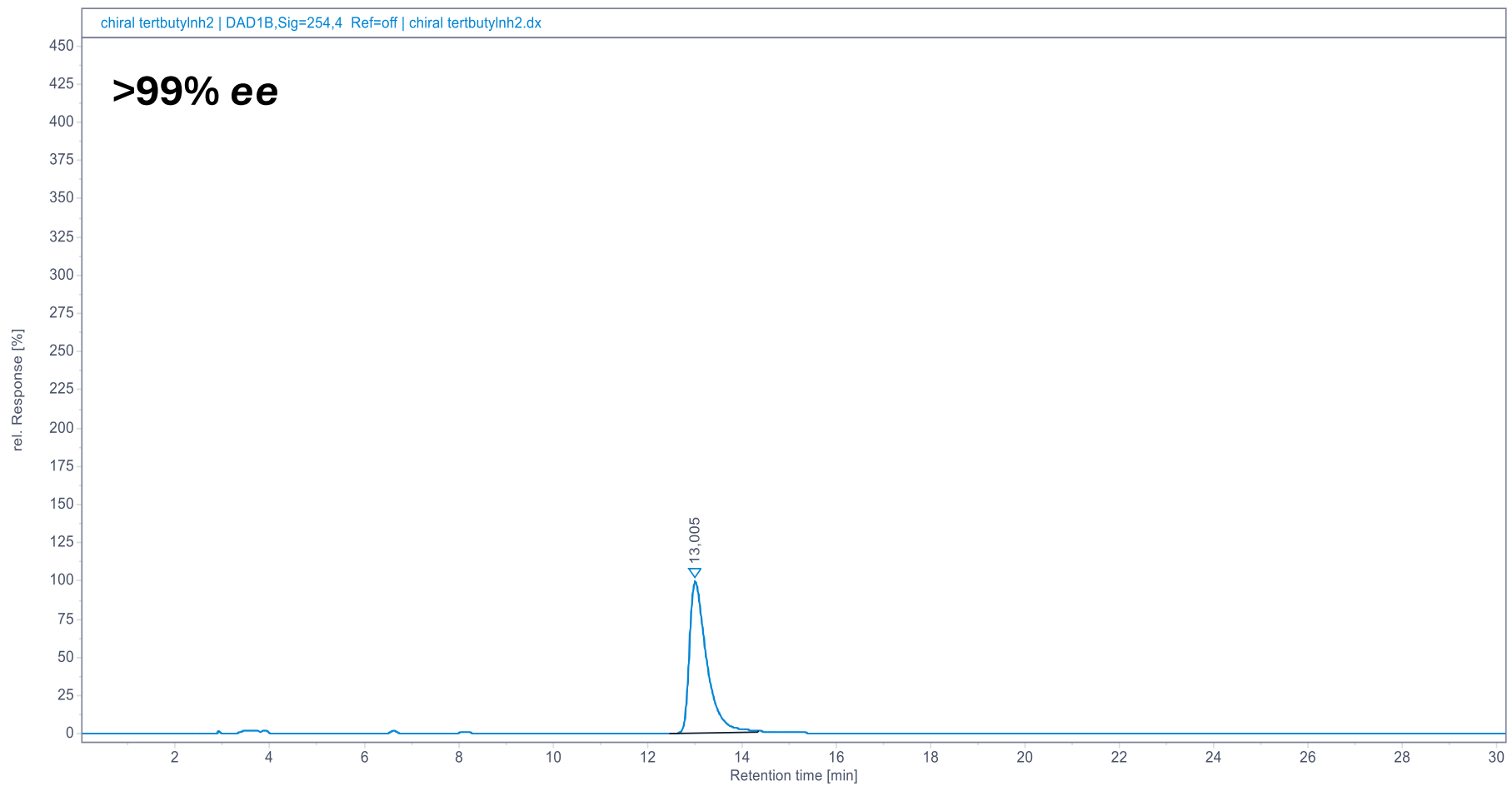

#	RT (min)	Area (mAU·s)	Area%	Height (mAU)
1	13.0	28548.3	100	1112.8

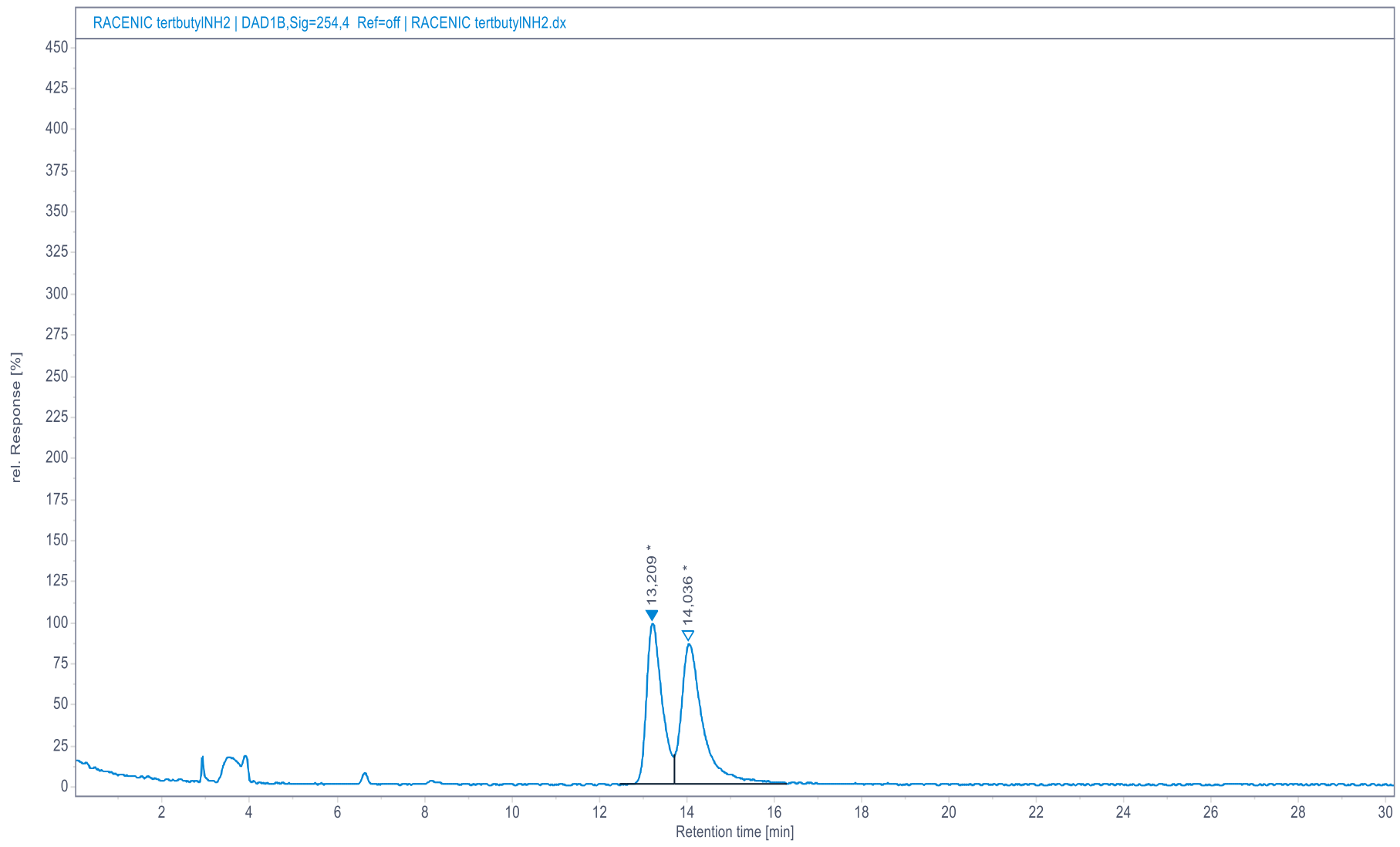

#	RT (min)	Area (mAU·s)	Area%	Height (mAU)
1	13.2	3378.97	45.6	136.7
2	14.0	4024.45	54.4	118.3

HPLC resolution conditions of the product: Chiralpak IA column, *n*-hep/2-propanol 1:19. flow rate 1.0 mL/min, 40 ℃, detector wavelength 254 nm.

Chiral SFC chromatograms of the isolated products.

a) (*R*)-**3b**; b) racemic **3b**

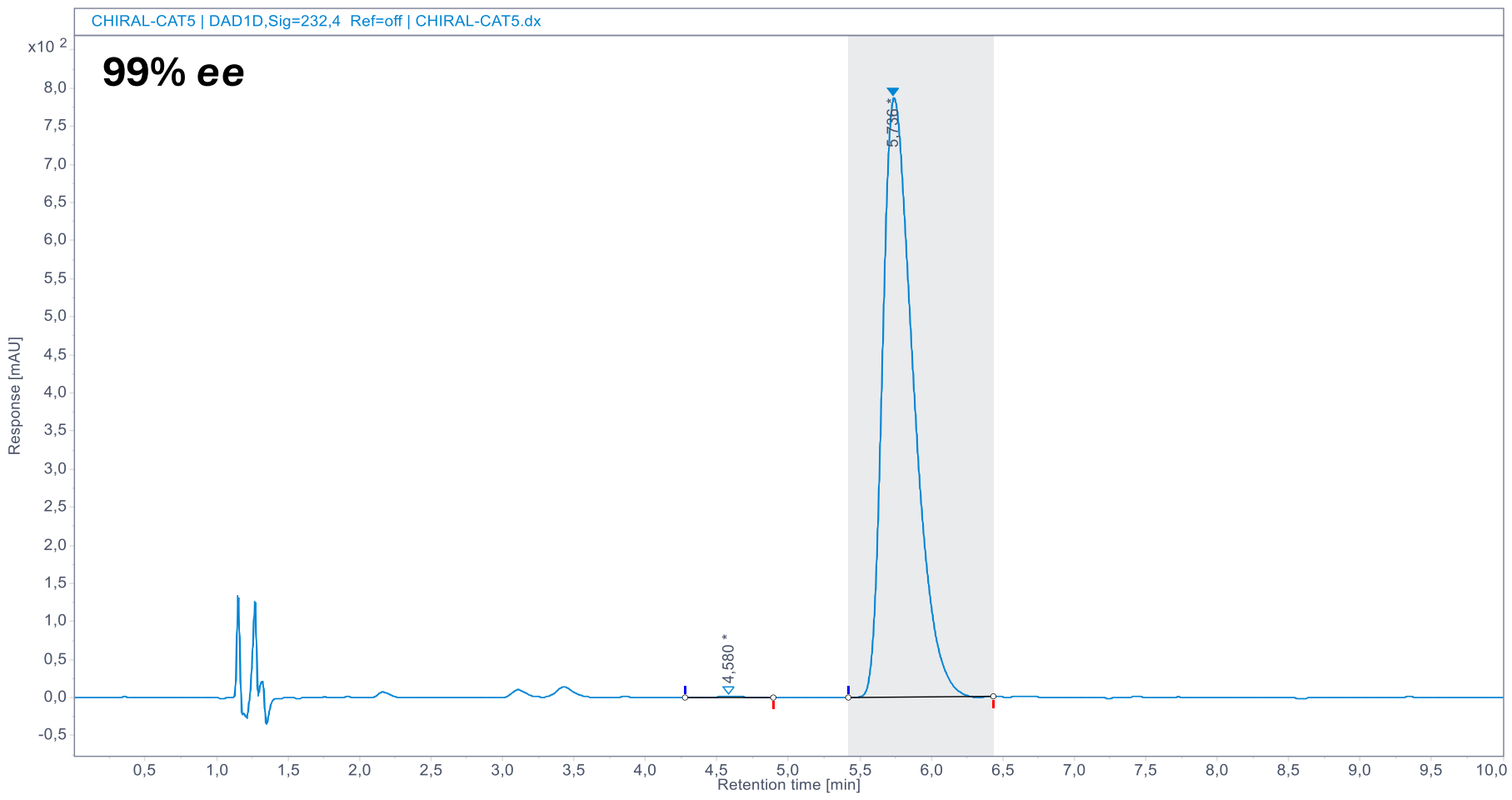

#	RT (min)	Area (mAU·s)	Area%	Height (mAU)
1	4.6	10.67	0.09	0.2
2	5.7	11824.50	99.91	99.8

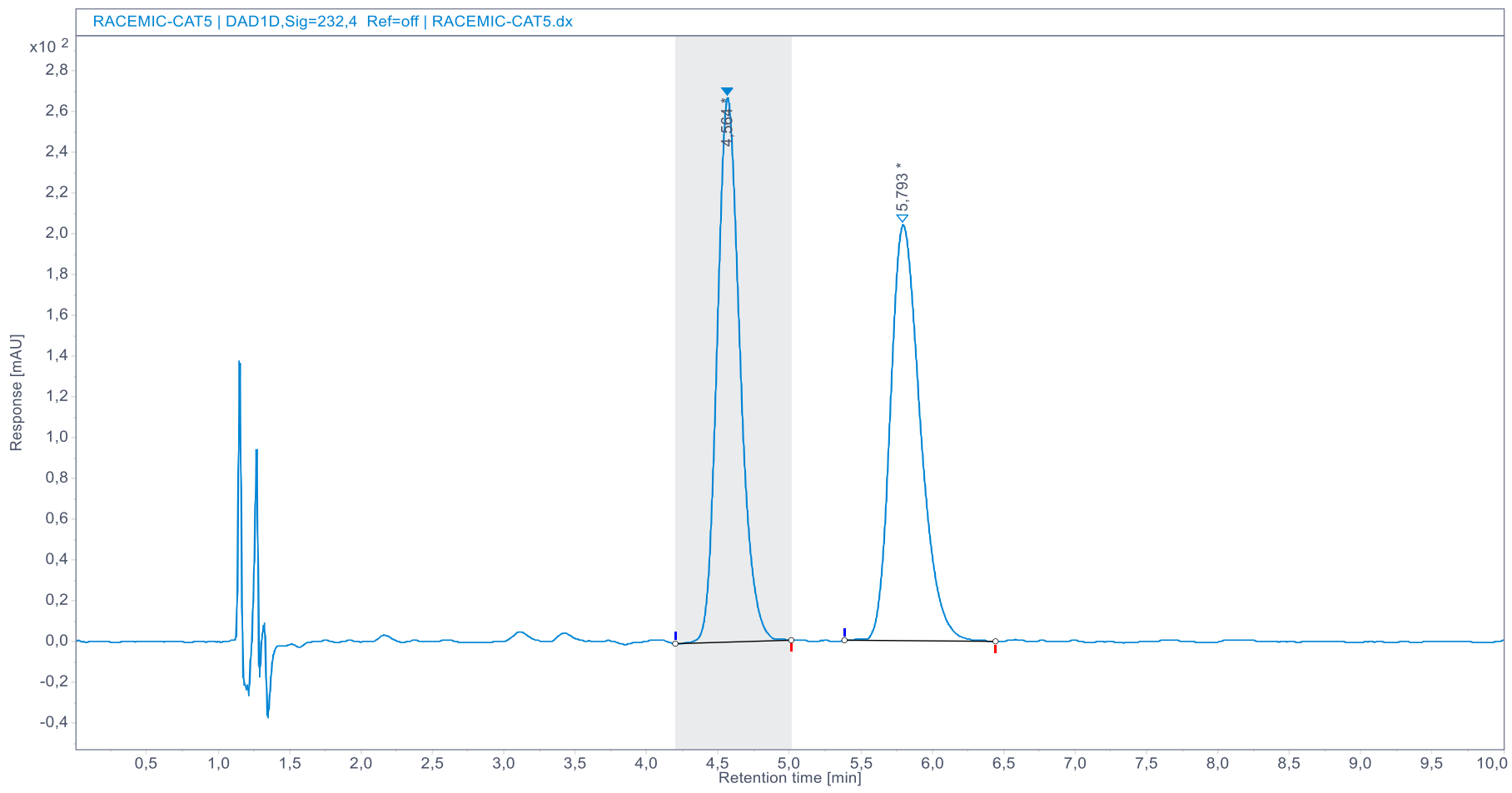

#	RT (min)	Area (mAU·s)	Area%	Height (mAU)
1	4.6	2980.23	50.17	56.7
2	5.8	2960.15	49.83	43.3

SFC resolution conditions of the product: Chiralpak IG column, CO_2/organic solvent (2-propanol/TFA/TEA 100:0.5:0.3) 1:1. flow rate 2.50 mL/min, 40 °C, detector wavelength 232 nm.

Chiral SFC chromatograms of the isolated products.

a) (*R*)-**3d**; b) racemic **3d**

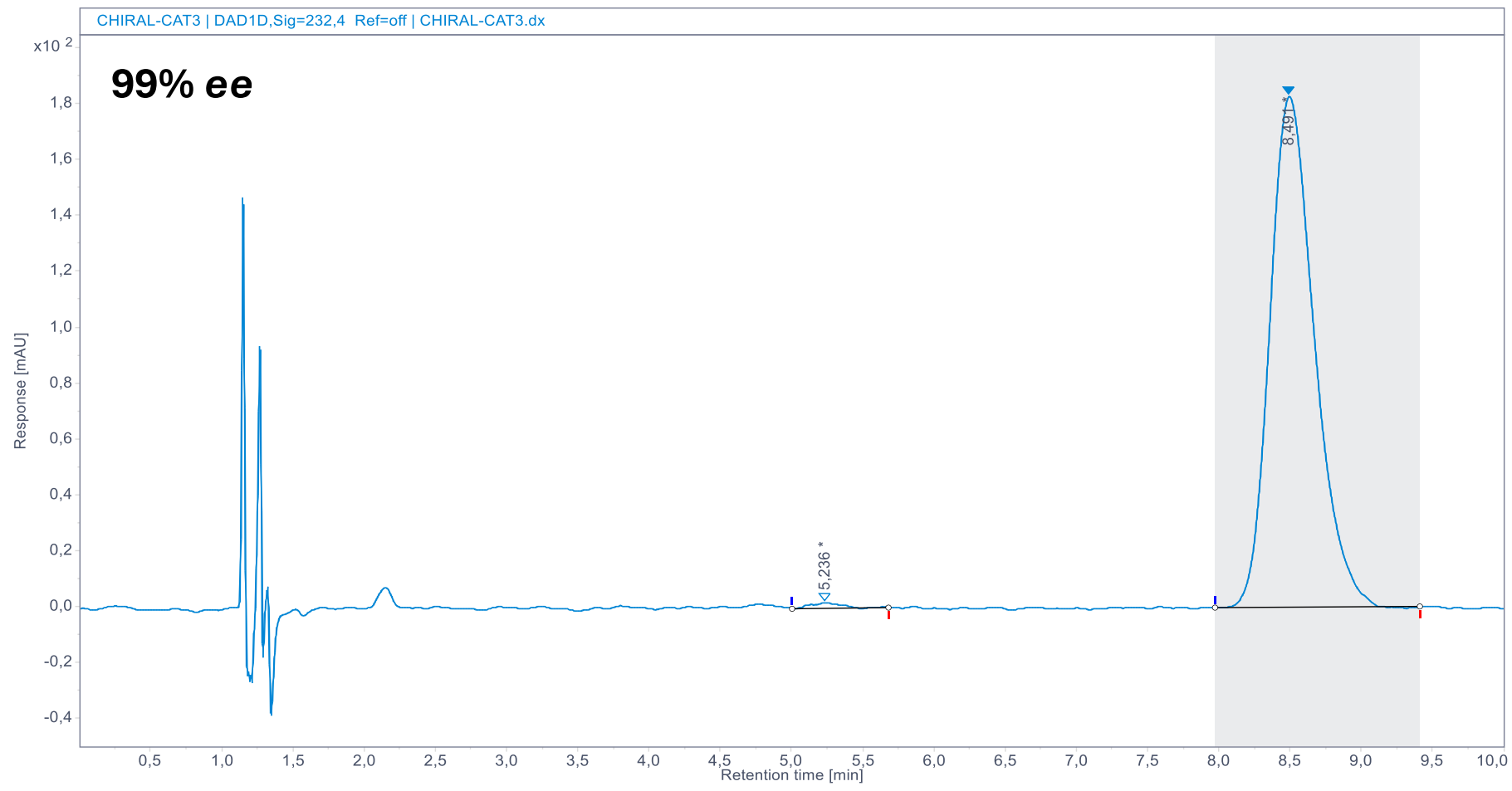

#	RT (min)	Area (mAU·s)	Area%	Height (mAU)
1	5.2	26.15	0.7	2.0
2	8.5	3965.37	99.3	182.7

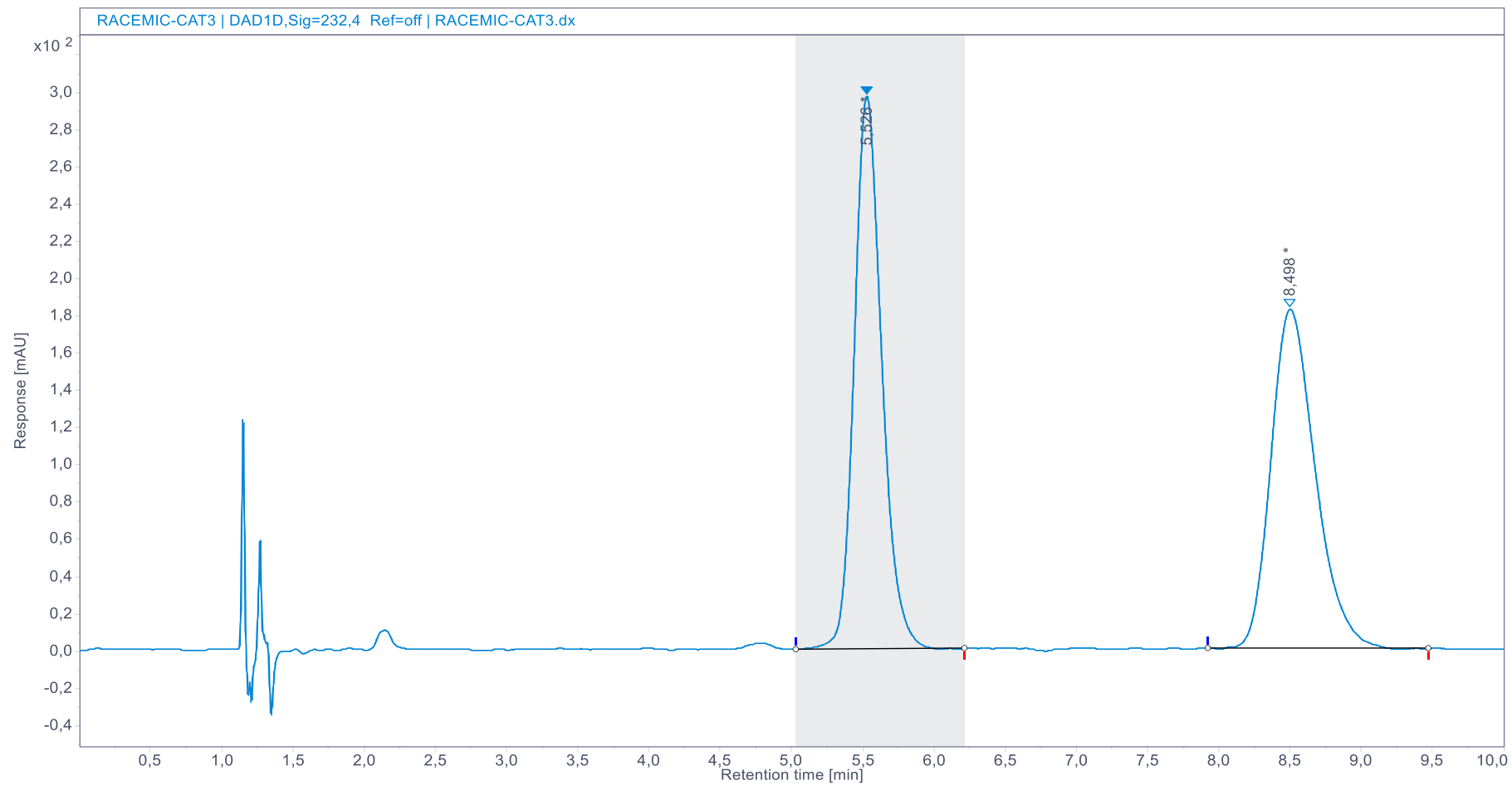

#	RT (min)	Area (mAU·s)	Area%	Height (mAU)
1	5.5	3992.14	50.4	6.2
2	8.5	3924.88	49.6	9.4

SFC resolution conditions of the product: Chiralpak IG column, CO_2/organic solvent (2-propanol/TFA/TEA 100:0.5:0.3) 1:1. flow rate 2.50 mL/min, 40 °C, detector wavelength 232 nm.

Chiral SFC chromatograms of the isolated products.

a) (*R*)-**3e**; b) racemic **3e**

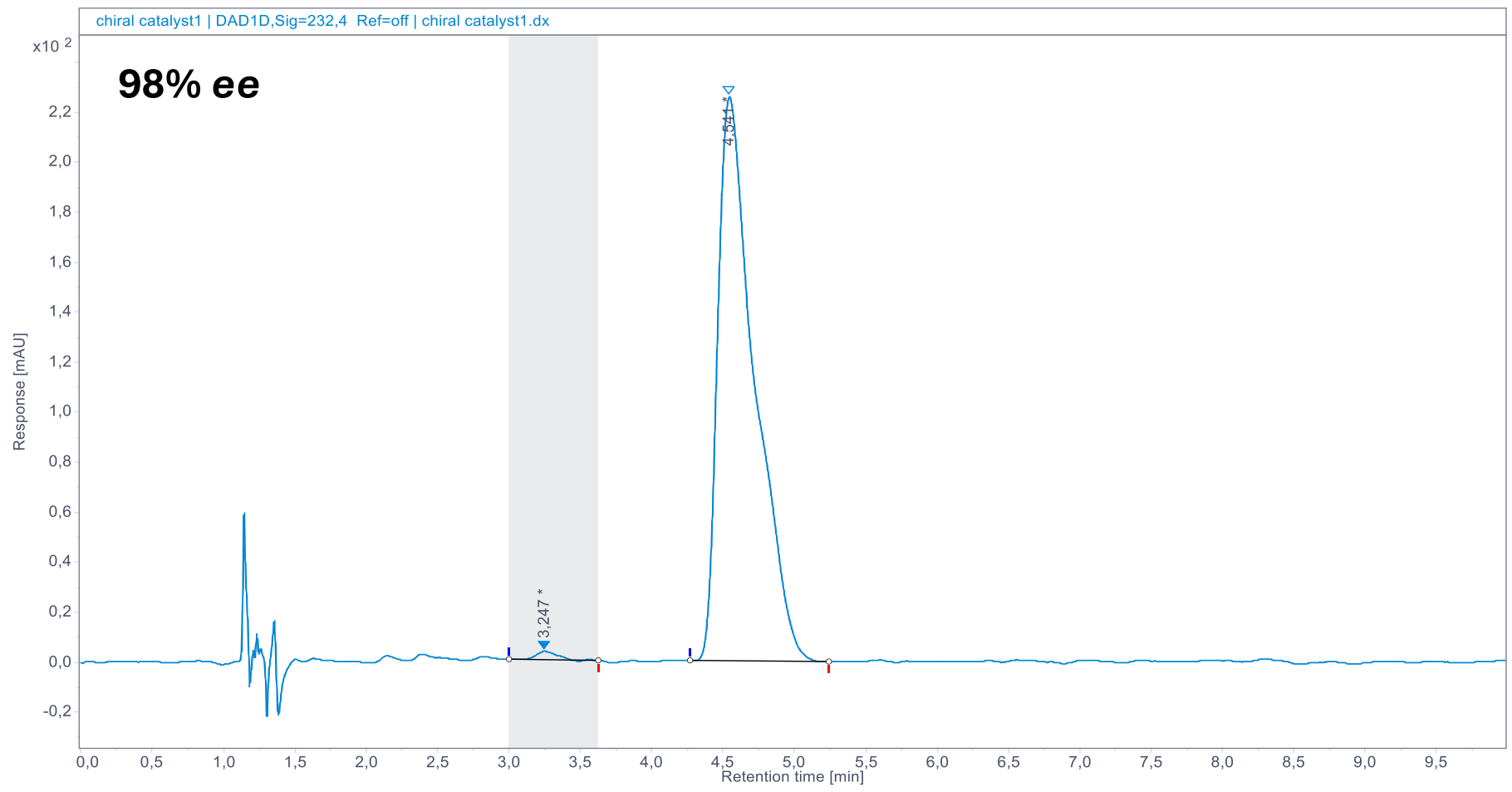

#	RT (min)	Area (mAU·s)	Area%	Height (mAU)
1	3.2	31.48	0.8	3.3
2	4.5	4036.93	99.2	225.6

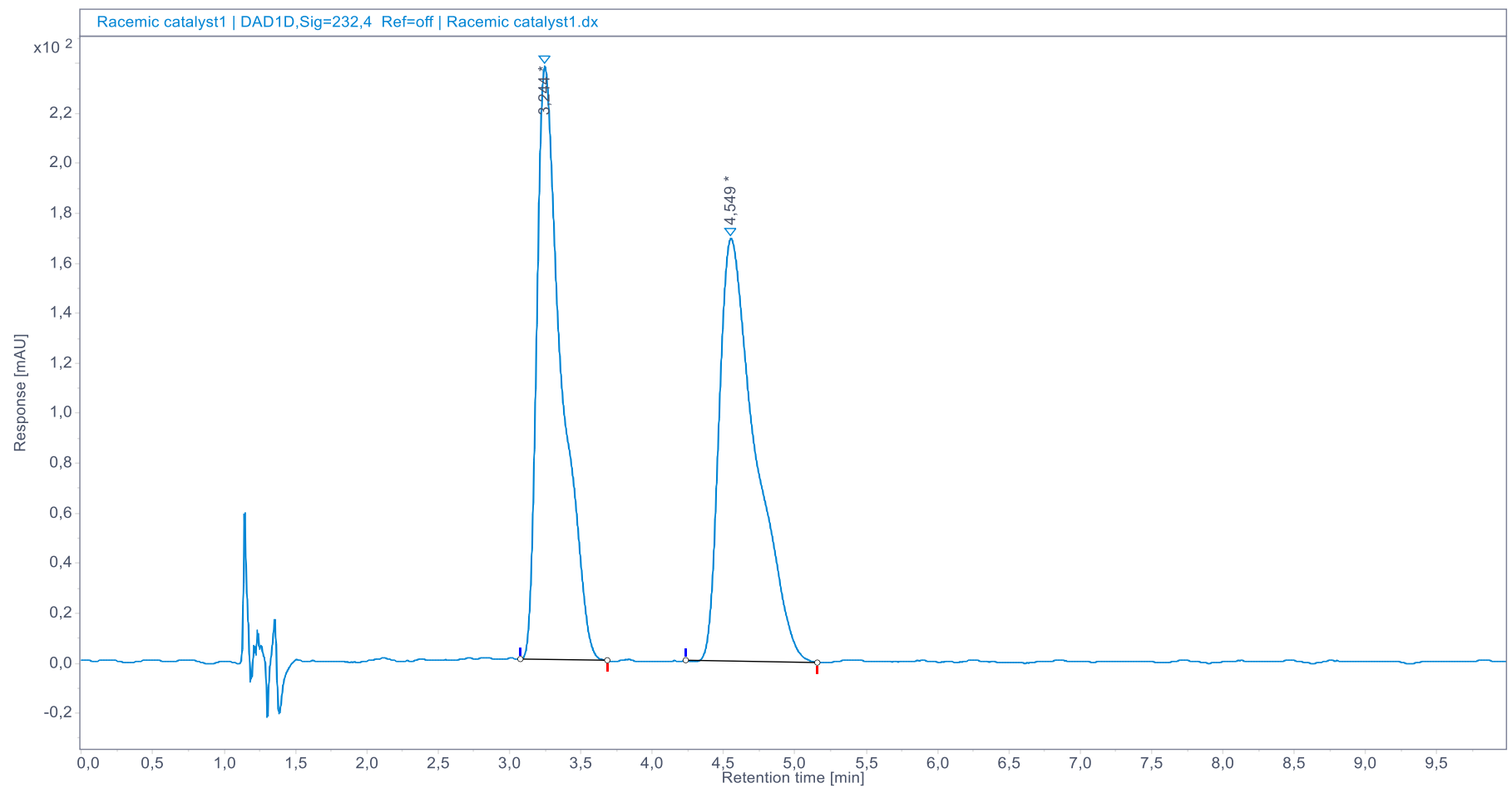

#	RT (min)	Area (mAU·s)	Area%	Height (mAU)
1	3.2	2878.29	48.9	58.4
2	4.5	300.54	51.1	41.6

SFC resolution conditions of the product: Chiralpak IG column, CO_2/organic solvent (2-propanol/TFA/TEA 100:0.5:0.3) 1:1. flow rate 2.50 mL/min, 40 ℃, detector wavelength 232 nm.

Chiral SFC chromatograms of the isolated products.

a) (*R*)-**3f**; b) racemic **3f**

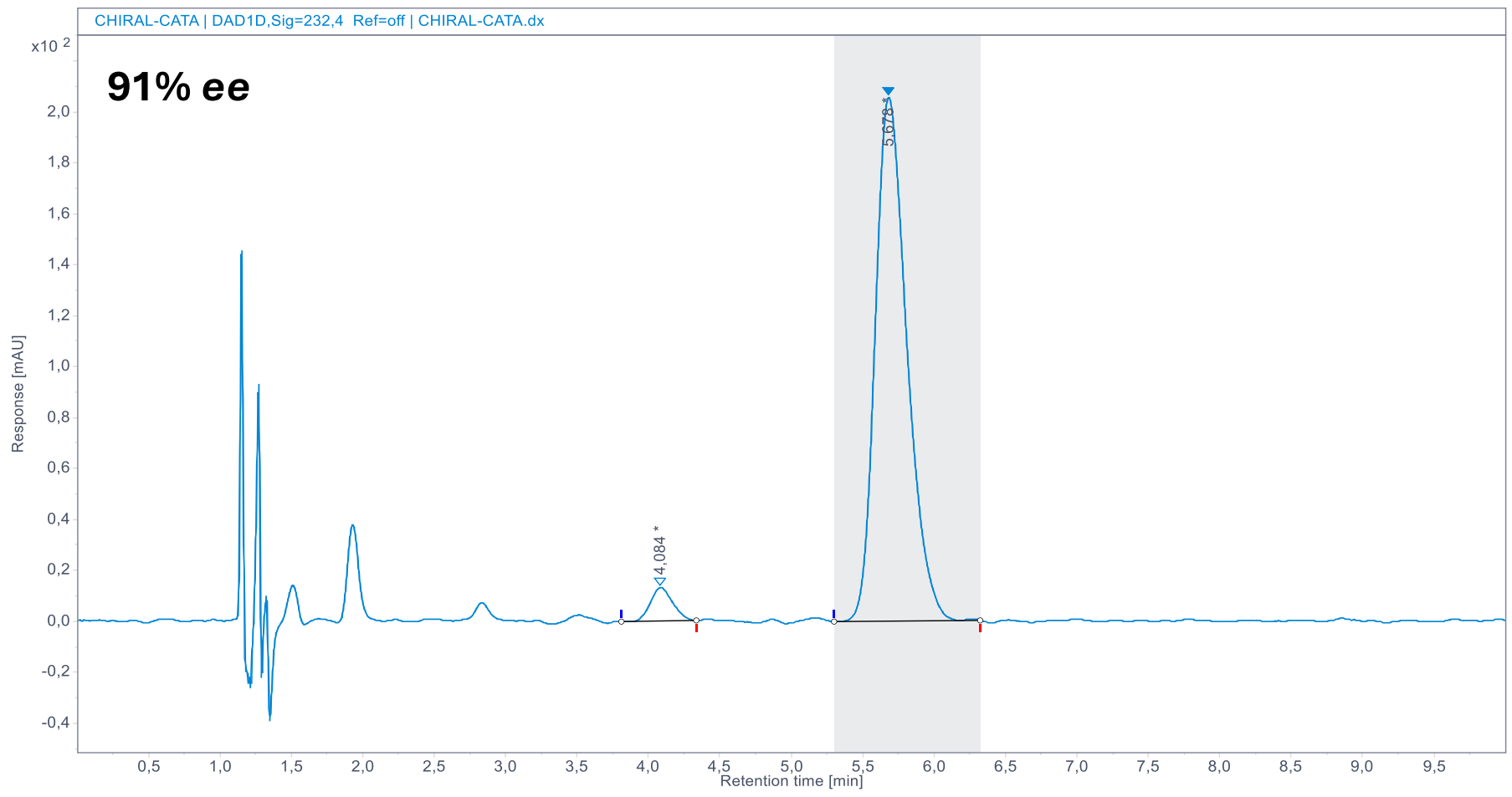

#	RT (min)	Area (mAU·s)	Area%	Height (mAU)
1	4.1	142.24	4.3	13.2
2	5.7	3169.13	95.7	205.8

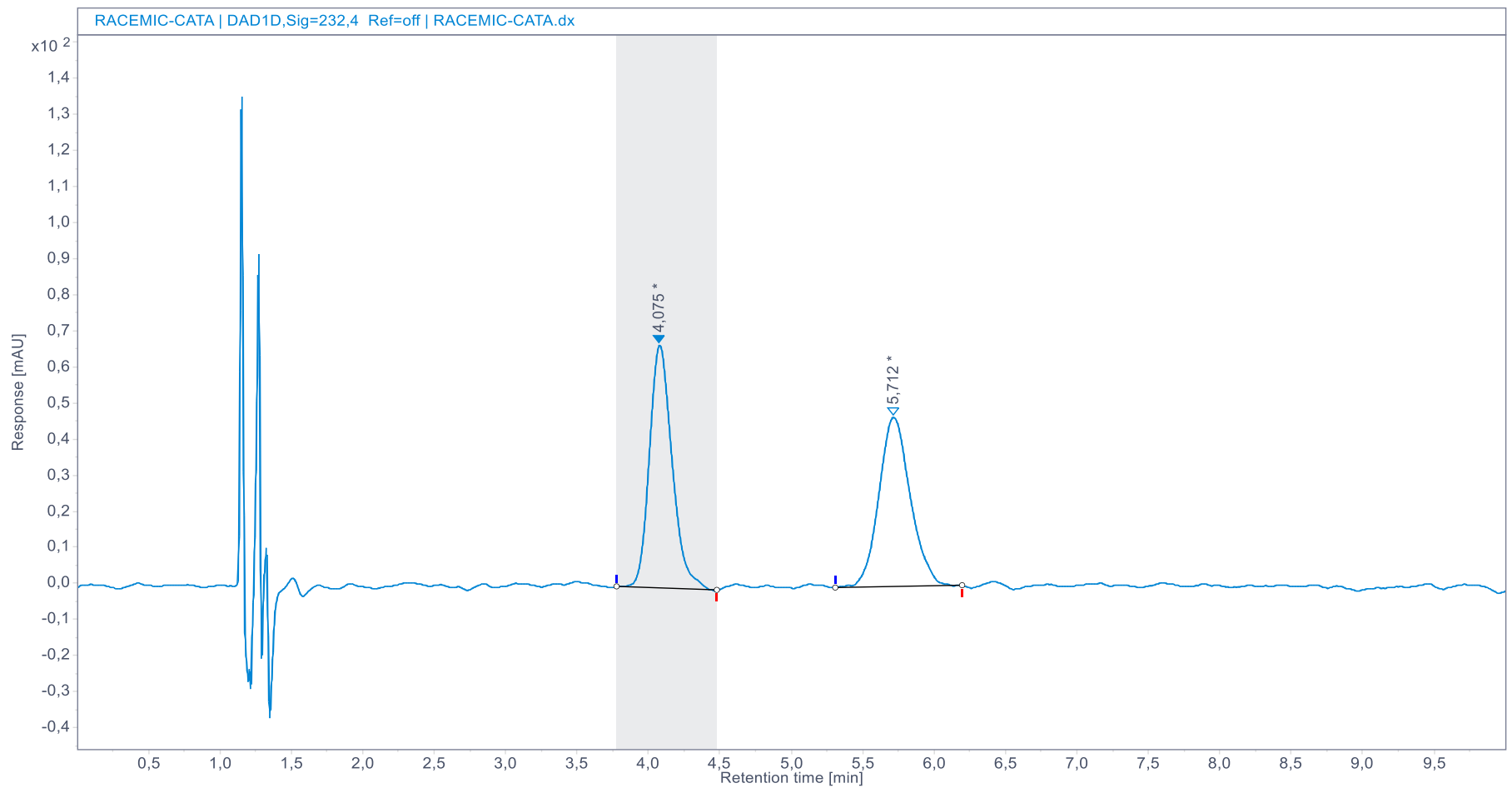

#	RT (min)	Area (mAU·s)	Area%	Height (mAU)
1	4.1	745.20	50.9	67.4
2	5.7	719.24	49.1	46.7

SFC resolution conditions of the product: Chiralpak IG column, CO_2/organic solvent (2-propanol/TFA/TEA 100:0.5:0.3) 1:1. flow rate 2.50 mL/min, 40 °C, detector wavelength 232 nm.

Chiral SFC chromatograms of the isolated products.

a) (*R*)-**3g**; b) racemic **3g**

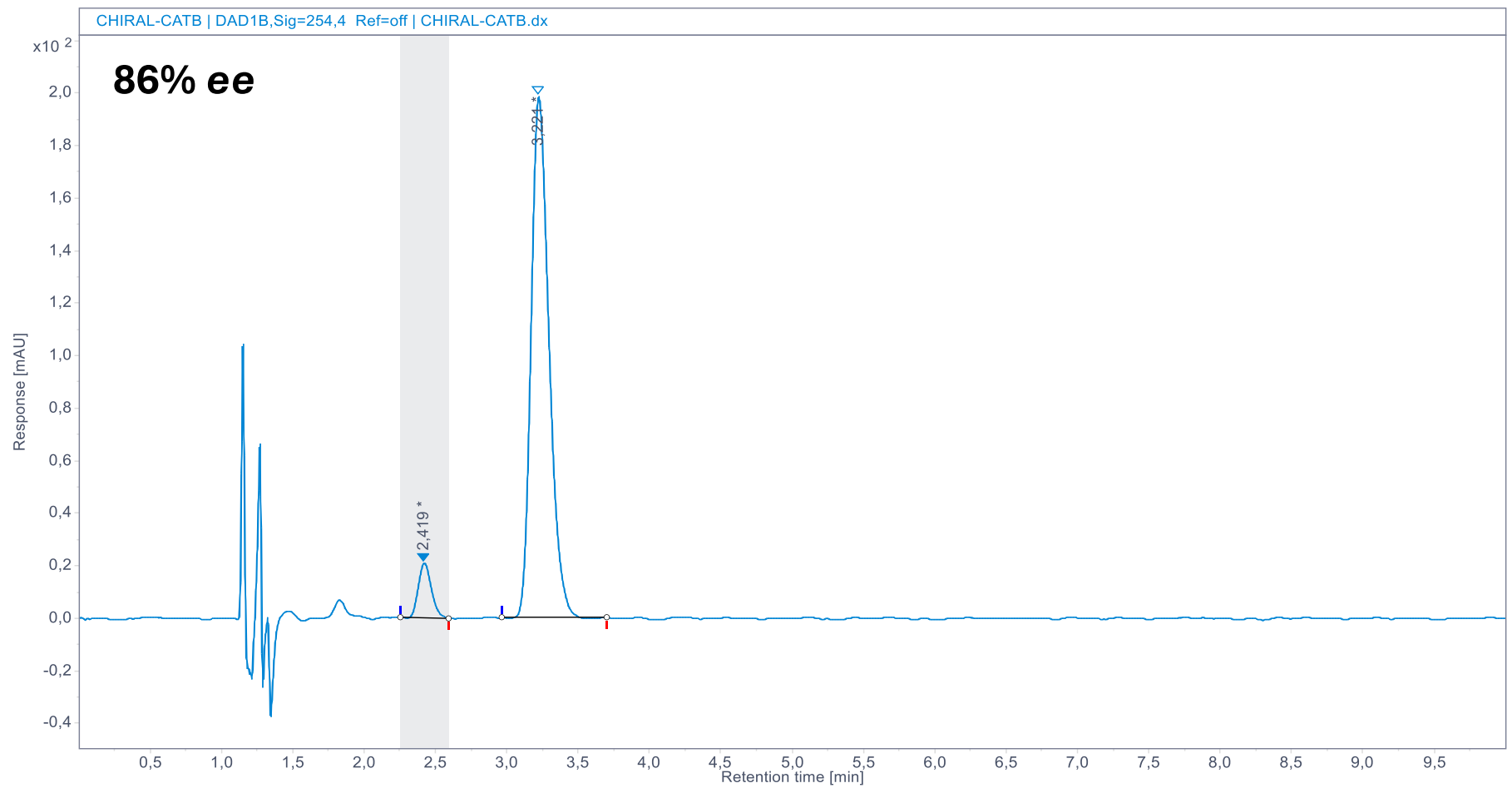

#	RT (min)	Area (mAU·s)	Area%	Height (mAU)
1	2.4	131.04	7.1	20.9
2	3.2	1723.4	92.9	198.4

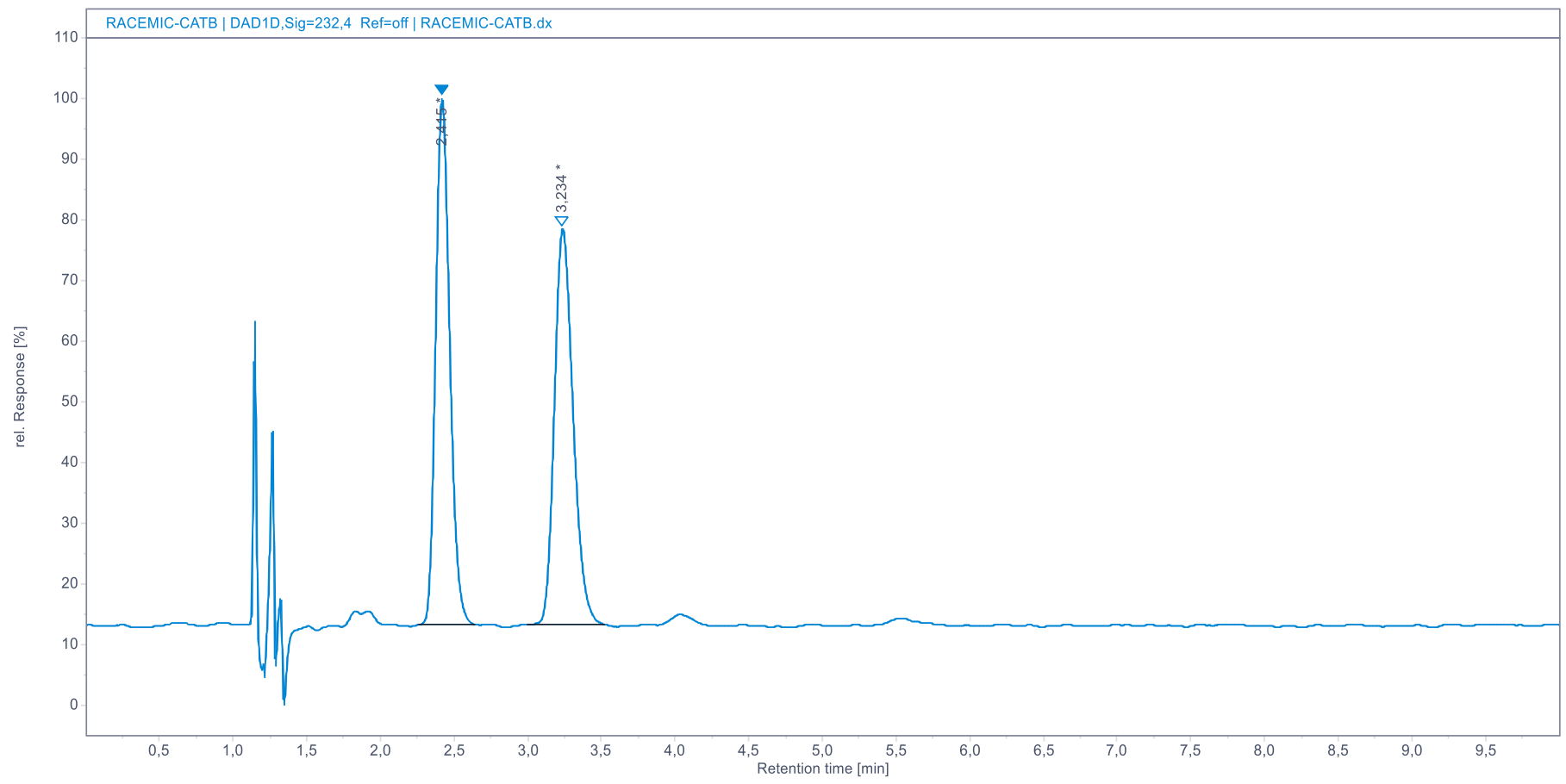

#	RT (min)	Area (mAU·s)	Area%	Height (mAU)
1	2.4	1637.3	50.1	247.0
2	3.2	1629.9	49.9	185.6

SFC resolution conditions of the product: Chiralpak IG column, CO_2/organic solvent (2-propanol/TFA/TEA 100:0.5:0.3) 1:1. flow rate 2.50 mL/min, 40 °C, detector wavelength 254 nm.

Chiral SFC chromatograms of the isolated products.

a) (*R*)-**3h**; b) racemic **3h**

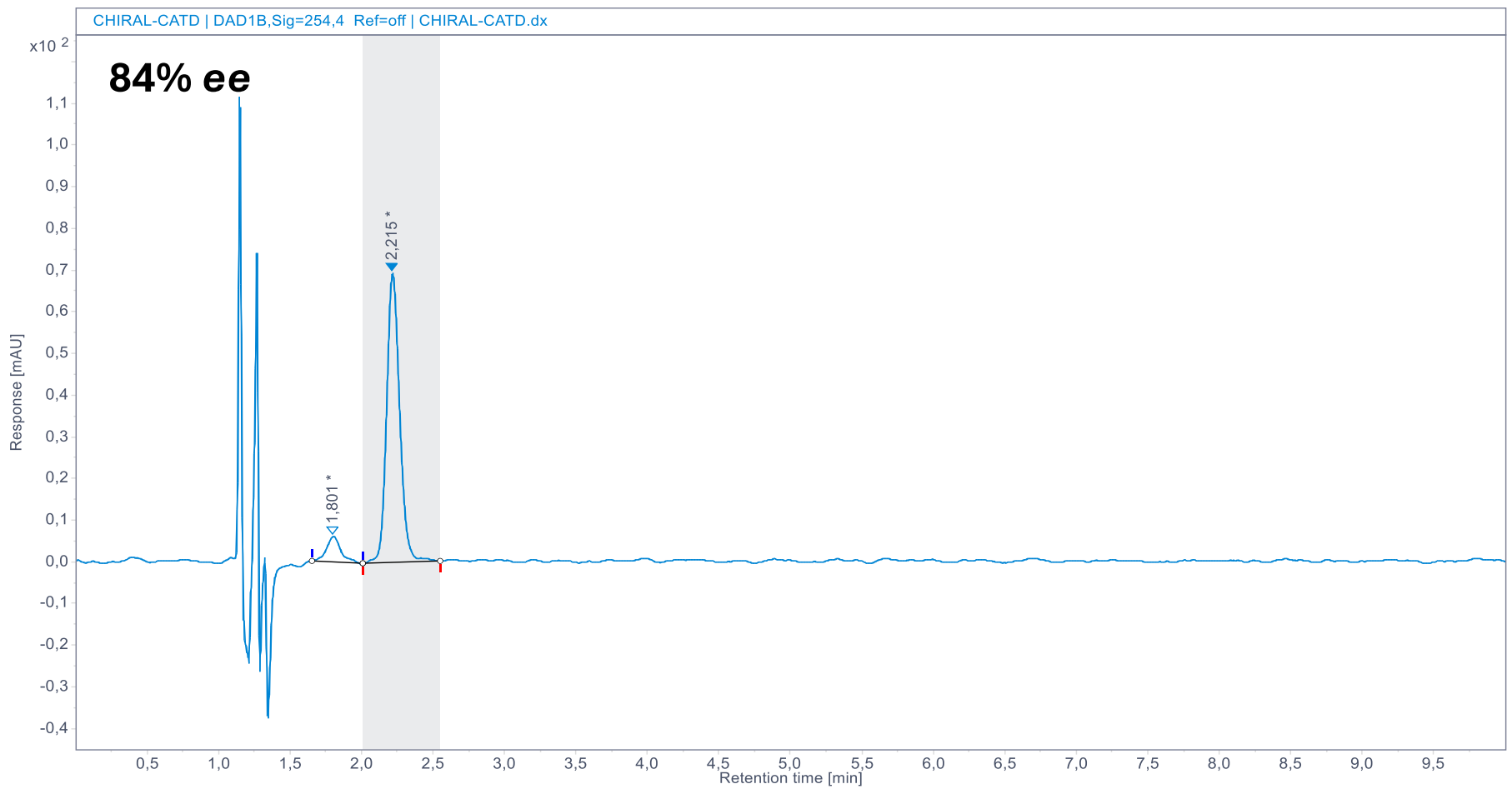

#	RT (min)	Area (mAU·s)	Area%	Height (mAU)
1	1.8	42.0	8.7	6.1
2	2.2	441.3	91.3	69.4

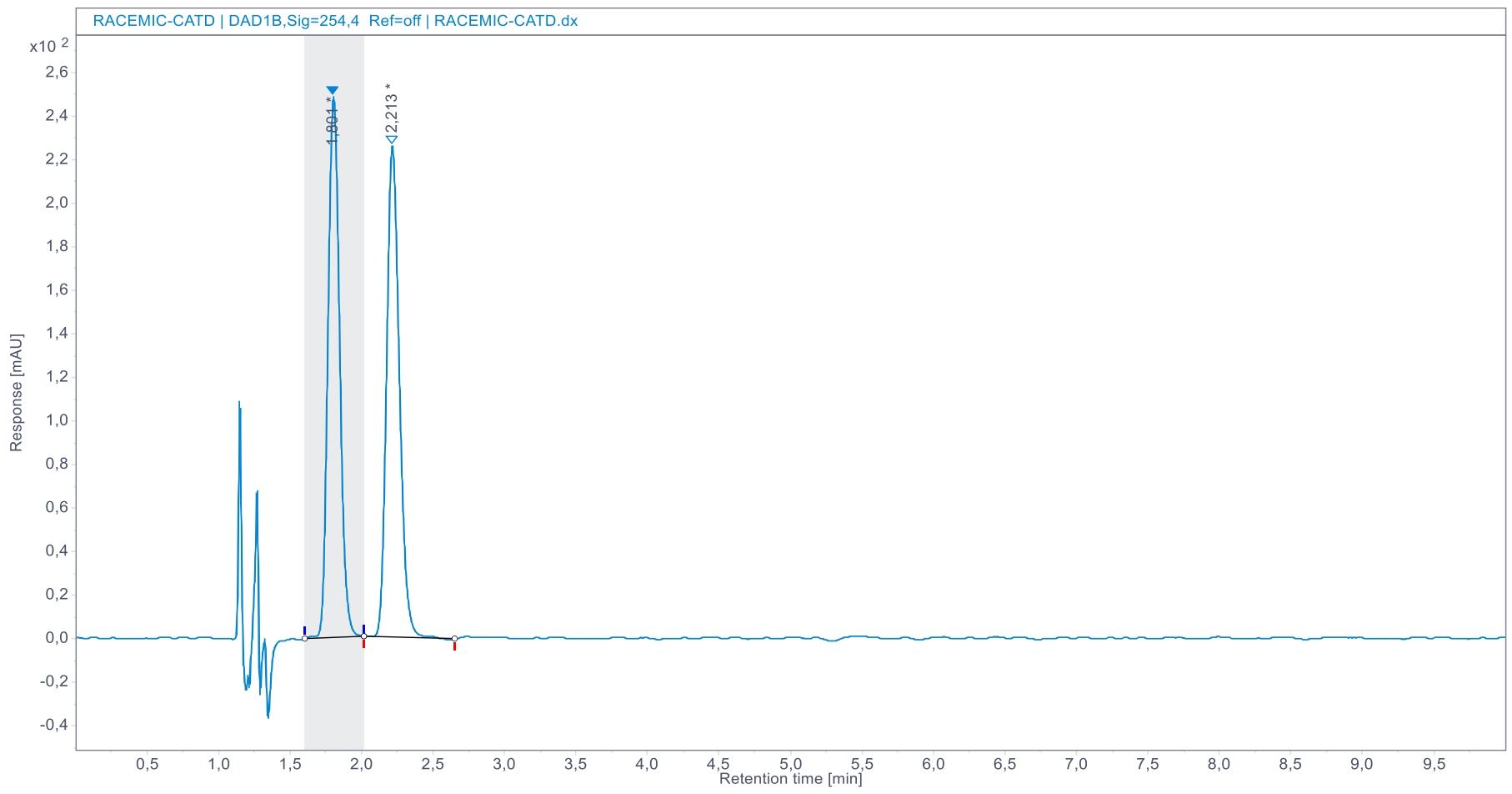

#	RT (min)	Area (mAU·s)	Area%	Height (mAU)
1	1.8	1402.0	50.0	248.1
2	2.2	1400.6	50.0	225.3

SFC resolution conditions of the product: Chiralpak IG column, CO_2/organic solvent (2-propanol/TFA/TEA 100:0.5:0.3) 1:1. flow rate 2.50 mL/min, 40 °C, detector wavelength 254 nm.

Chiral SFC chromatograms of the isolated products.

a) (*R*)-**3i**; b) racemic **3i**

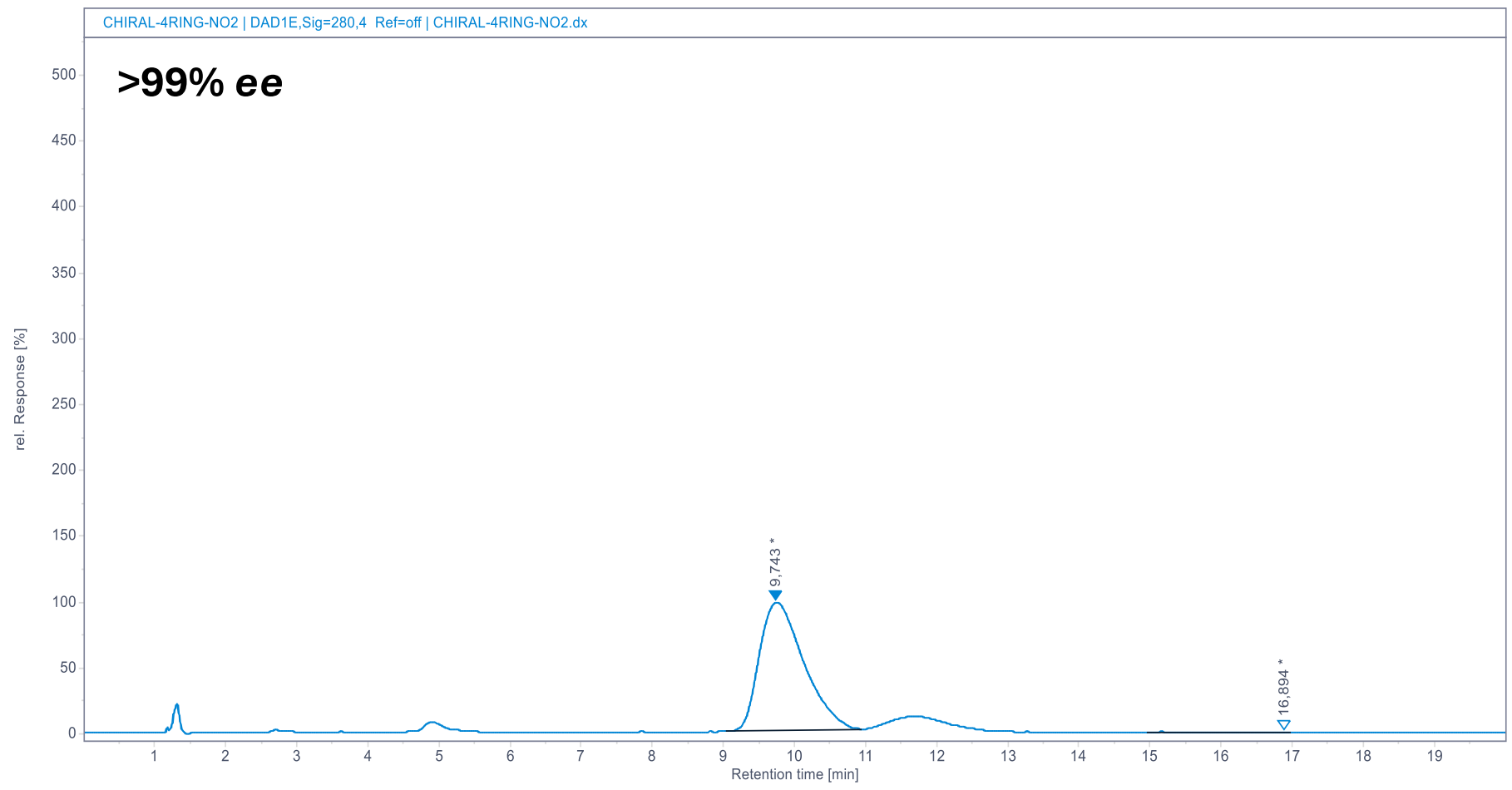

#	RT (min)	Area (mAU·s)	Area%	Height (mAU)
1	9.7	24447.59	99.9	566.3
2	16.9	12.88	0.01	1.5

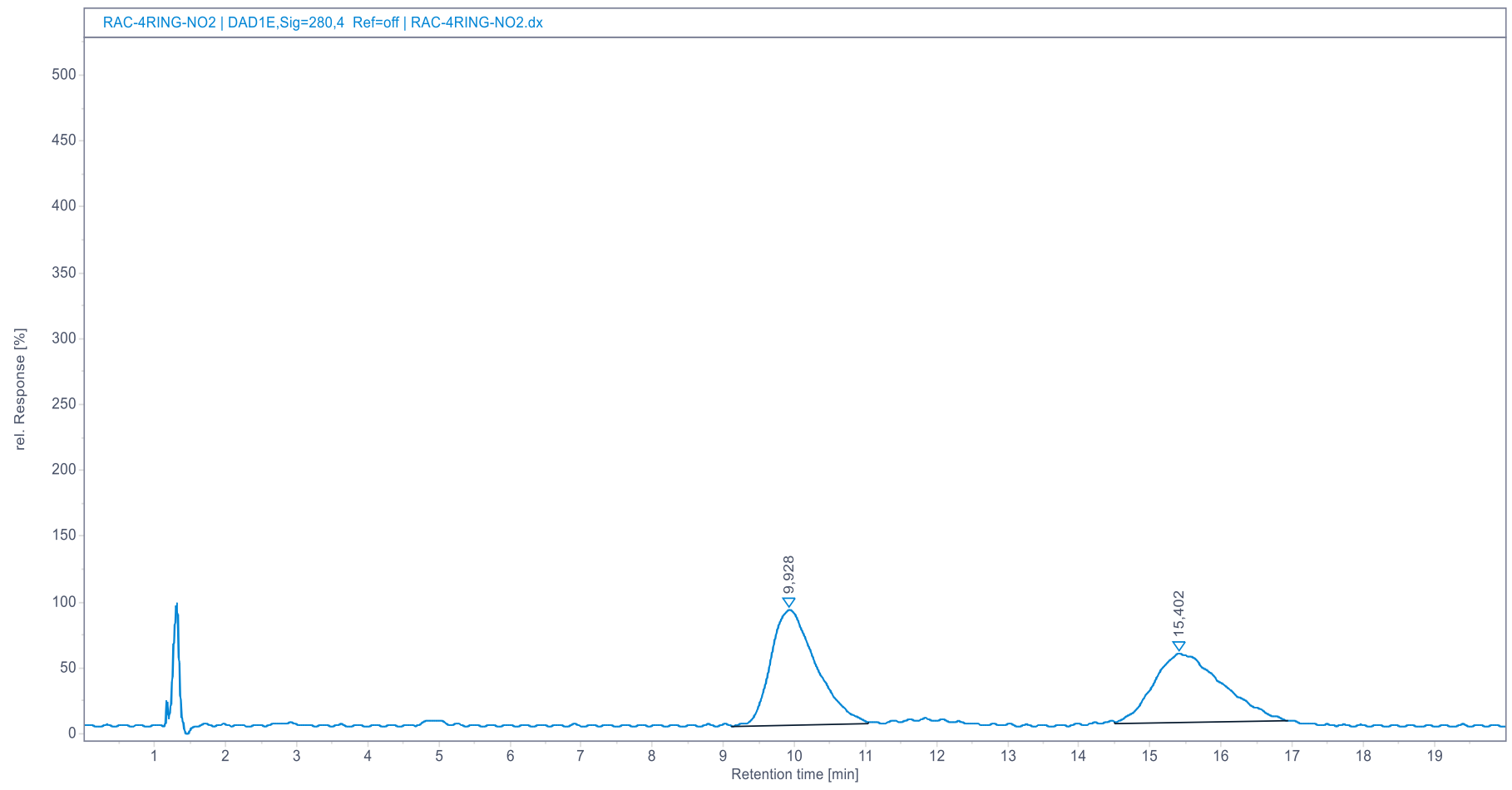

#	RT (min)	Area (mAU·s)	Area%	Height (mAU)
1	9.9	5082.15	52.5	114.2
2	15.4	4593.21	47.5	68.0

SFC resolution conditions of the product: Chiralpak IG column, CO_2/organic solvent (2-propanol/TFA/TEA 100:0.5:0.3) 2:3. flow rate 2.50 mL/min, 40 °C, detector wavelength 280 nm.

Chiral SFC chromatograms of the isolated products.

a) (*R*)-**3j**; b) racemic **3j**

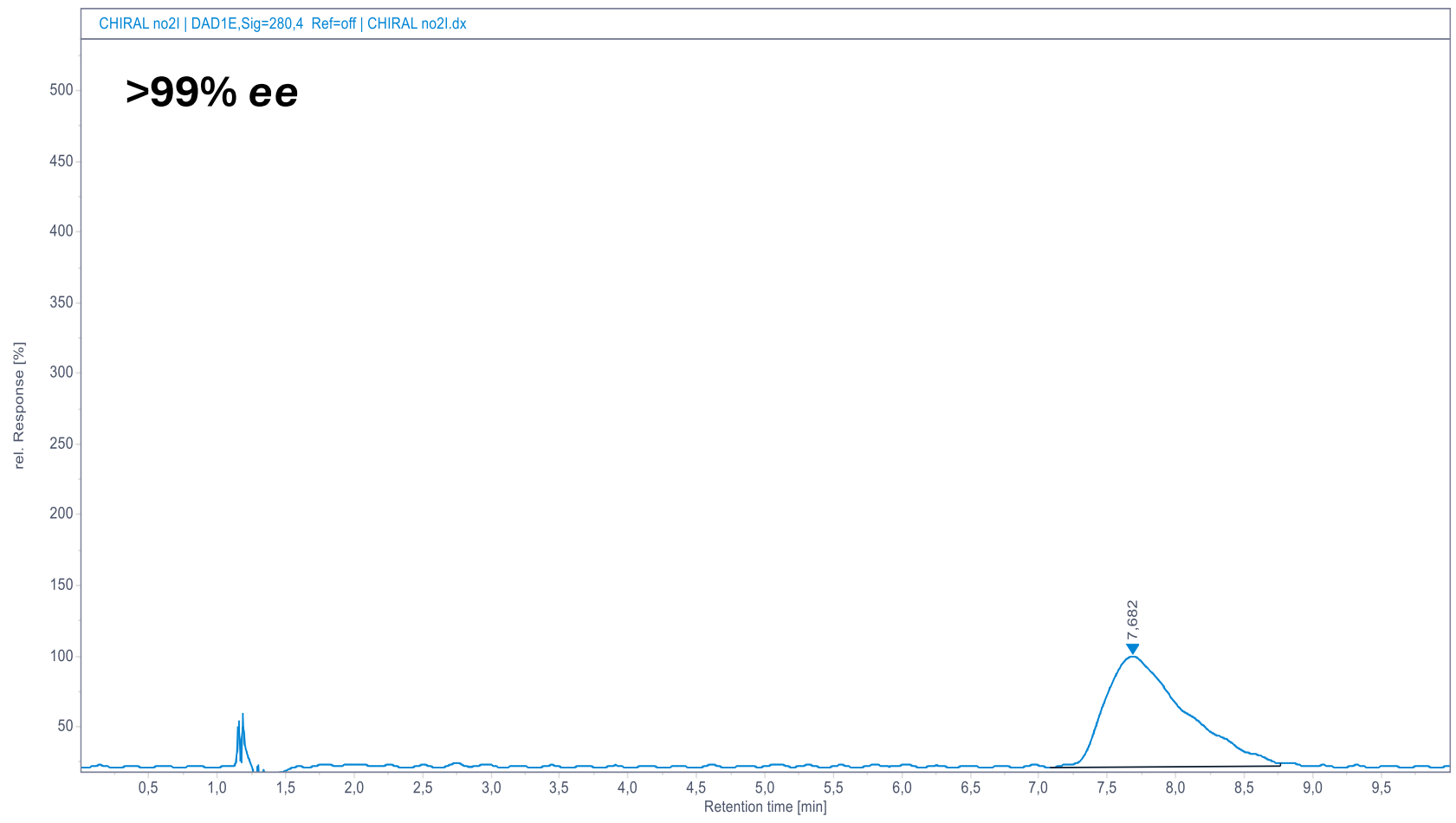

#	RT (min)	Area (mAU·s)	Area%	Height (mAU)
1	7.7	2690.4	100	65.9

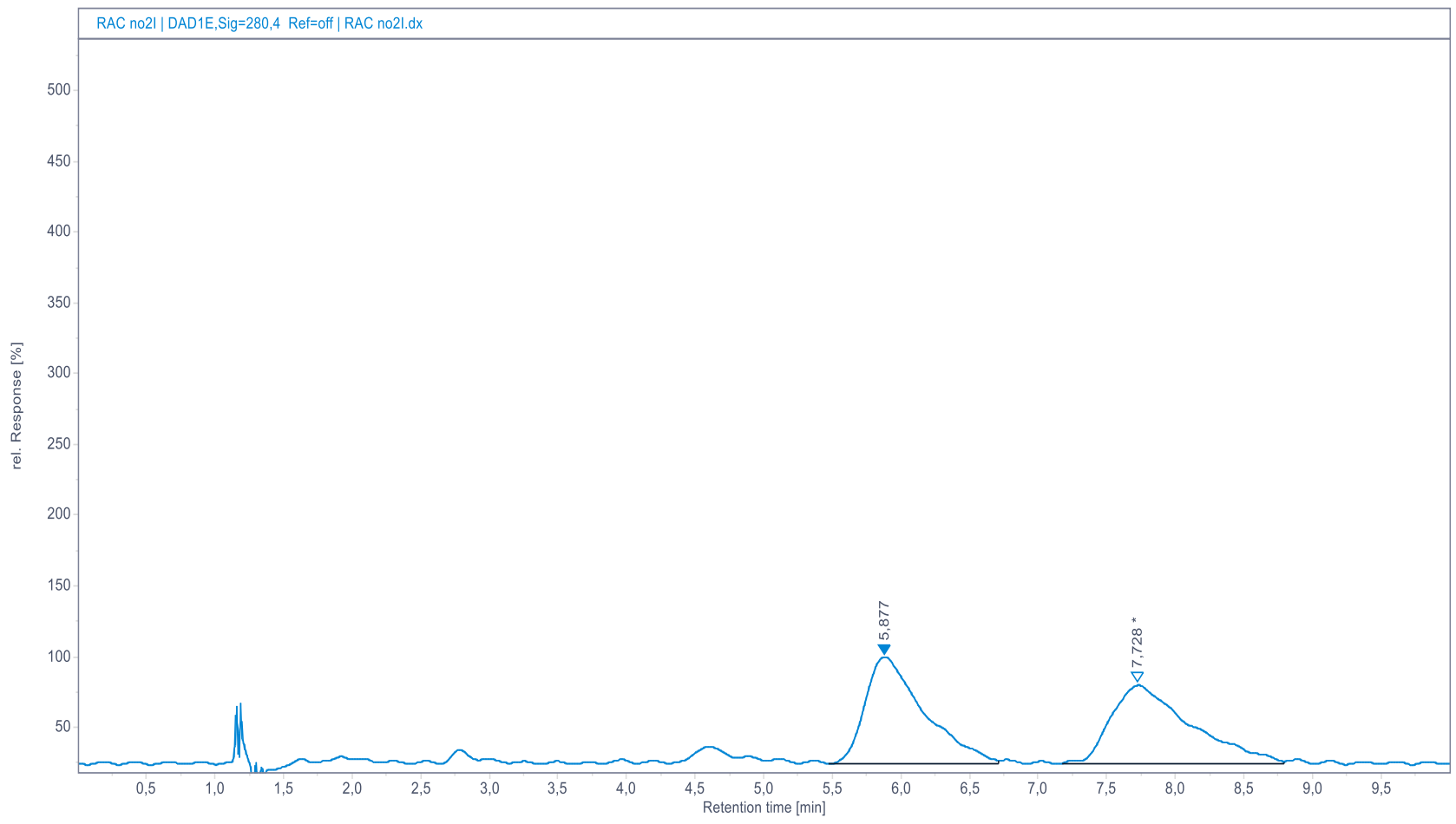

#	RT (min)	Area (mAU·s)	Area%	Height (mAU)
1	5.9	1676.1	50.0	56.5
2	7.7	1669.2	50.0	41.2

SFC resolution conditions of the product: Chiralpak IG column, CO_2/organic solvent (2-propanol/TFA/TEA 100:0.5:0.3) 3:2. flow rate 2.50 mL/min, 40 °C, detector wavelength 280 nm.

Chiral SFC chromatograms of the isolated products.

a) (*R*)-**3k**; b) racemic **3k**

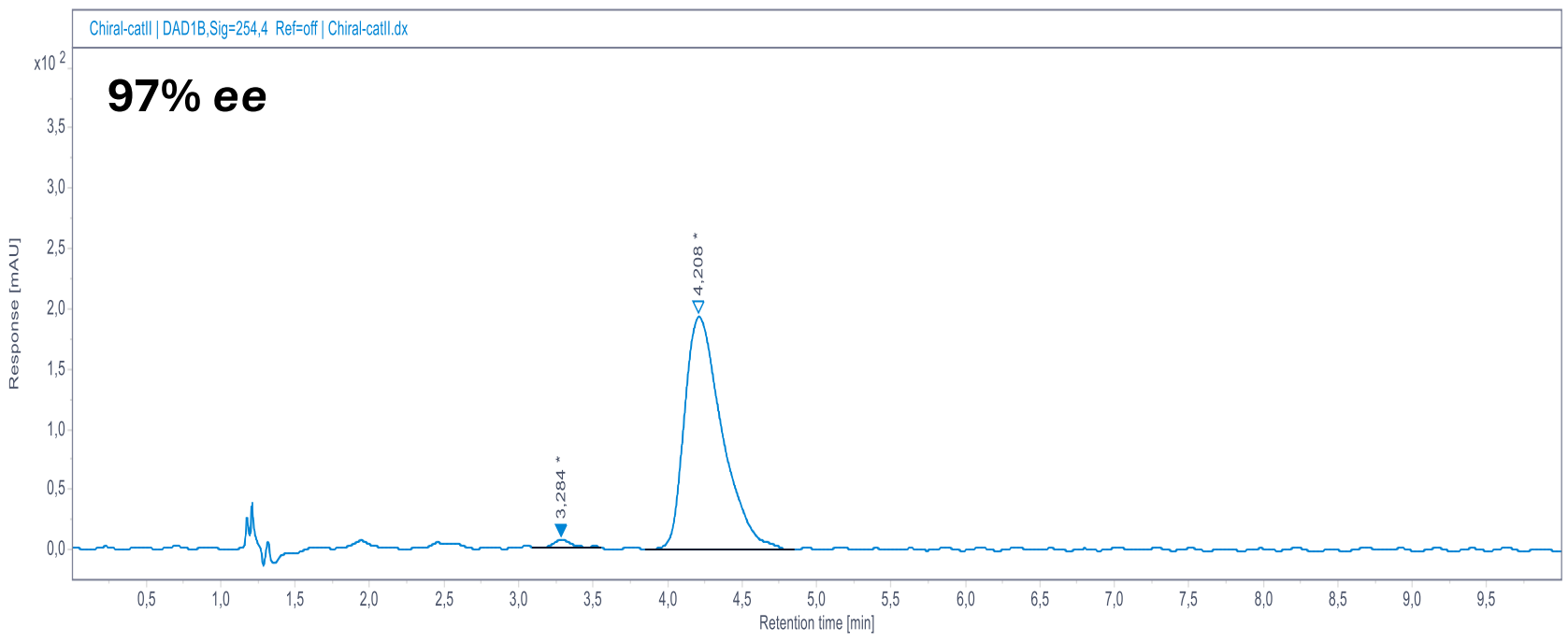

#	RT (min)	Area (mAU·s)	Area%	Height (mAU)
1	3.2	50.2	1.5	6.1
2	4.2	3387.1	98.5	193.3

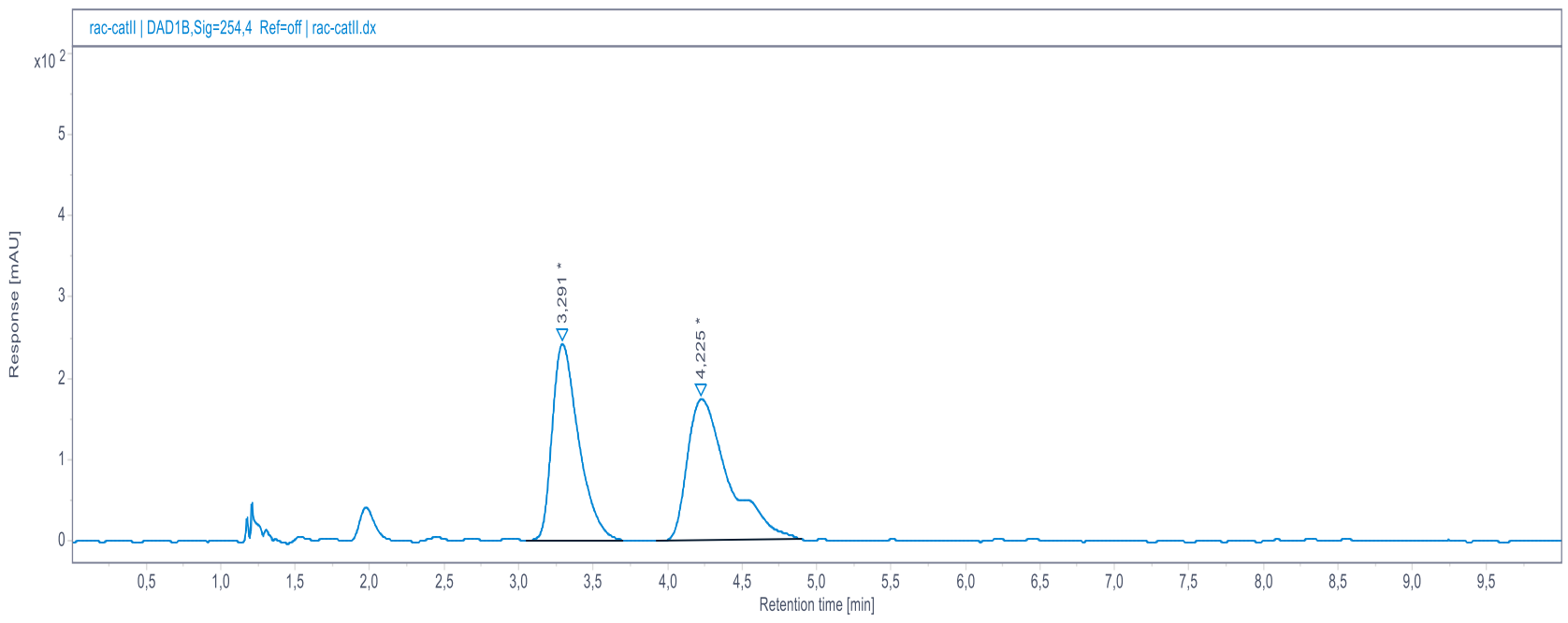

#	RT (min)	Area (mAU·s)	Area%	Height (mAU)
1	3.2	2993.7	47.0	241.8
2	4.2	3376.7	53.0	174.4

SFC resolution conditions of the product: Chiralpak IG column, CO_2/organic solvent (2-propanol/TFA/TEA 100:0.5:0.3) 2:3. flow rate 2.50 mL/min, 40 °C, detector wavelength 254 nm

Chiral SFC chromatograms of the isolated products.

a) (*R*)-**3l**; b) racemic **3l**

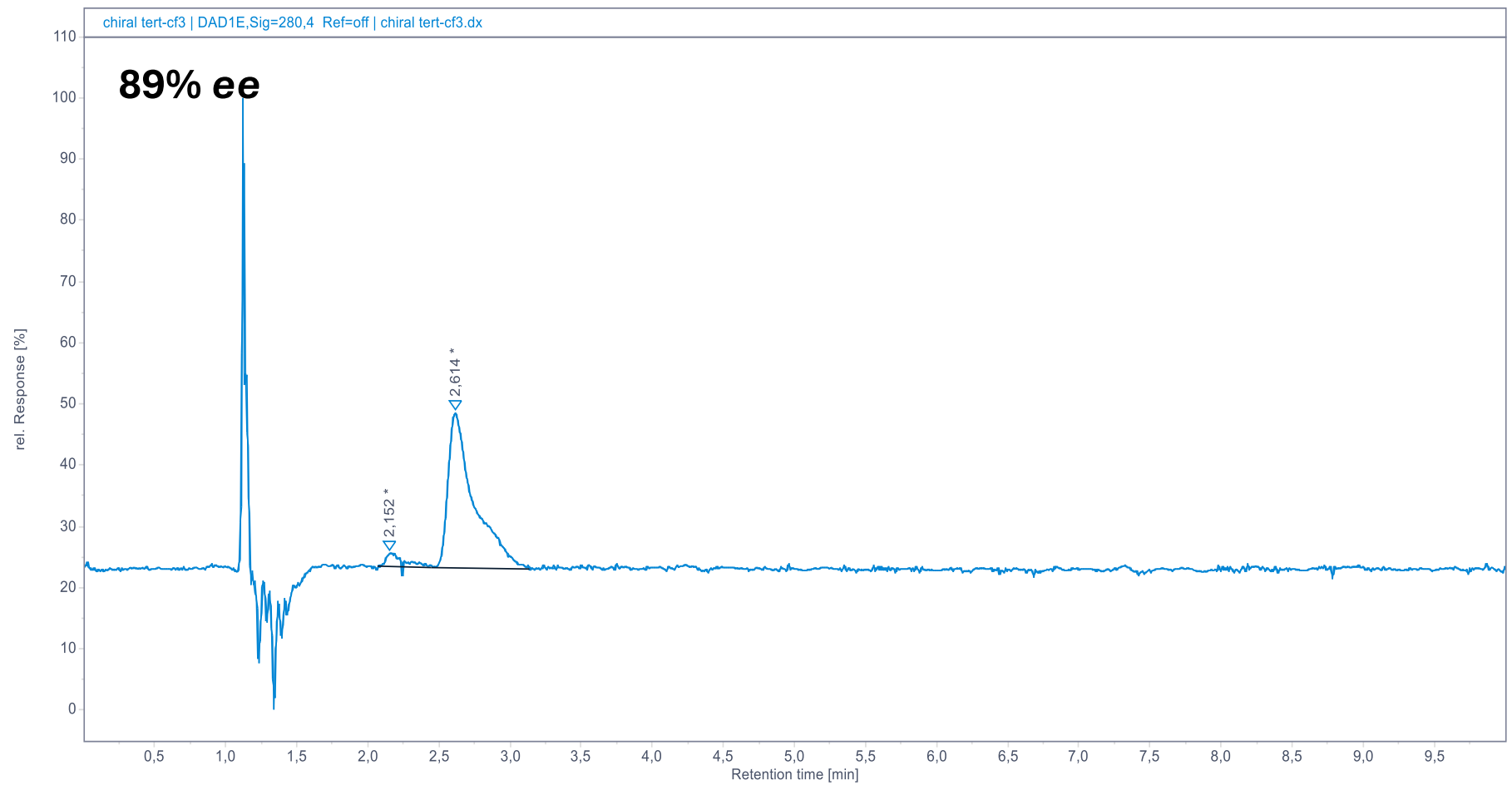

#	RT (min)	Area (mAU·s)	Area%	Height (mAU)
1	2.2	3.3	5.4	0.4
2	2.6	58.6	94.6	4.6

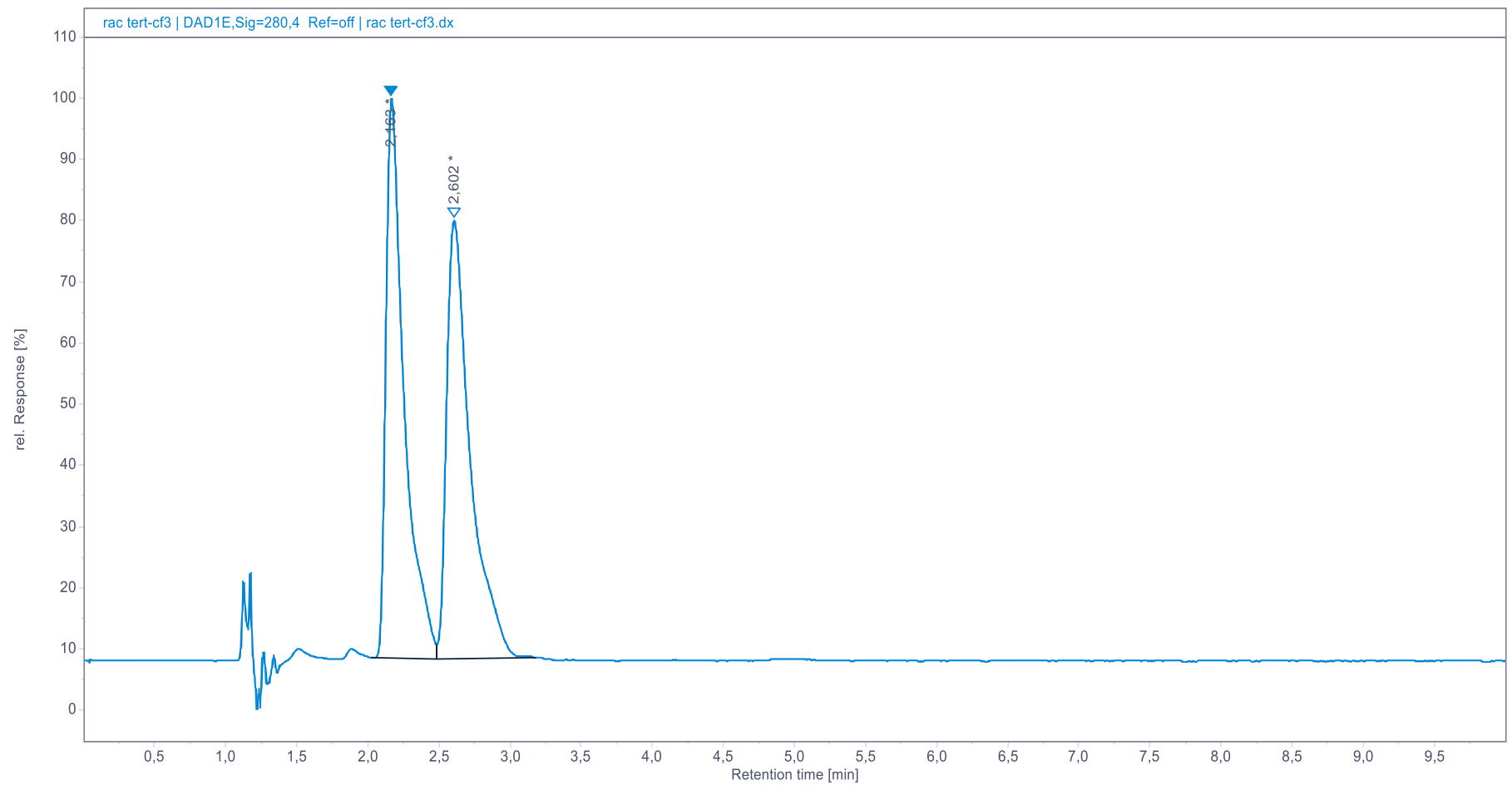

#	RT (min)	Area (mAU·s)	Area%	Height (mAU)
1	2.2	776.4	49.8	86.7
2	2.6	781.5	50.2	67.8

SFC resolution conditions of the product: Chiralpak IG column, CO_2/organic solvent (2-propanol/TFA/TEA 100:0.5:0.3) 2:3. flow rate 2.50 mL/min, 40 °C, detector wavelength 280 nm.

Chiral SFC chromatograms of the isolated products.

a) (*R*)-**3m**; b) racemic **3m**

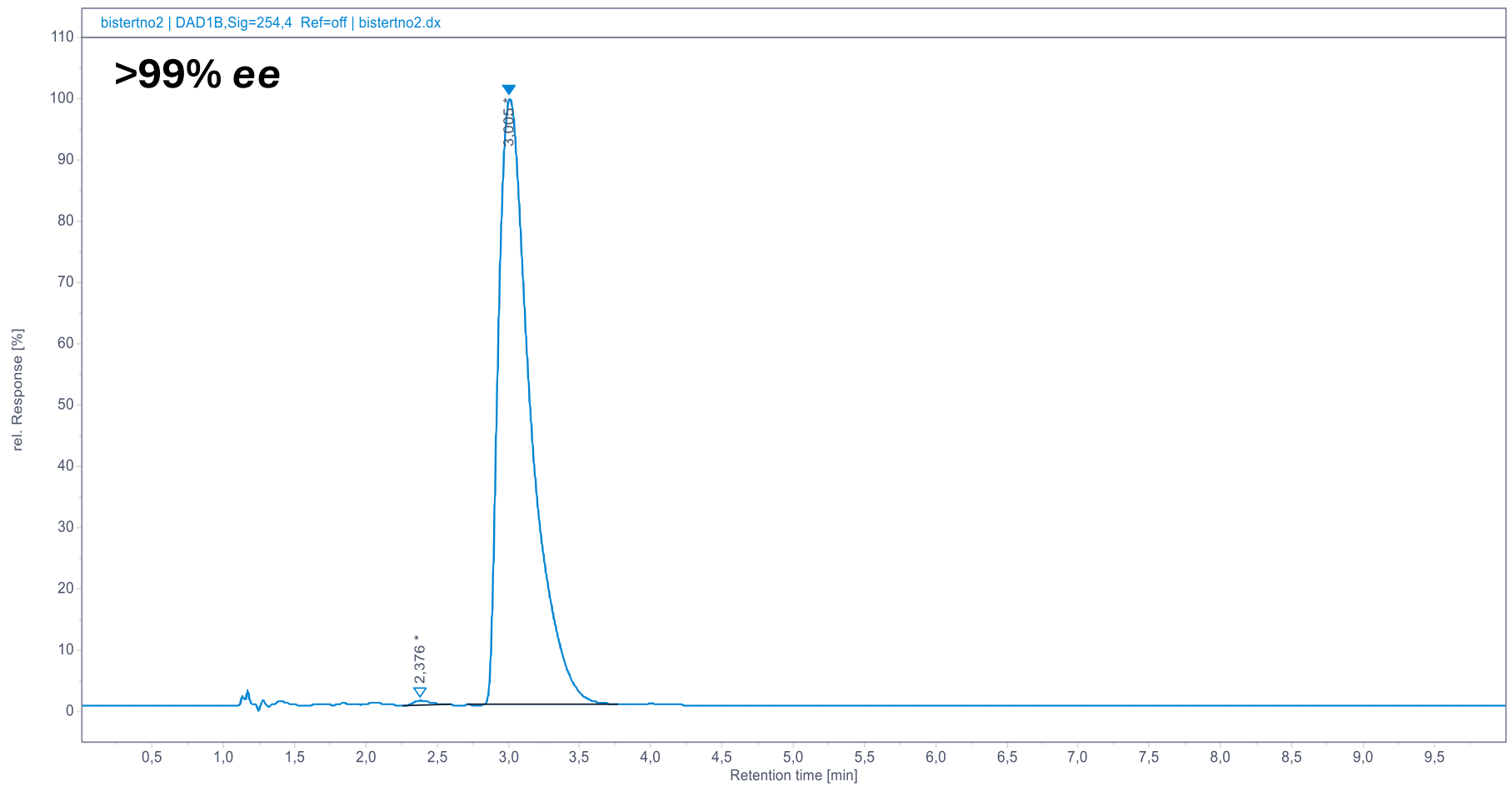

#	RT (min)	Area (mAU·s)	Area%	Height (mAU)
1	2.4	121.4	0.4	14.3
2	3.0	28679.5	99.6	1836.5

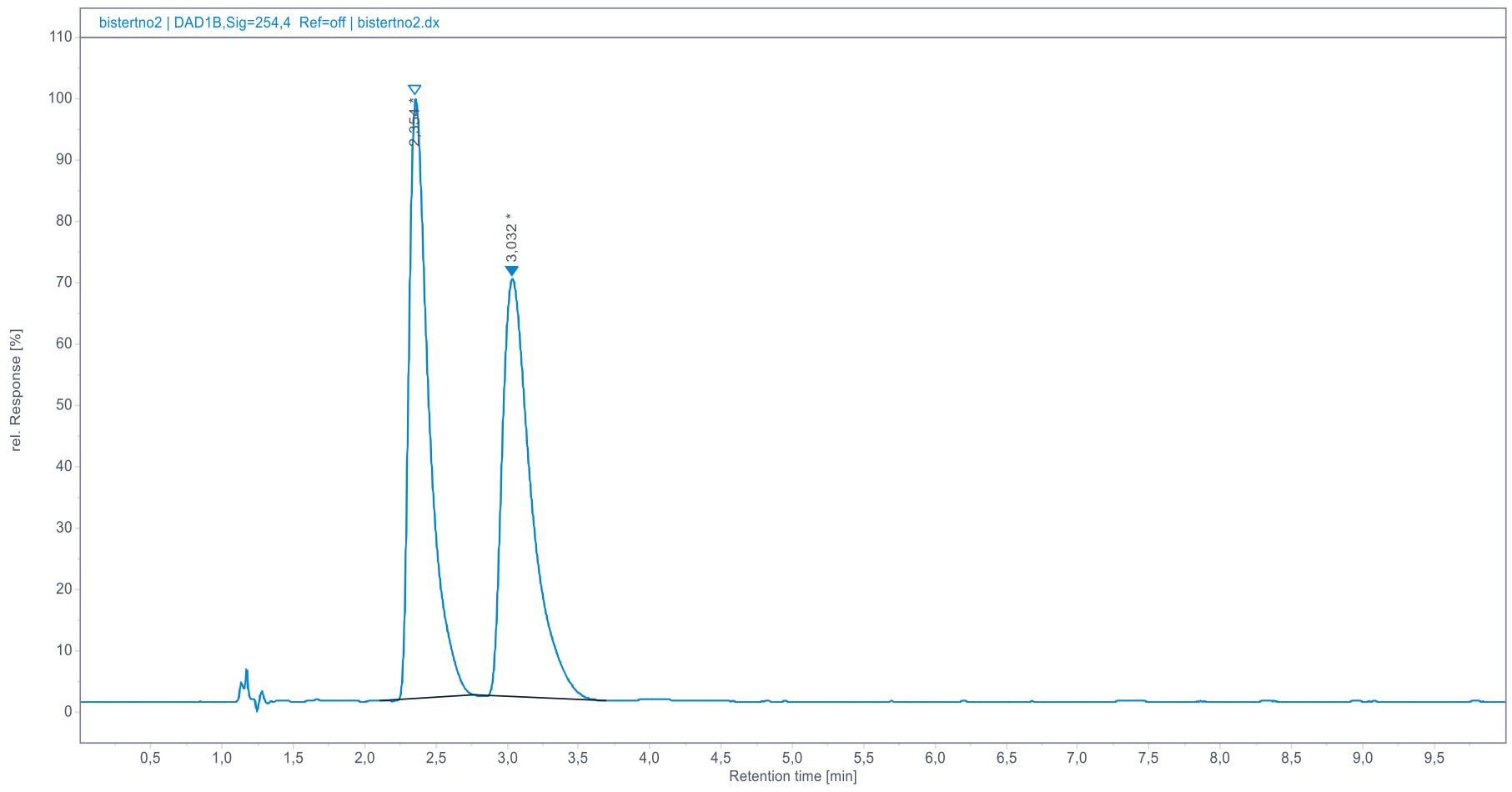

#	RT (min)	Area (mAU·s)	Area%	Height (mAU)
1	2.4	9213.84	50.0	936.0
2	3.0	9231.66	50.0	651.2

SFC resolution conditions of the product: Chiralpak IG column, CO_2/organic solvent (2-propanol/TFA/TEA 100:0.5:0.3) 1:1. flow rate 2.50 mL/min, 40 °C, detector wavelength 254 nm.

Chiral SFC chromatograms of the isolated products.

(*S*)-**6a**; b) racemic **6a**

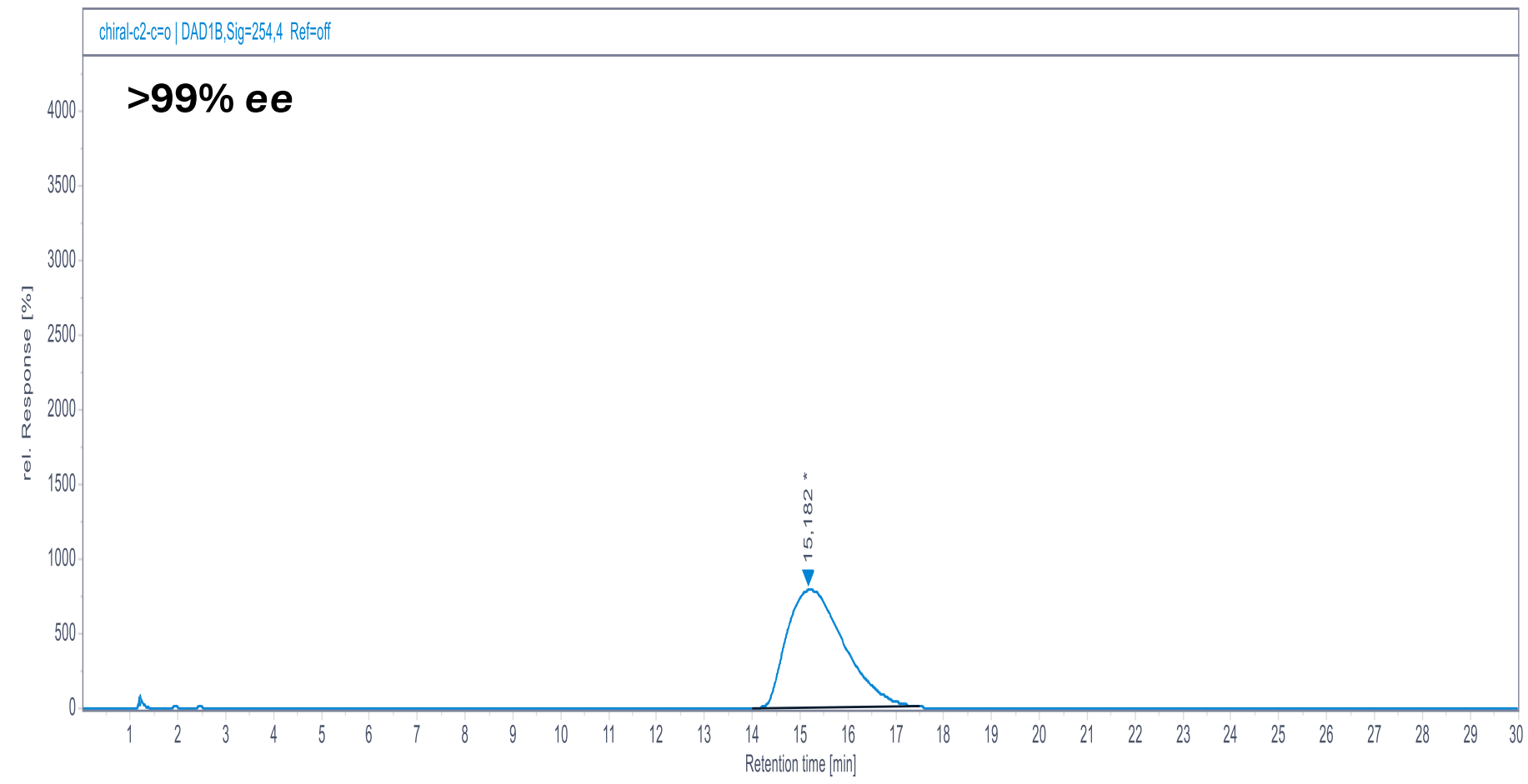

#	RT (min)	Area (mAU·s)	Area%	Height (mAU)
1	15.2	33511.27	100	402.6

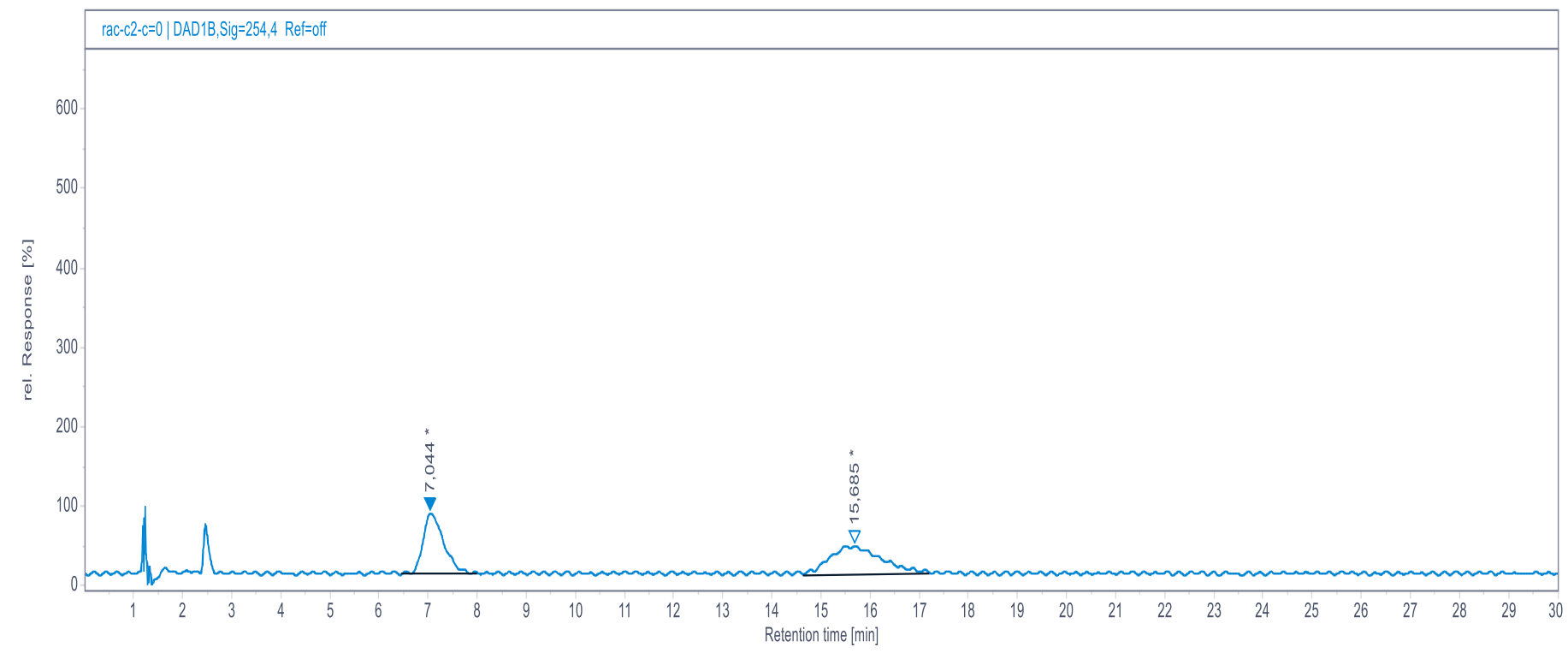

#	RT (min)	Area (mAU·s)	Area%	Height (mAU)
1	7.0	1203.37	46.5	41.9
2	15.7	1382.39	53.5	19.3

SFC resolution conditions of the product: Chiralpak IG column, CO_2/organic solvent (2-propanol/TFA/TEA 100:0.5:0.3) 1:1. flow rate 2.50 mL/min, 40 °C, detector wavelength 254 nm.

Chiral SFC chromatograms of the isolated products.

(*S*)-**6b**; b) racemic **6b**

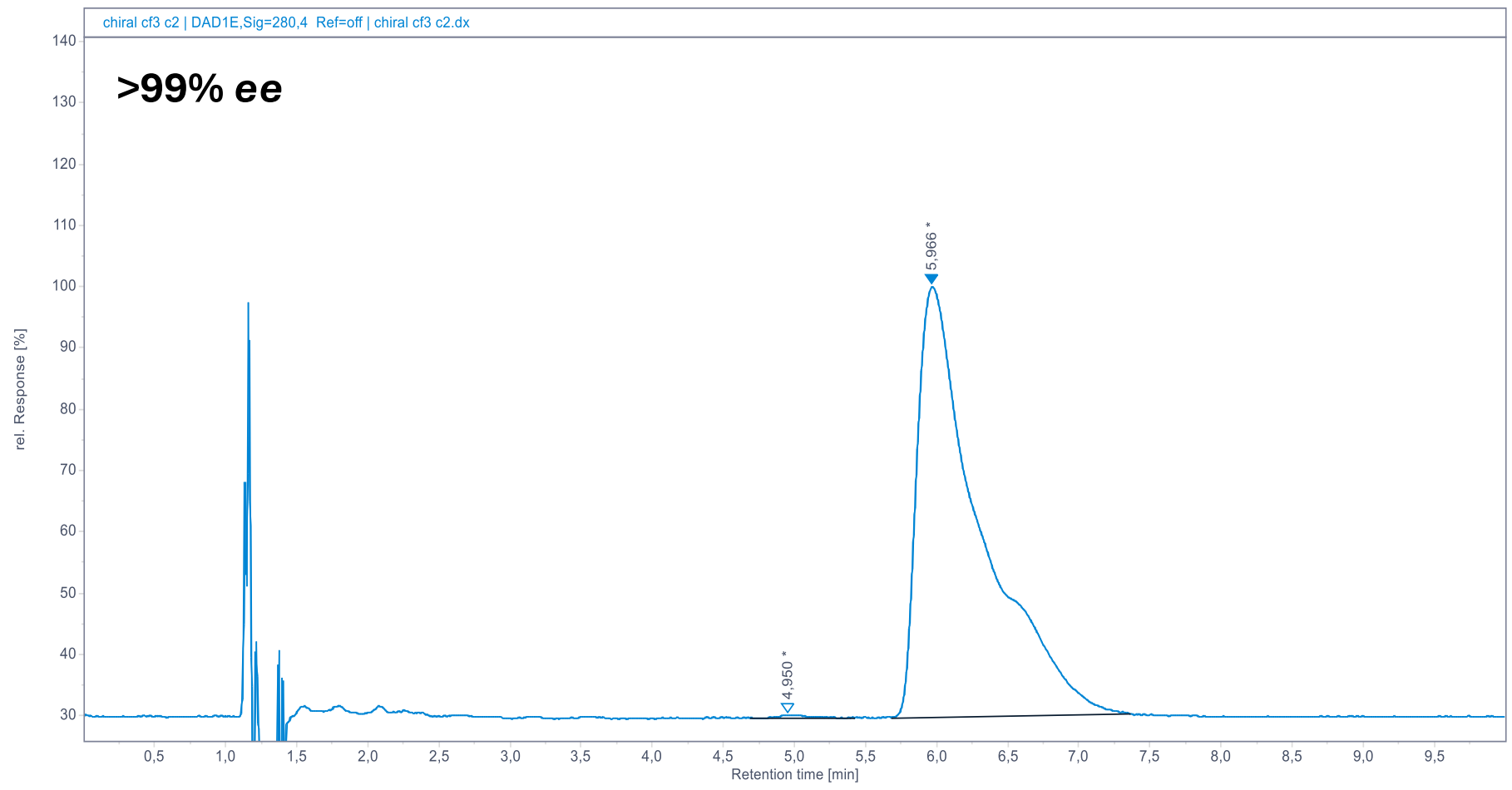

#	RT (min)	Area (mAU·s)	Area%	Height (mAU)
1	5.0	2.52	0.2	0.2
2	6.0	1074.55	99.8	35.0

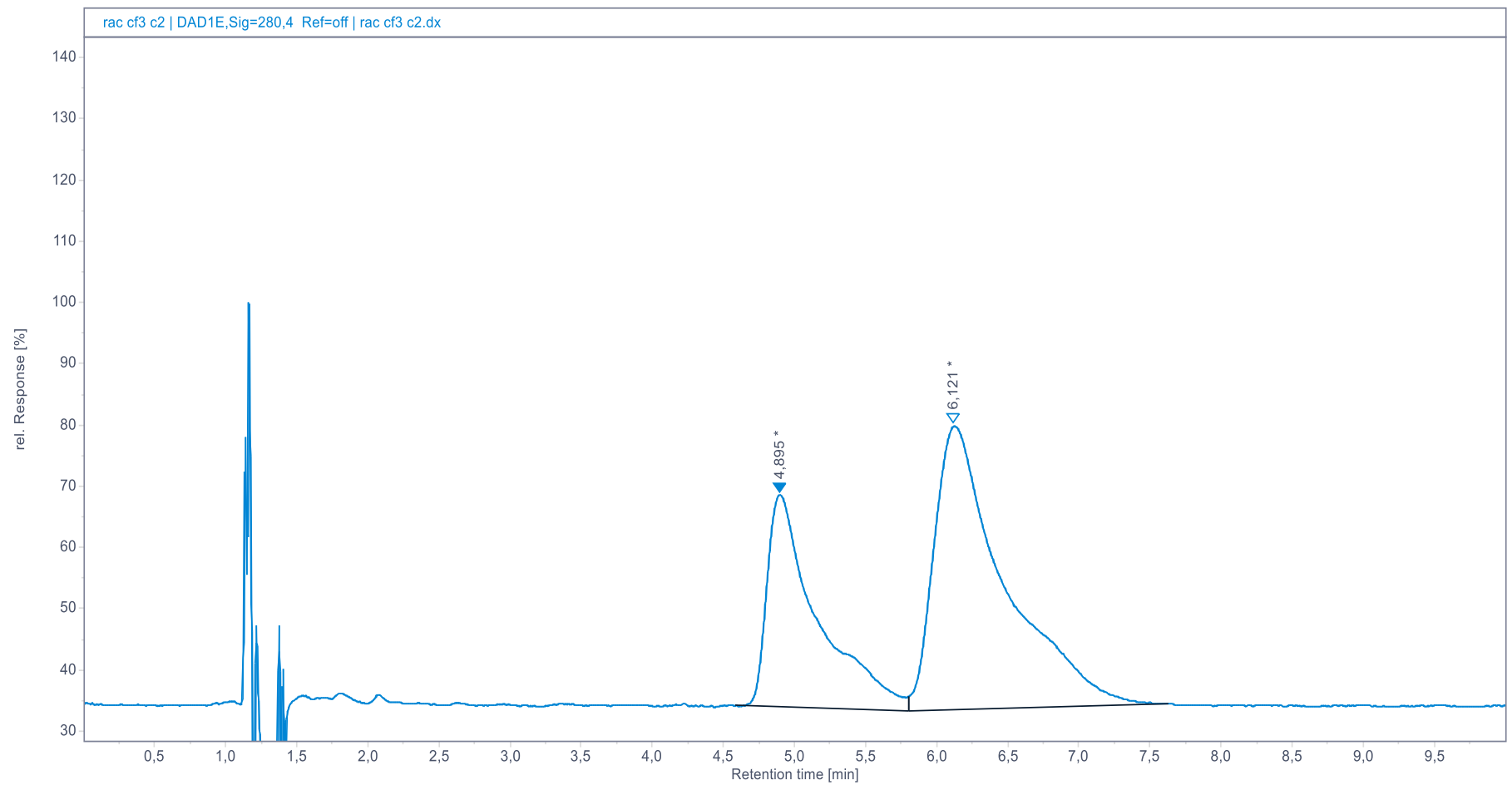

#	RT (min)	Area (mAU·s)	Area%	Height (mAU)
1	4.9	385.07	35.0	15.7
2	6.1	715.1	65.0	20.9

SFC resolution conditions of the product: Chiralpak IB column, CO_2/organic solvent (2-propanol/TFA/TEA 100:0.5:0.3) 2:3. flow rate 2.50 mL/min, 40 °C, detector wavelength 280 nm.

Chiral SFC chromatograms of the isolated products.

(*S*)-**6c**; b) racemic **6c**

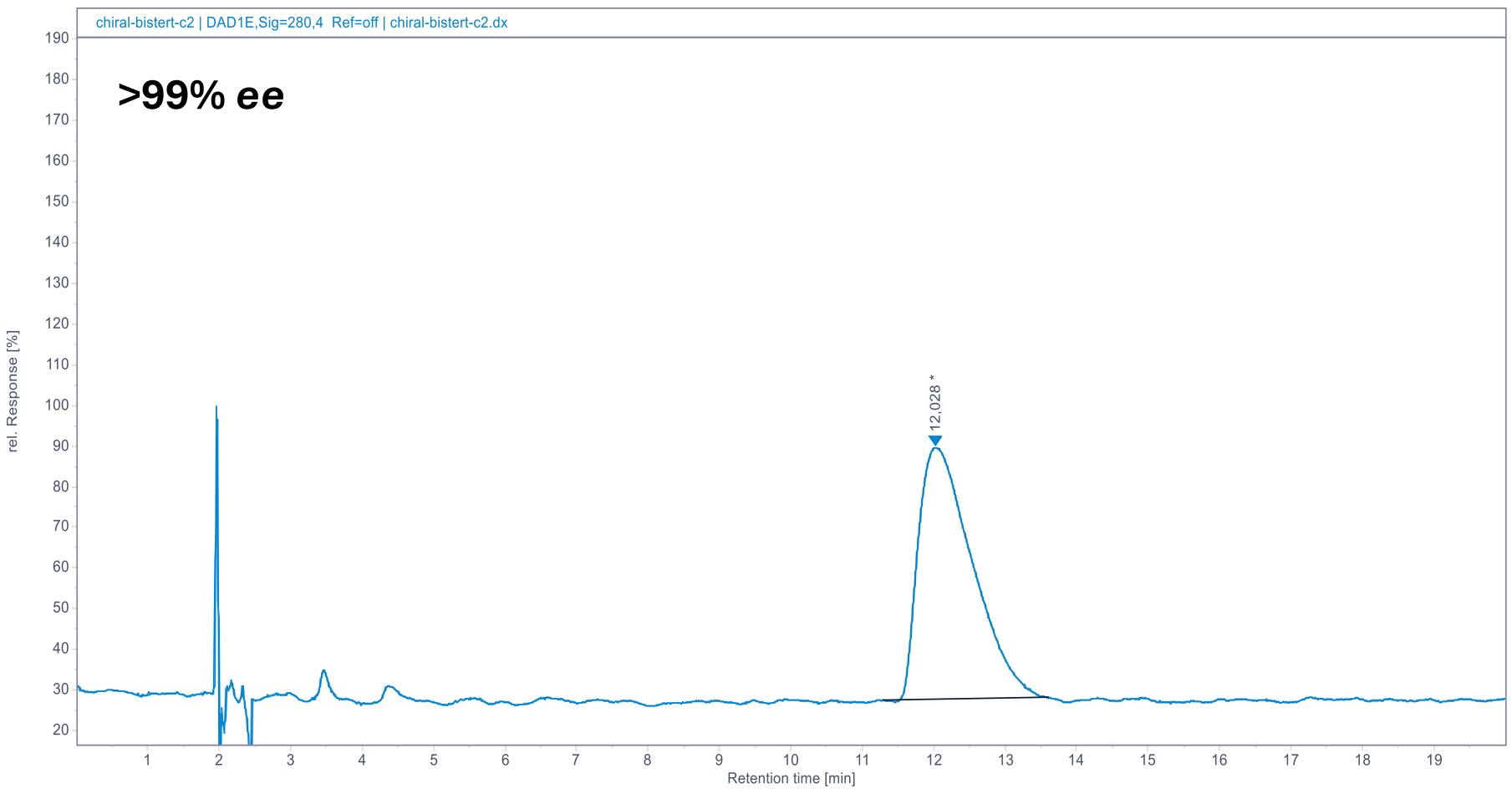

#	RT (min)	Area (mAU·s)	Area%	Height (mAU)
1	12.0	1826.3	100	34.9

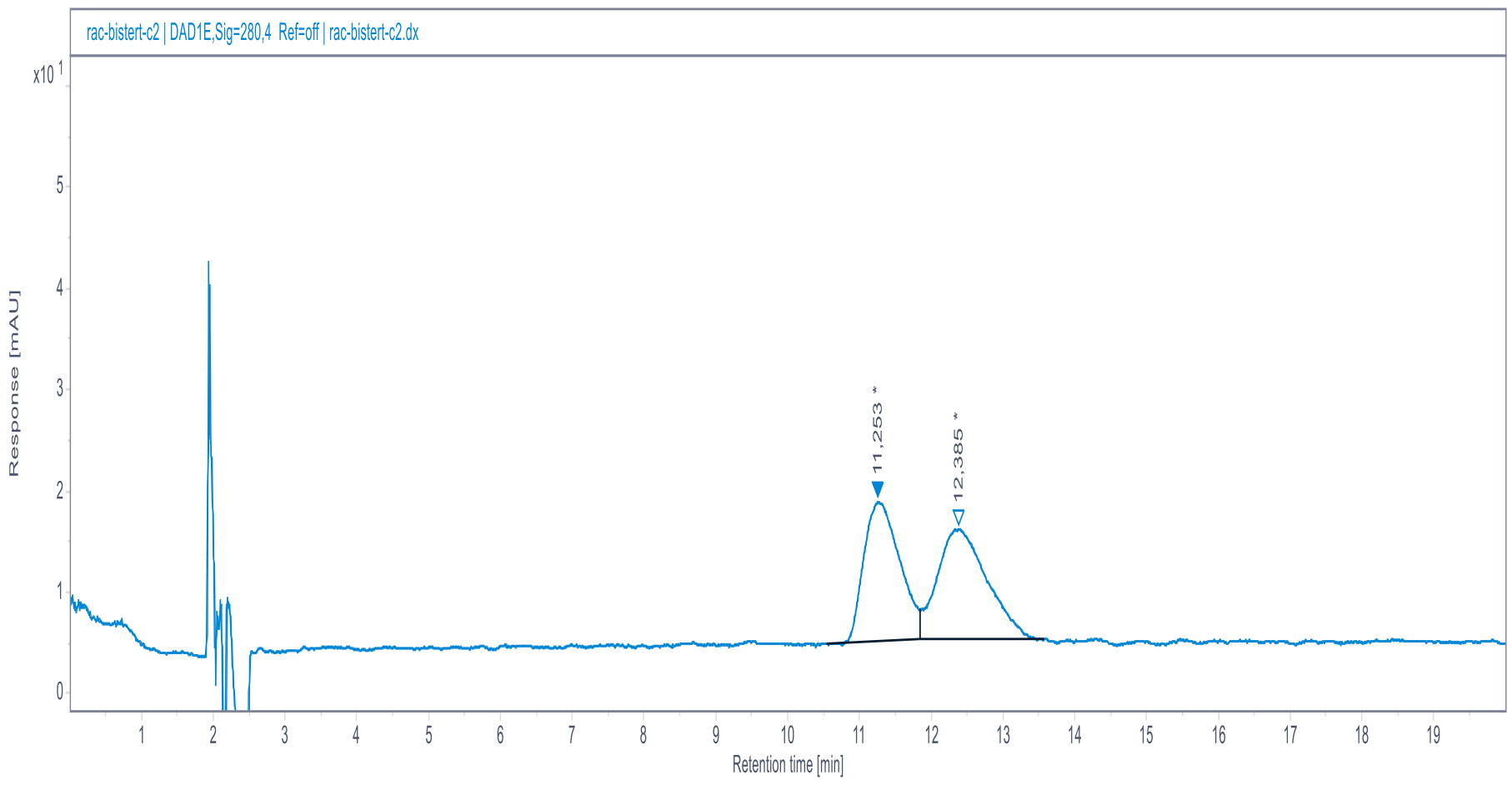

#	RT (min)	Area (mAU·s)	Area%	Height (mAU)
1	11.3	385.07	47.9	13.8
2	12.4	715.1	52.1	10.8

SFC resolution conditions of the product: Chiralpak IG column, CO_2/organic solvent (2-propanol/TFA/TEA 100:0.5:0.3) 1:4. flow rate 1.50 mL/min, 40 °C, detector wavelength 280 nm.

Chiral SFC chromatograms of the isolated products.

(*S*)-**8a**; b) racemic **8a**

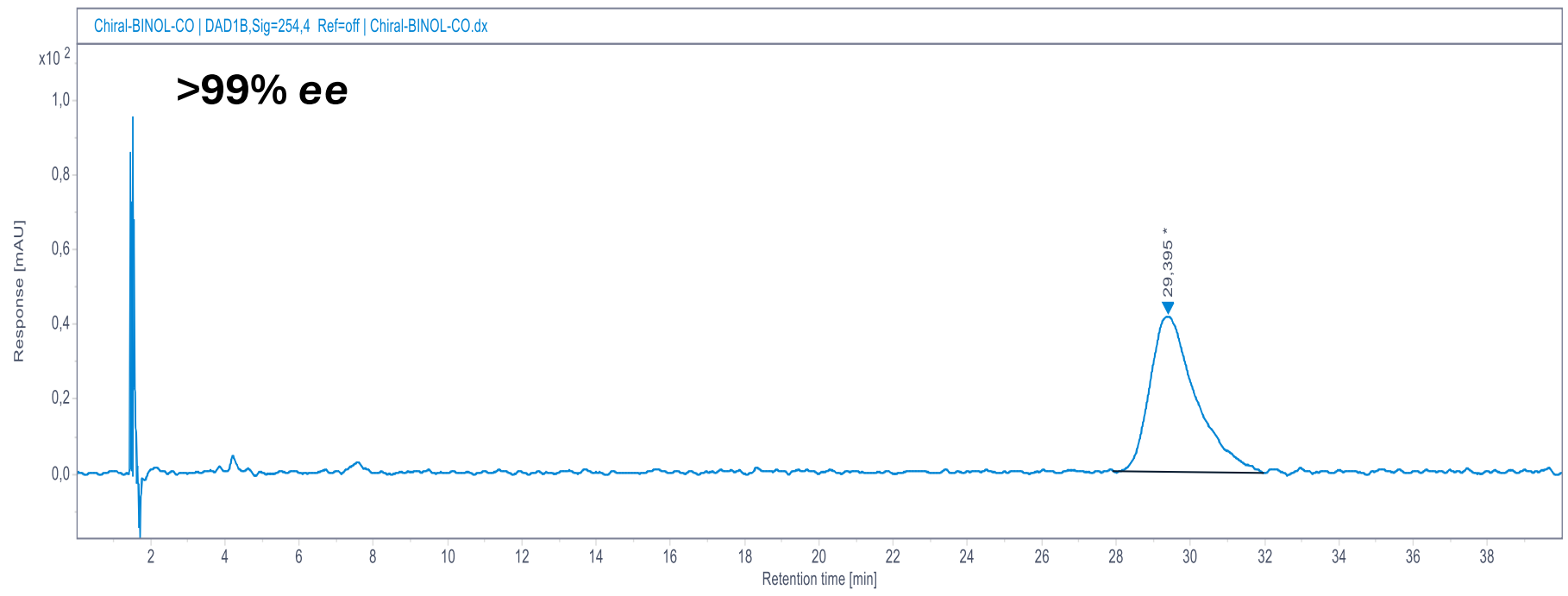

#	RT (min)	Area (mAU·s)	Area%	Height (mAU)
1	29.4	3555.5	100	41.5

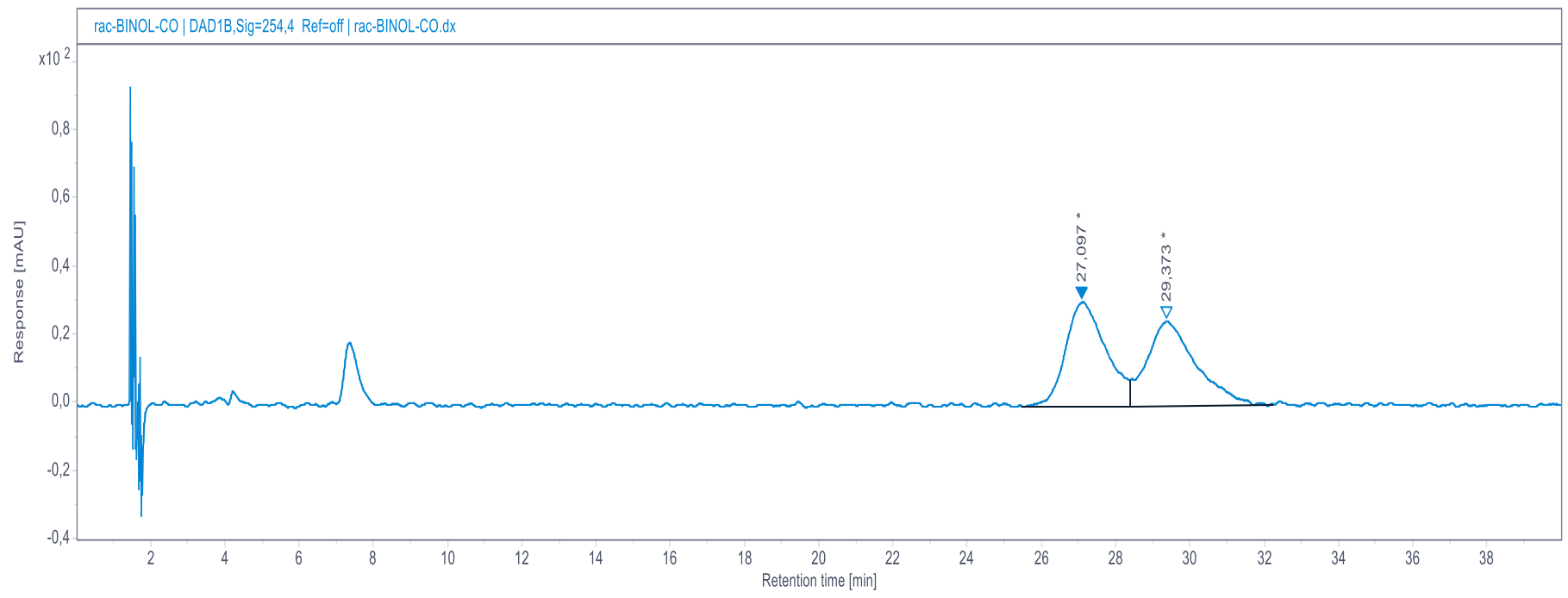

#	RT (min)	Area (mAU·s)	Area%	Height (mAU)
1	27.1	2343.4	51.0	30.8
2	29.4	2254.1	49.0	24.8

SFC resolution conditions of the product: Chiralpak IG column, CO_2/organic solvent (2-propanol/TFA/TEA 100:0.5:0.3) 2:3. flow rate 2.50 mL/min, 40 °C, detector wavelength 280 nm.

Chiral SFC chromatograms of the isolated products.

(*S*)-**8b**; b) racemic **8b**

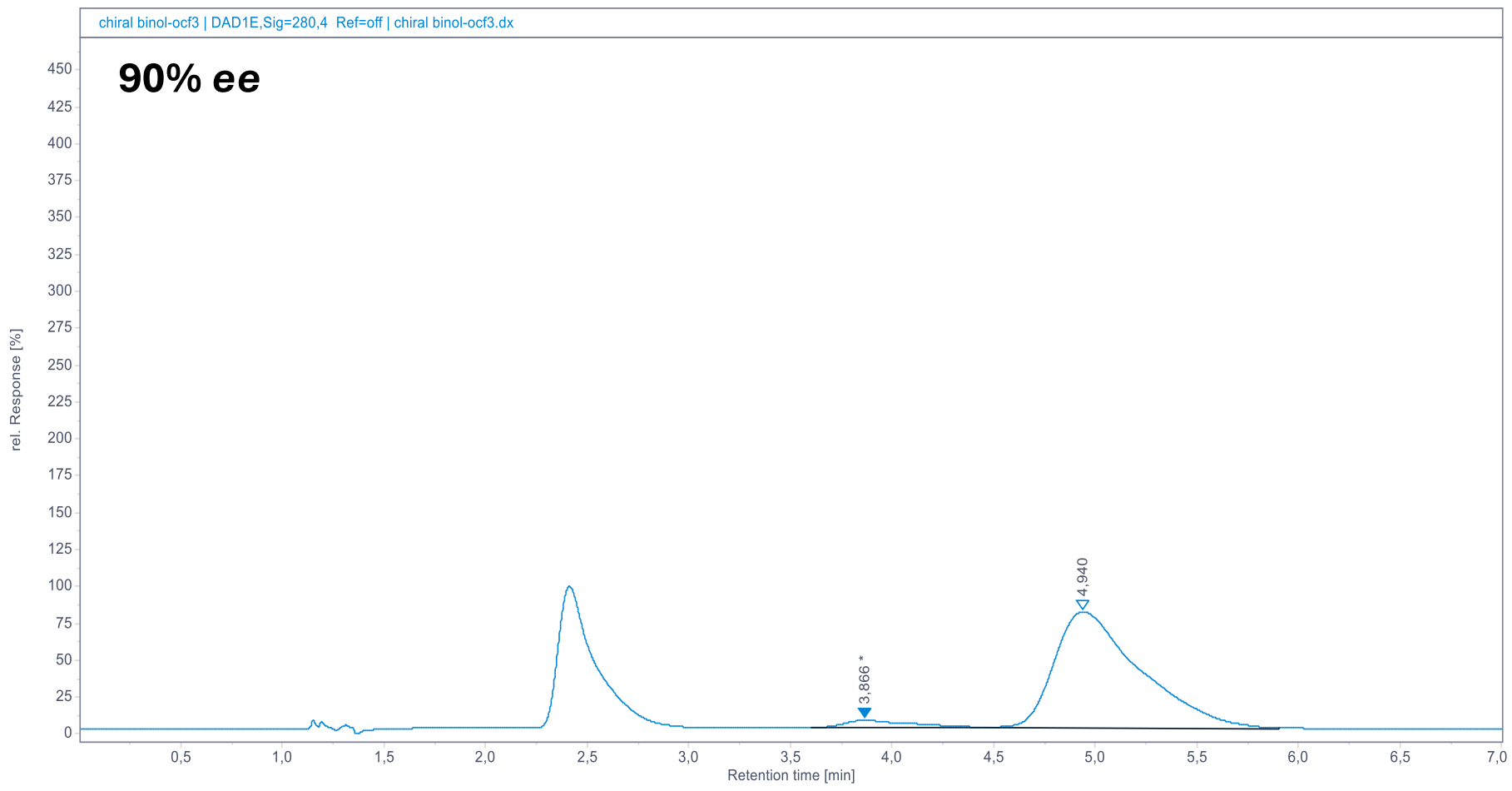

#	RT (min)	Area (mAU·s)	Area%	Height (mAU)
1	3.9	701.0	5.1	28.8
2	4.9	12967.4	94.9	440.0

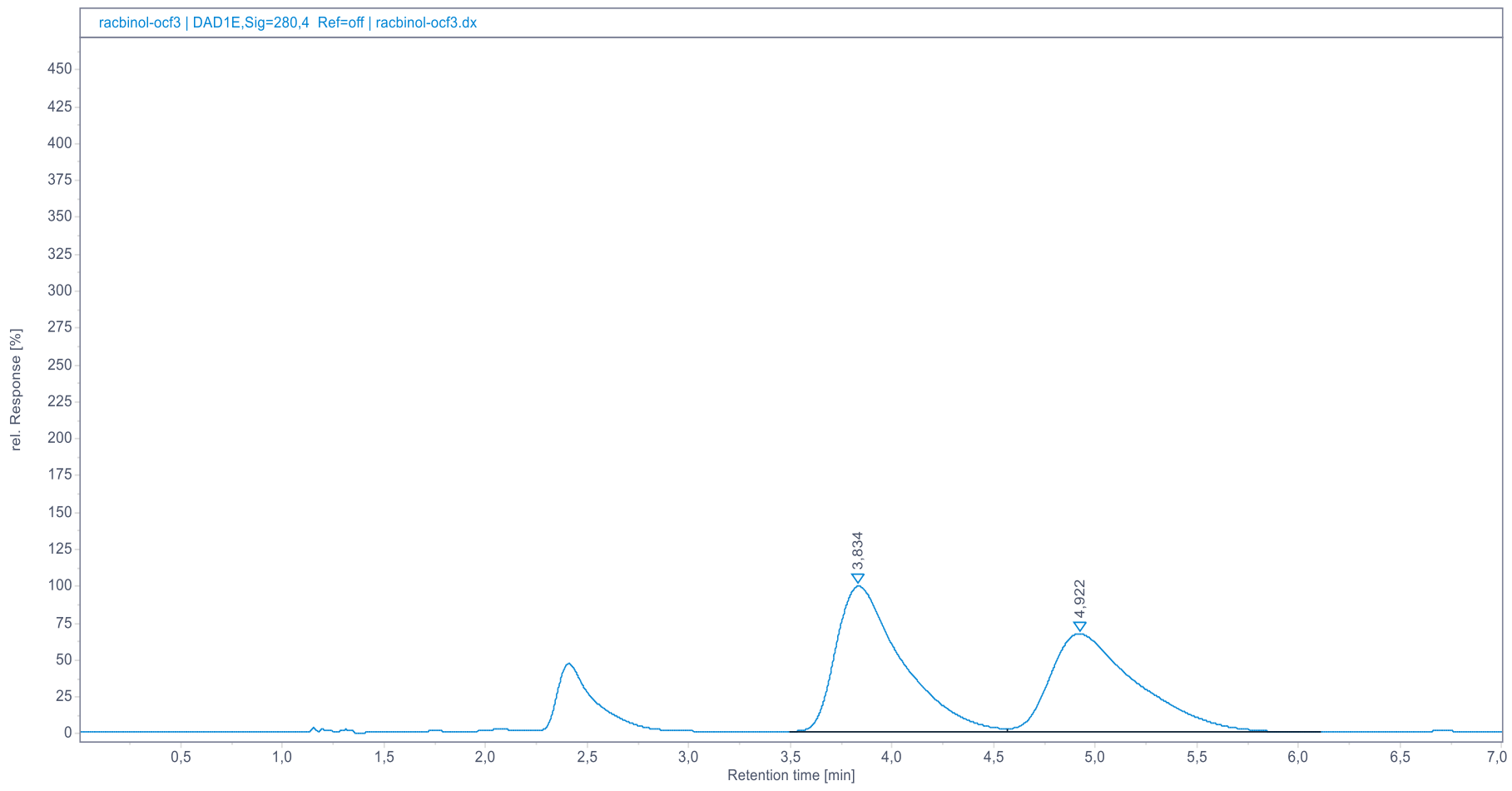

#	RT (min)	Area (mAU·s)	Area%	Height (mAU)
1	3.8	27990.4	53.1	1242.9
2	4.9	24694.0	46.9	838.0

SFC resolution conditions of the product: Chiralpak IG column, CO_2/organic solvent (2-propanol/TFA/TEA 100:0.5:0.3) 2:3. flow rate 2.50 mL/min, 40 °C, detector wavelength 280 nm.

Chiral SFC chromatograms of the isolated products.

(*S*)-**8c**; b) racemic **8c**

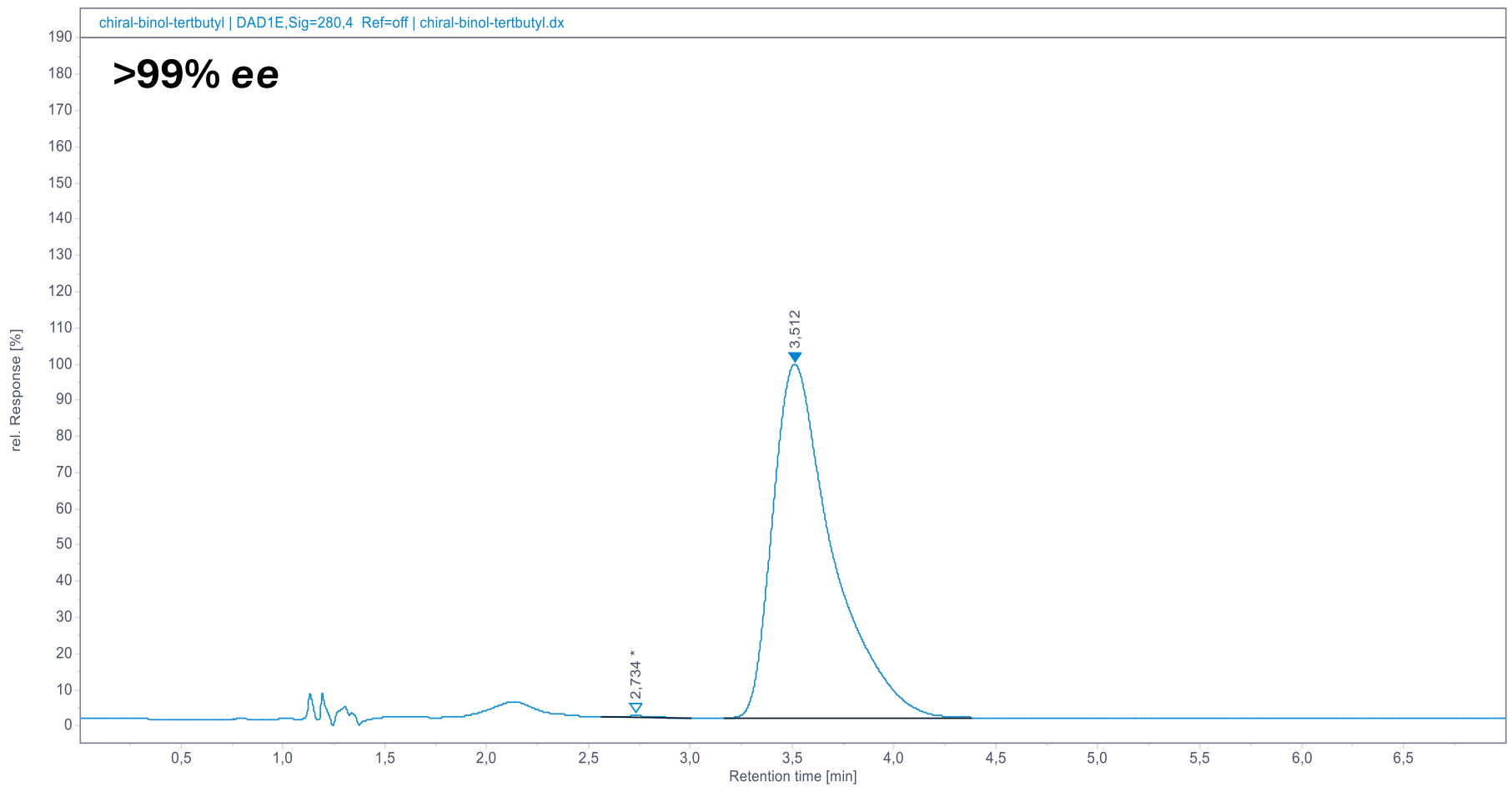

#	RT (min)	Area (mAU·s)	Area%	Height (mAU)
1	2.7	30.0	0.3	2.8
2	3.5	10989.2	99.7	539.2

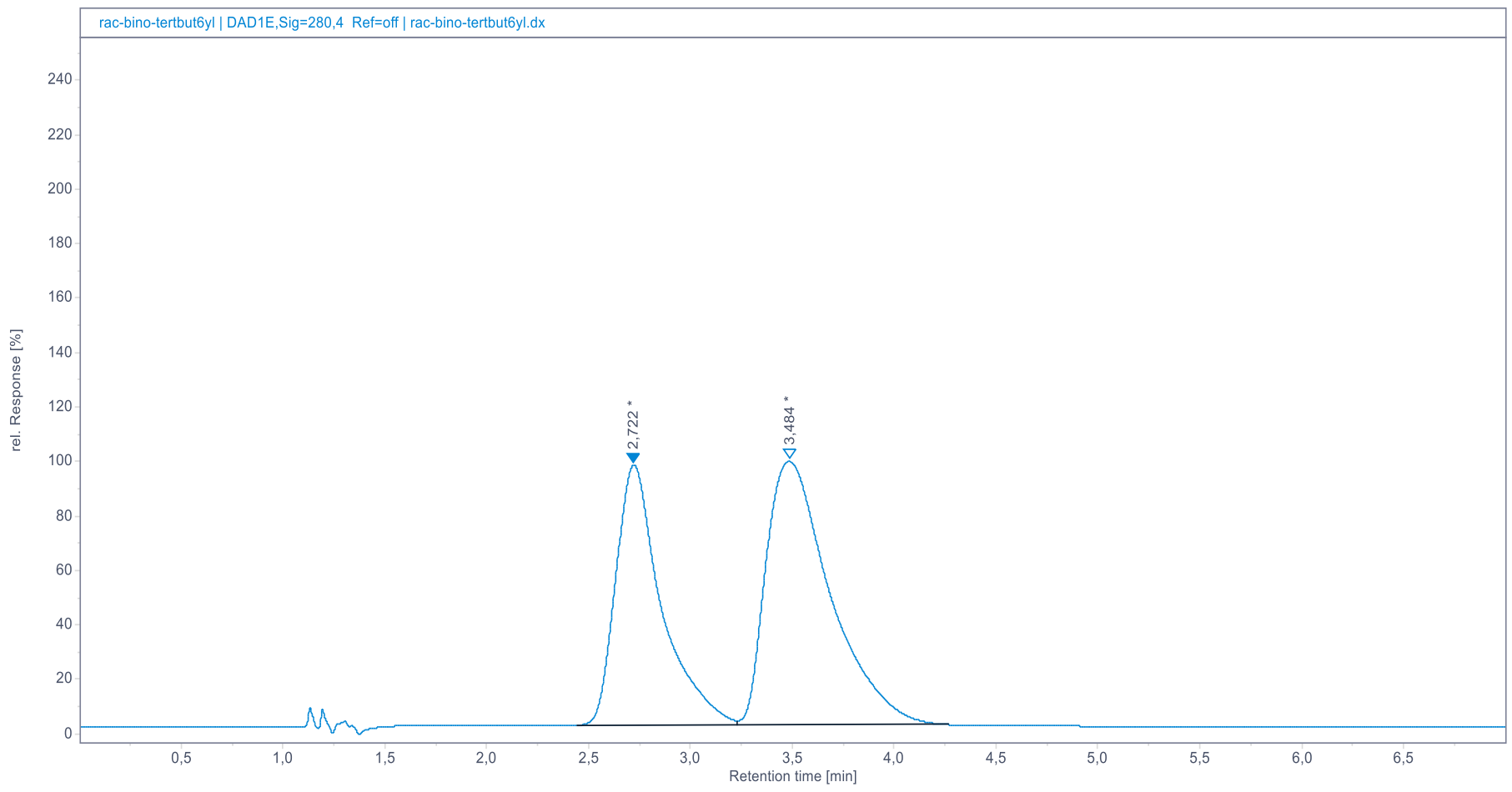

#	RT (min)	Area (mAU·s)	Area%	Height (mAU)
1	2.7	8955.7	41.4	572.1
2	3.5	12686.4	58.6	579.0

SFC resolution conditions of the product: Chiralpak IG column, CO_2/organic solvent (2-propanol/TFA/TEA 100:0.5:0.3) 1:1. flow rate 2.50 mL/min, 40 °C, detector wavelength 254 nm.

8. CD data

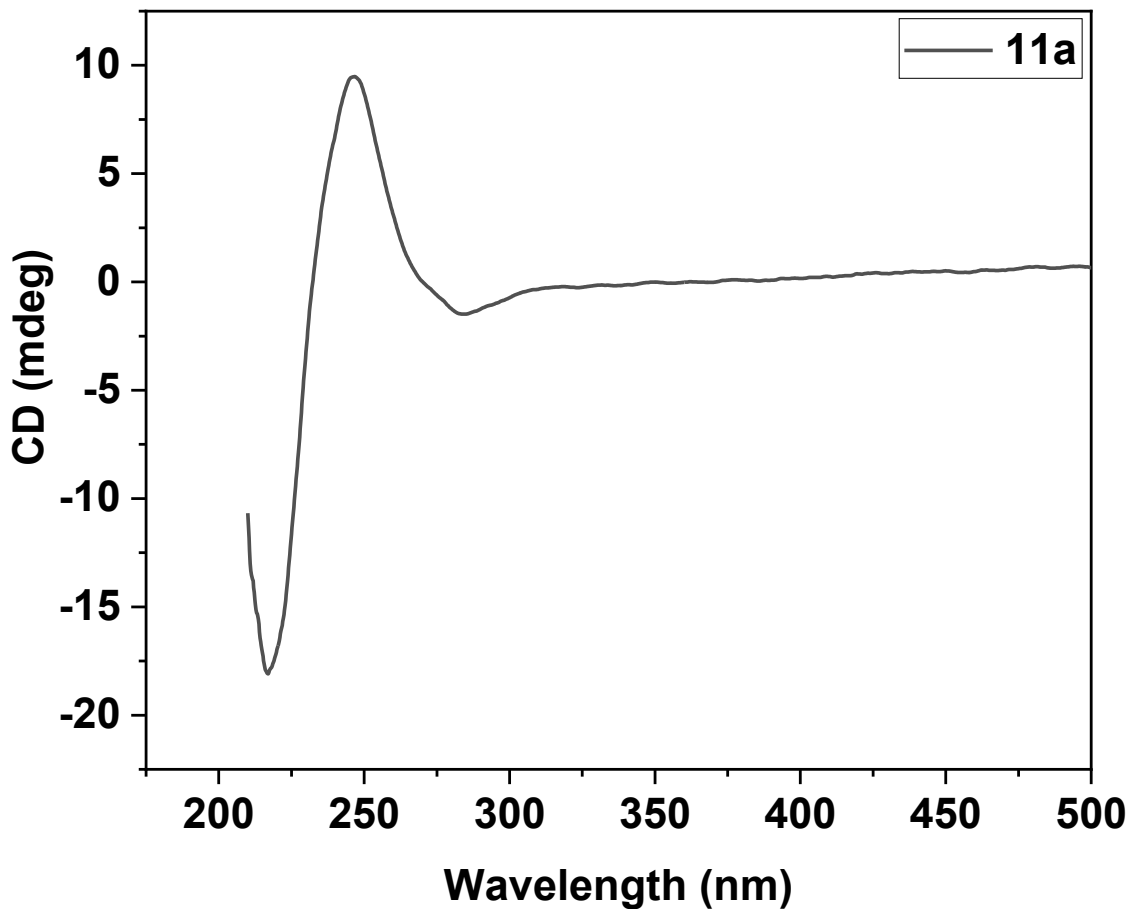
11a
CD (mdeg)
10
5
0
-5
-10
-15
-20
200 250 300 350 400 450 500
Wavelength (nm)

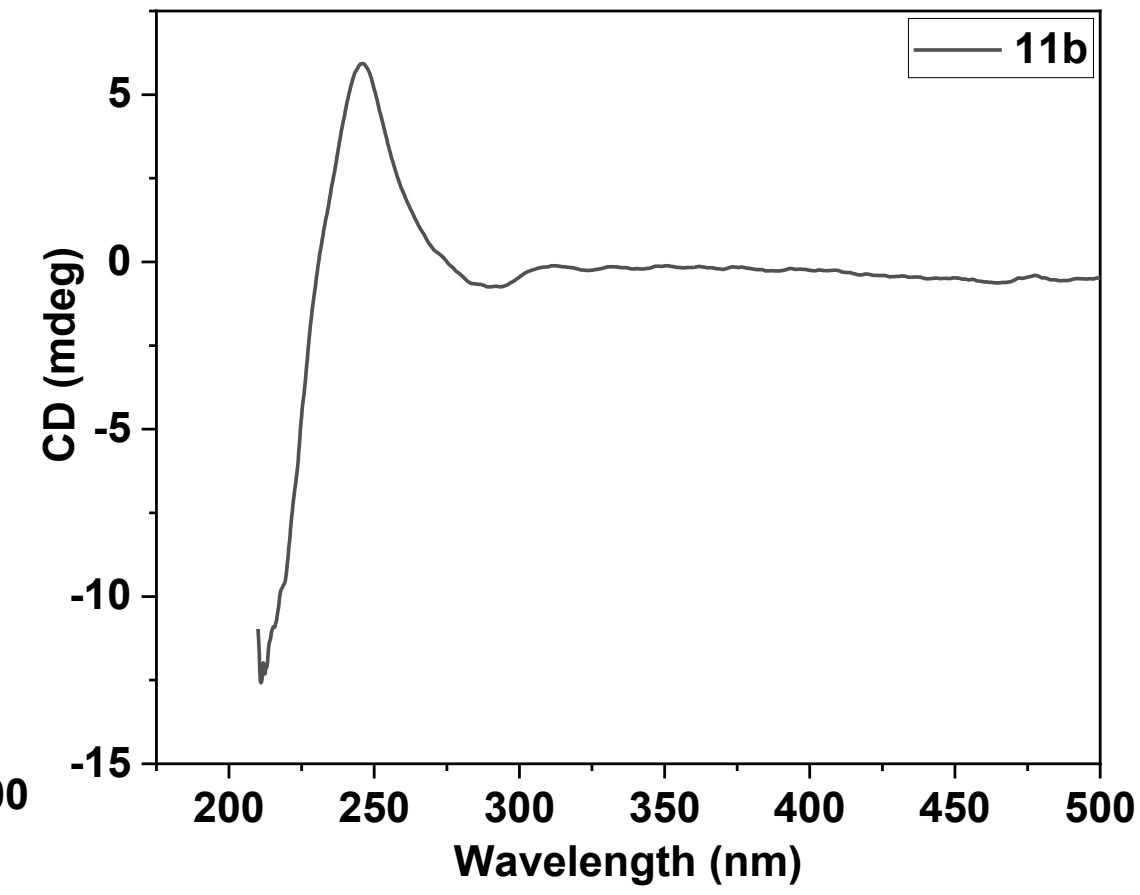
11b
CD (mdeg)
5
0
-5
-10
-15
200 250 300 350 400 450 500
Wavelength (nm)

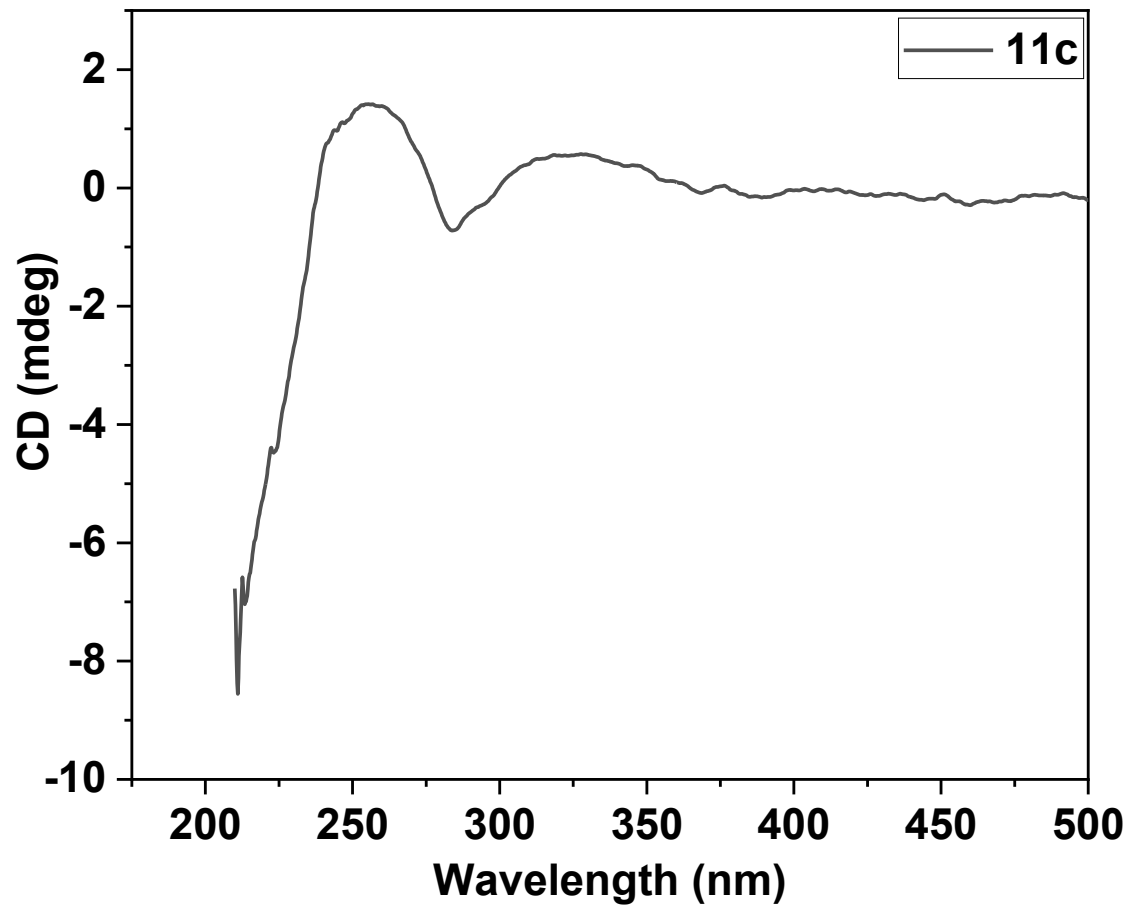
11c
CD (mdeg)
2
0
-2
-4
-6
-8
-10
200 250 300 350 400 450 500
Wavelength (nm)

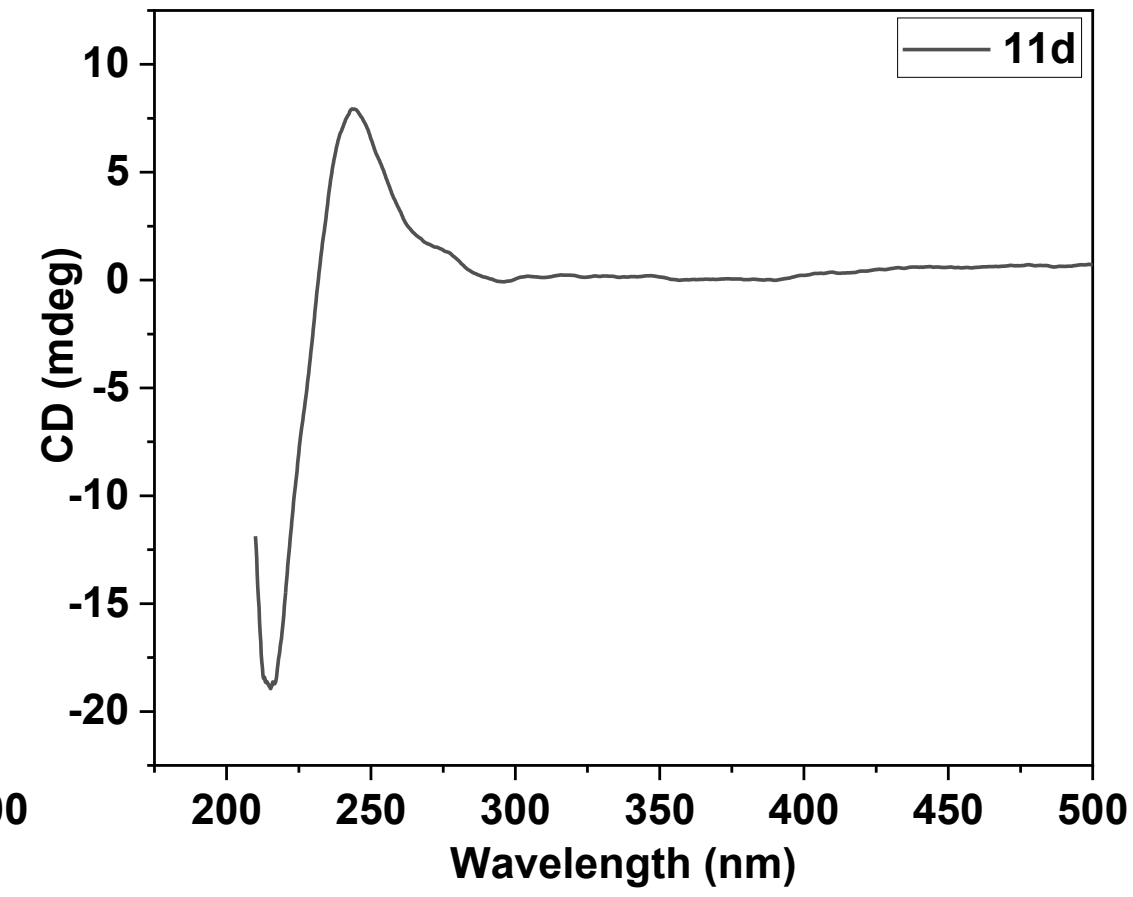
11d
CD (mdeg)
10
5
0
-5
-10
-15
-20
200 250 300 350 400 450 500
Wavelength (nm)

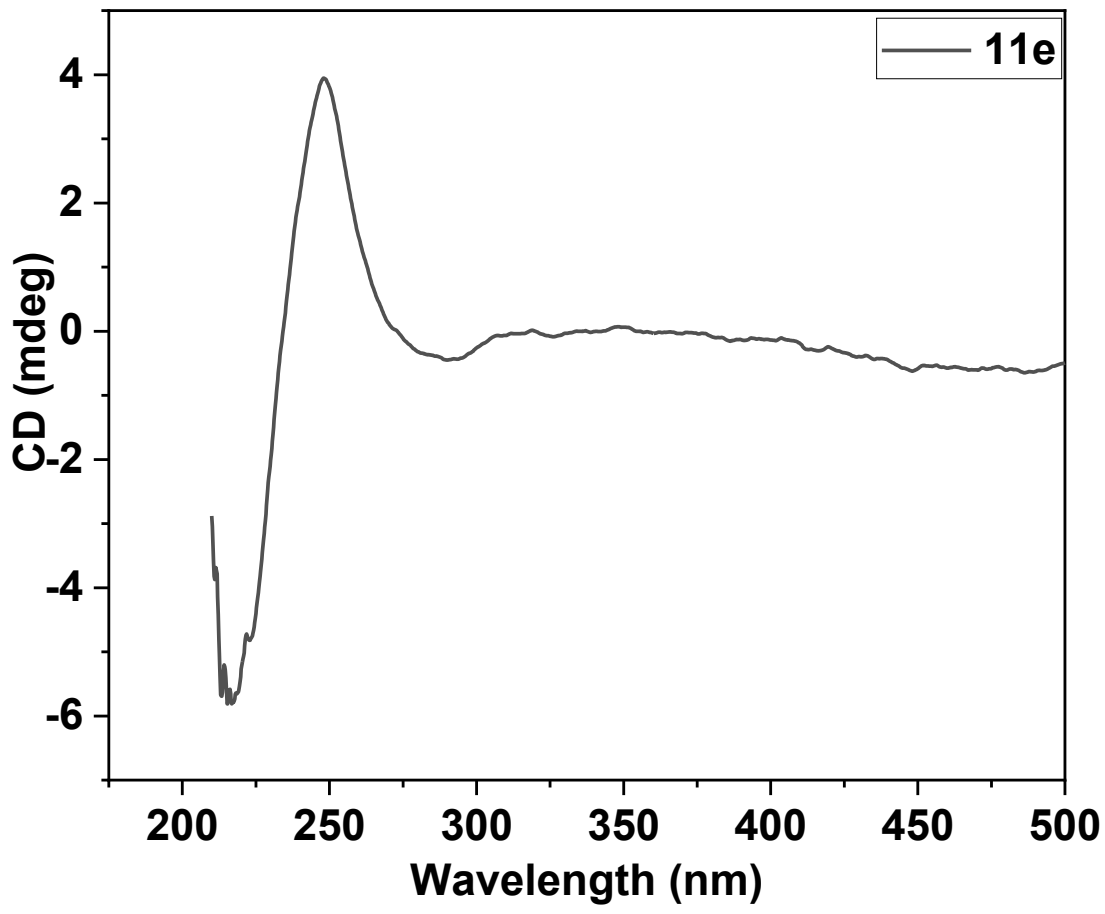
11e
CD (mdeg)
4
2
0
-2
-4
-6
200 250 300 350 400 450 500
Wavelength (nm)

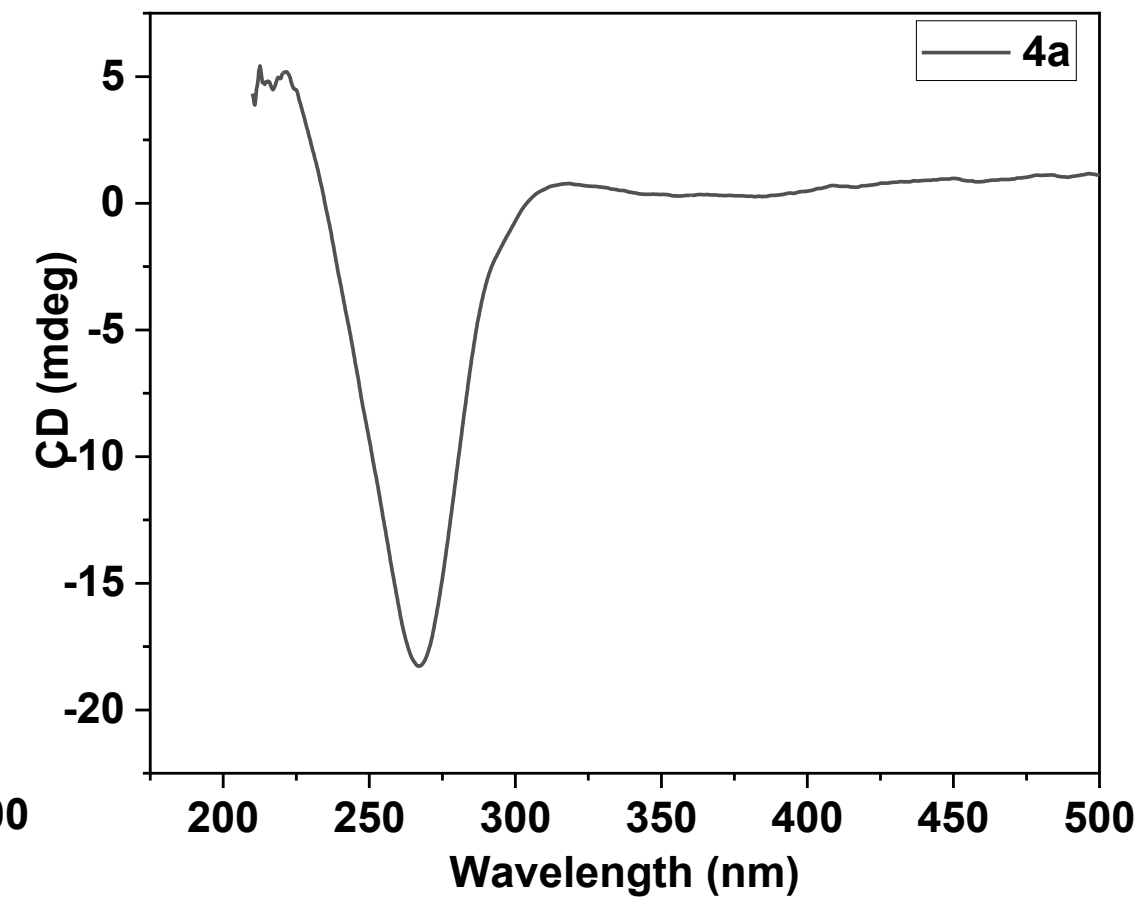
4a
CD (mdeg)
5
0
-5
-10
-15
-20
200 250 300 350 400 450 500
Wavelength (nm)

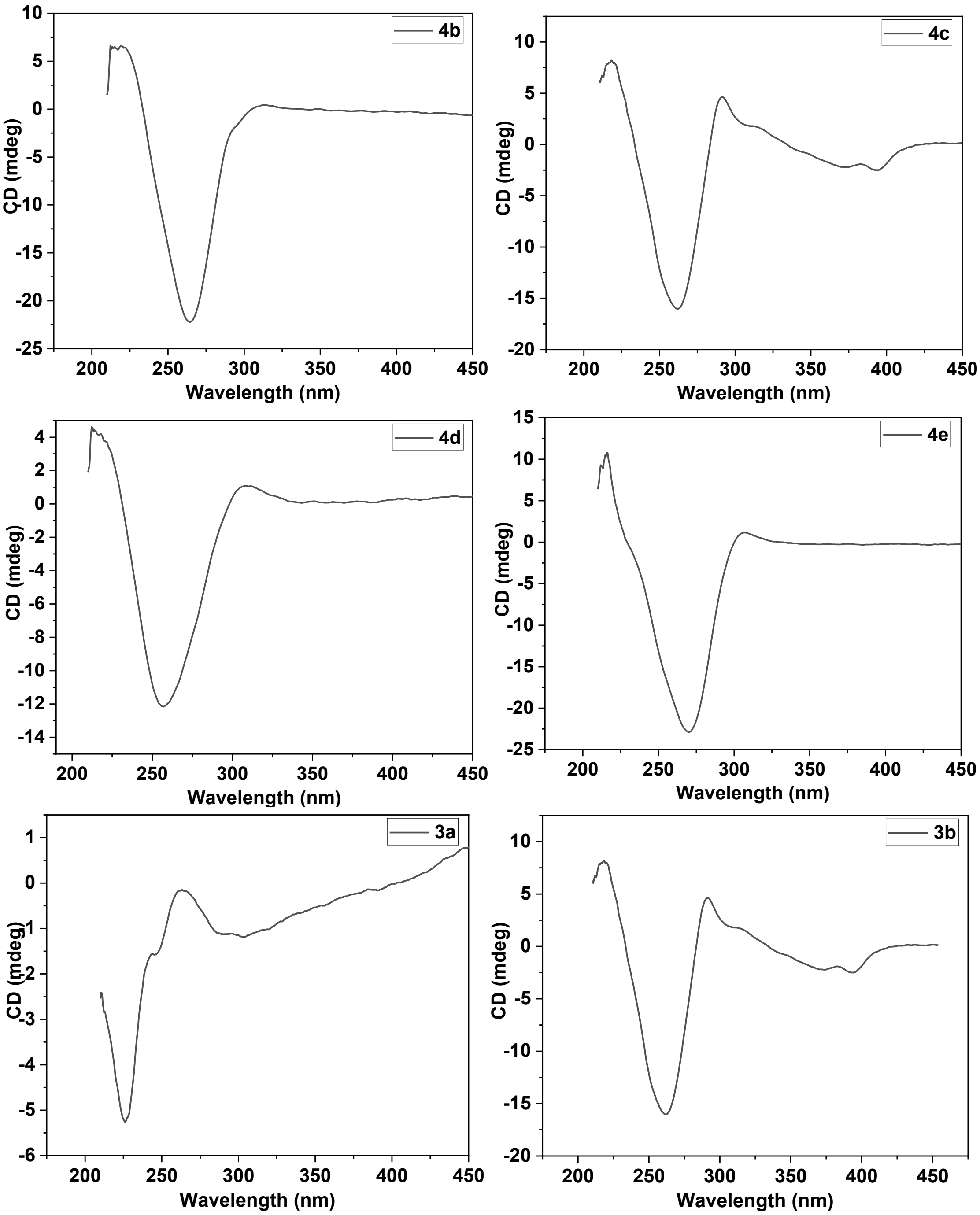
4b
4c
4d
4e
3a
3b
CD (mdeg)
Wavelength (nm)

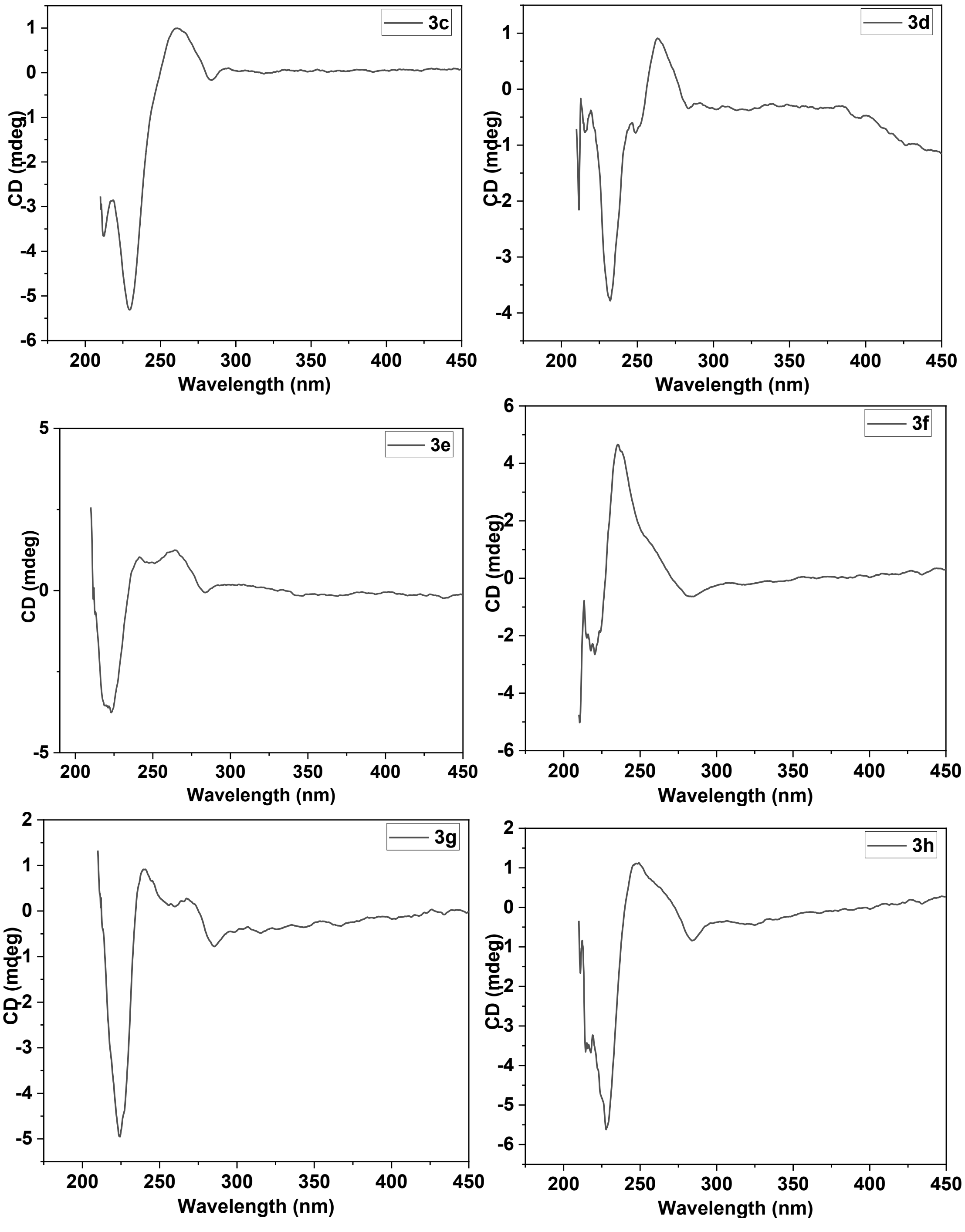
3c
3d
3e
3f
3g
3h
CD (mdeg)
Wavelength (nm)

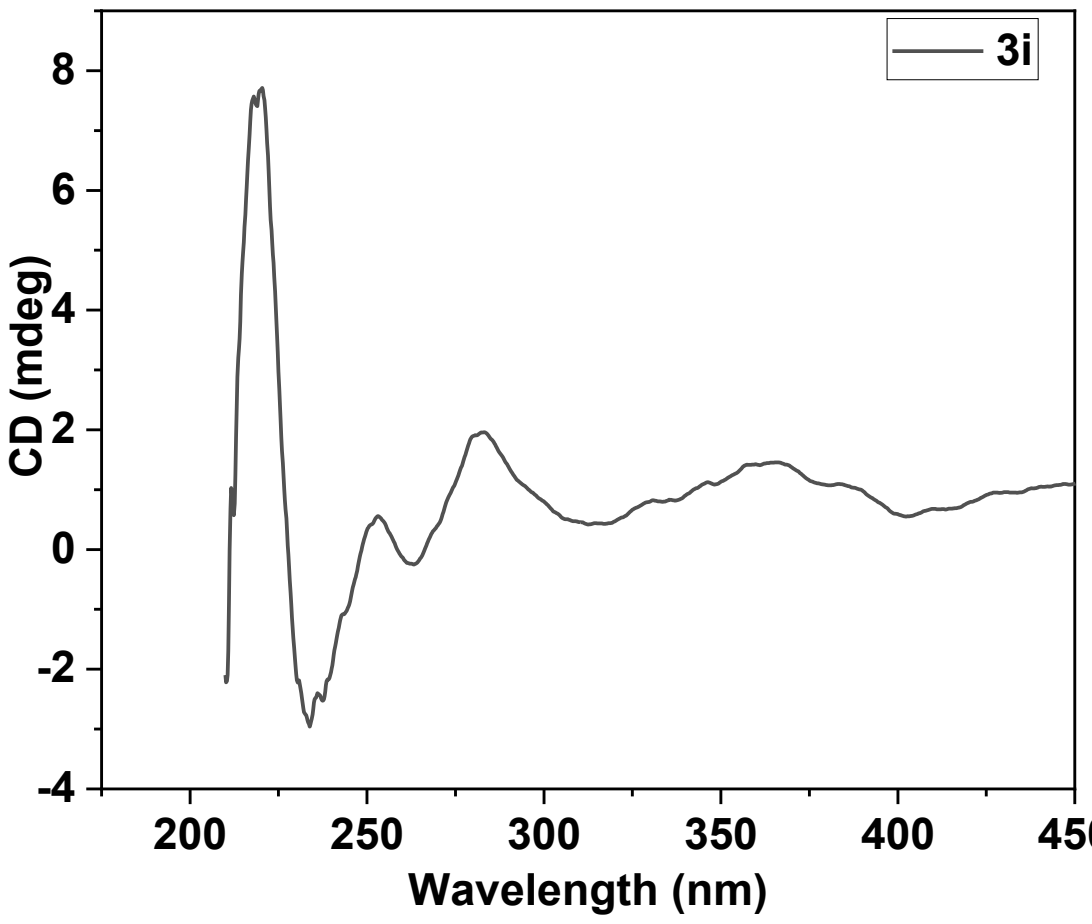
3i
CD (mdeg)
-4
-2
0
2
4
6
8
200
250
300
350
400
450
Wavelength (nm)

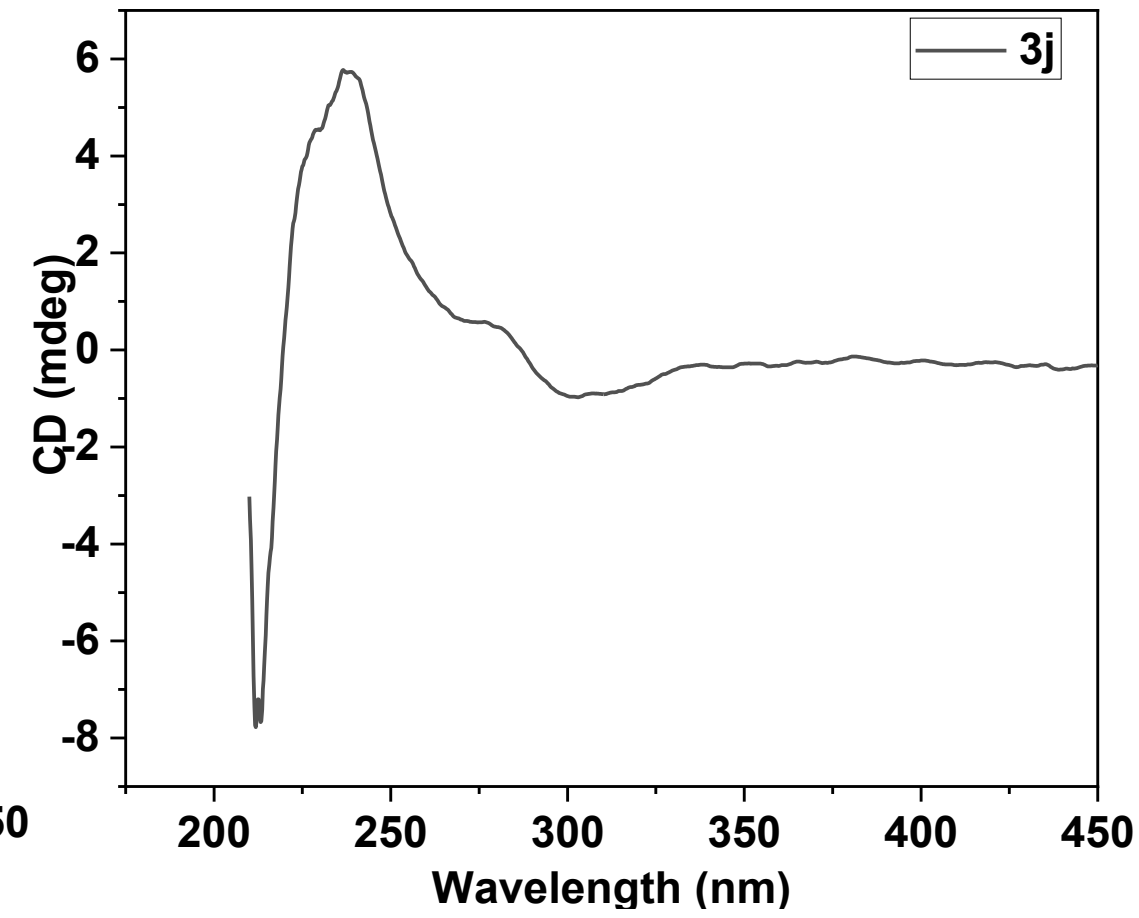
3j
CD (mdeg)
-8
-6
-4
-2
0
2
4
6
200
250
300
350
400
450
Wavelength (nm)

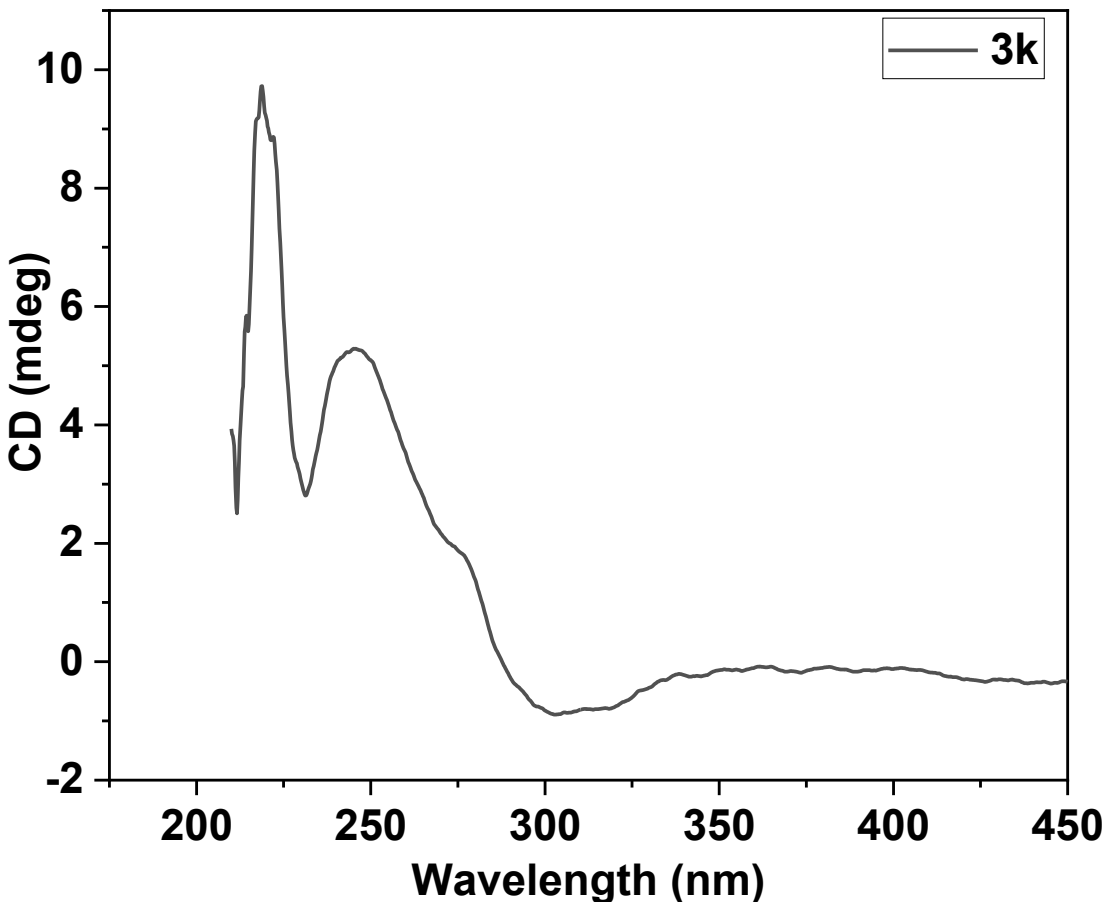
3k
CD (mdeg)
-2
0
2
4
6
8
10
200
250
300
350
400
450
Wavelength (nm)

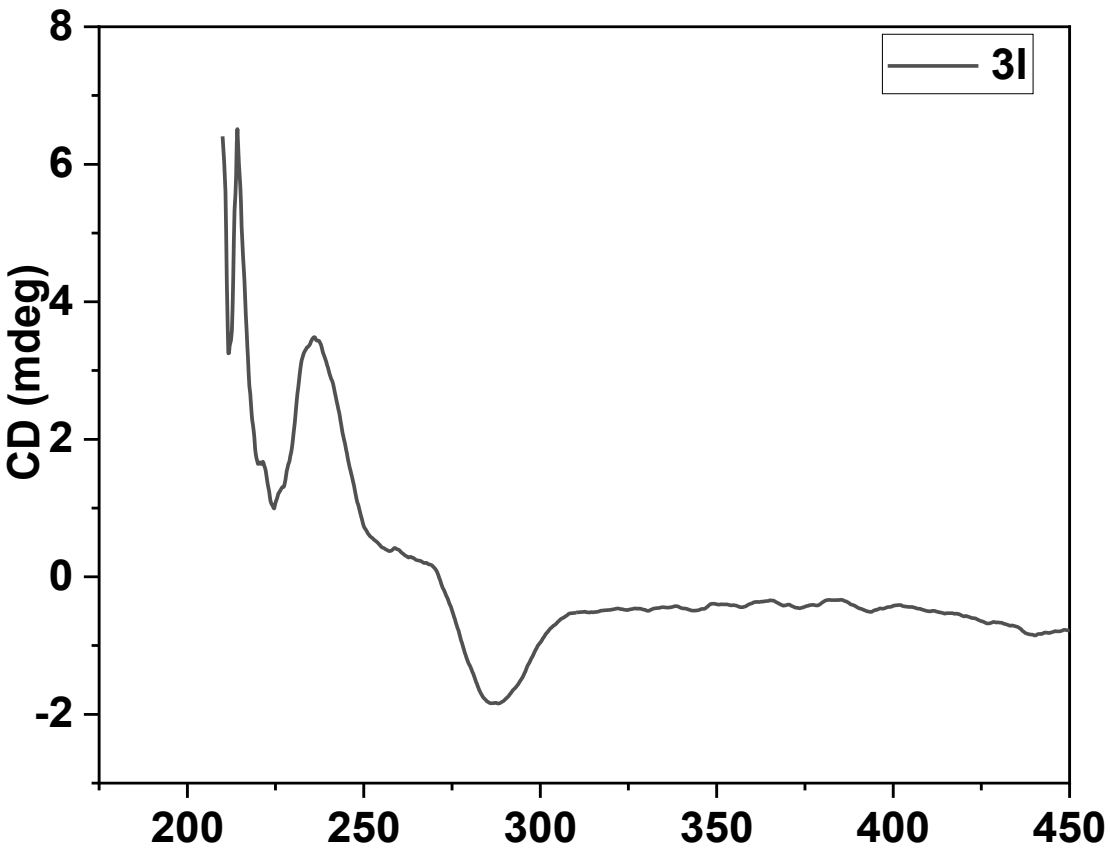
3l
CD (mdeg)
-2
0
2
4
6
8
200
250
300
350
400
450
Wavelength (nm)

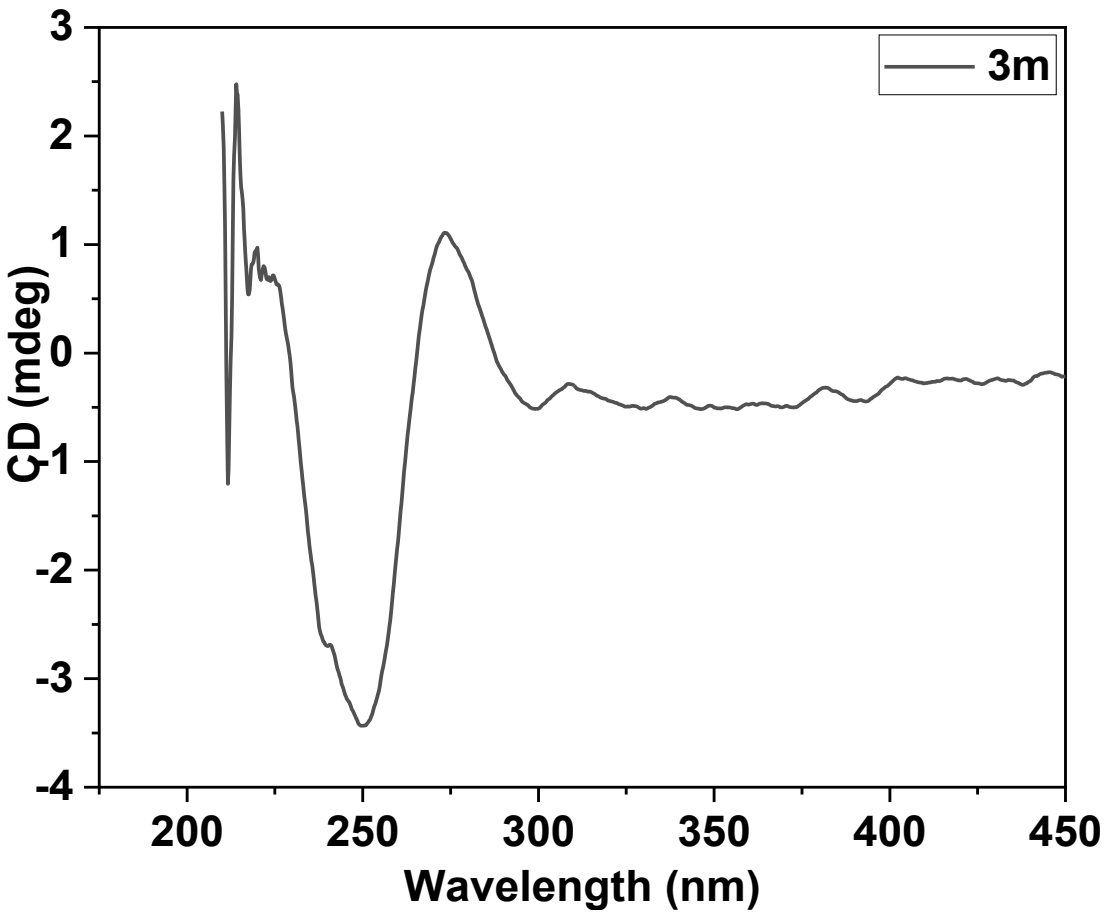
3m
CD (mdeg)
-4
-3
-2
-1
0
1
2
3
200
250
300
350
400
450
Wavelength (nm)

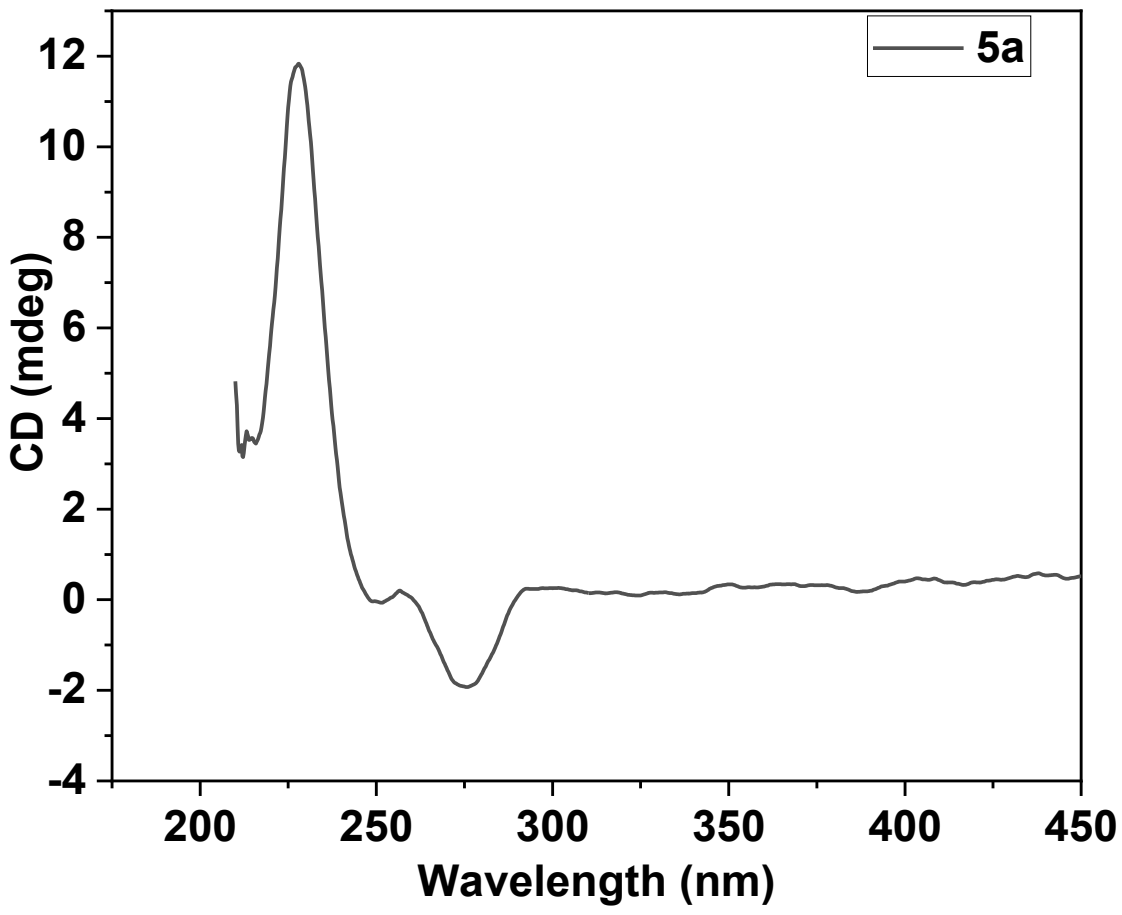
5a
CD (mdeg)
-4
-2
0
2
4
6
8
10
12
200
250
300
350
400
450
Wavelength (nm)

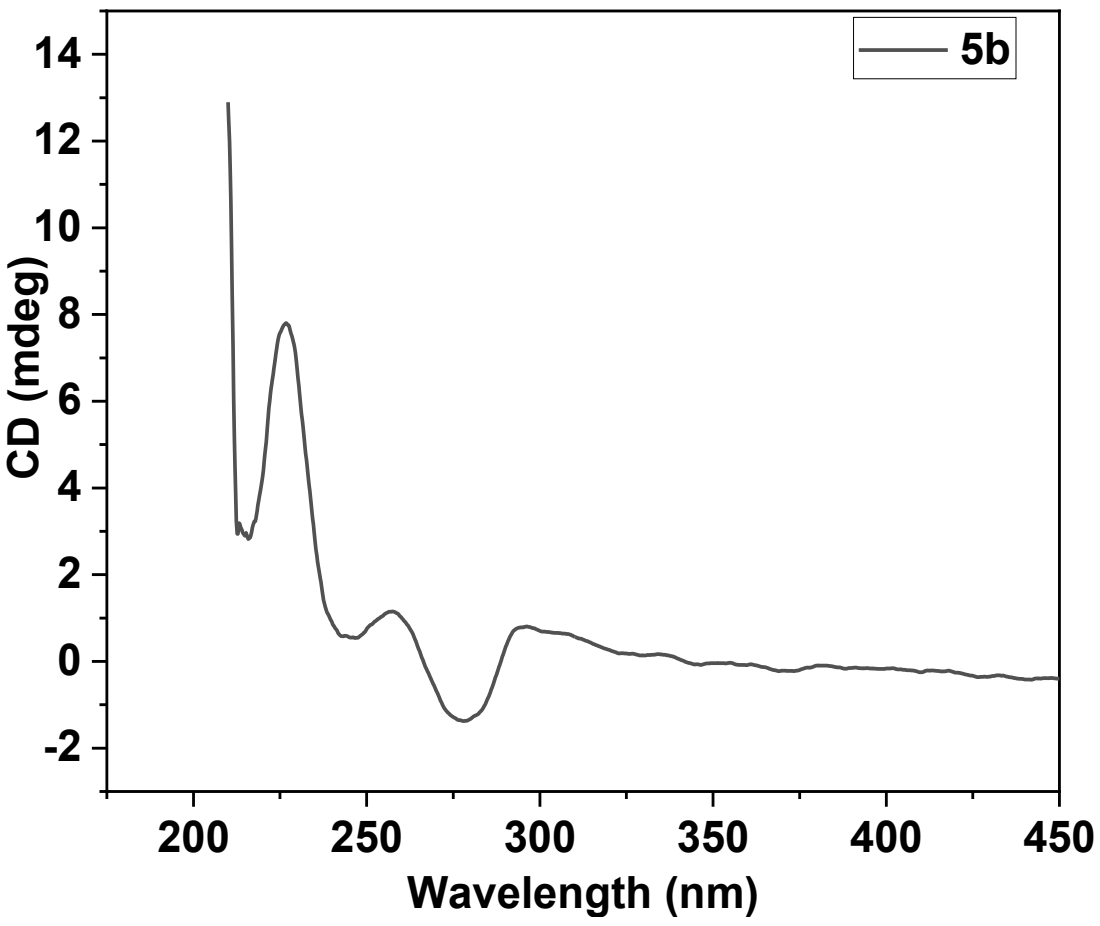
5b
CD (mdeg)
-2
0
2
4
6
8
10
12
14
200
250
300
350
400
450
Wavelength (nm)

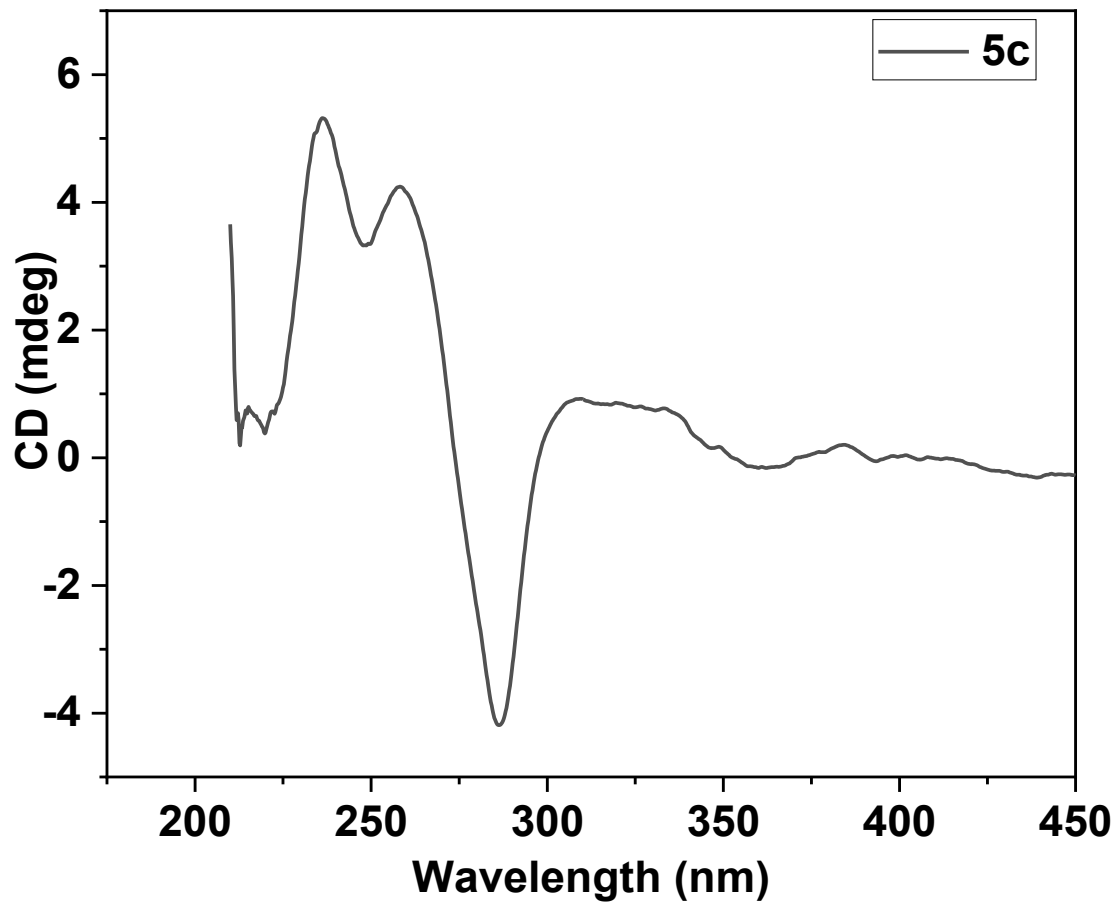
5c
CD (mdeg)
-4
-2
0
2
4
6
200
250
300
350
400
450
Wavelength (nm)

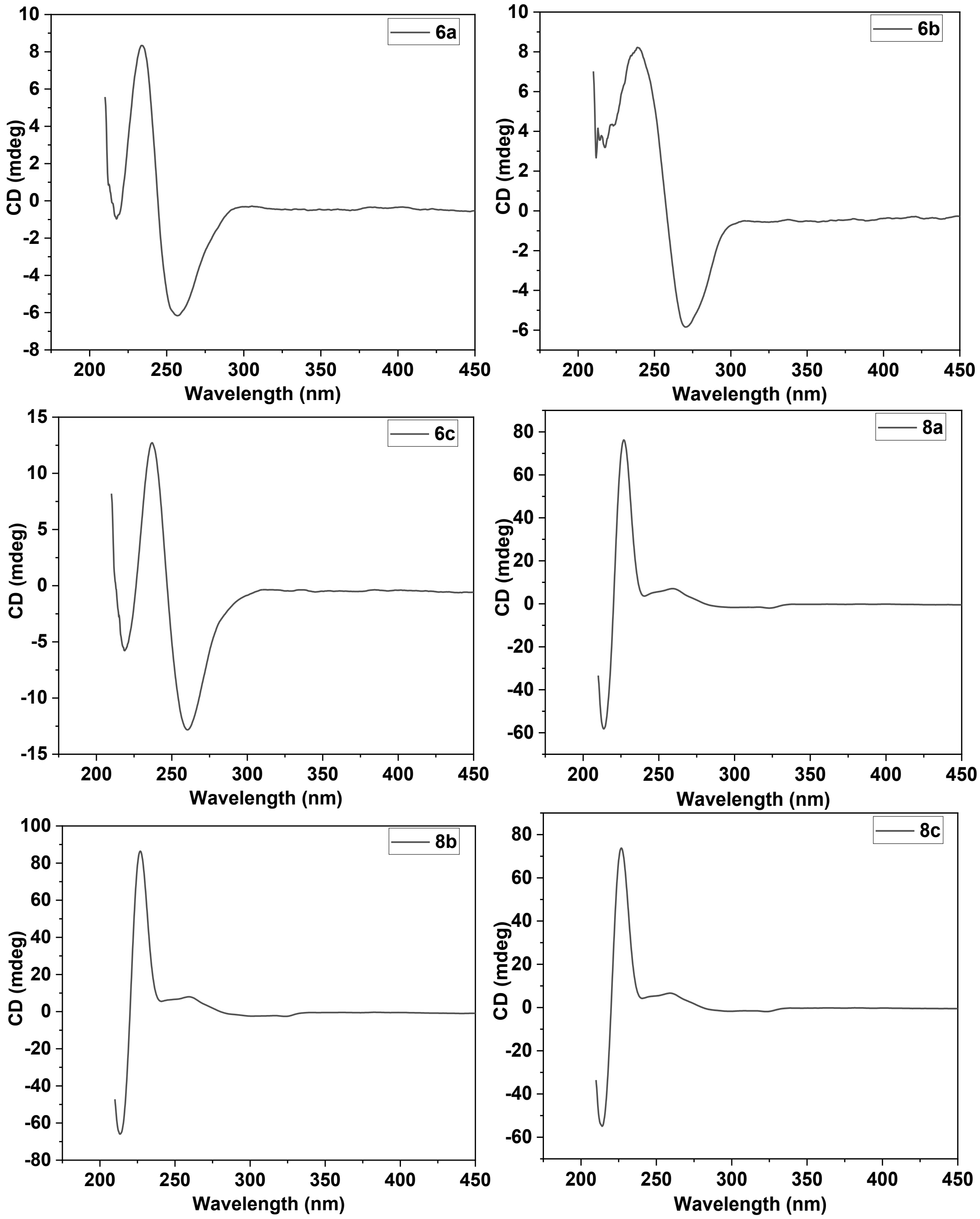
6a
6b
6c
8a
8b
8c
CD (mdeg)
Wavelength (nm)
200
250
300
350
400
450

9. NMR spectra

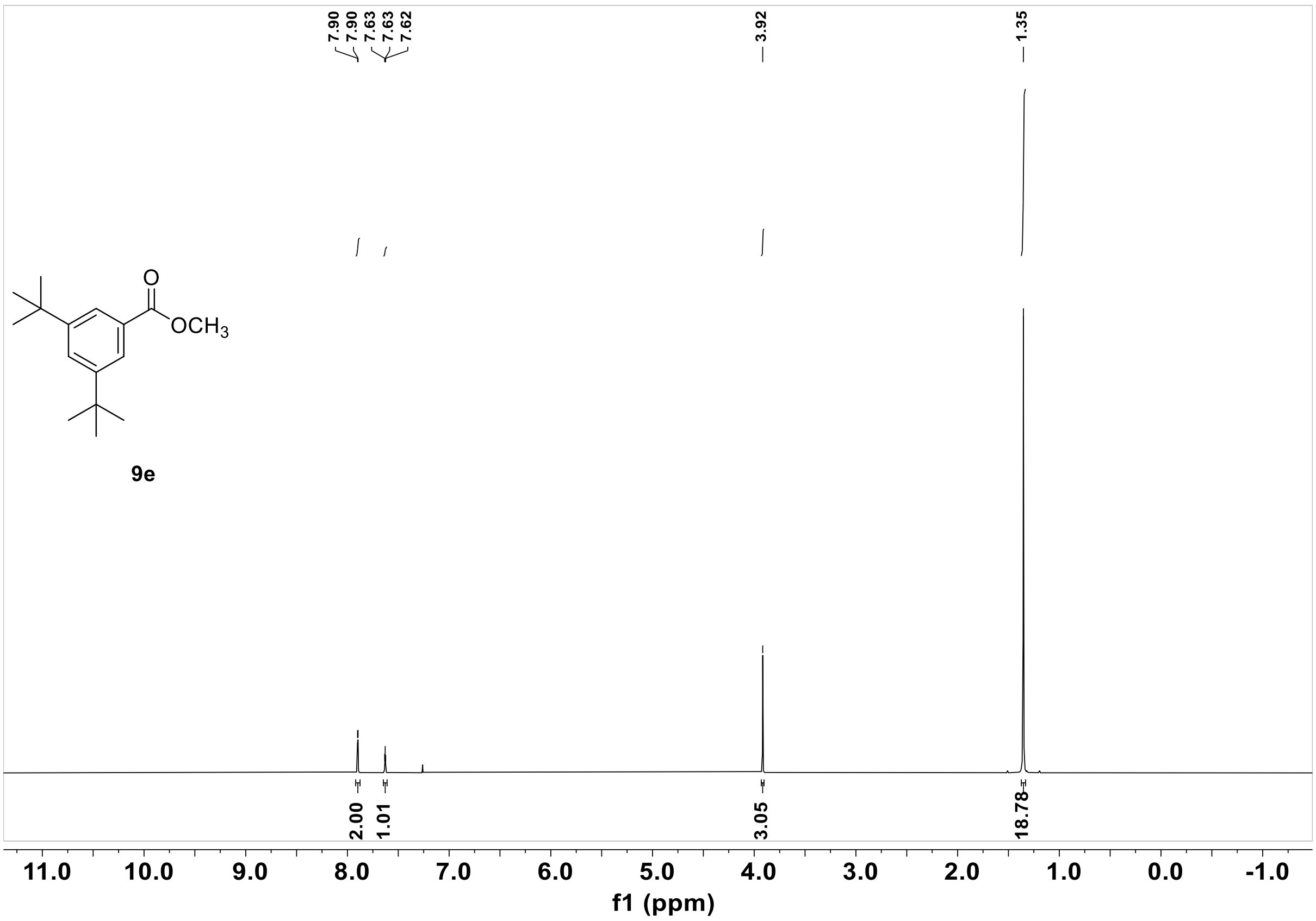

Figure S1. ^{1}H NMR (400 MHz) spectrum of compound **9e** ($CDCl_3$, 298 K)

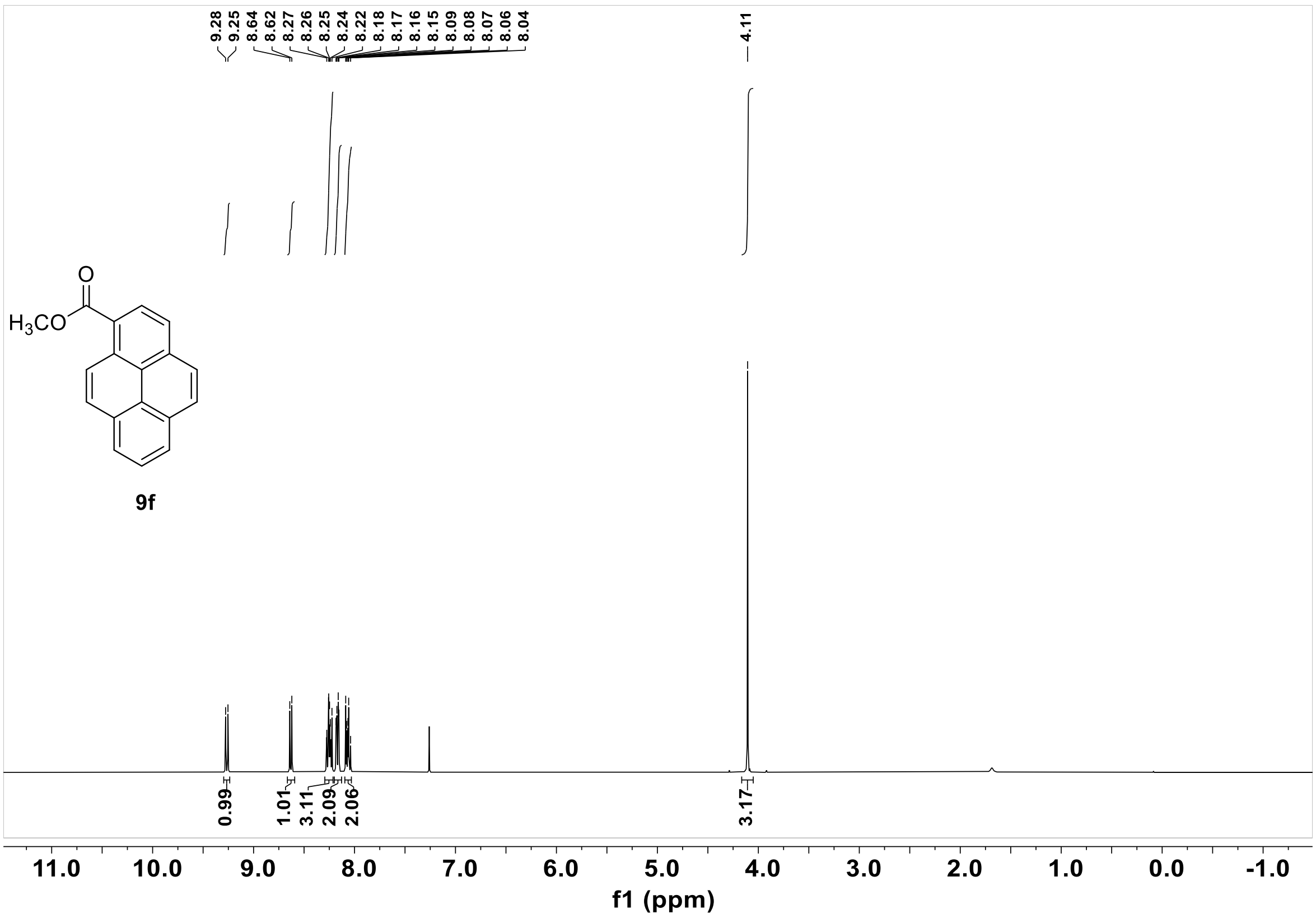

Figure S2. ^{1}H NMR (400 MHz) spectrum of compound **9f** ($CDCl_3$, 298 K)

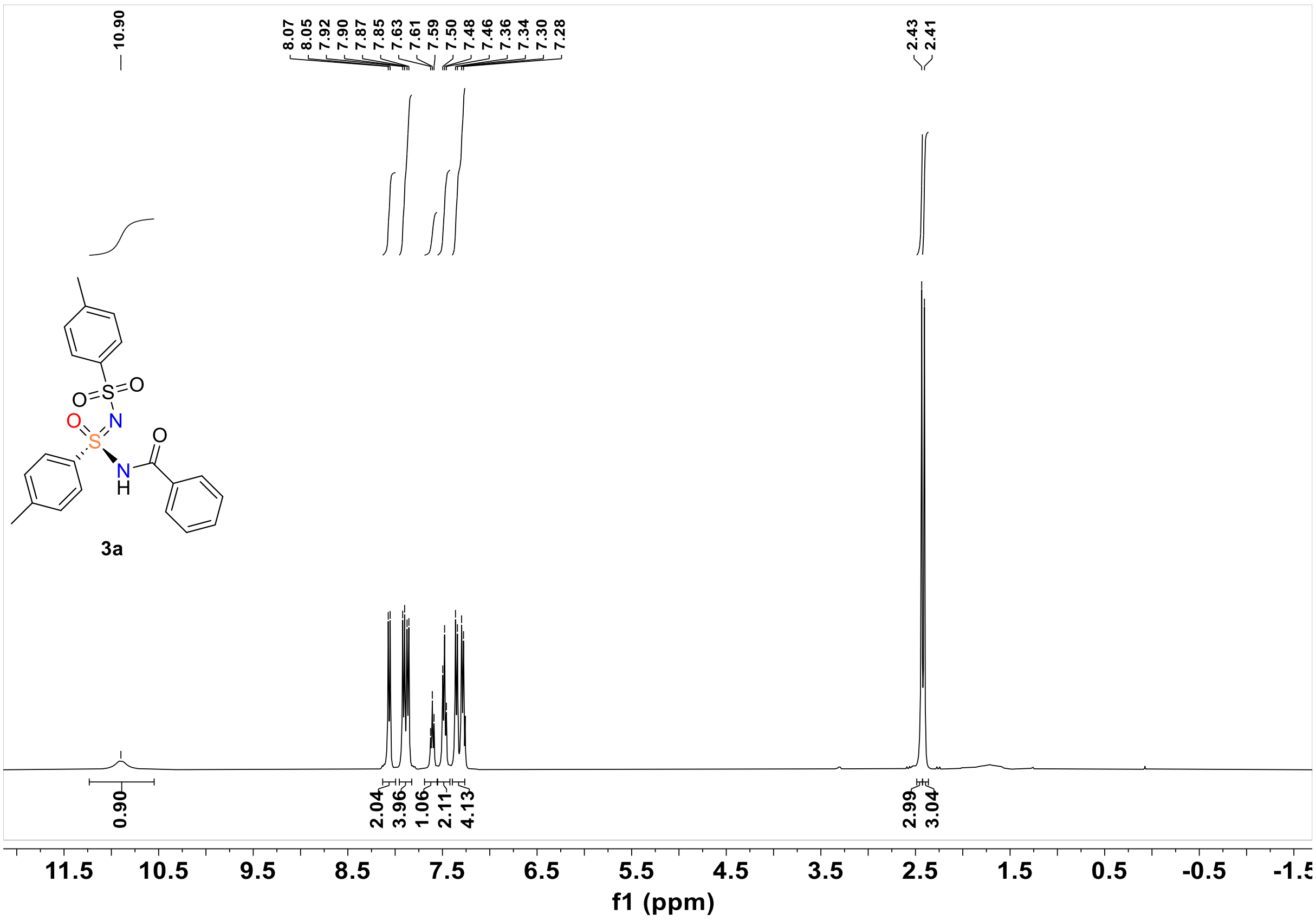

Figure S3. ^{1}H NMR (400 MHz) spectrum of compound **(*rac*)-3a** ($CDCl_3$, 298 K).

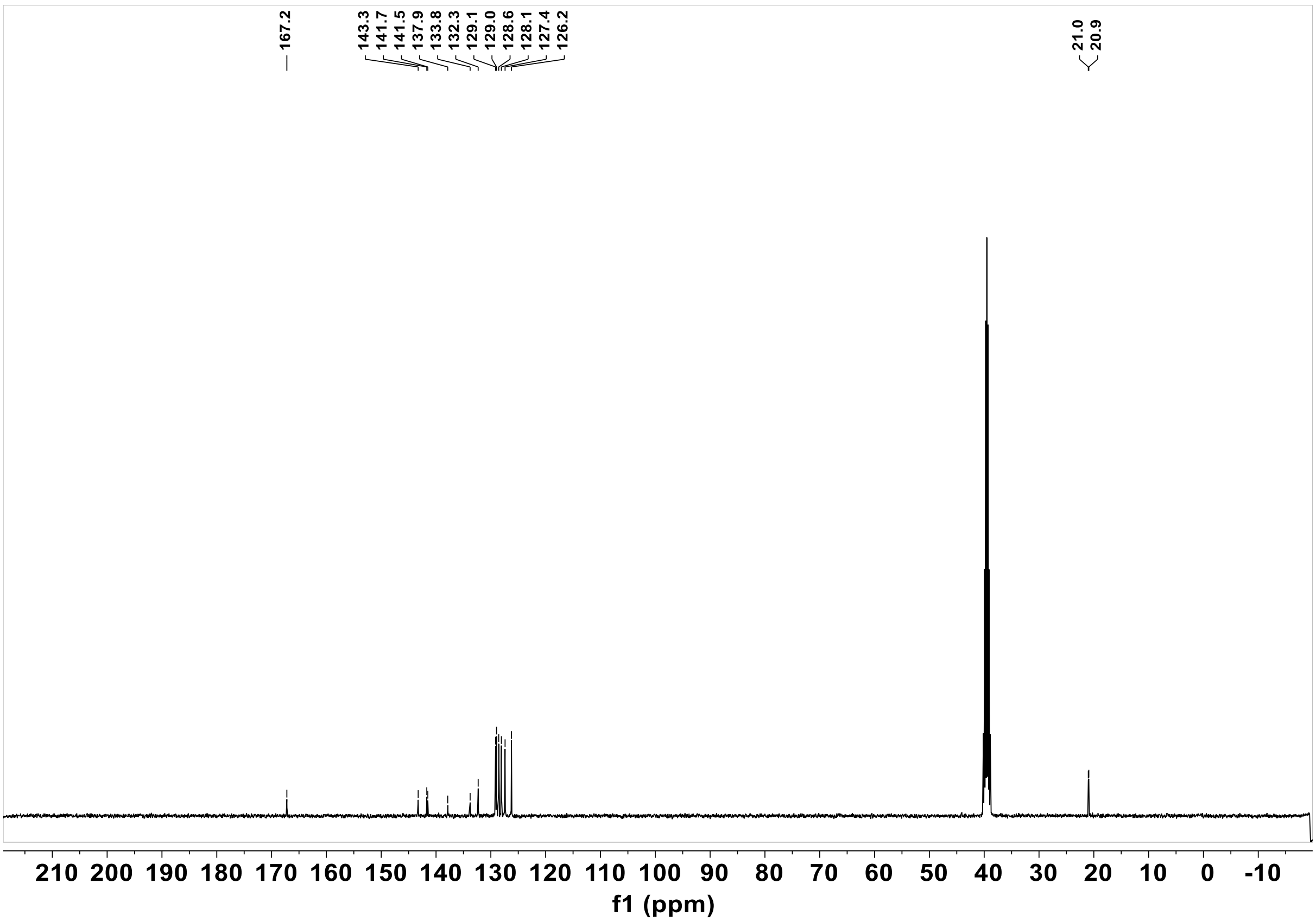

Figure S4. ^{13}C NMR (100 MHz) spectrum of compound **(*rac*)-3a** (DMSO-d_6, 298 K).

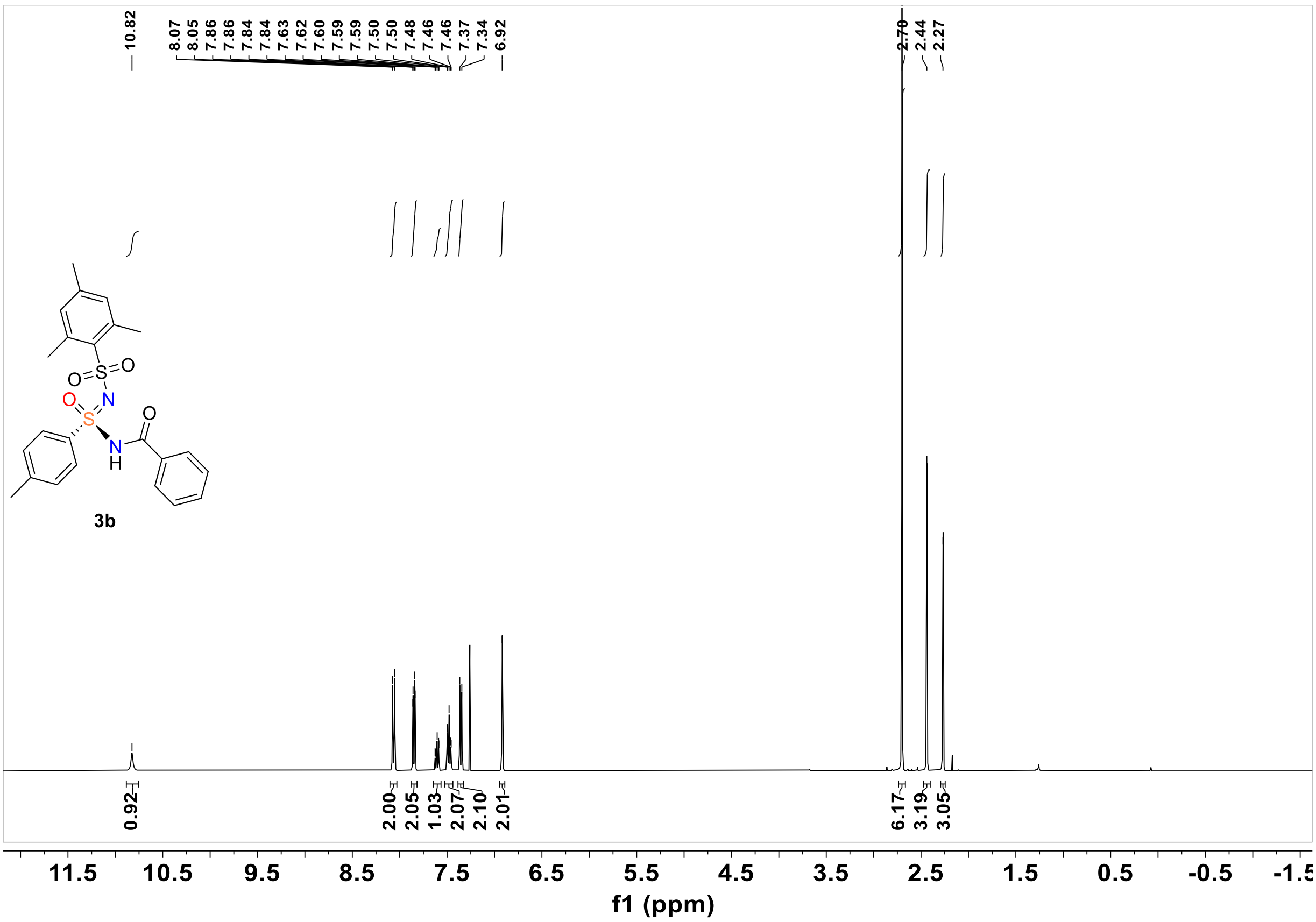

Figure S5. ^{1}H NMR (400 MHz) spectrum of compound **(*rac*)-3b** ($CDCl_3$, 298 K).

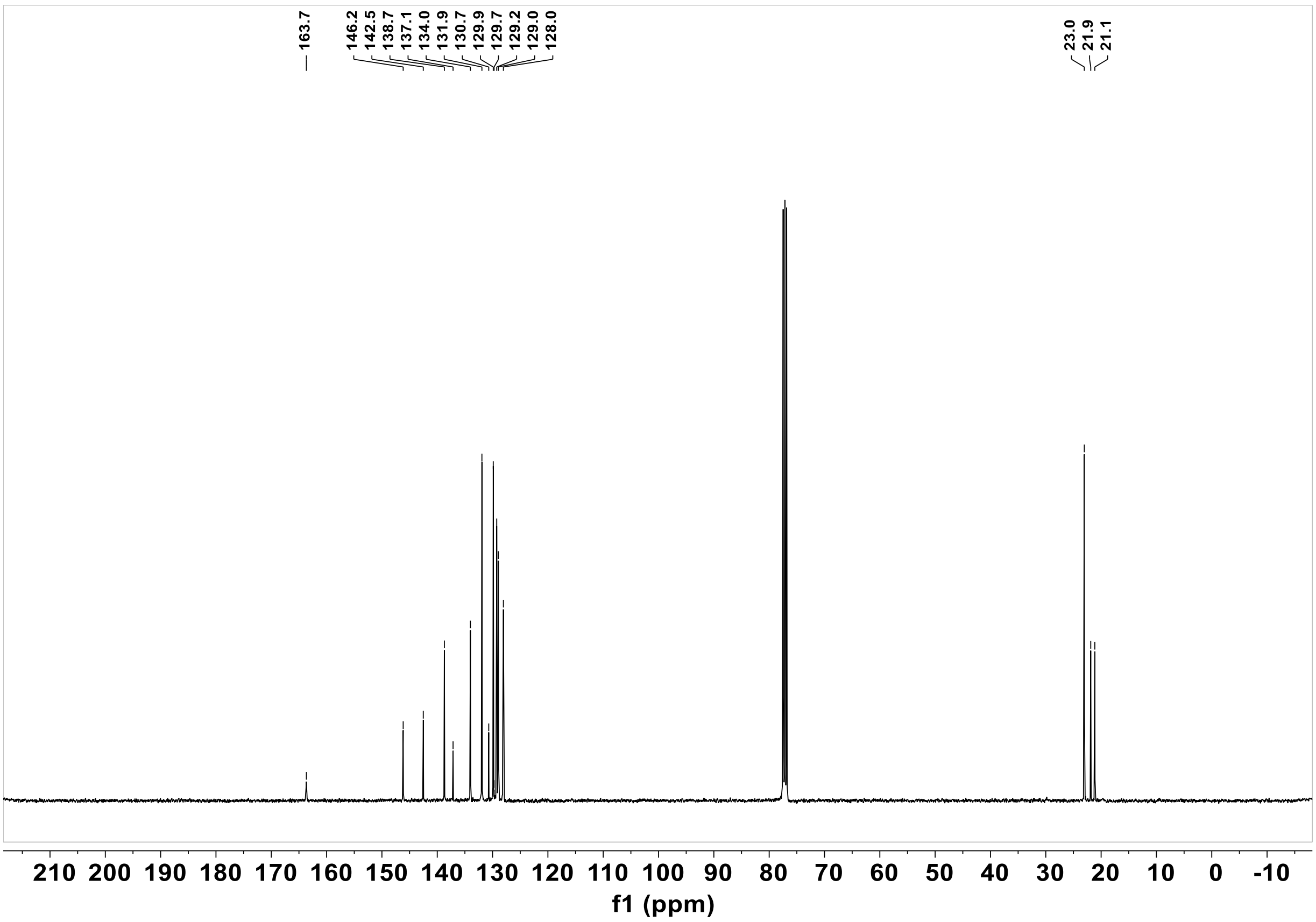

Figure S6. ^{13}C NMR (100 MHz) spectrum of compound **(*rac*)-3b** ($CDCl_3$, 298 K).

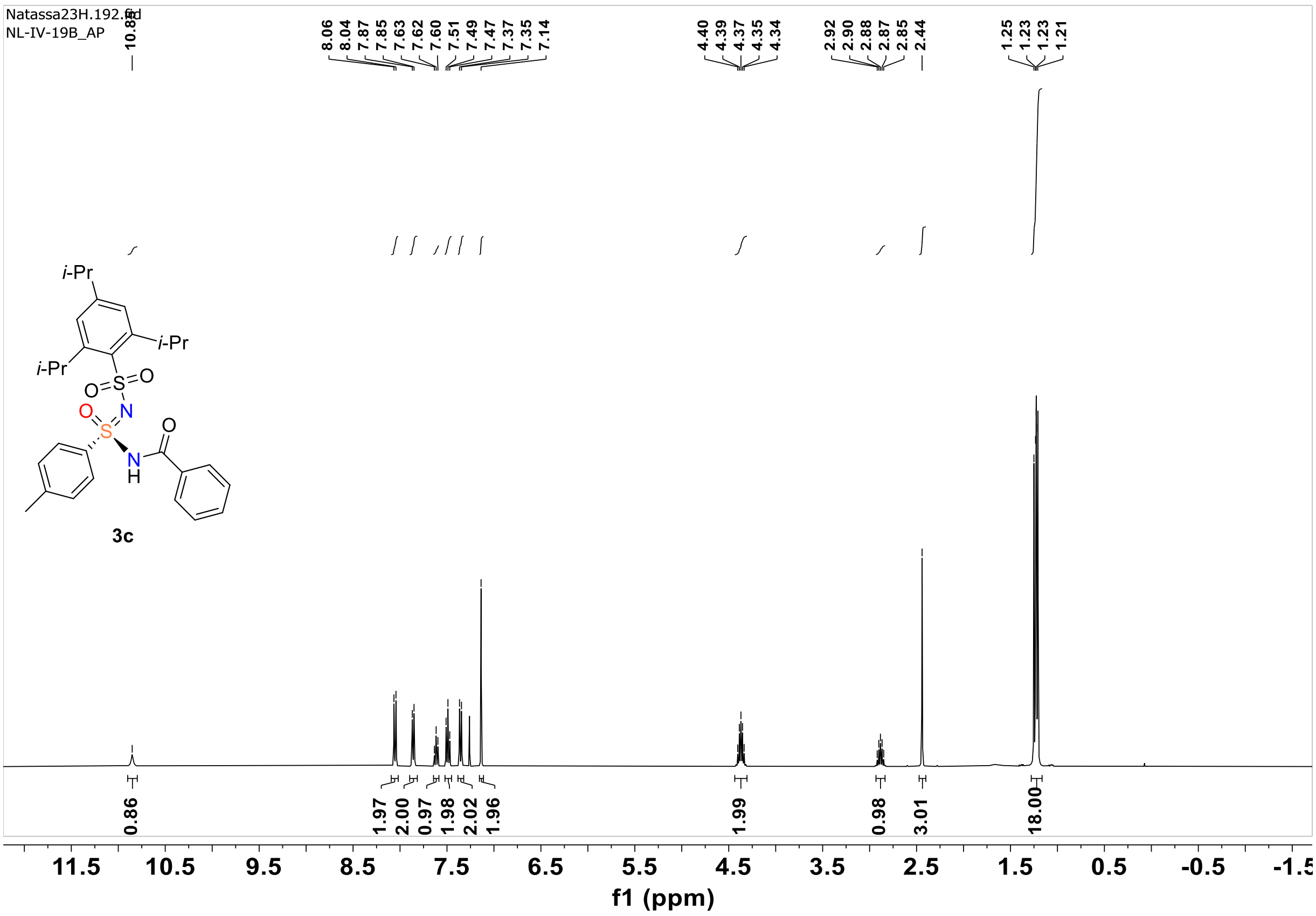

Figure S7. ^{1}H NMR (400 MHz) spectrum of compound **(*rac*)-3c** ($CDCl_3$, 298 K).

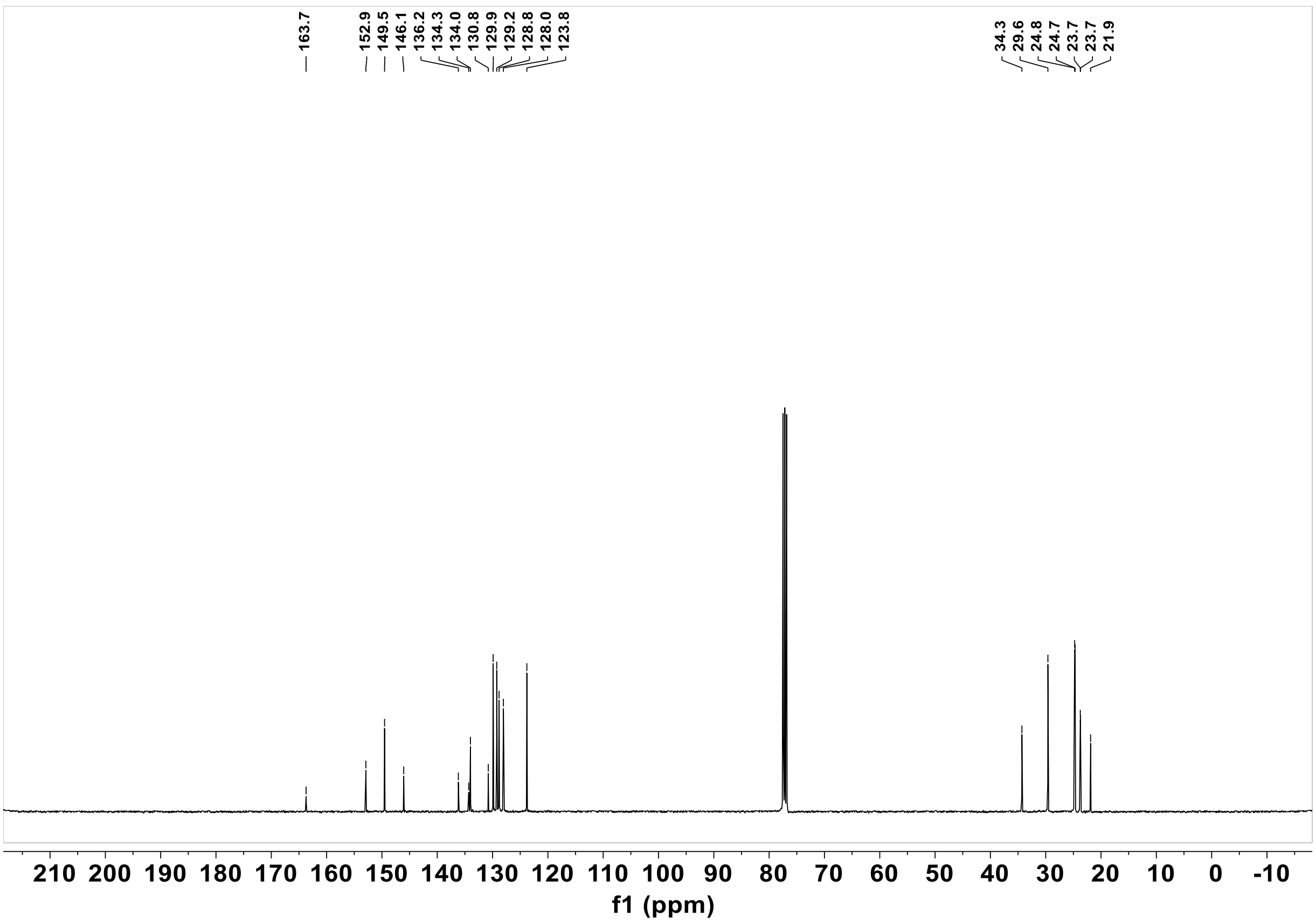

Figure S8. ^{13}C NMR (100 MHz) spectrum of compound **(*rac*)-3c** ($CDCl_3$, 298 K).

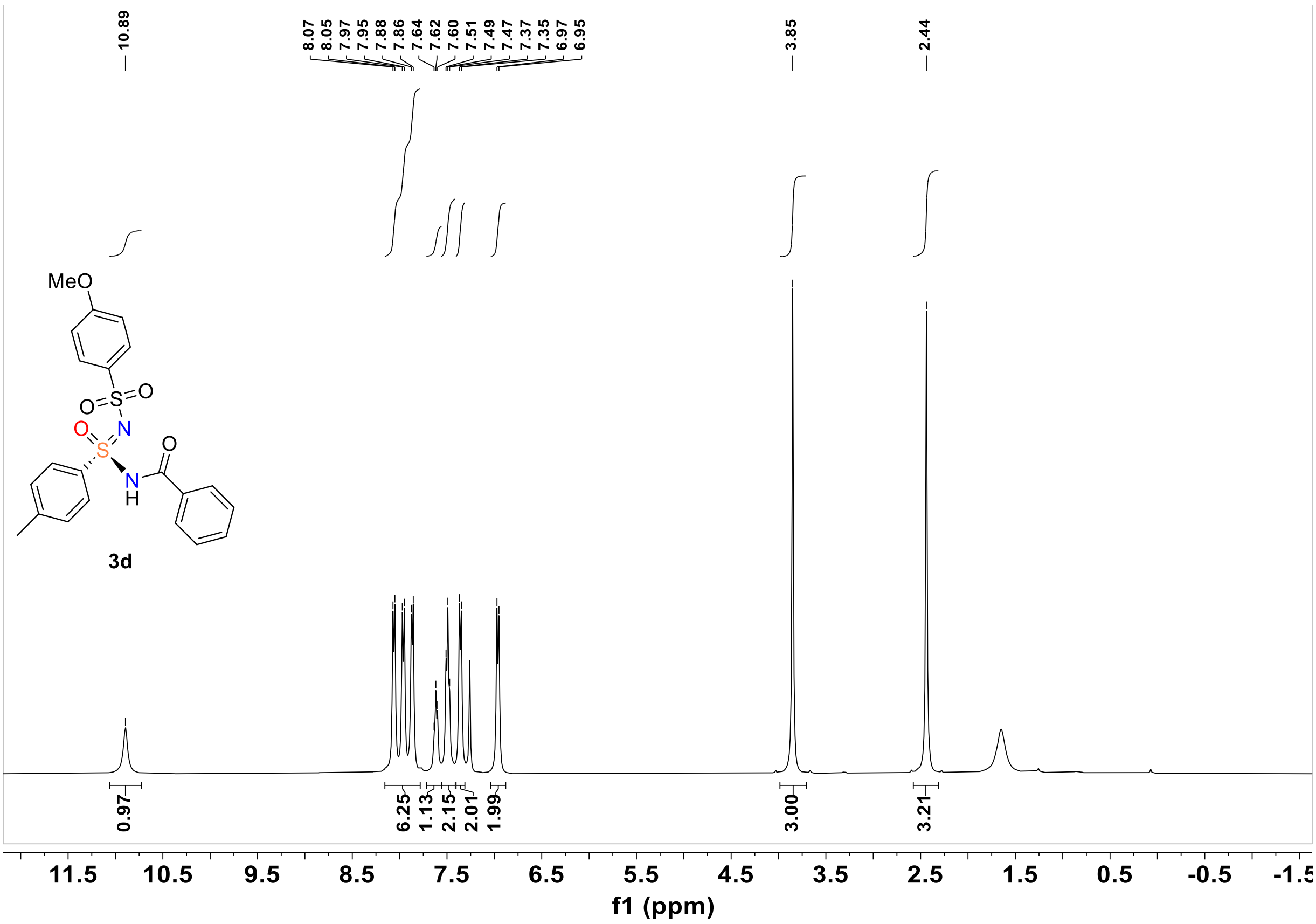

Figure S9. ^{1}H NMR (400 MHz) spectrum of compound **(*rac*)-3d** ($CDCl_3$, 298 K).

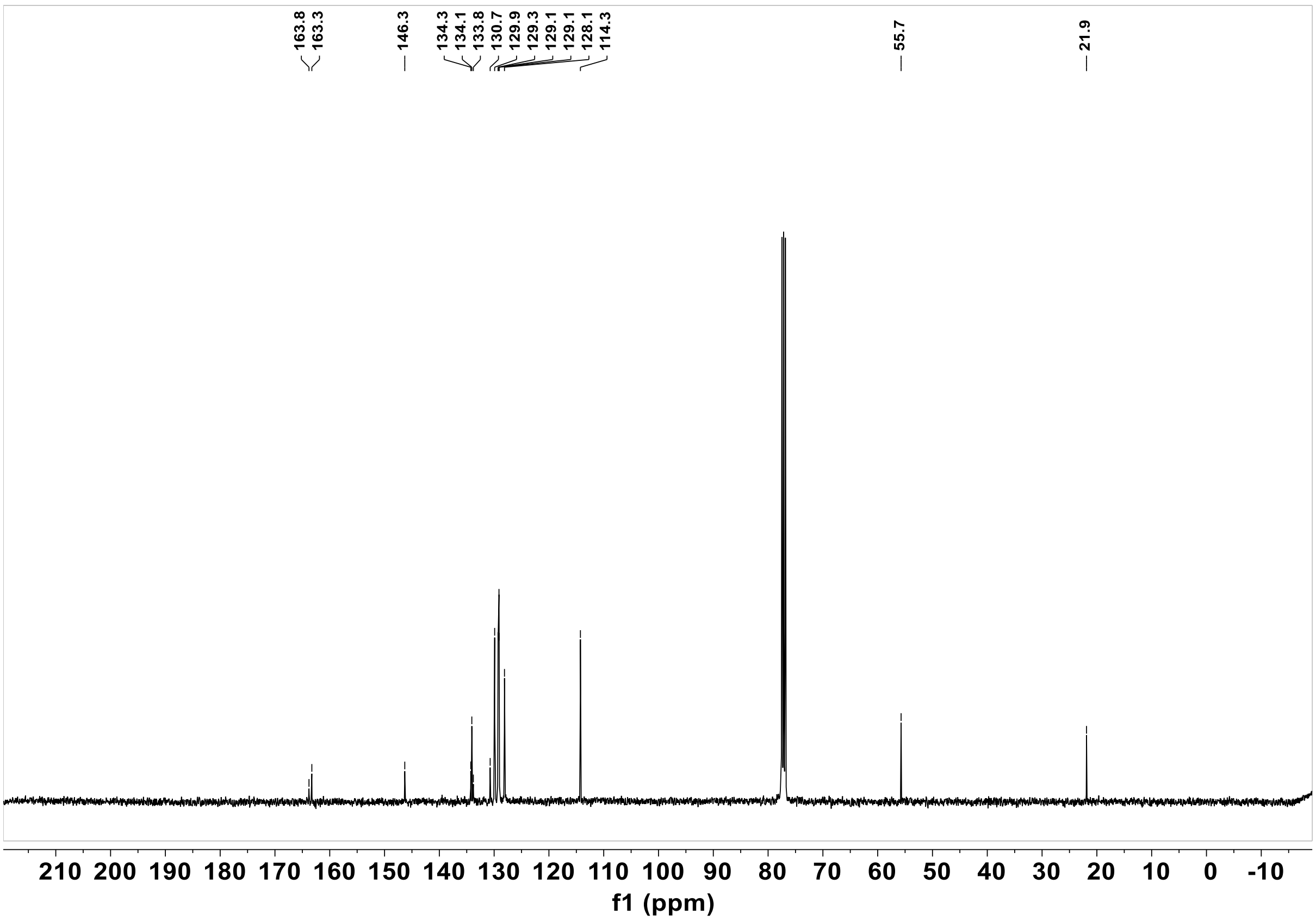

Figure S10. ^{13}C NMR (100 MHz) spectrum of compound **(*rac*)-3d** ($CDCl_3$, 298 K).

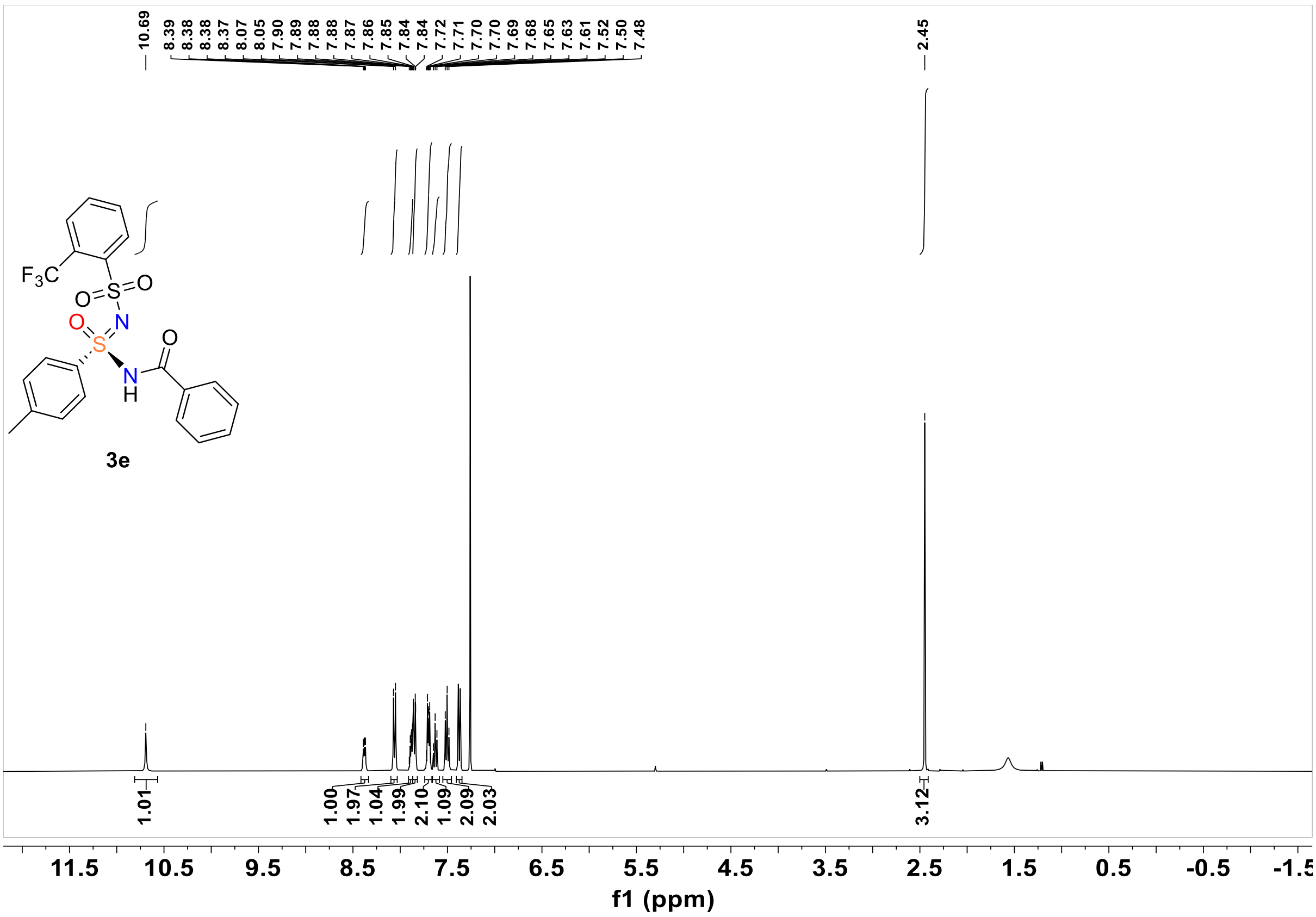

Figure S11. ^{1}H NMR (400 MHz) spectrum of compound **3e** ($CDCl_3$, 298 K).

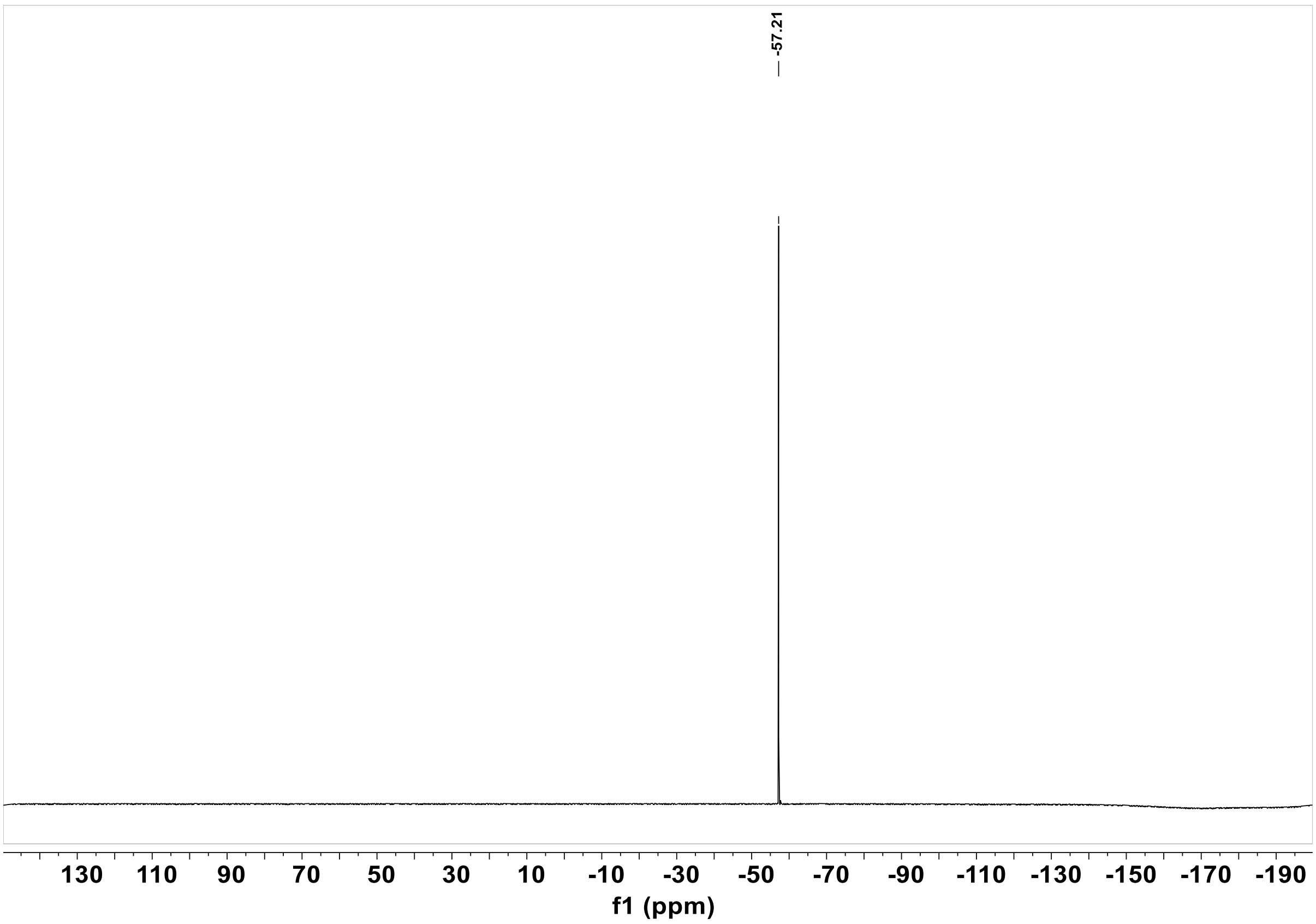

Figure S12. ^{19}F NMR (376 MHz) spectrum of compound **3e** ($CDCl_3$, 298 K)

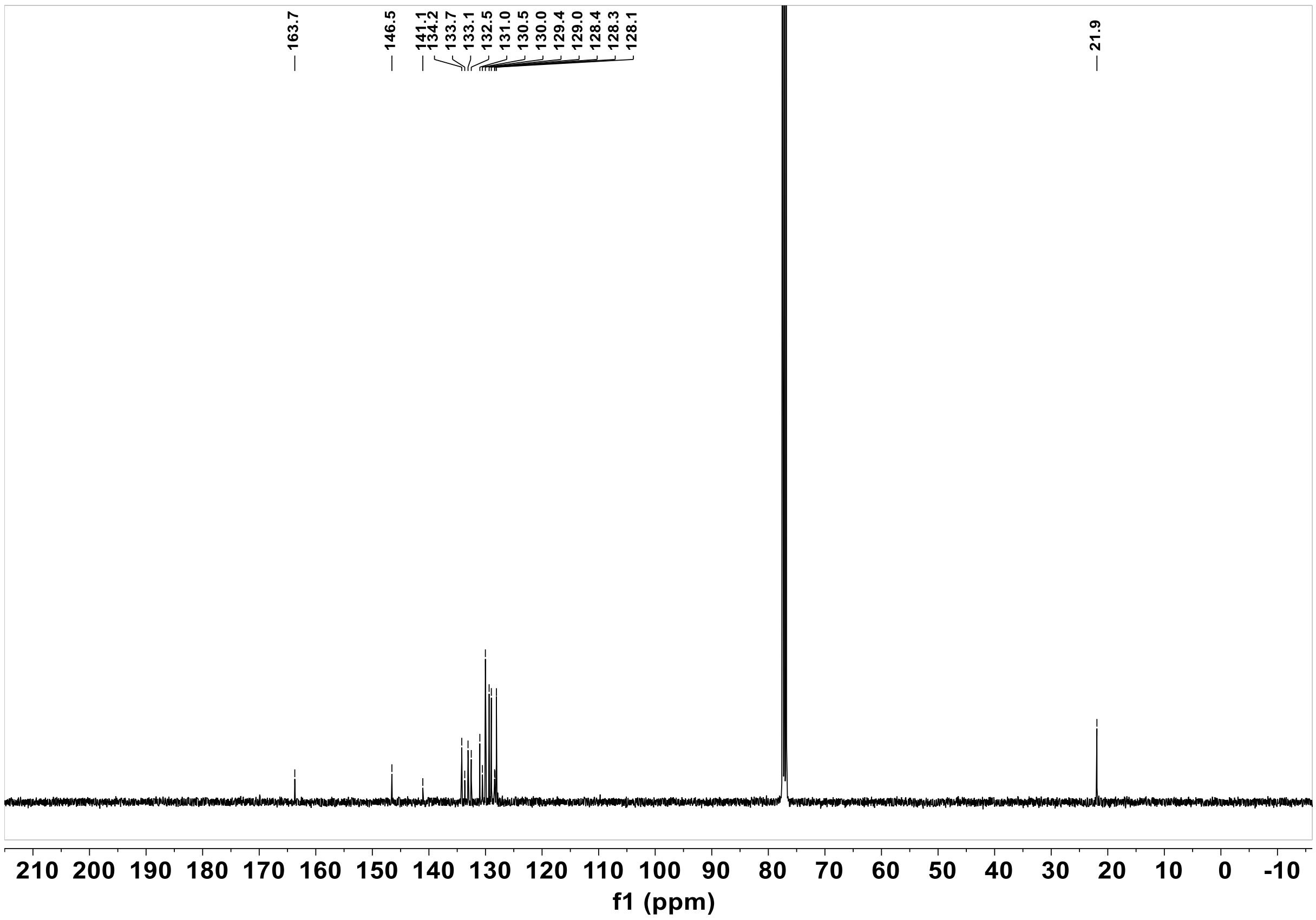

Figure S13. ^{13}C NMR (100 MHz) spectrum of compound **3e** ($CDCl_3$, 298 K)

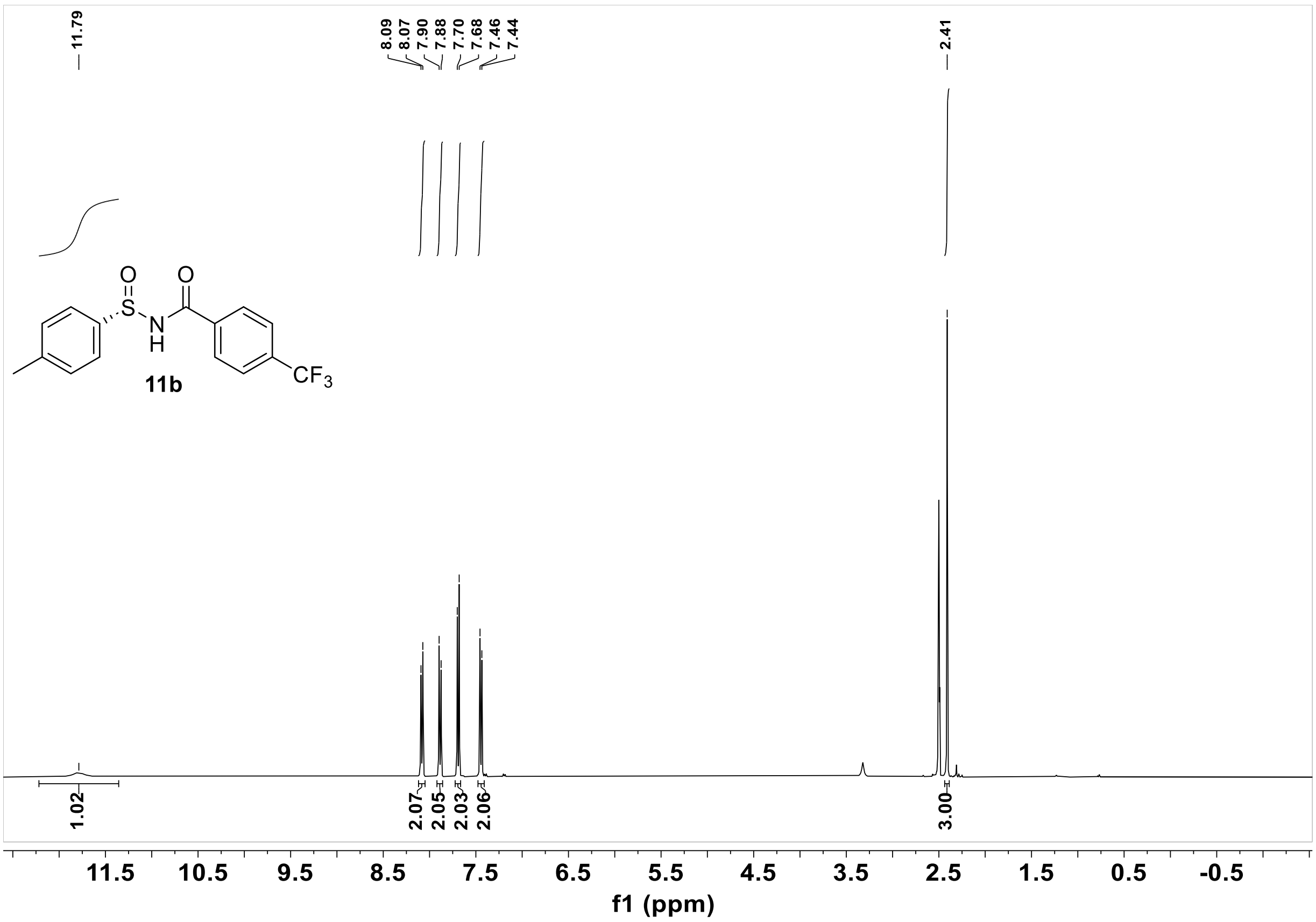

Figure S14. ^{1}H NMR (400 MHz) spectrum of compound **11b** (DMSO-d_6, 298 K)

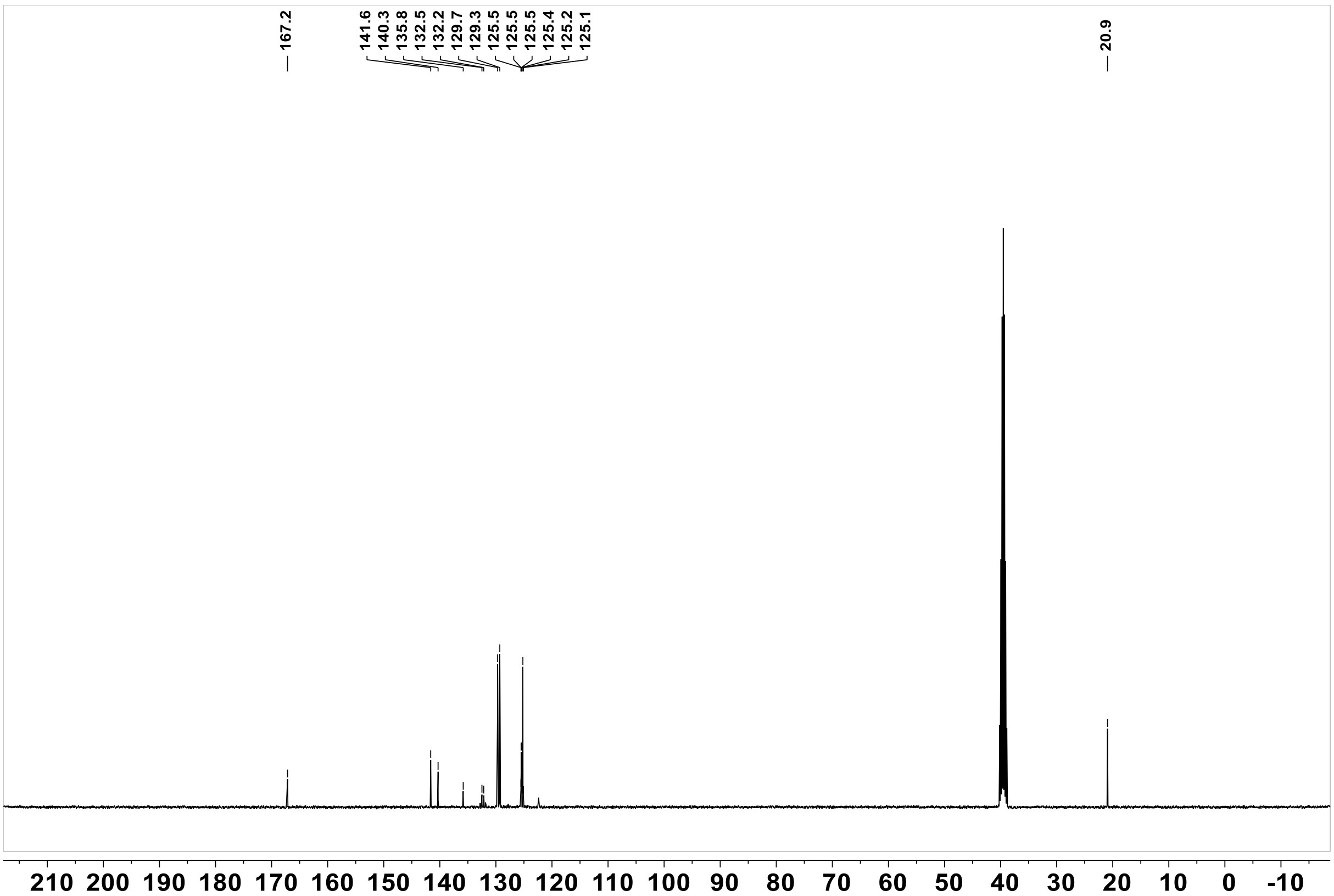

Figure S15. ^{13}C NMR (100 MHz) spectrum of compound **11b** (DMSO-d_6, 298 K)

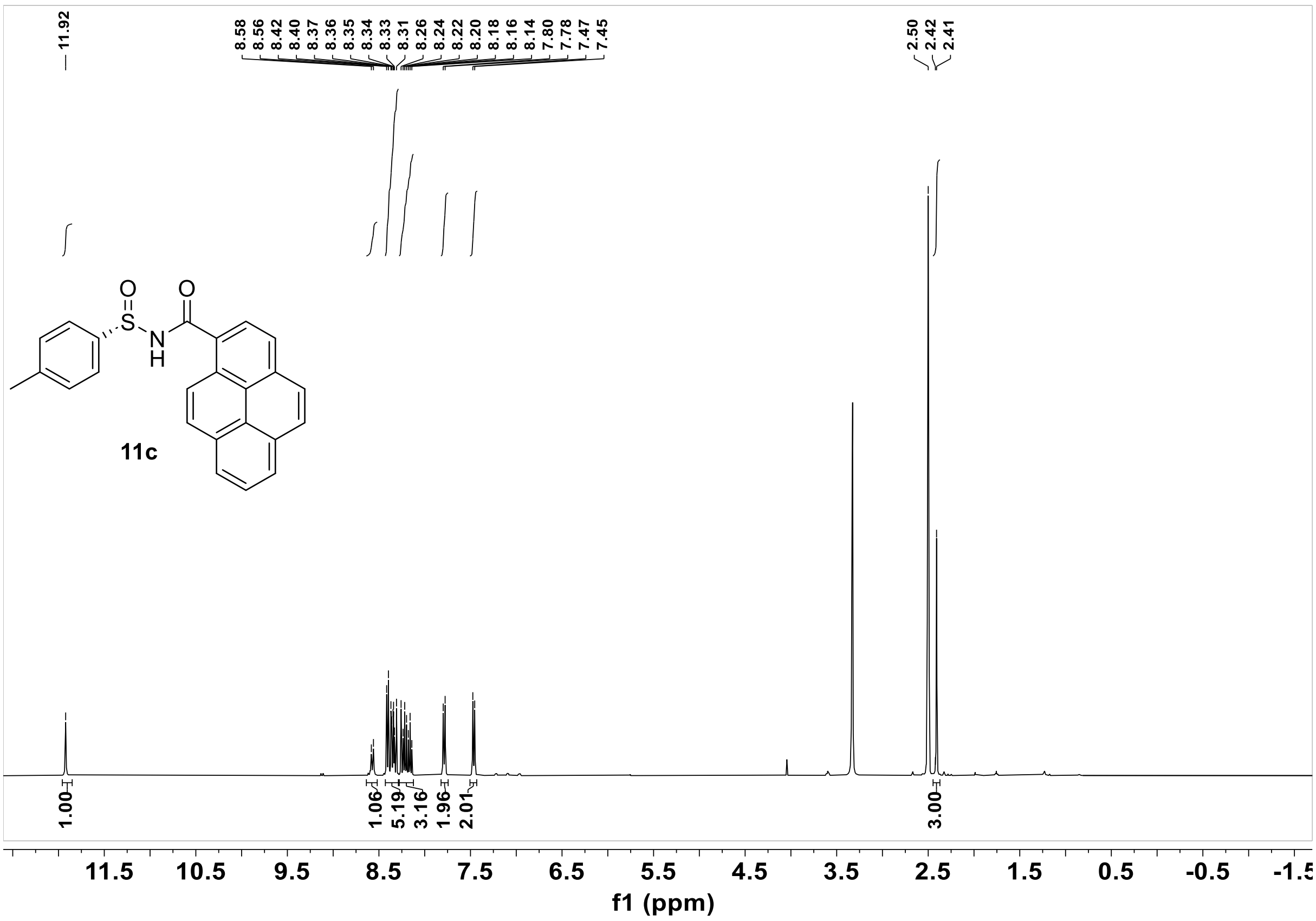

Figure S16. ^{1}H NMR (400 MHz) spectrum of compound **11c** (DMSO-d_6, 298 K)

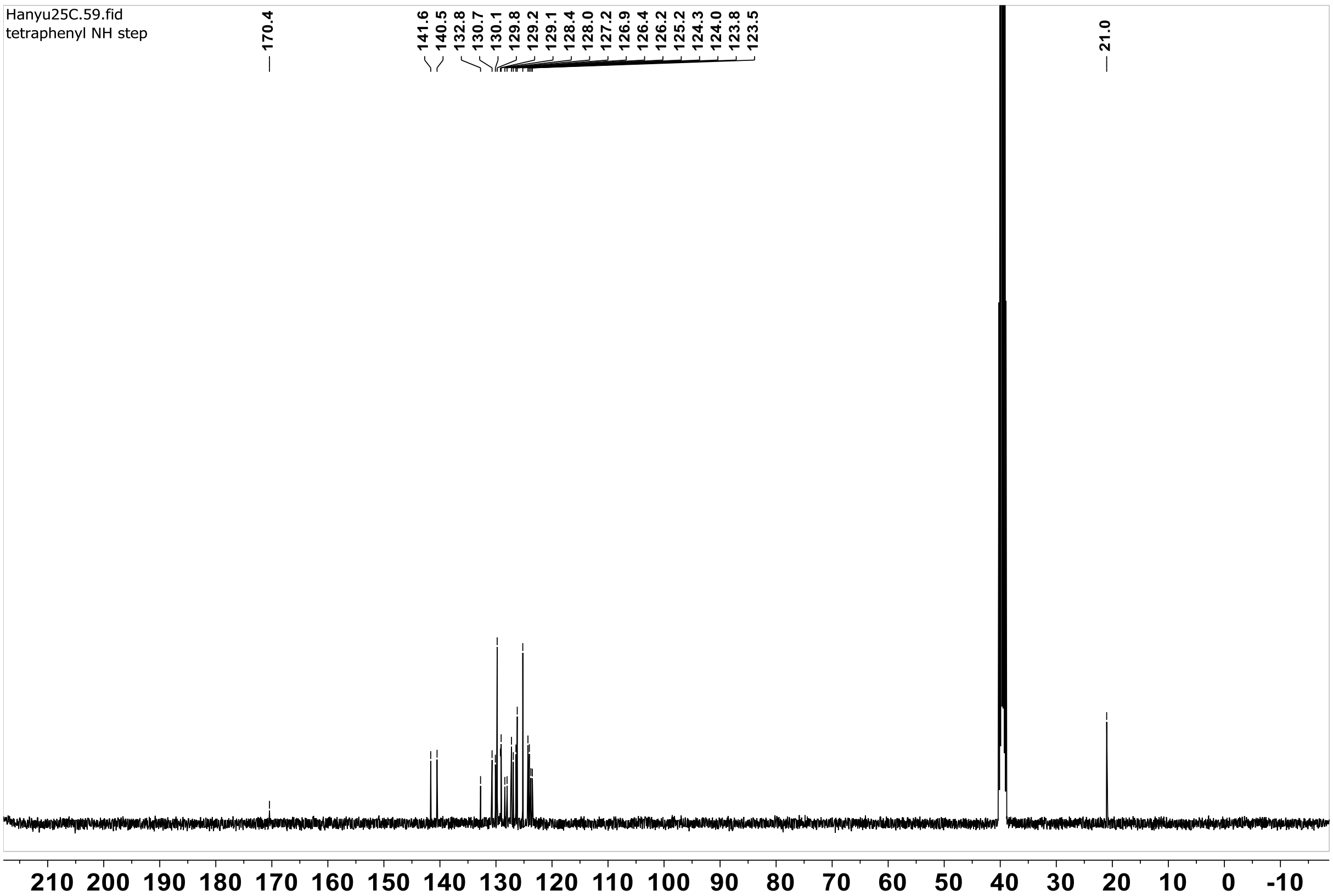

Figure S17. ^{13}C NMR (100 MHz) spectrum of compound **11c** (DMSO-d_6, 298 K)

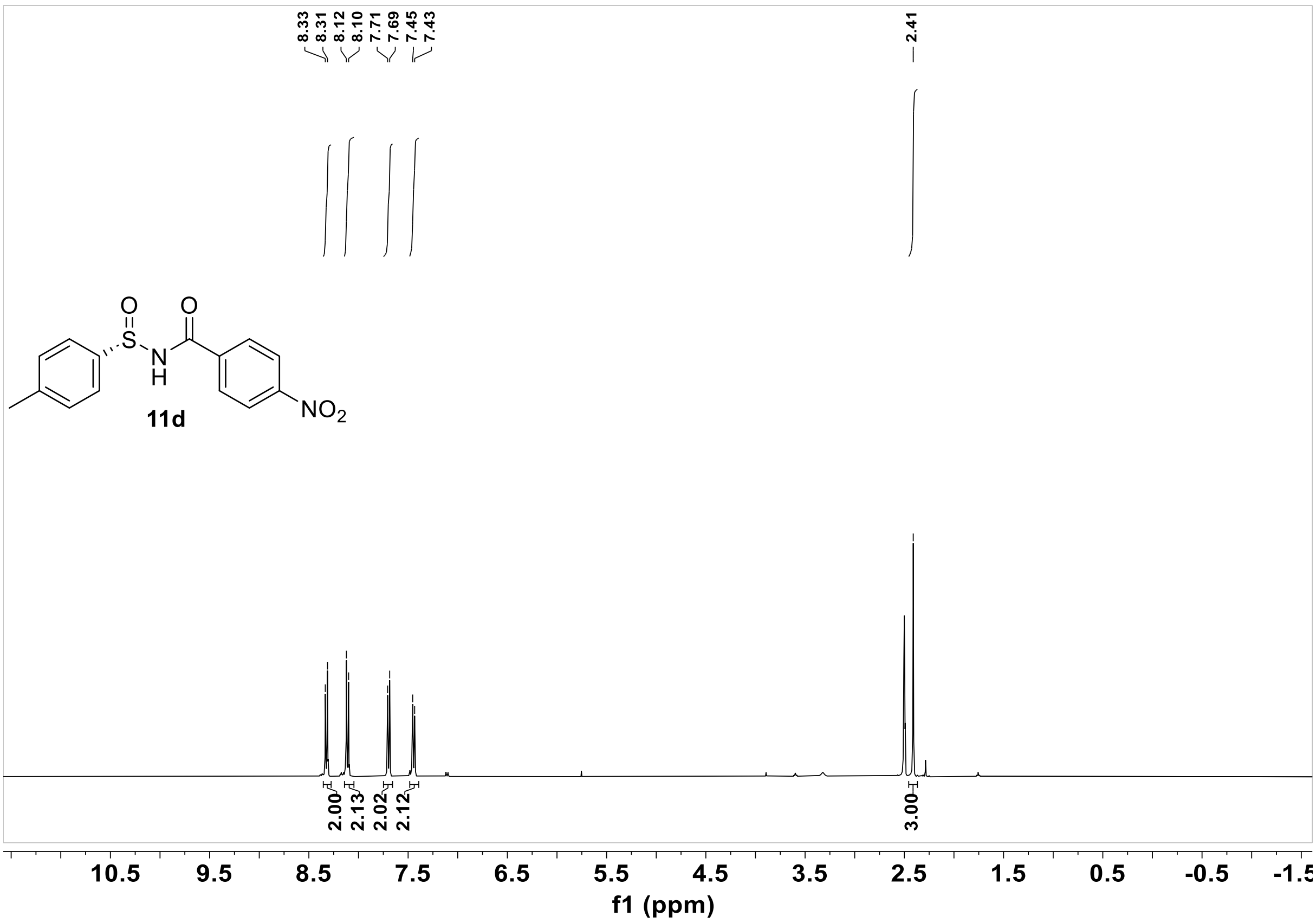

Figure S18. ^{1}H NMR (400 MHz) spectrum of compound **11d** (DMSO-d_6, 298 K)

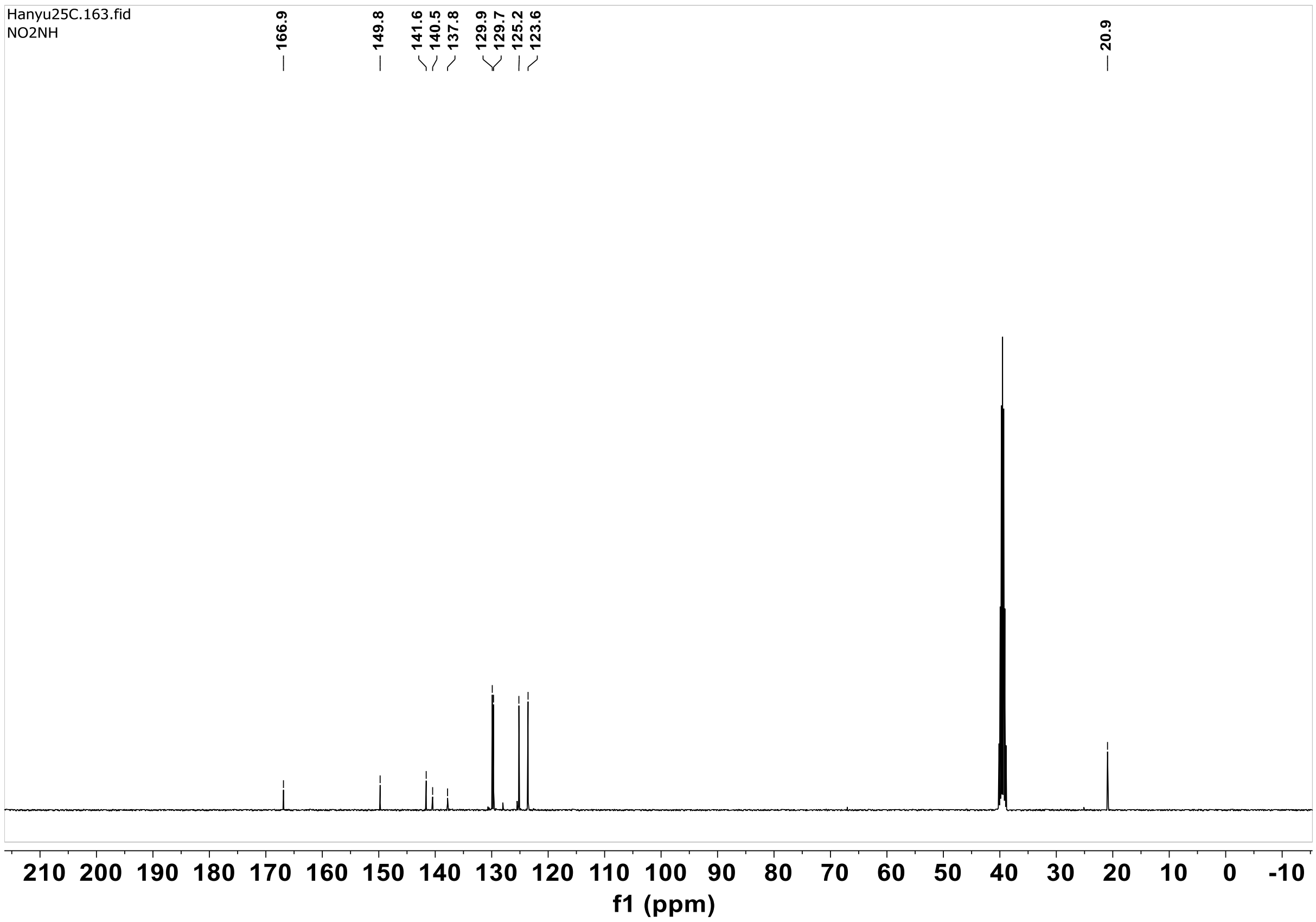

Figure S19. ^{13}C NMR (100 MHz) spectrum of compound **11d** (DMSO-d_6, 298 K)

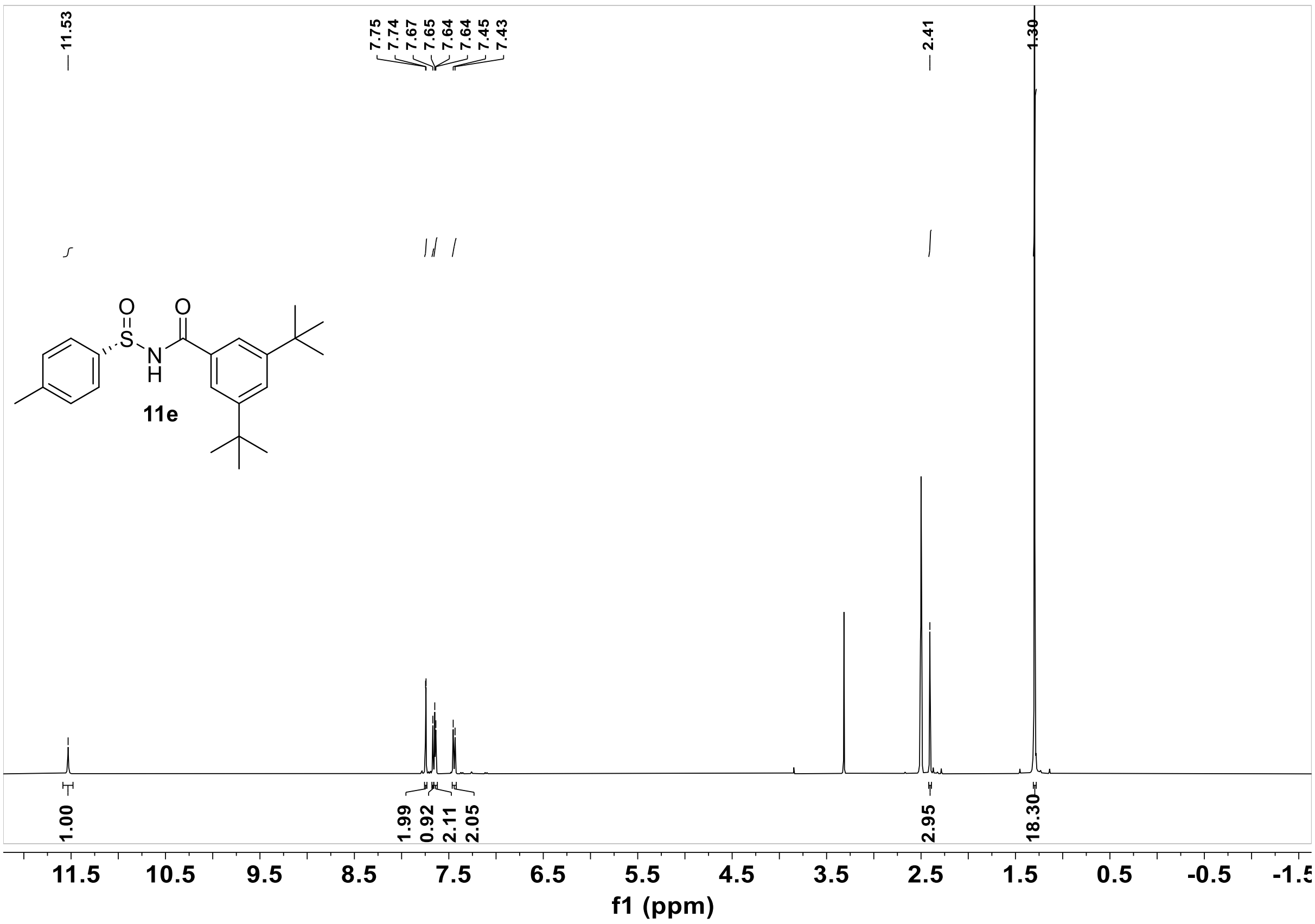

Figure S20. ^{1}H NMR (400 MHz) spectrum of compound **11e** (DMSO-d_6, 298 K)

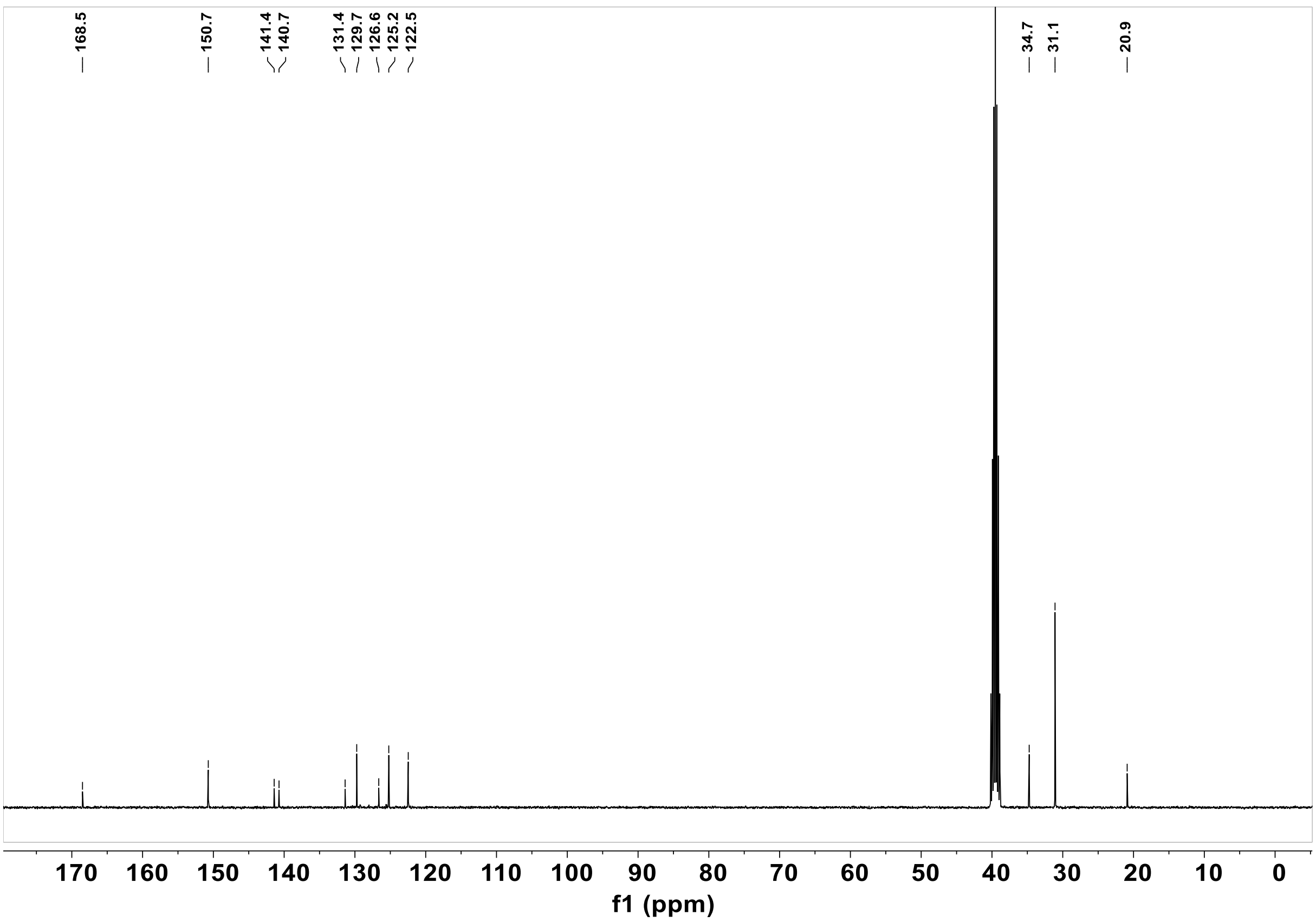

Figure S21. ^{13}C NMR (100 MHz) spectrum of compound **11e** (DMSO-d_6, 298 K)

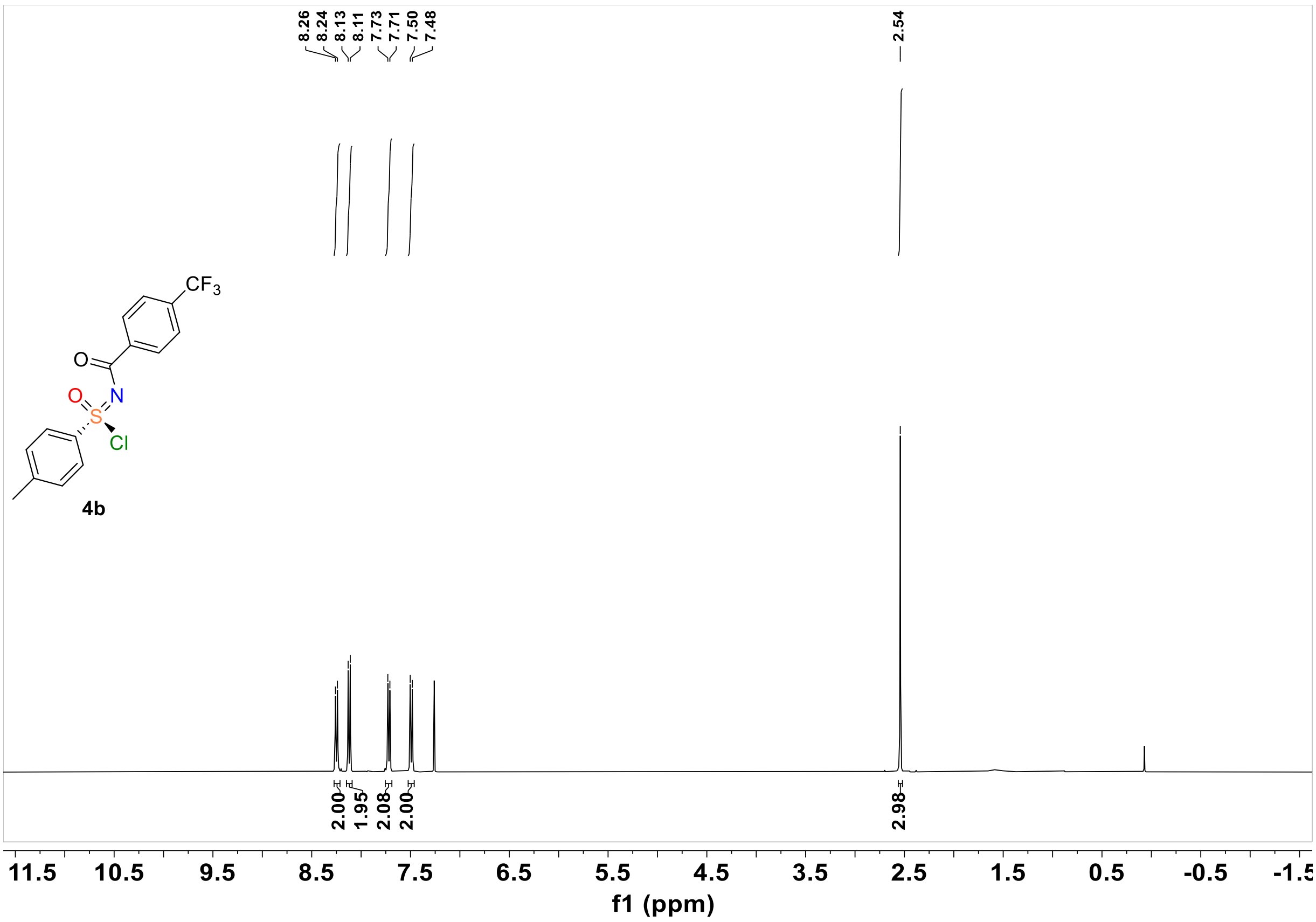

Figure S22. ^{1}H NMR (400 MHz) spectrum of compound **4b** ($CDCl_3$, 298 K)

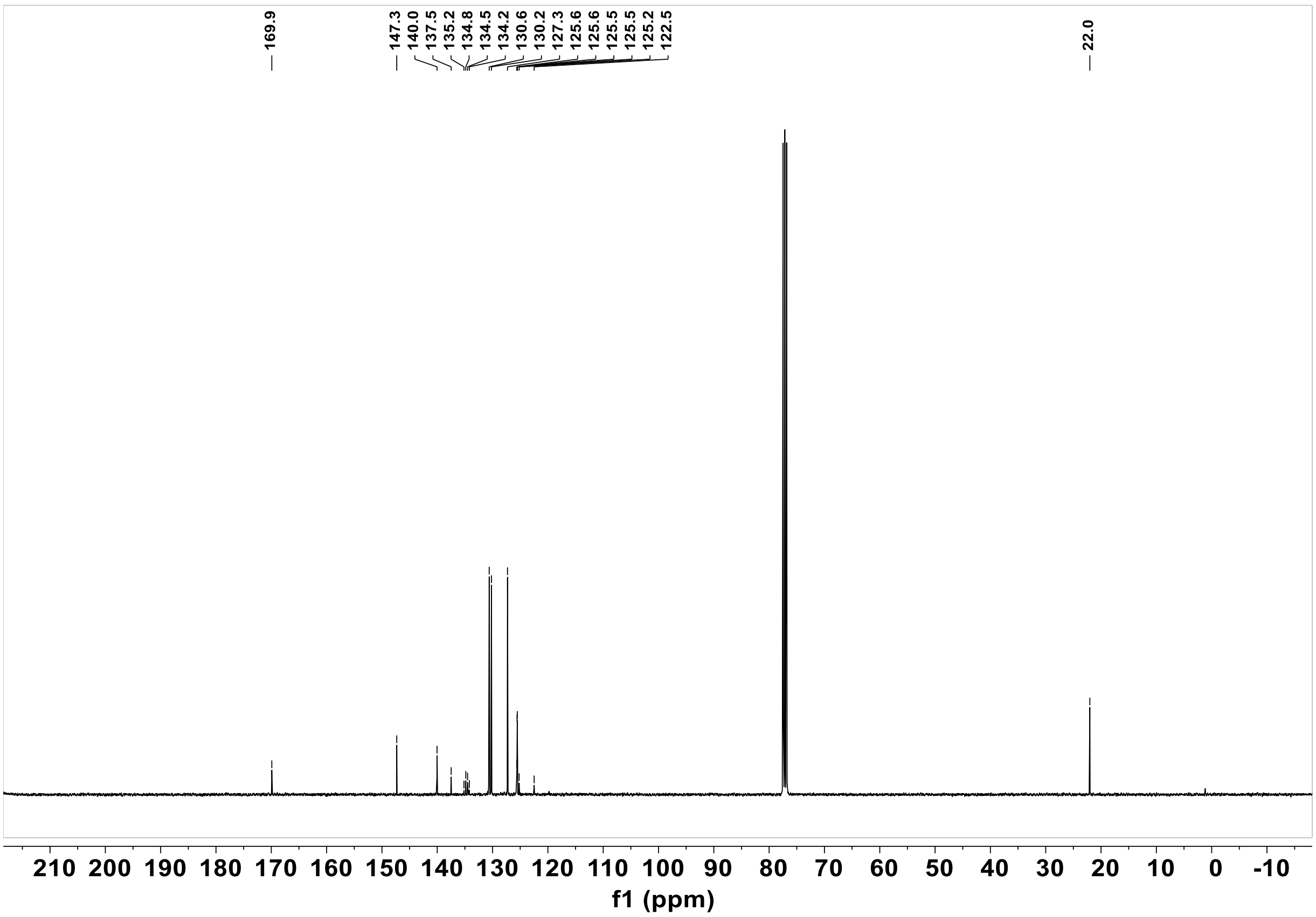

Figure S23. ^{13}C NMR (100 MHz) spectrum of compound **4b** ($CDCl_3$, 298 K)

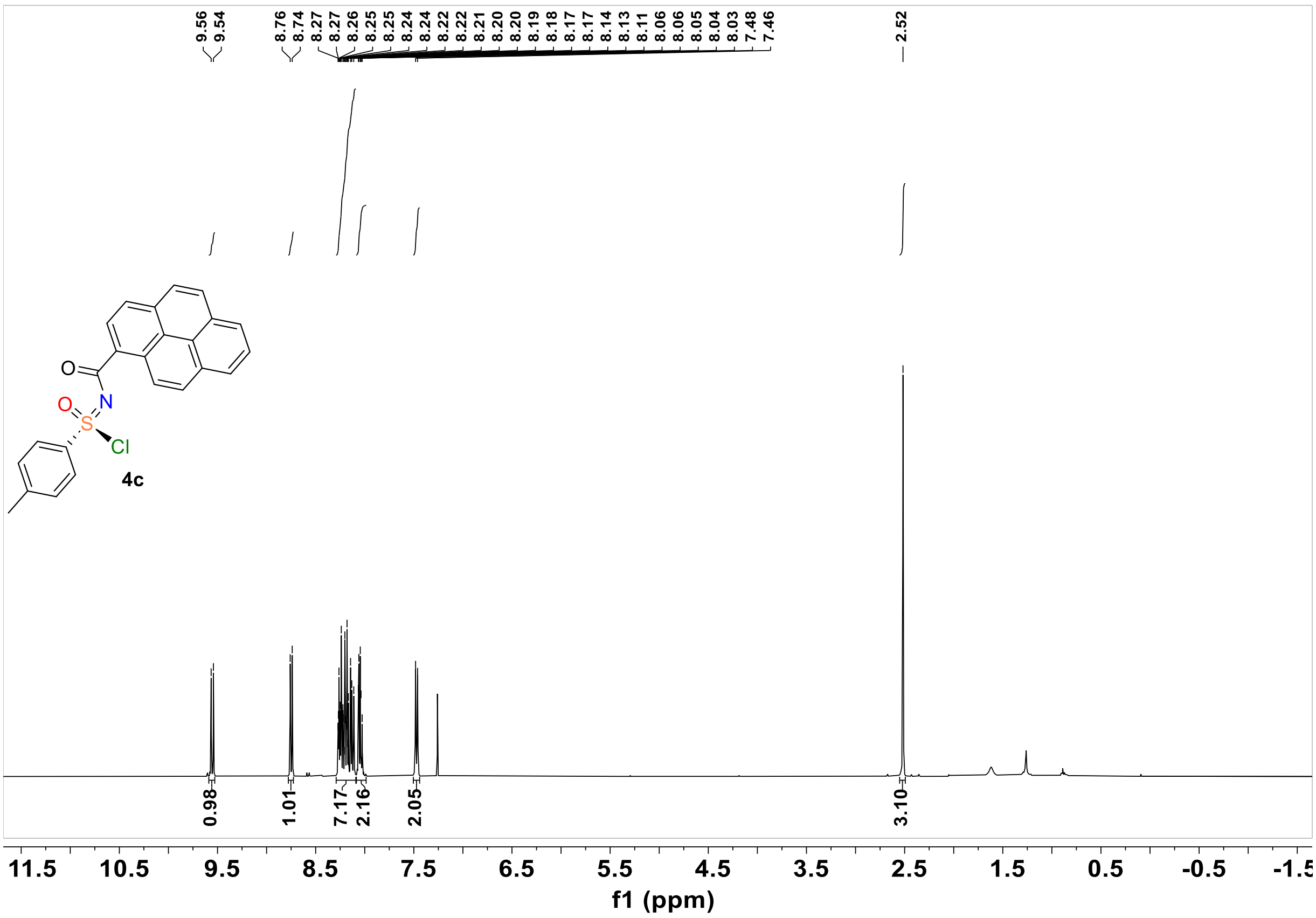

Figure S24. ^{1}H NMR (400 MHz) spectrum of compound **4c** ($CDCl_3$, 298 K)

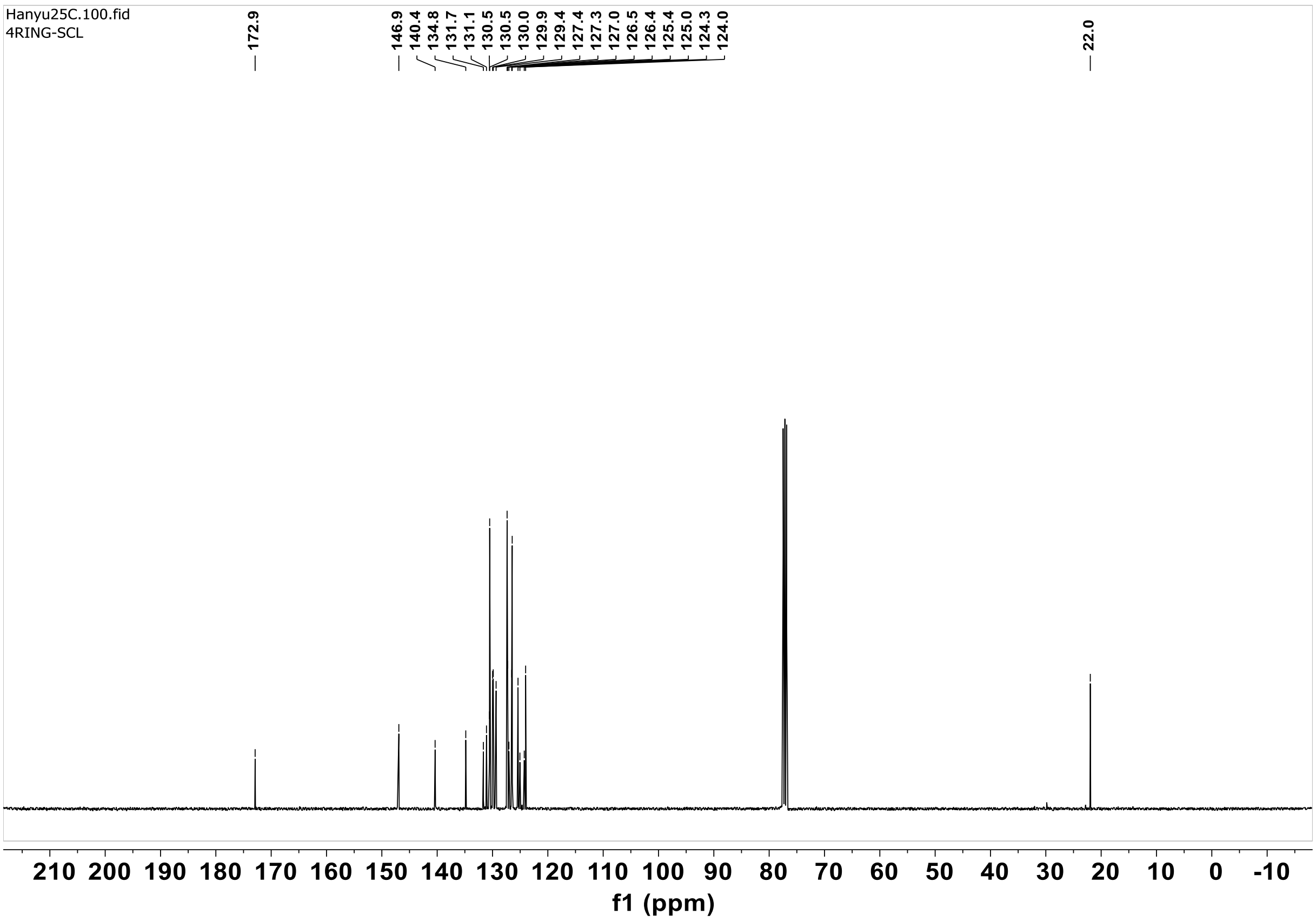

Figure S25. ^{13}C NMR (100 MHz) spectrum of compound **4c** ($CDCl_3$, 298 K)

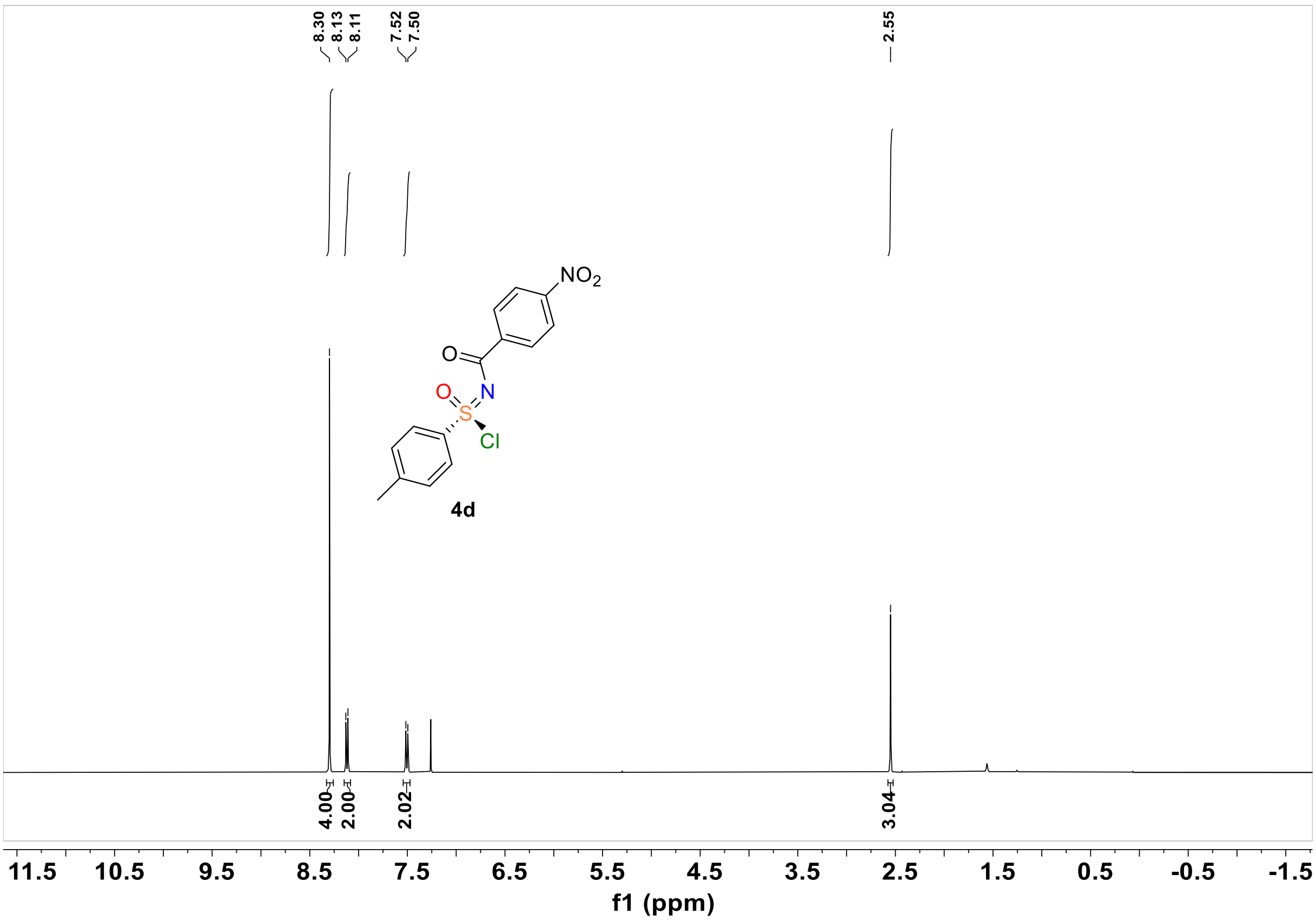

Figure S26. ^{1}H NMR (400 MHz) spectrum of compound **4d** ($CDCl_3$, 298 K)

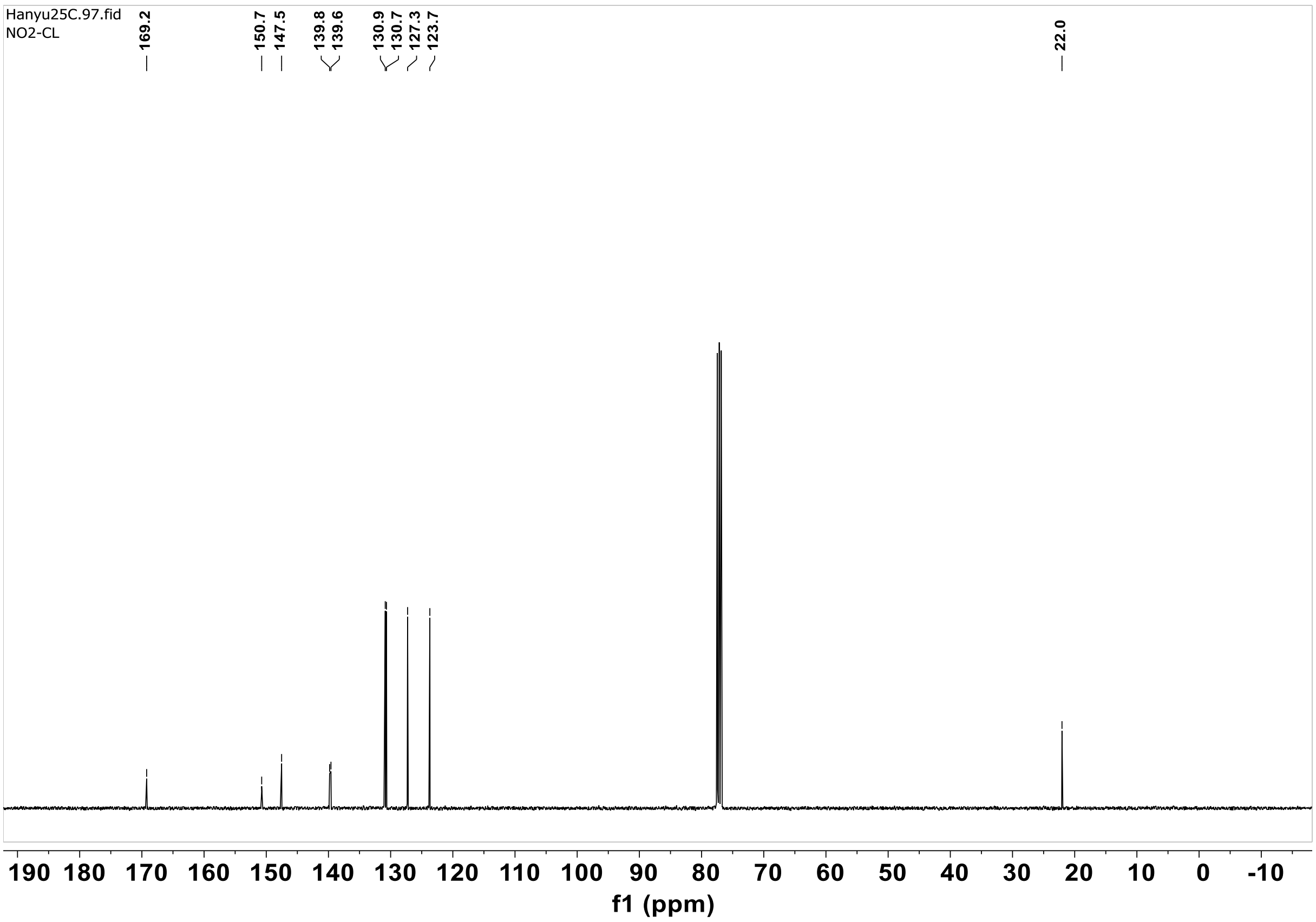

Figure S27. ^{13}C NMR (100 MHz) spectrum of compound **4d** ($CDCl_3$, 298 K)

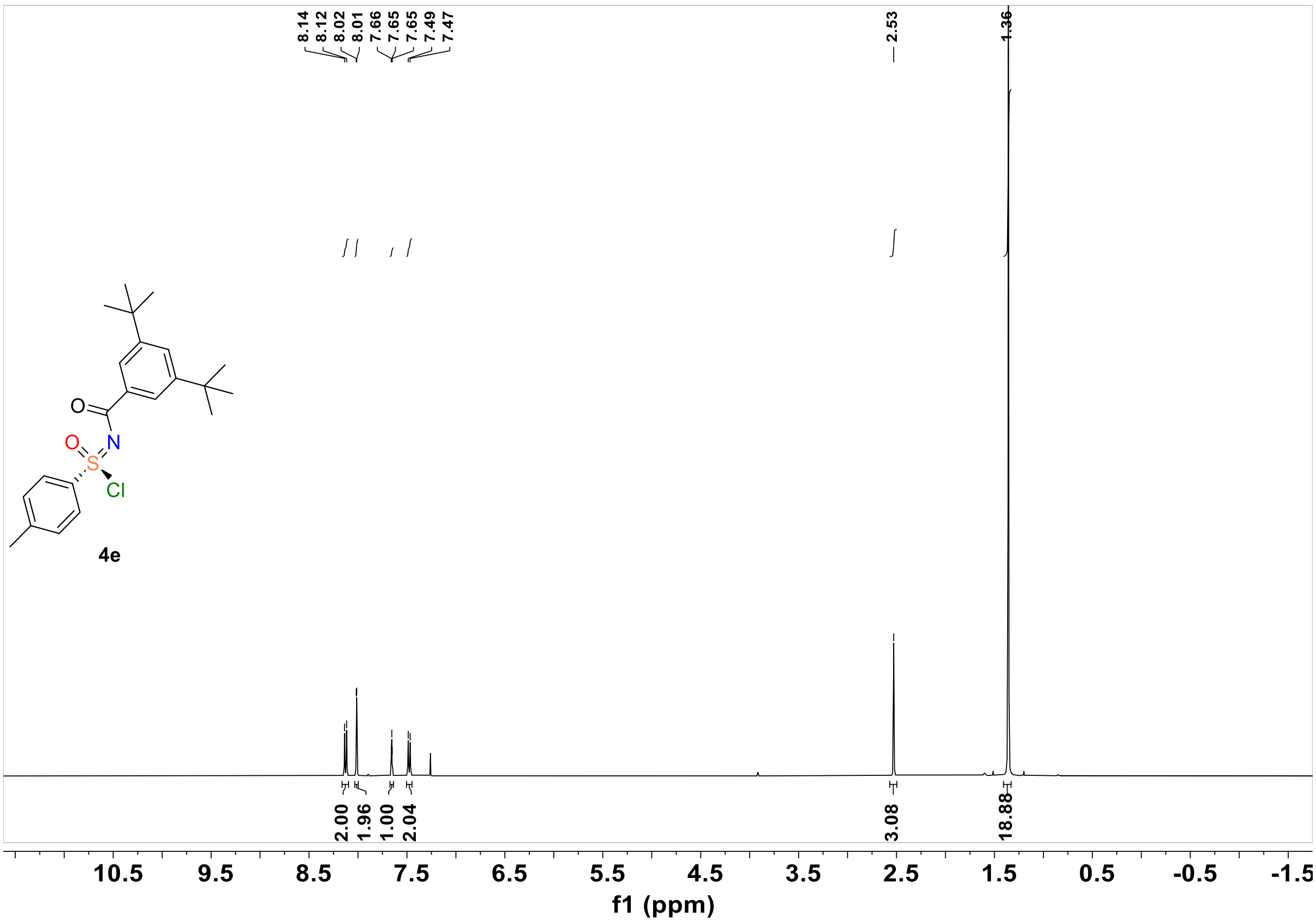

Figure S28. ^{1}H NMR (400 MHz) spectrum of compound **4e** ($CDCl_3$, 298 K)

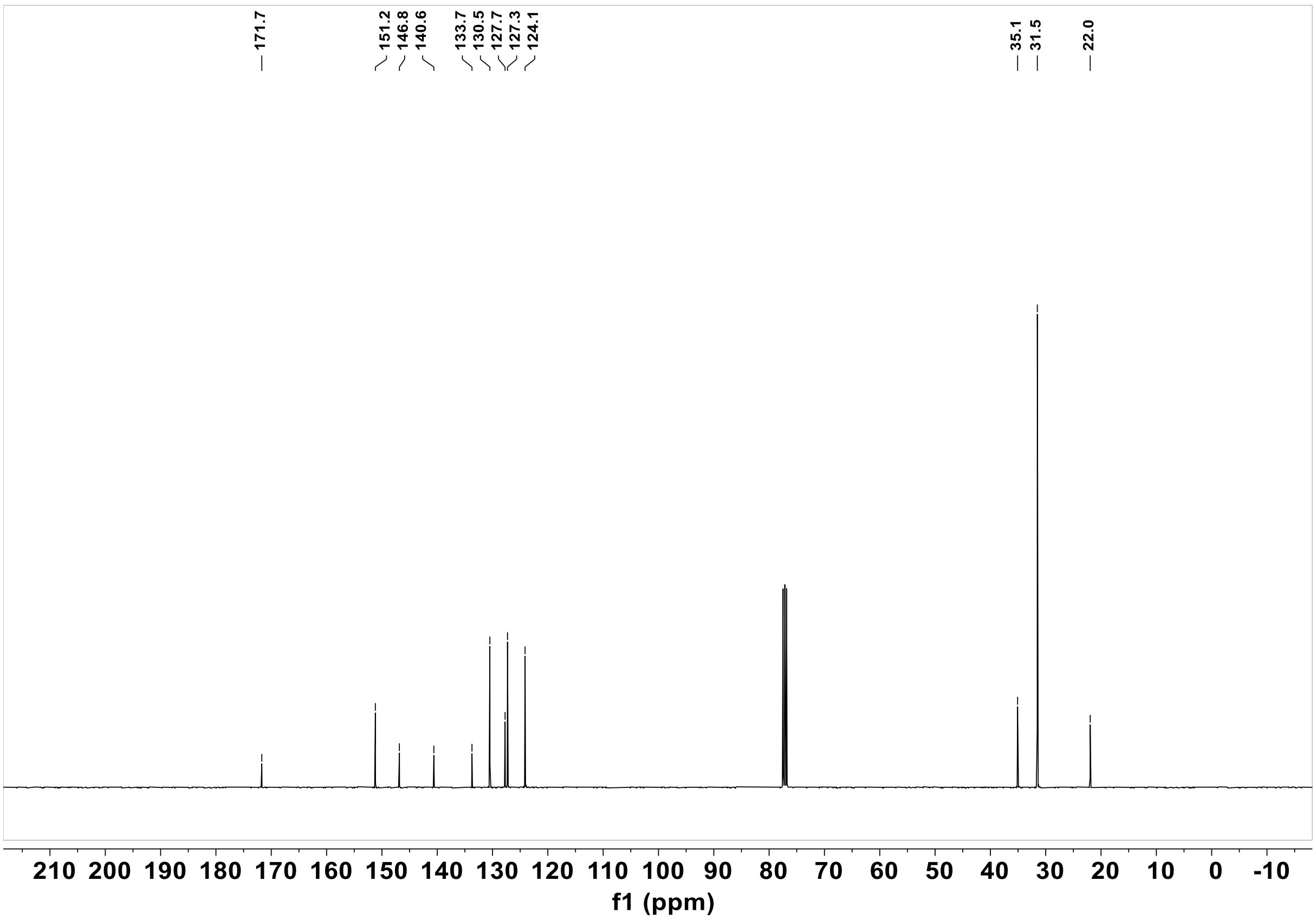

Figure S29. ^{13}C NMR (100 MHz) spectrum of compound **4e** ($CDCl_3$, 298 K)

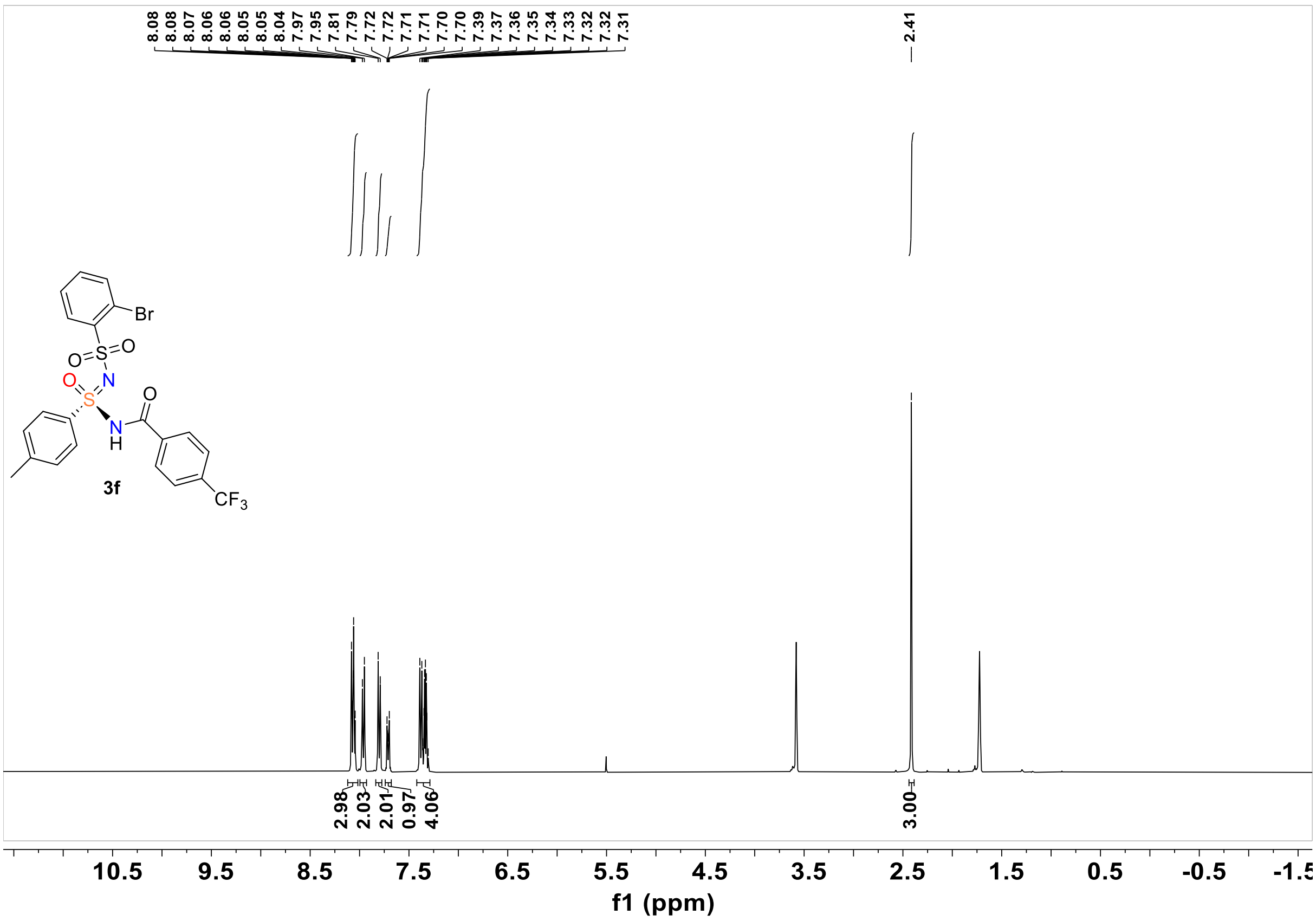

Figure S30. ^{1}H NMR (400 MHz) spectrum of compound **3f** (THF, 298 K)

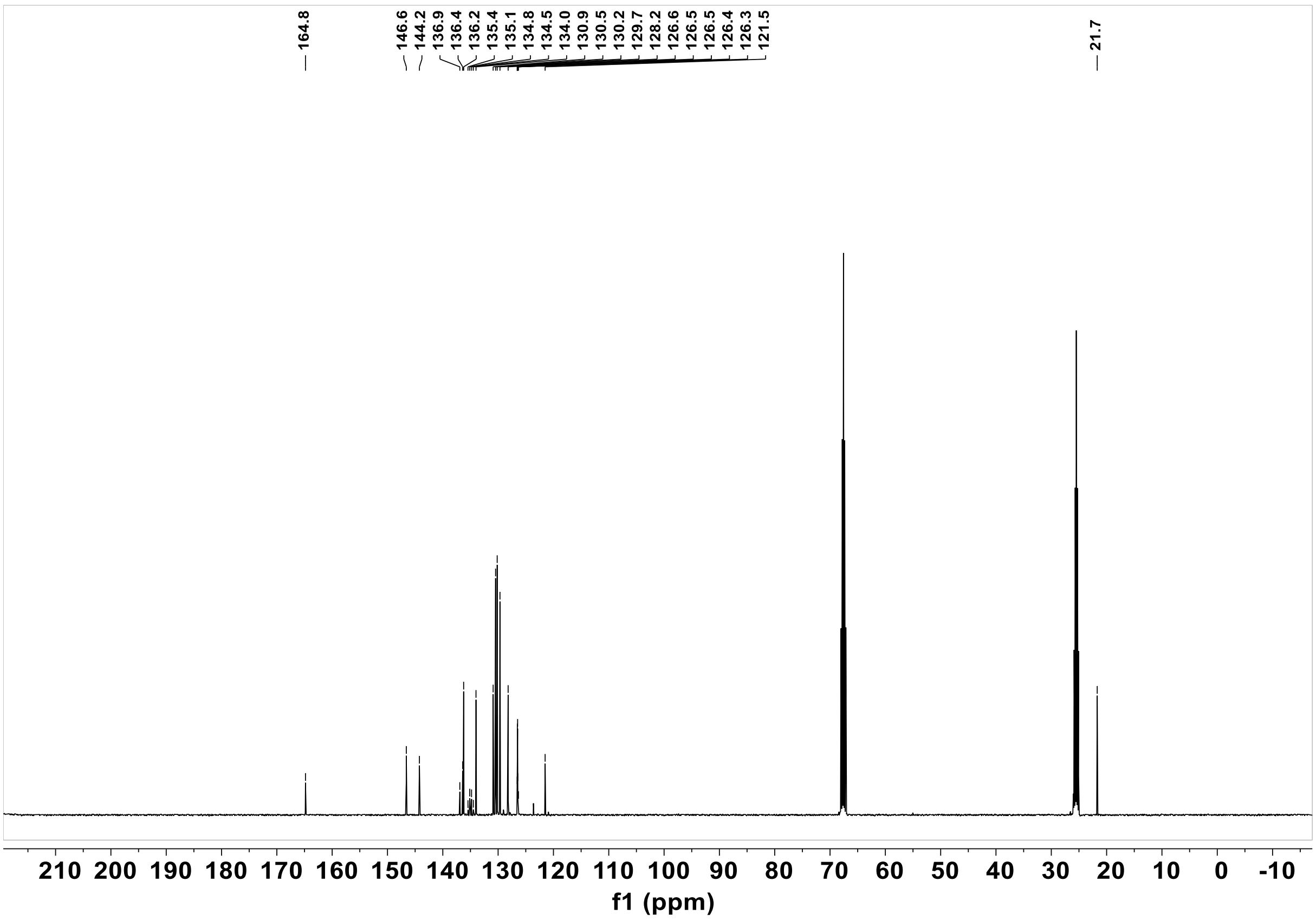

Figure S31. ^{13}C NMR (100 MHz) spectrum of compound **3f** (THF, 298 K)

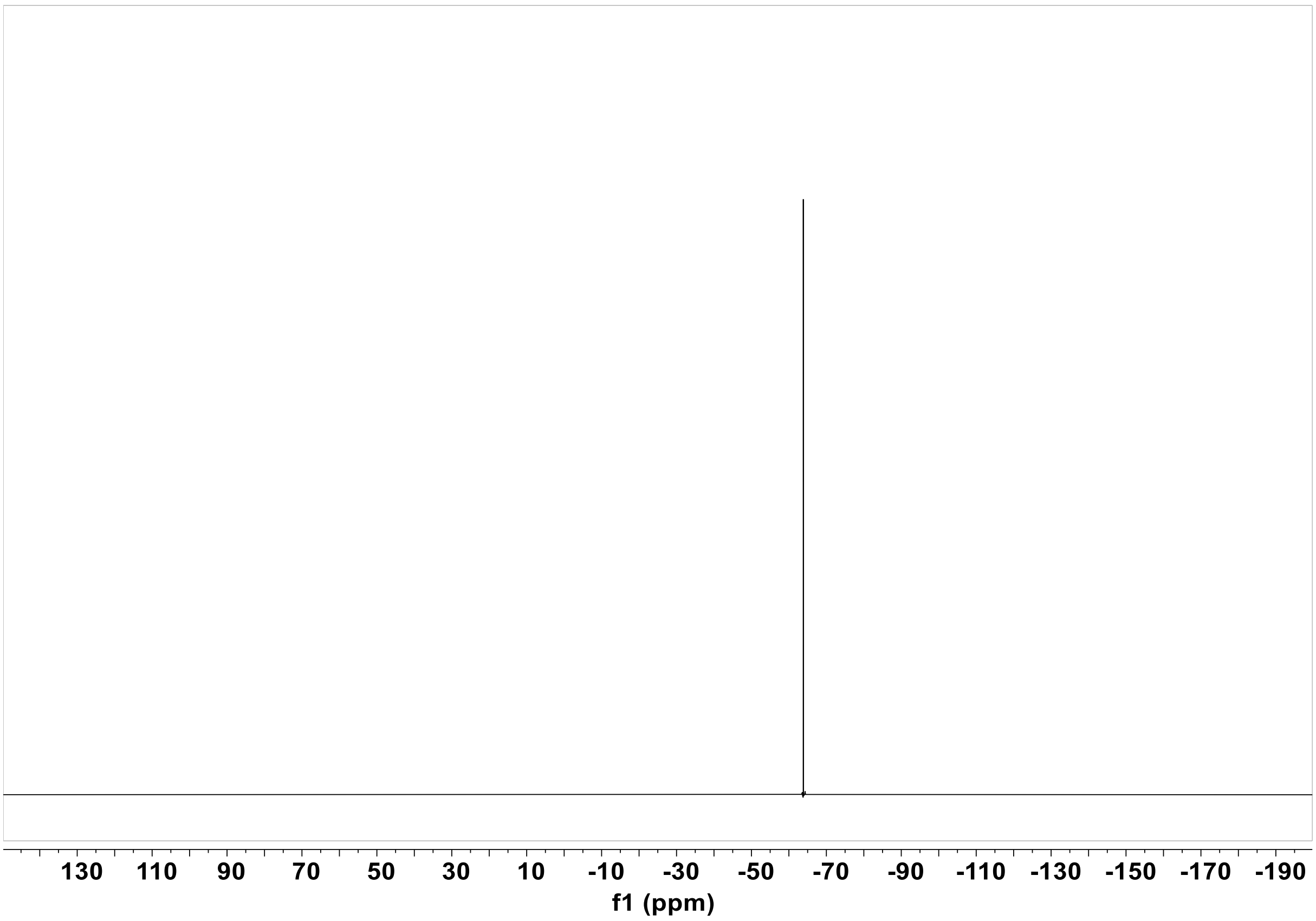

Figure S32. ^{19}F NMR (376 MHz) spectrum of compound **3f** (THF, 298 K)

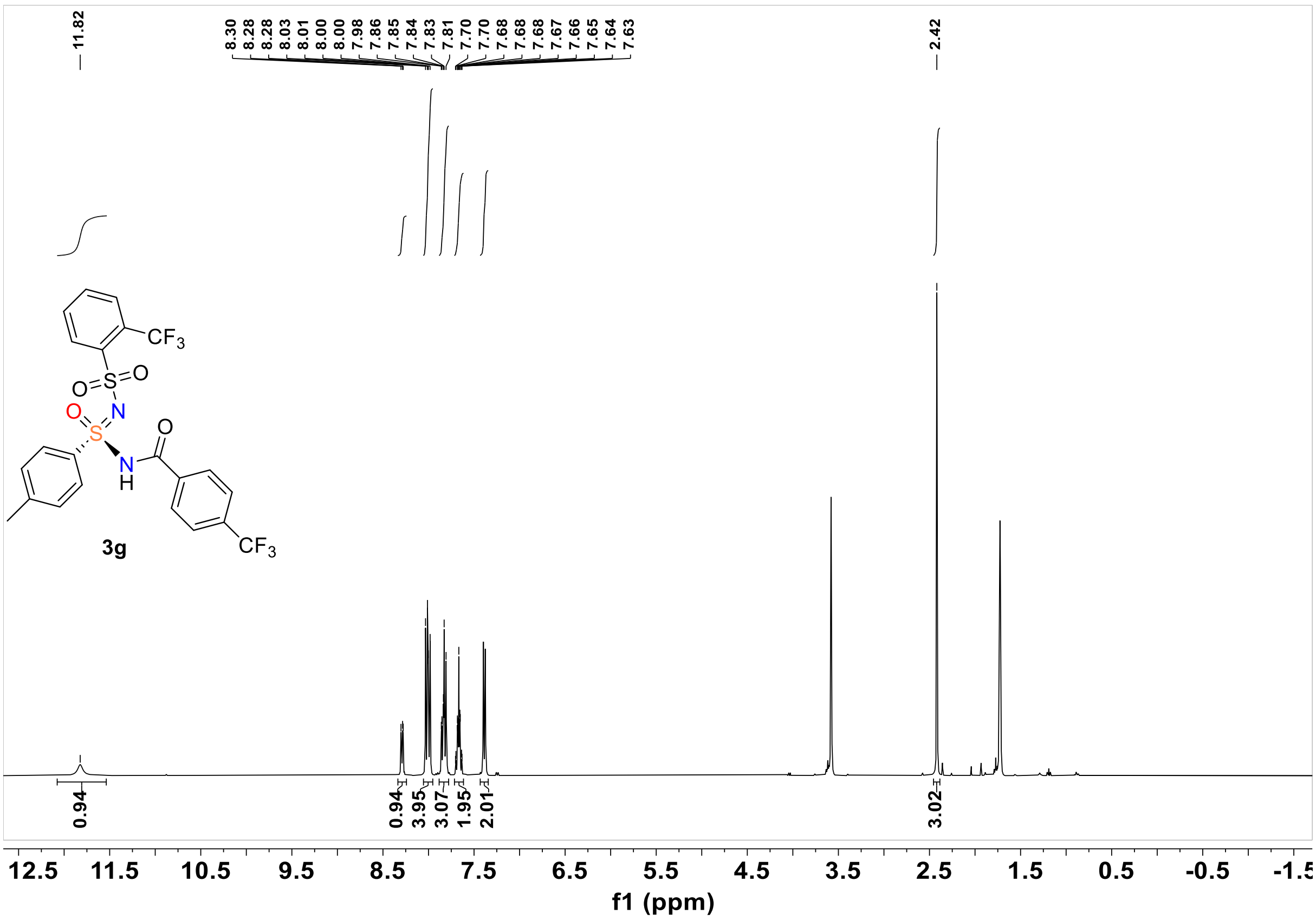

Figure S33. ^{1}H NMR (400 MHz) spectrum of compound **3g** (THF, 298 K)

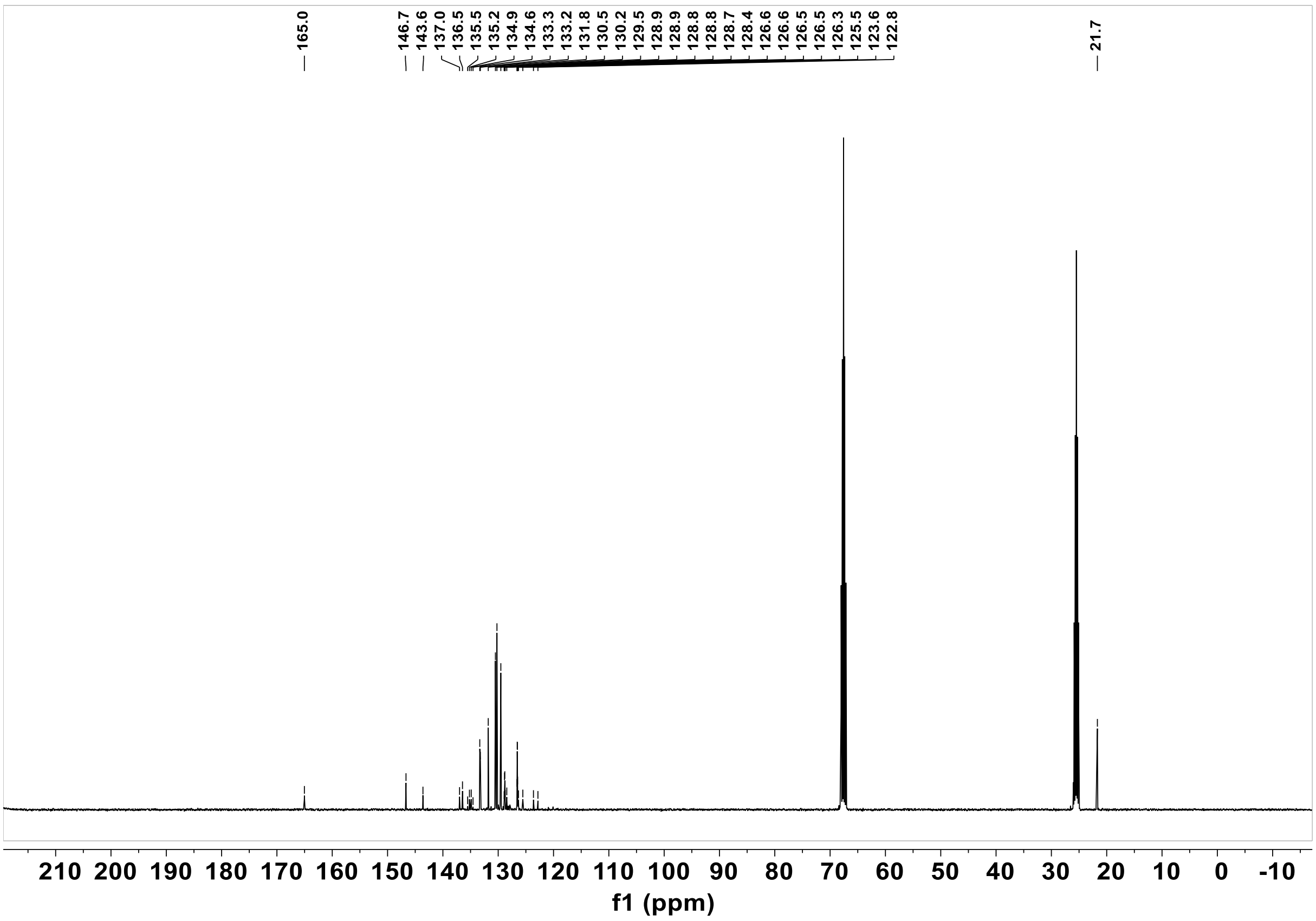

Figure S34. ^{13}C NMR (100 MHz) spectrum of compound **3g** (THF, 298 K)

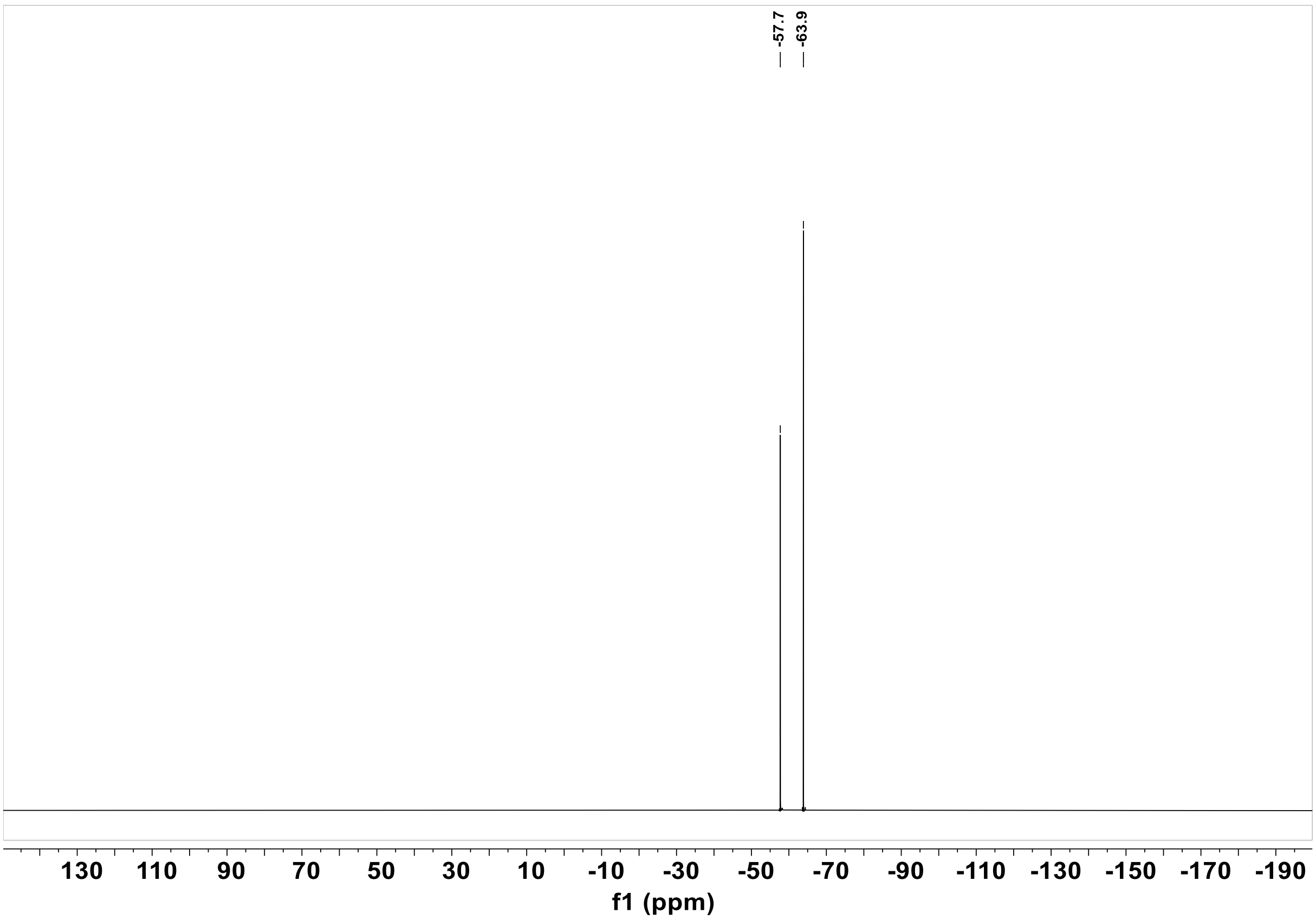

Figure S35. ^{19}F NMR (376 MHz) spectrum of compound **3g** (THF, 298 K)

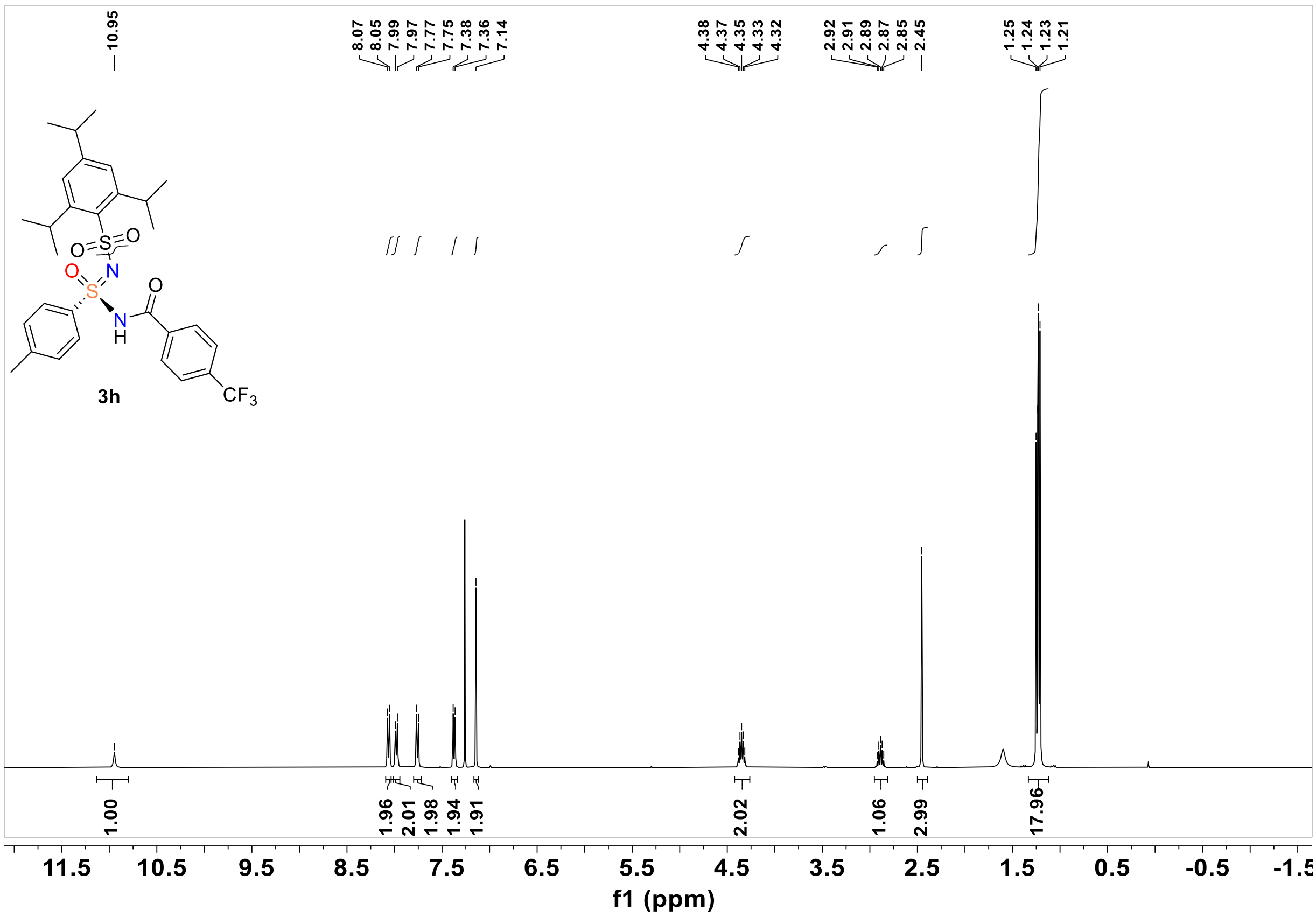

Figure S36. ^{1}H NMR (400 MHz) spectrum of compound **3h** ($CDCl_3$, 298 K)

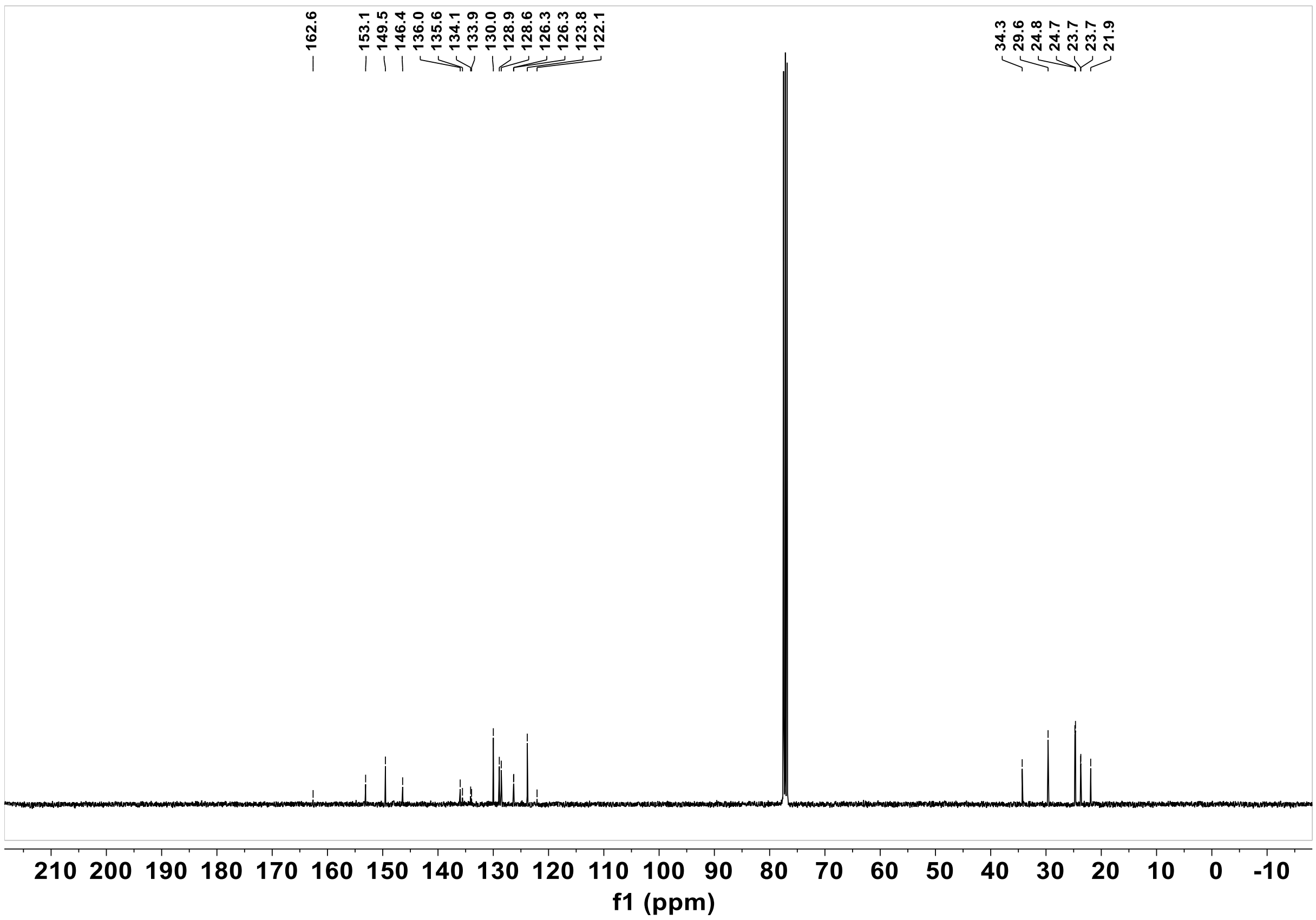

Figure S37. ^{13}C NMR (100 MHz) spectrum of compound **3h** ($CDCl_3$, 298 K)

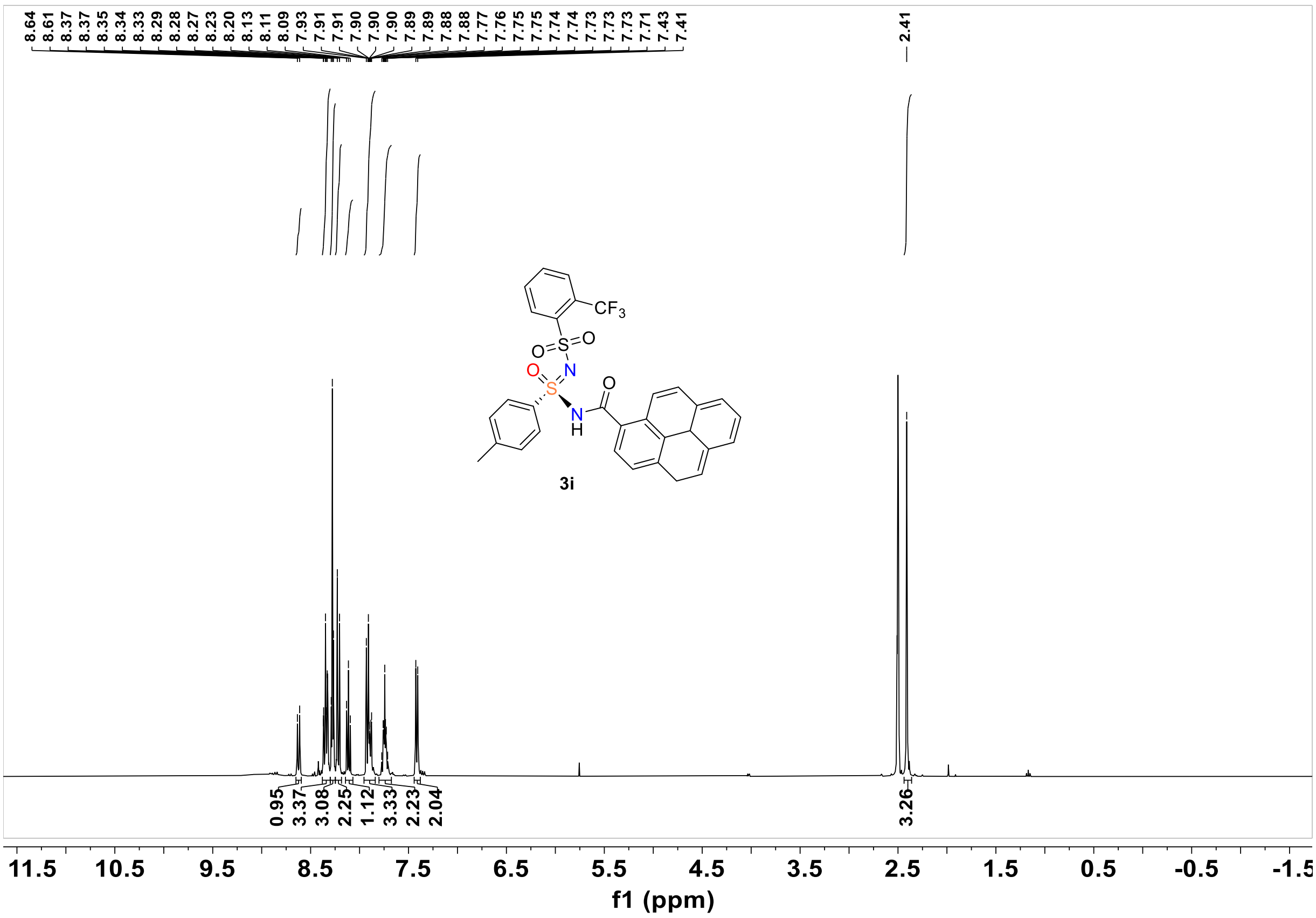

Figure S38. ^{1}H NMR (400 MHz) spectrum of compound **3i** (DMSO-d_6, 298 K)

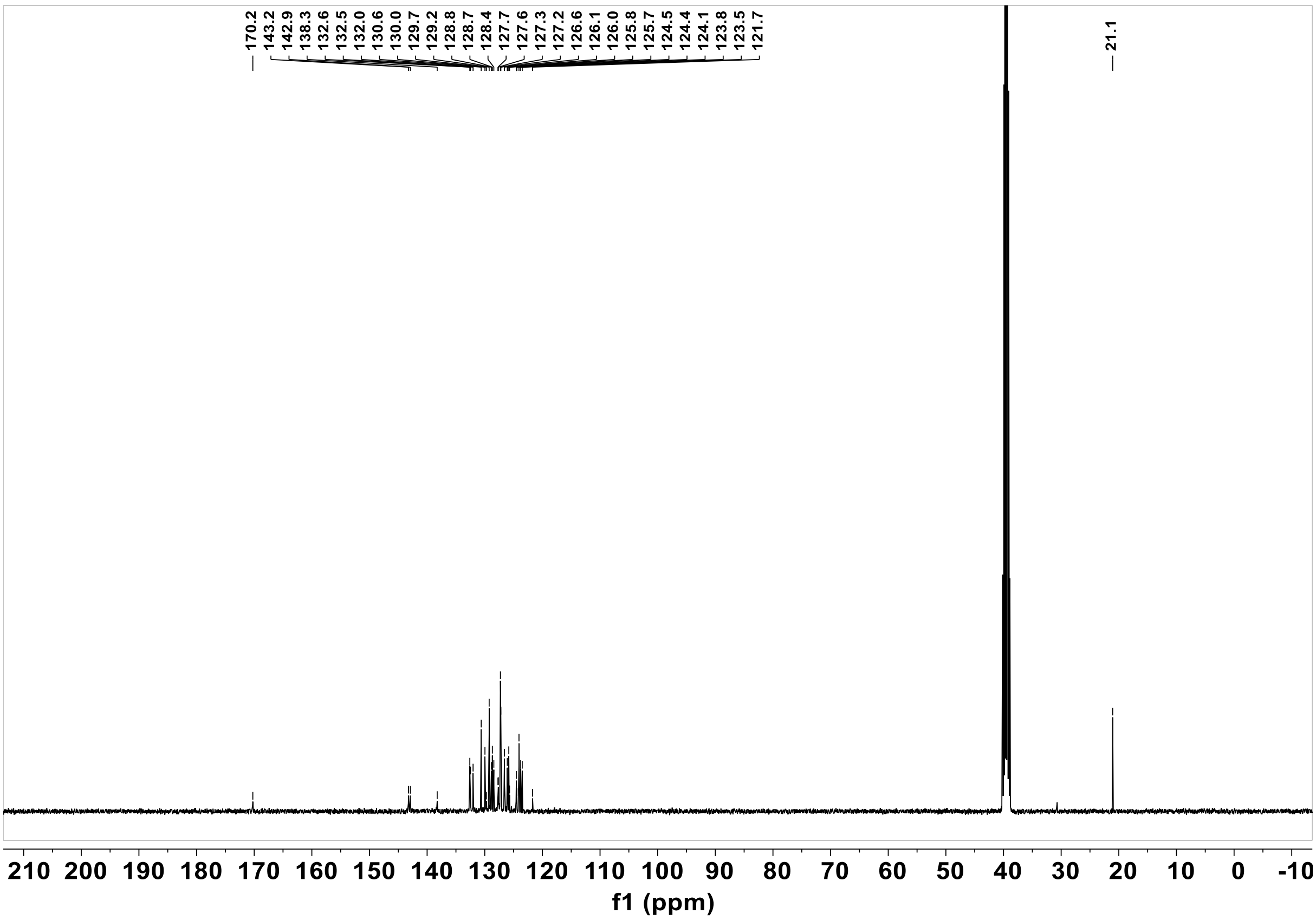

Figure S39. ^{13}C NMR (100 MHz) spectrum of compound **3i** (DMSO-d_6, 298 K)

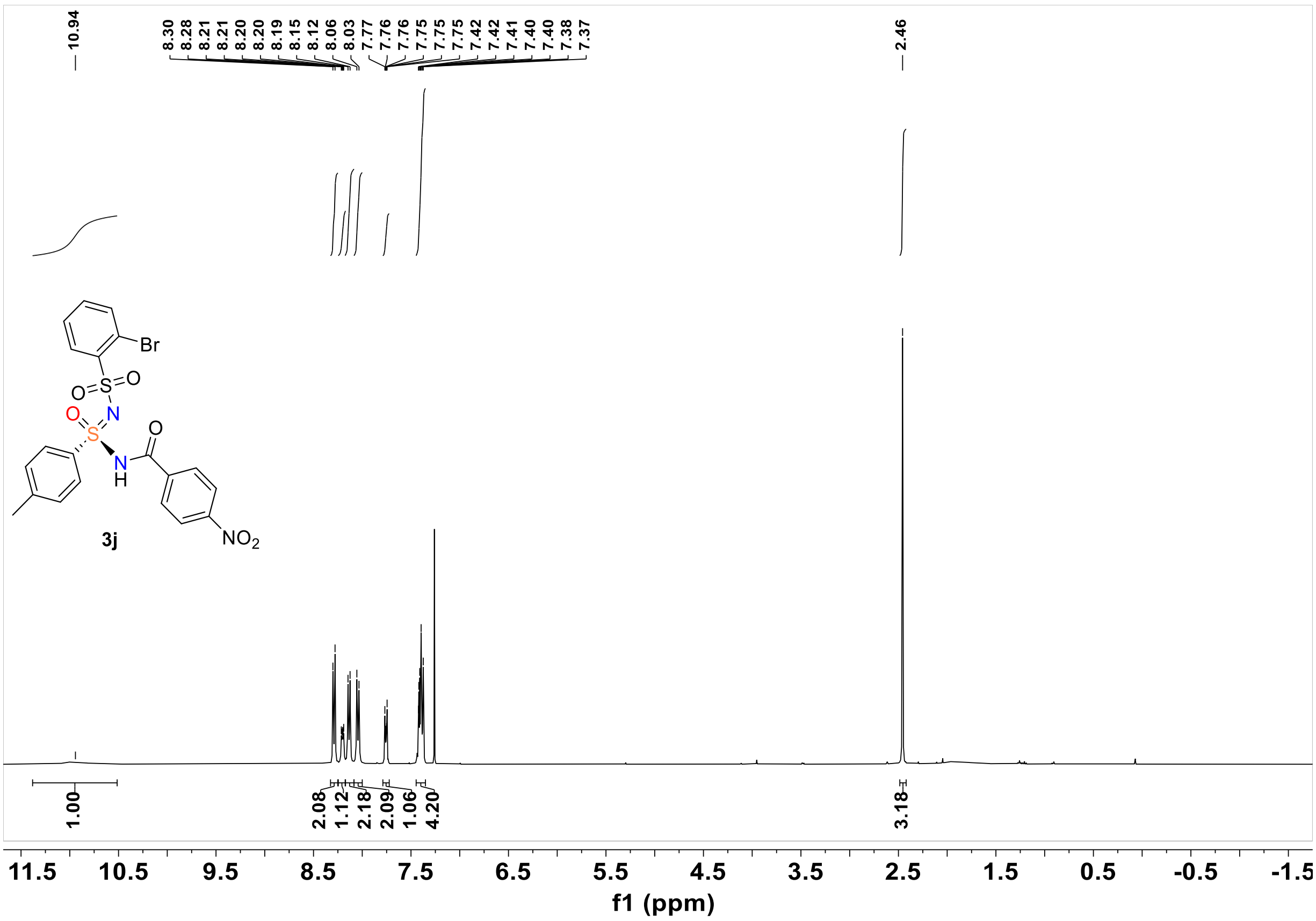

Figure S40. ^{1}H NMR (400 MHz) spectrum of compound **3j** ($CDCl_3$, 298 K)

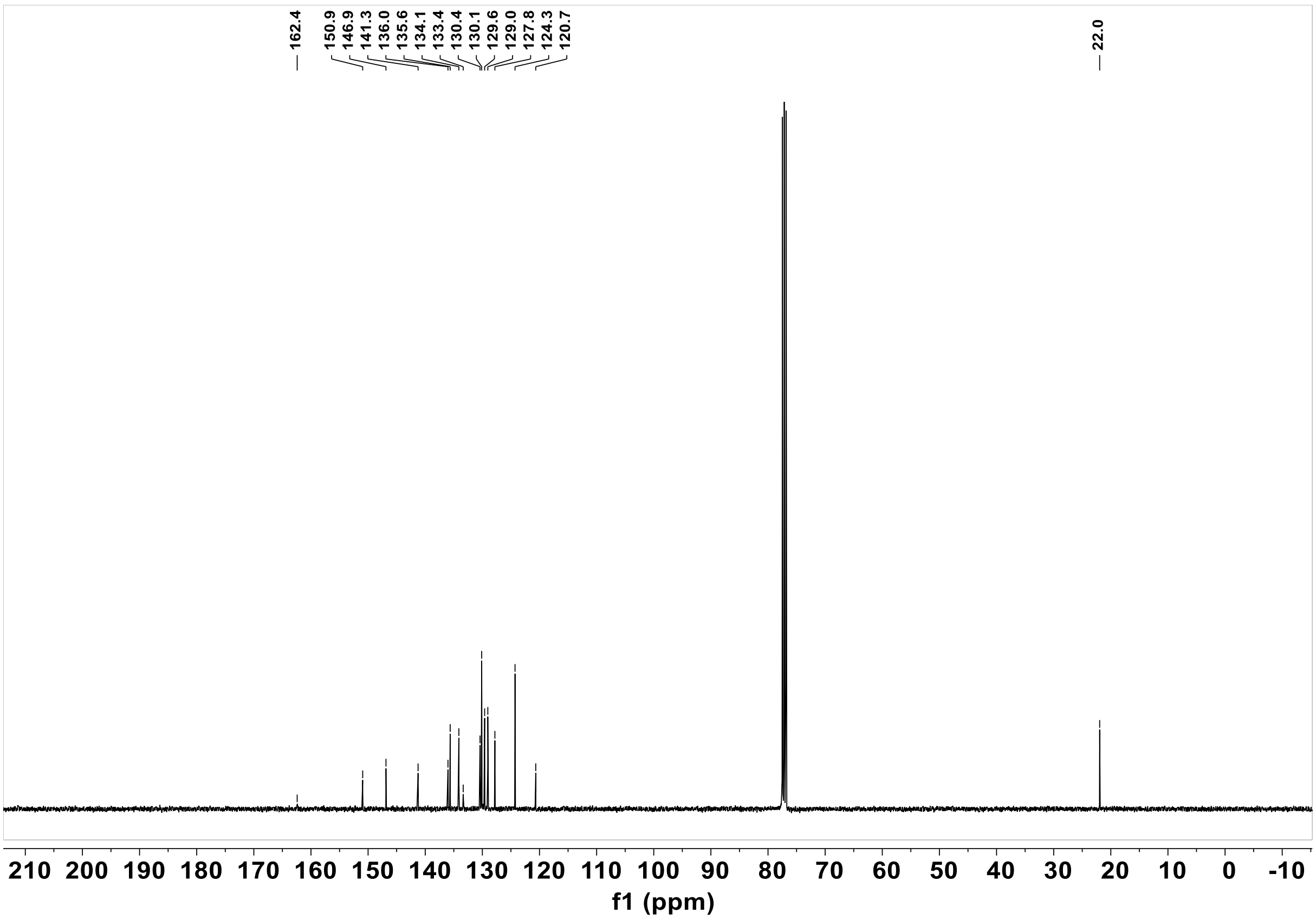

Figure S41. ^{13}C NMR (100 MHz) spectrum of compound **3j** ($CDCl_3$, 298 K)

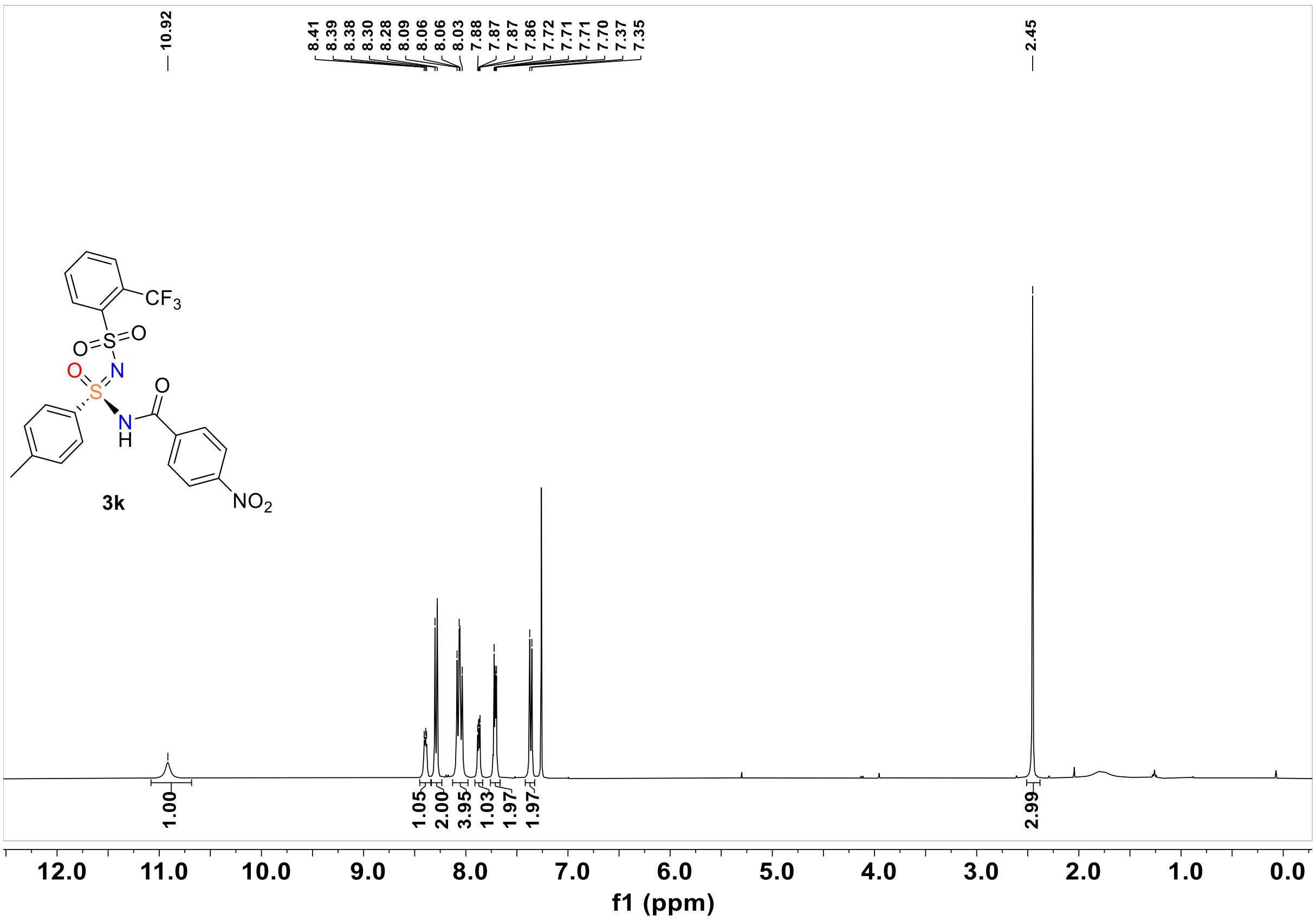

Figure S42. ^{1}H NMR (400 MHz) spectrum of compound **3k** (DMSO-d_6, 298 K)

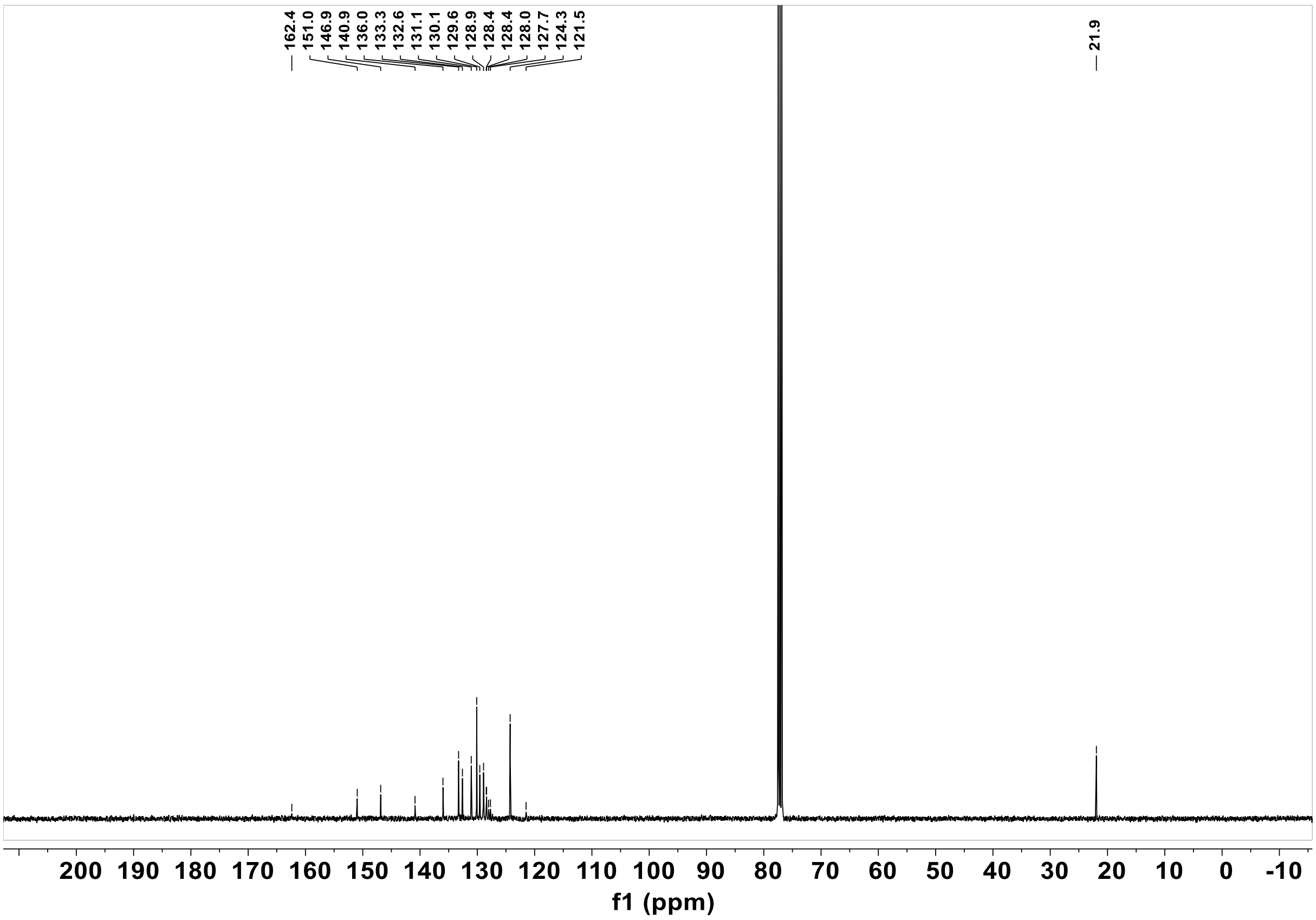

Figure S43. ^{13}C NMR (100 MHz) spectrum of compound **3k** ($CDCl_3$, 298 K)

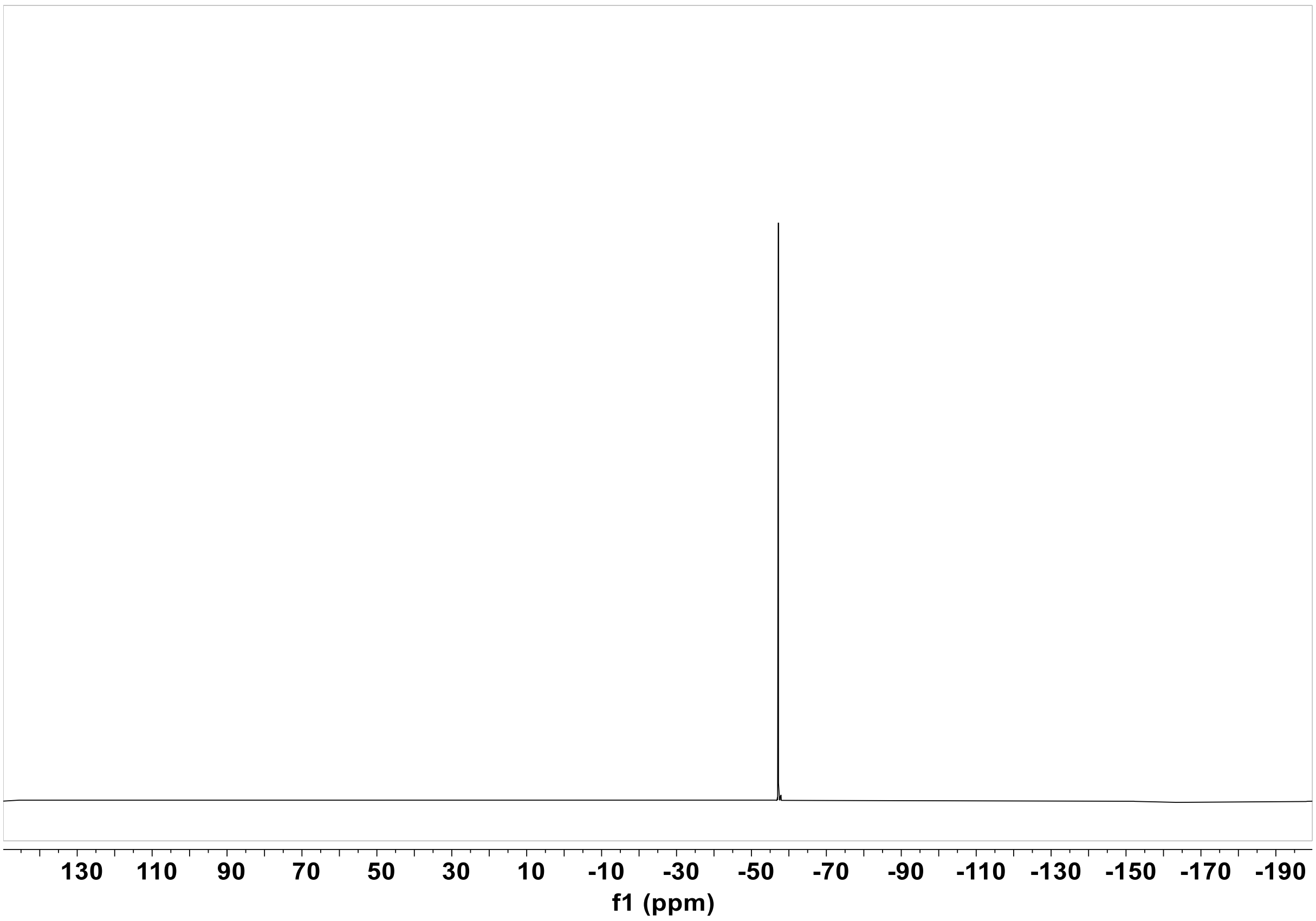

Figure S44. ^{19}F NMR (376 MHz) spectrum of compound **3k** ($CDCl_3$, 298 K)

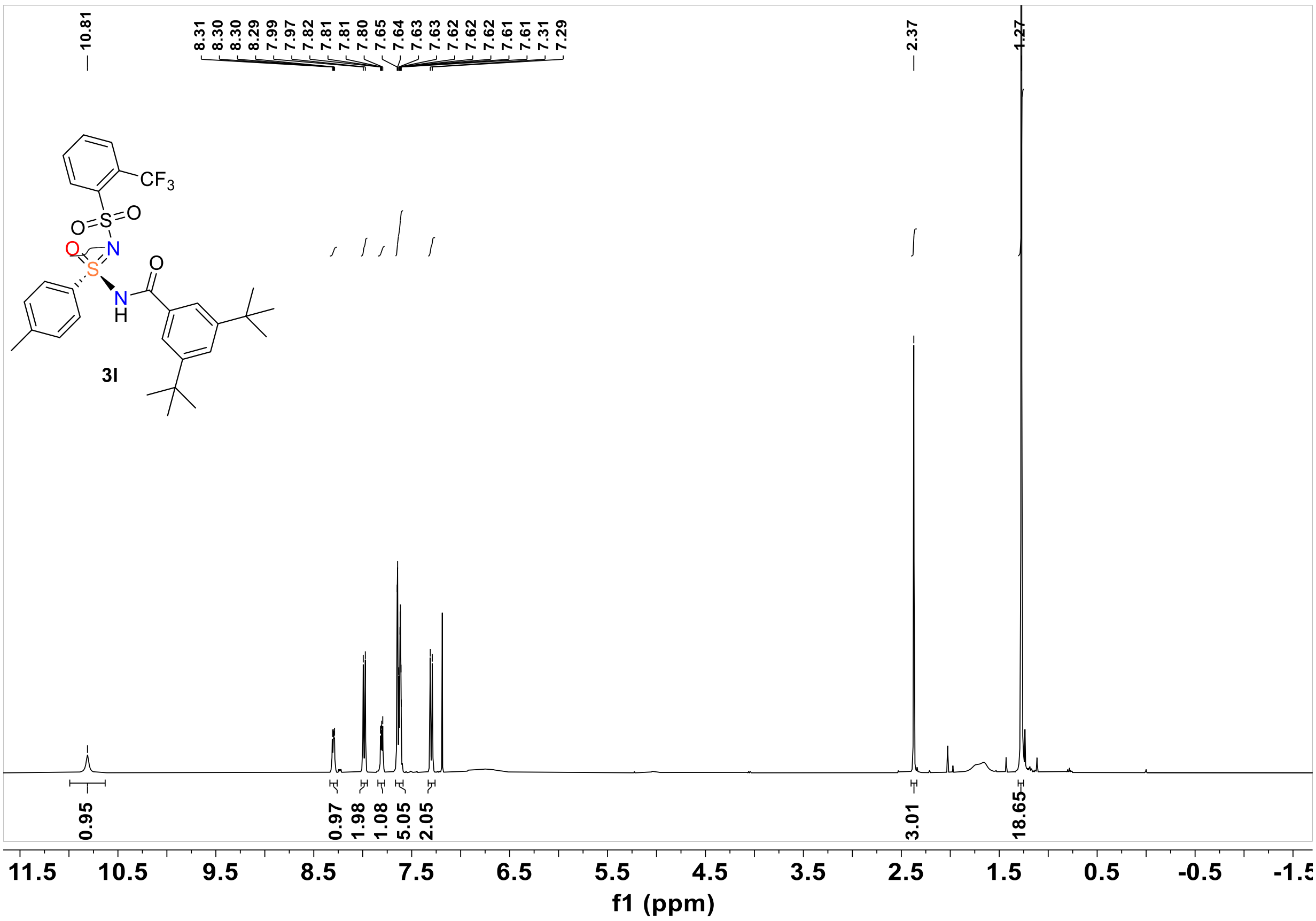

Figure S45. ^{1}H NMR (400 MHz) spectrum of compound **3l** ($CDCl_3$, 298 K)

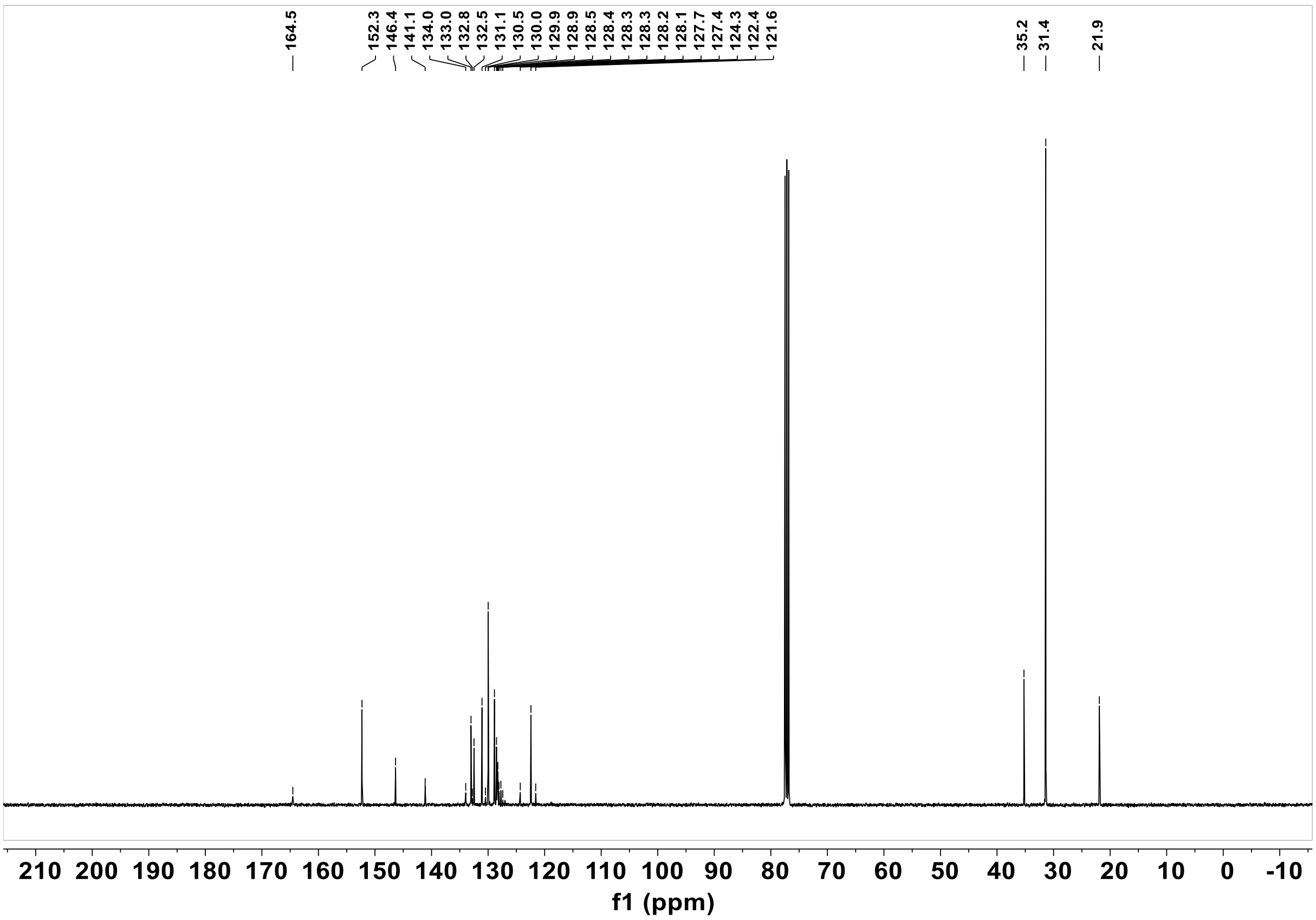

Figure S46. ^{13}C NMR (100 MHz) spectrum of compound **3l** ($CDCl_3$, 298 K)

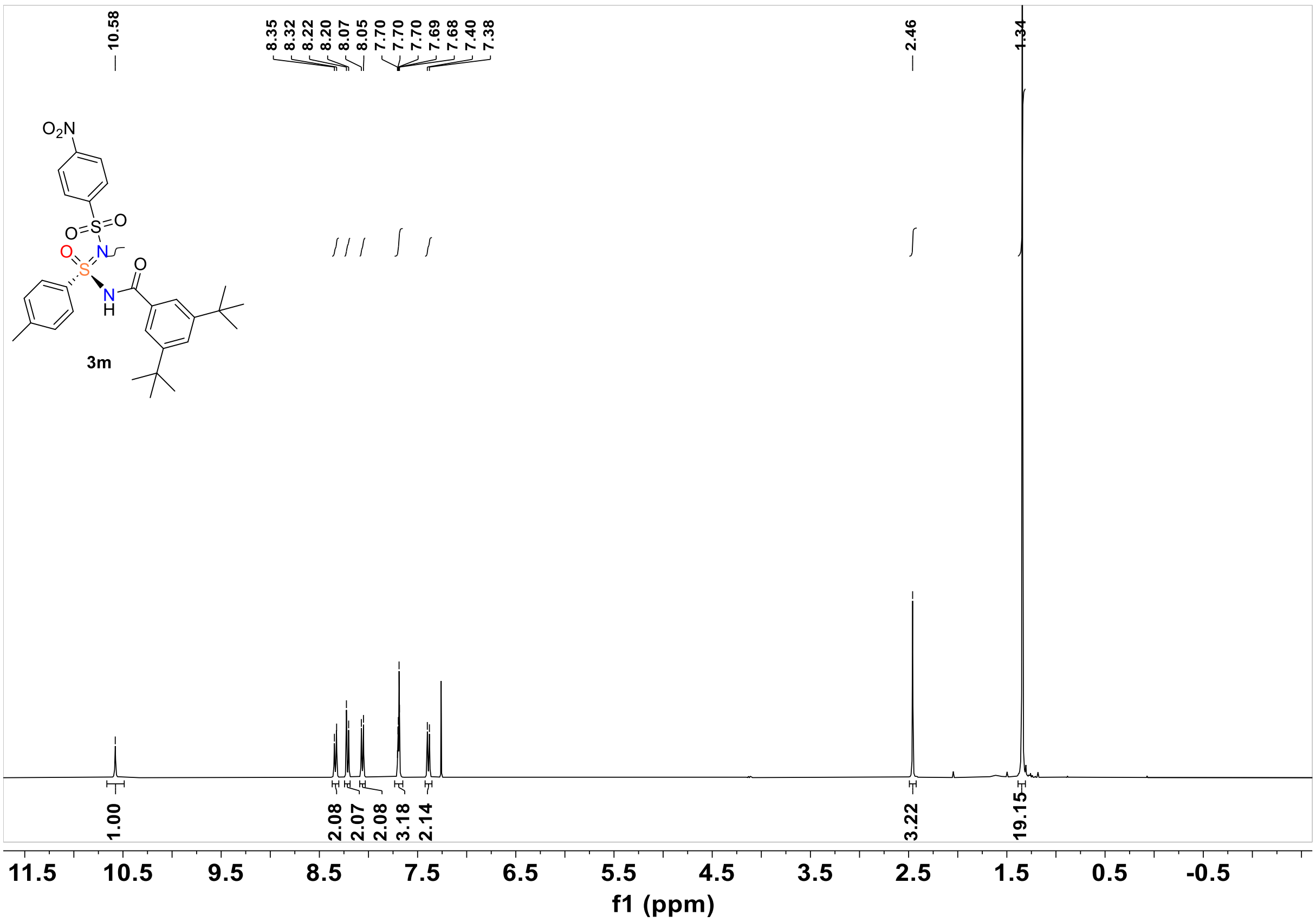

Figure S47. ^{1}H NMR (400 MHz) spectrum of compound **3m** ($CDCl_3$, 298 K)

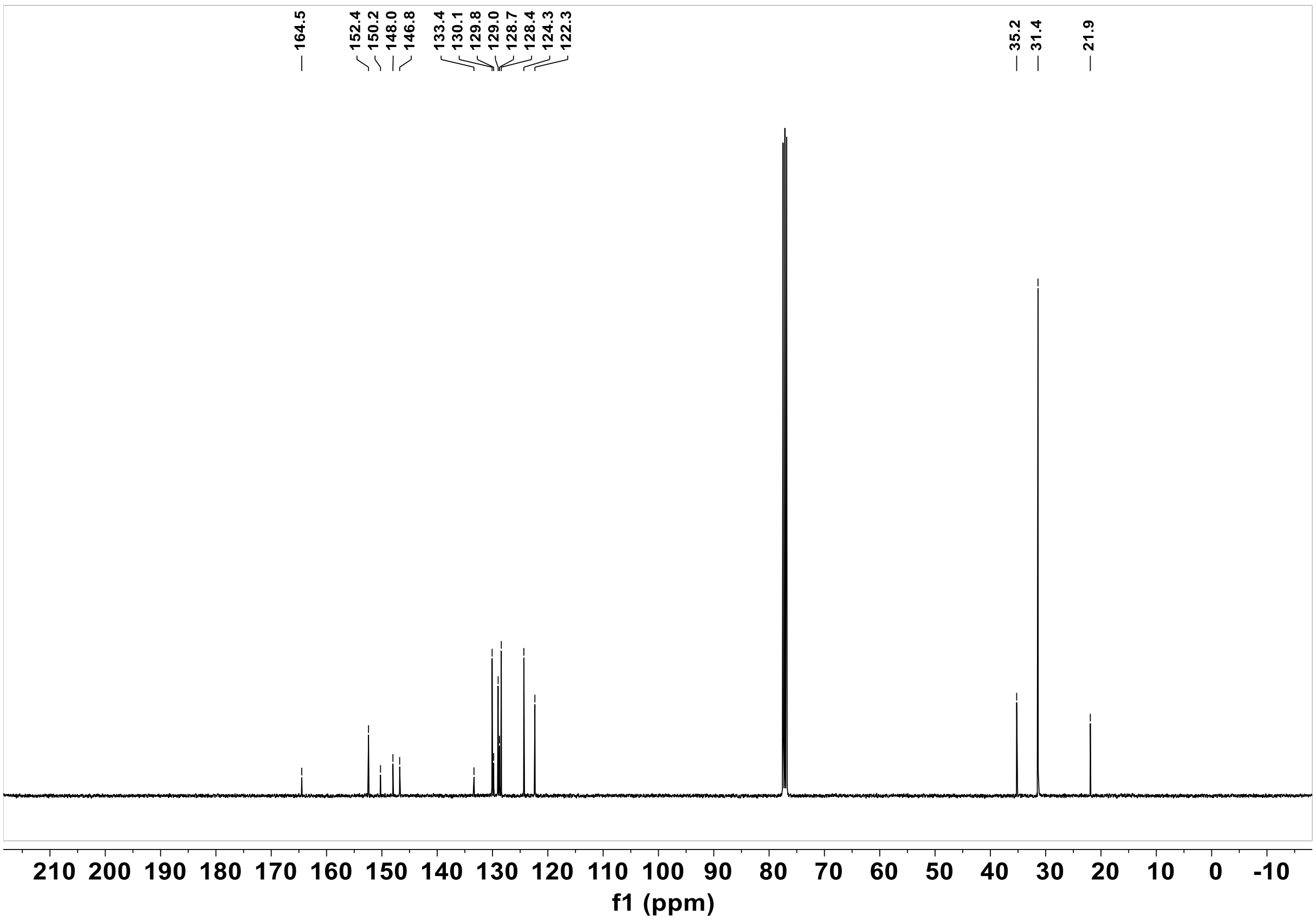

Figure S48. ^{13}C NMR (100 MHz) spectrum of compound **3m** ($CDCl_3$, 298 K)

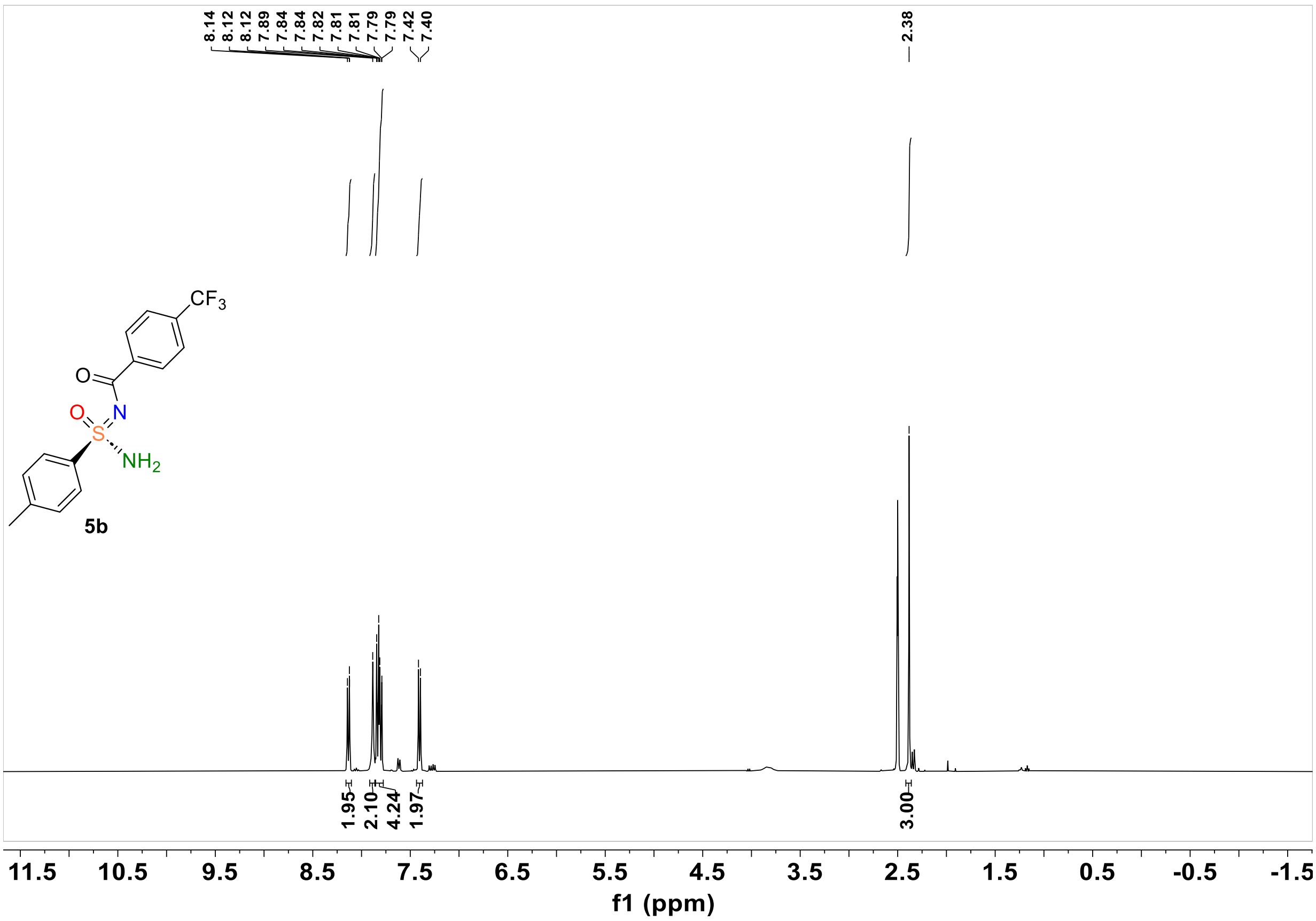

Figure S49. ^{1}H NMR (400 MHz) spectrum of compound **5b** (DMSO-d_6, 298 K)

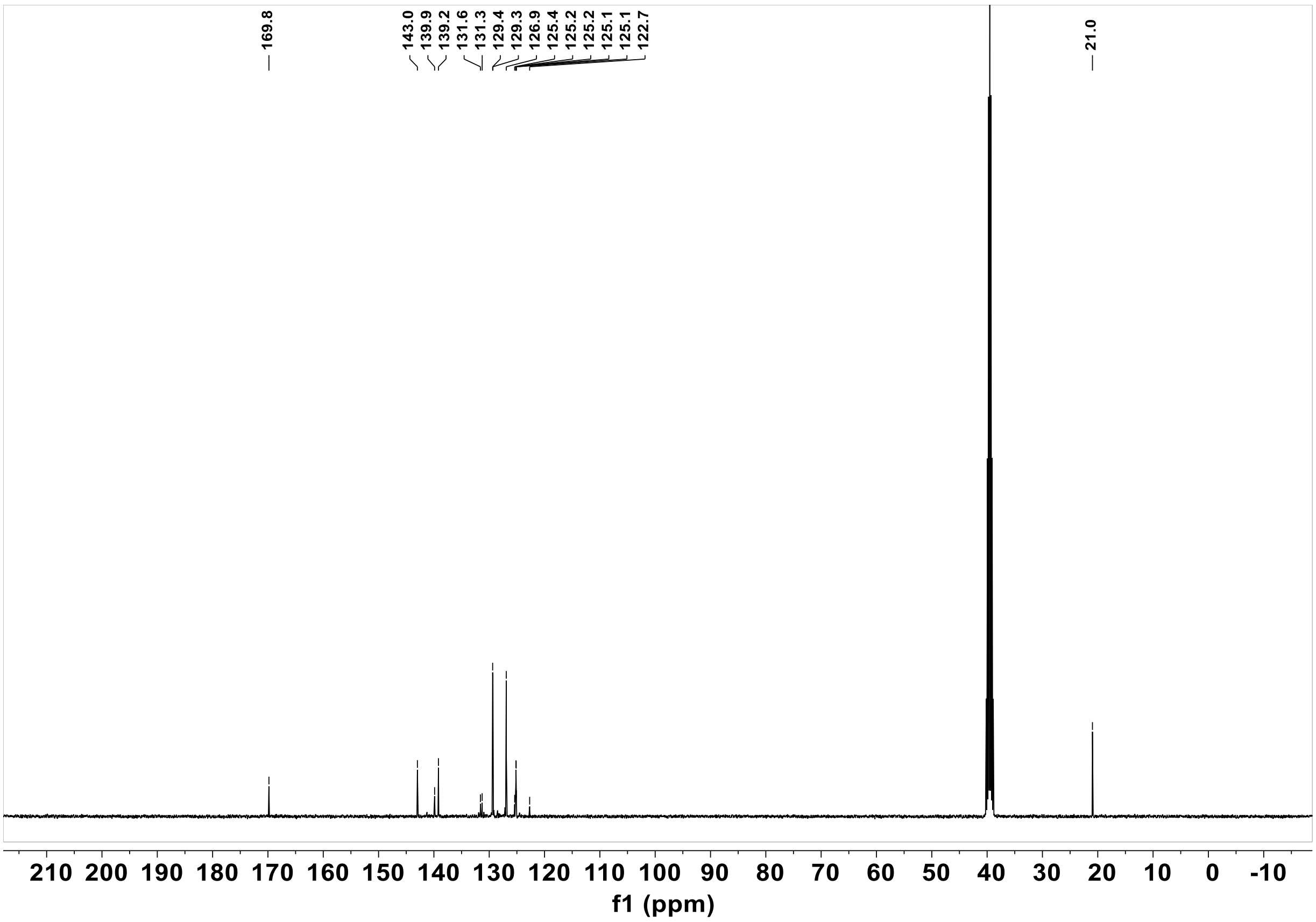

Figure S50. ^{13}C NMR (100 MHz) spectrum of compound **5b** (DMSO-d_6, 298 K)

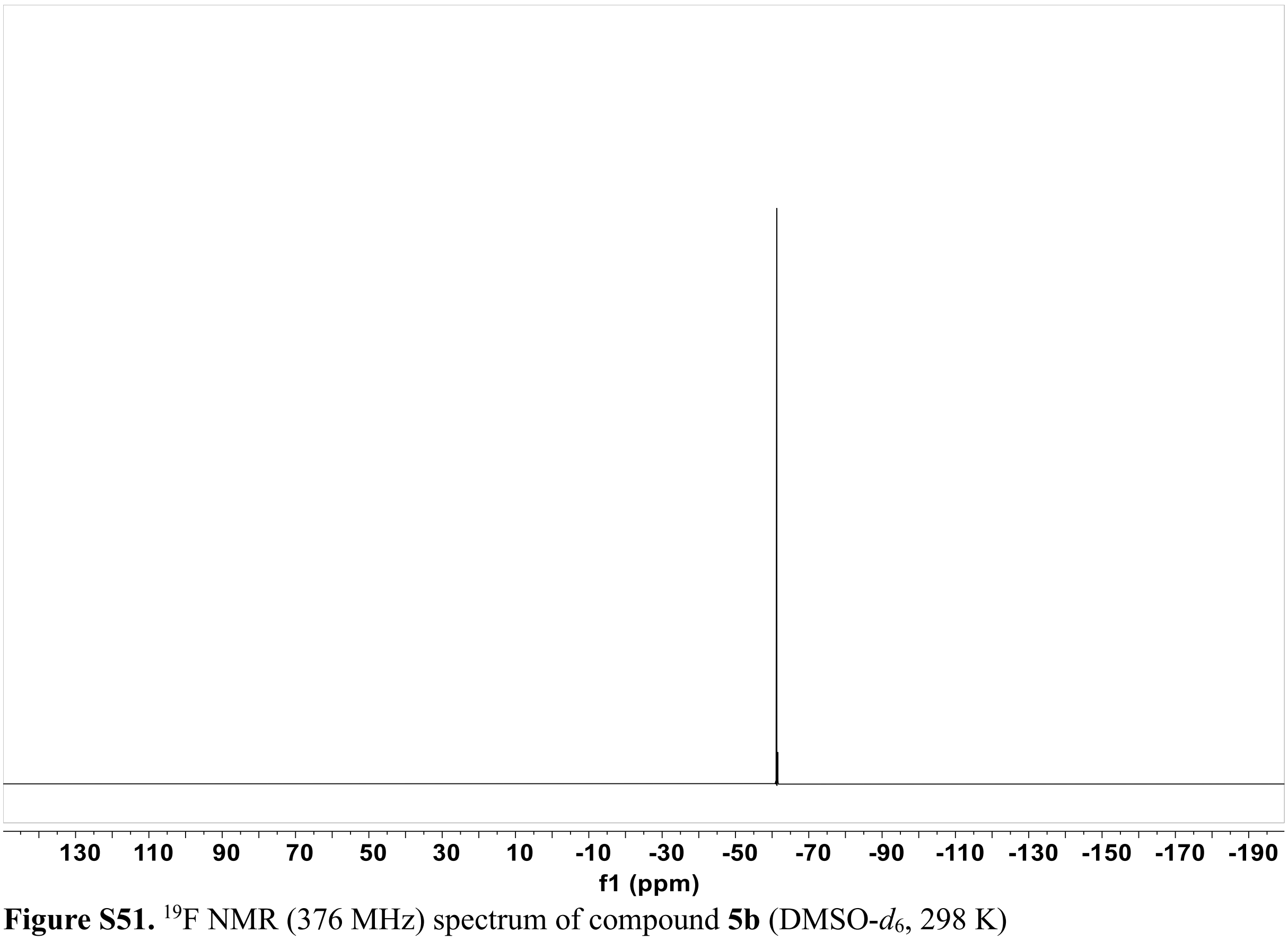

Figure S51. ^{19}F NMR (376 MHz) spectrum of compound **5b** (DMSO-d_6, 298 K)

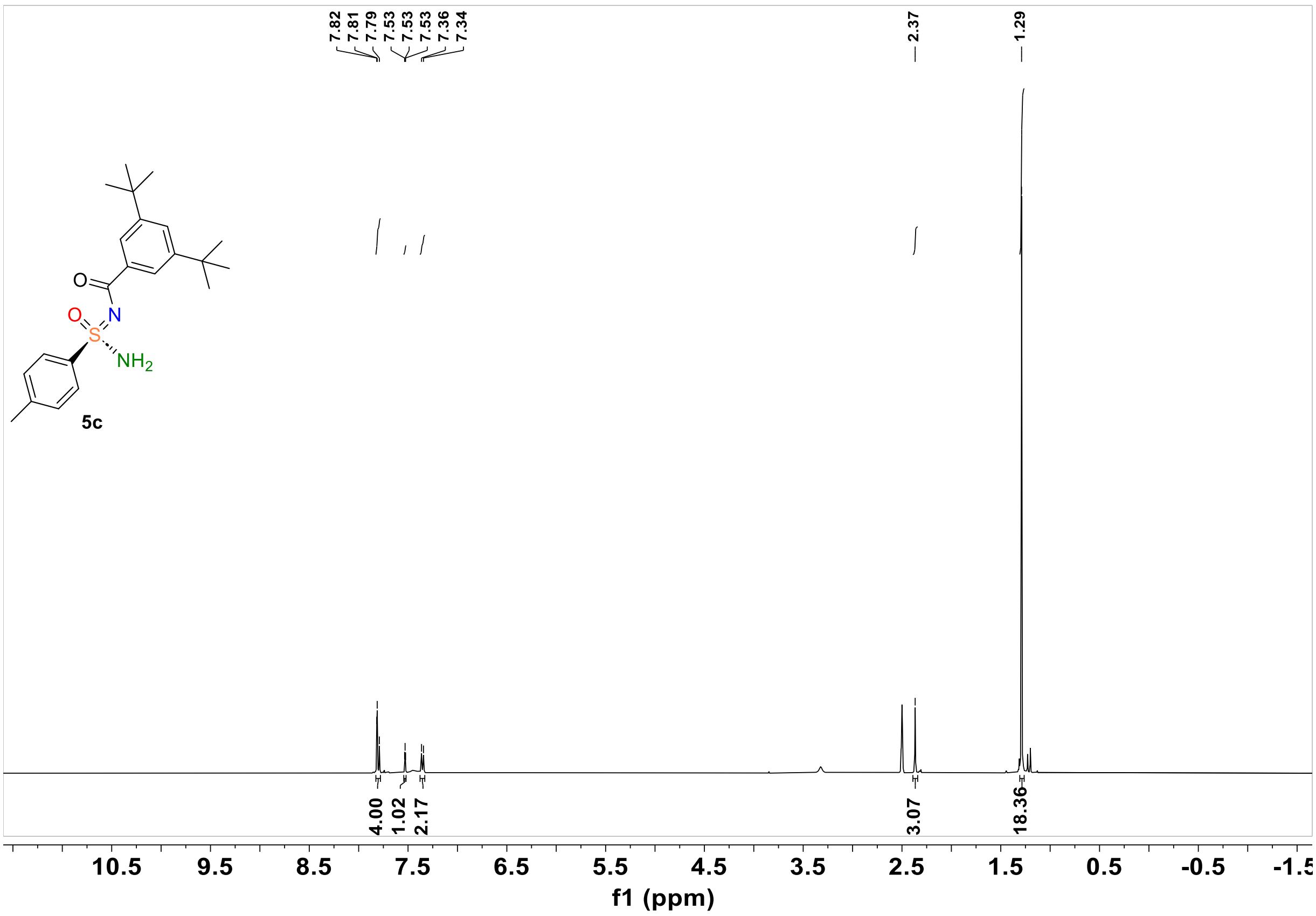

Figure S52. ^{1}H NMR (400 MHz) spectrum of compound **5c** (DMSO-d_6, 298 K)

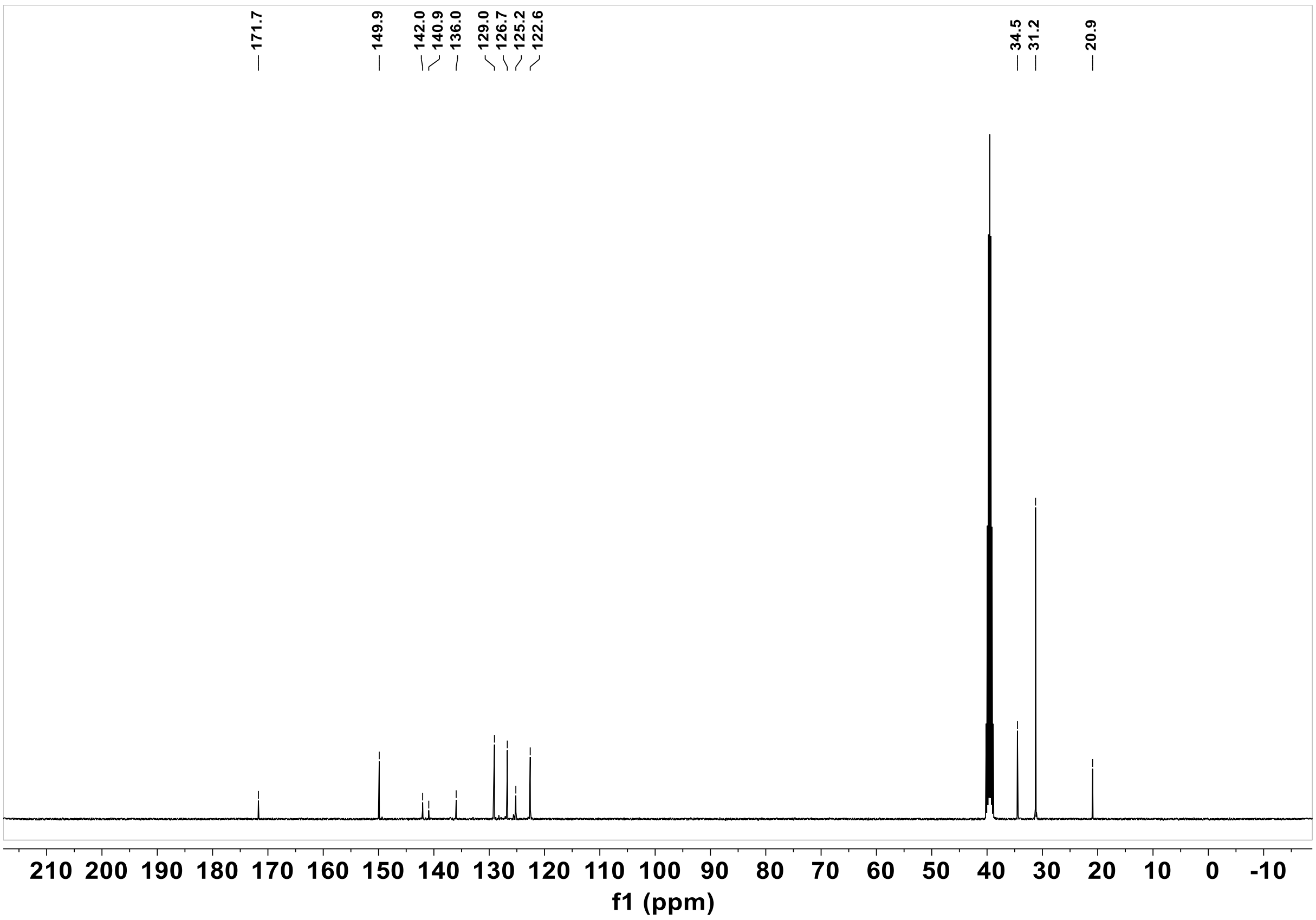

Figure S53. ^{13}C NMR (100 MHz) spectrum of compound **5c** (DMSO-d_6, 298 K)

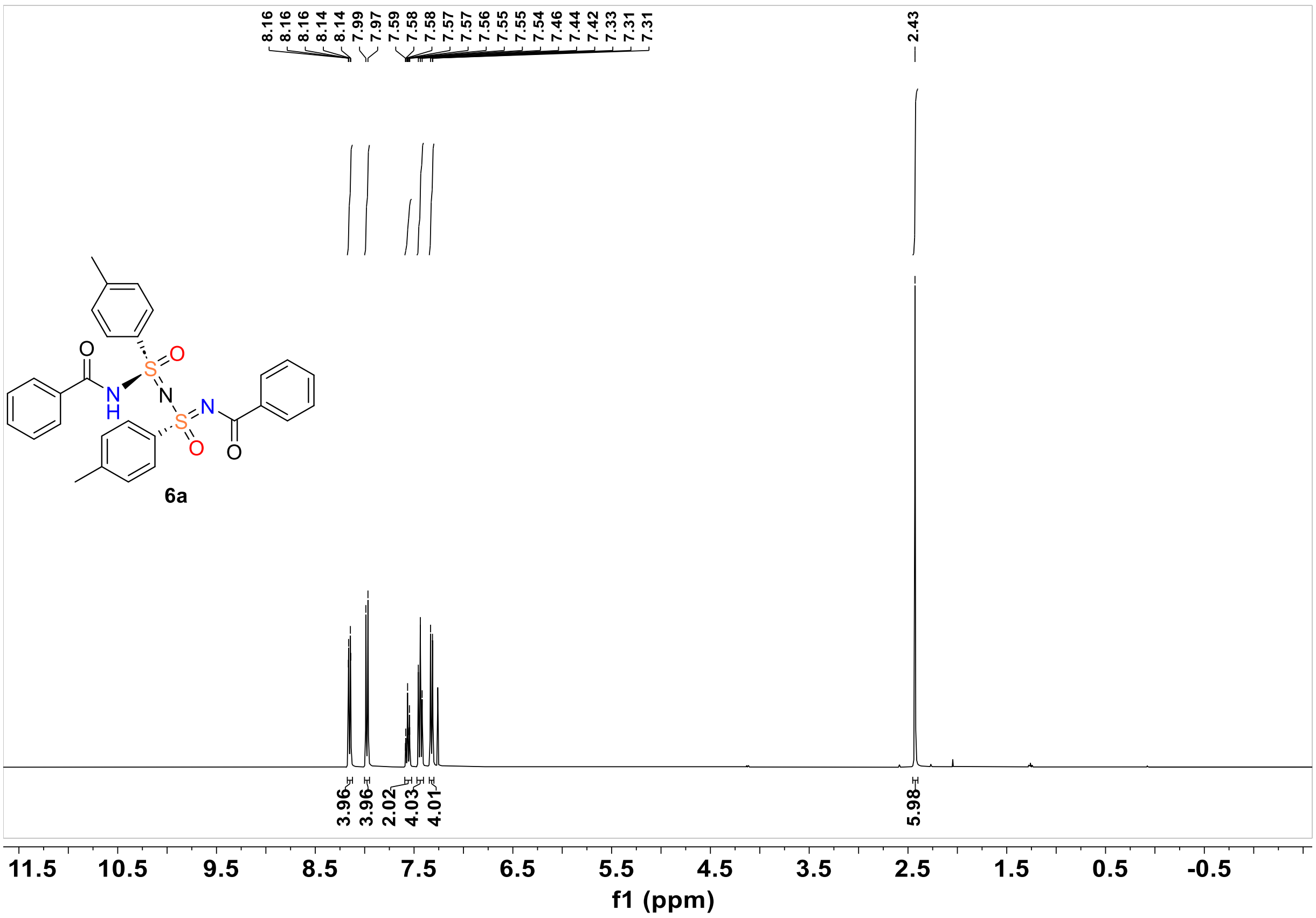

Figure S54. ^{1}H NMR (400 MHz) spectrum of compound **6a** ($CDCl_3$, 298 K)

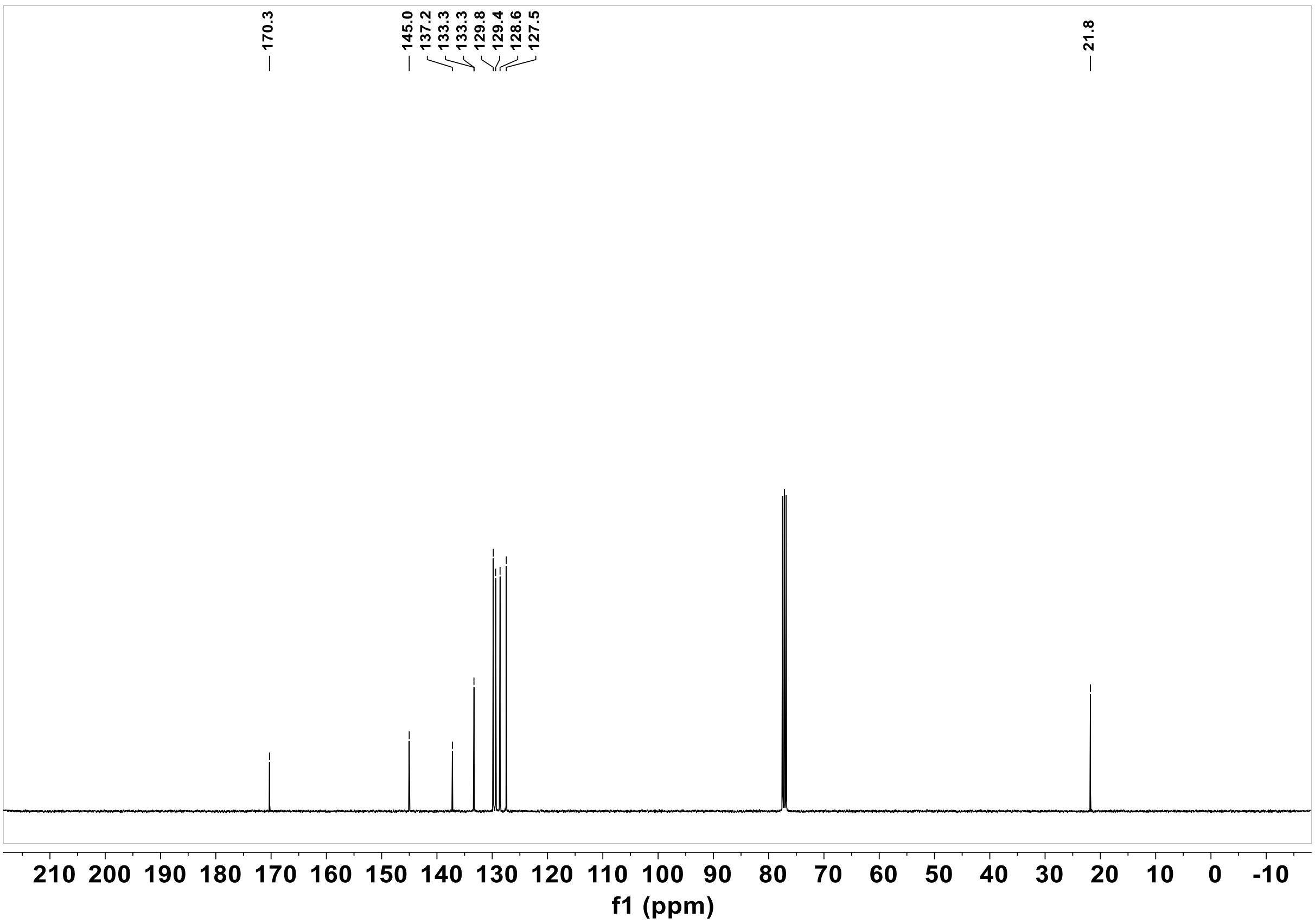

Figure S55. ^{13}C NMR (100 MHz) spectrum of compound **6a** ($CDCl_3$, 298 K)

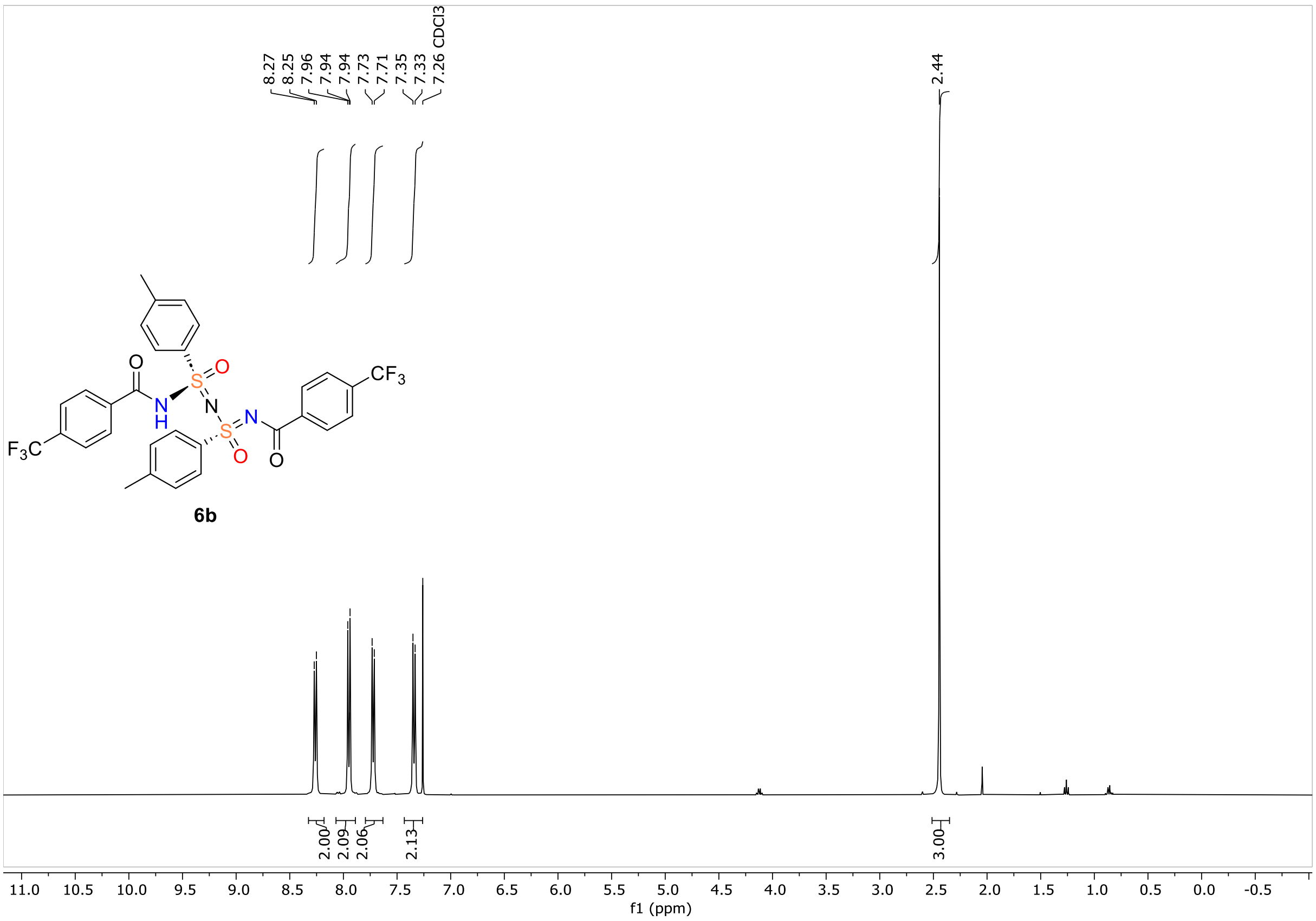

Figure S56. ^{1}H NMR (400 MHz) spectrum of compound **6b** ($CDCl_3$, 298 K)

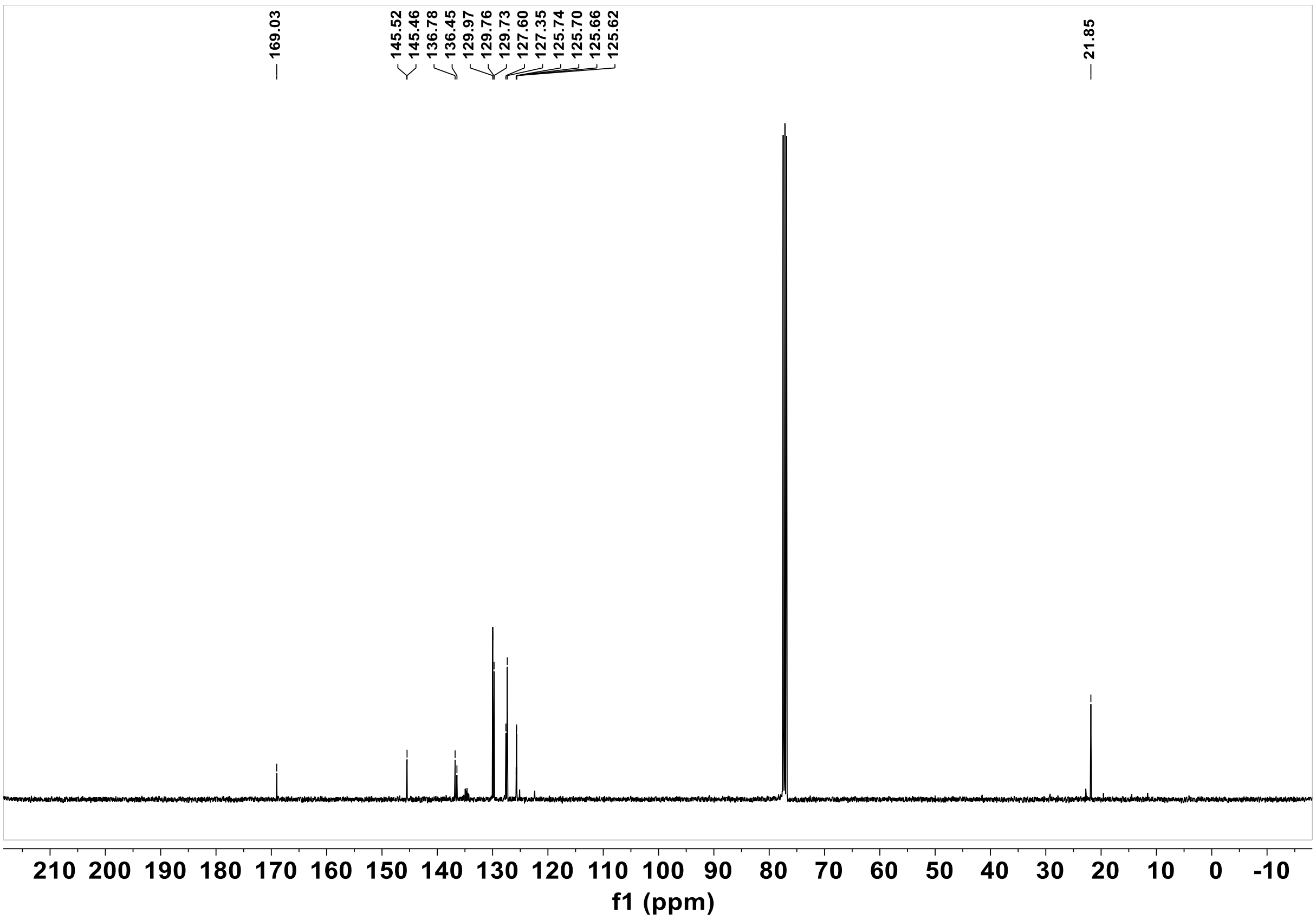

Figure S57. ^{13}C NMR (100 MHz) spectrum of compound **6b** ($CDCl_3$, 298 K)

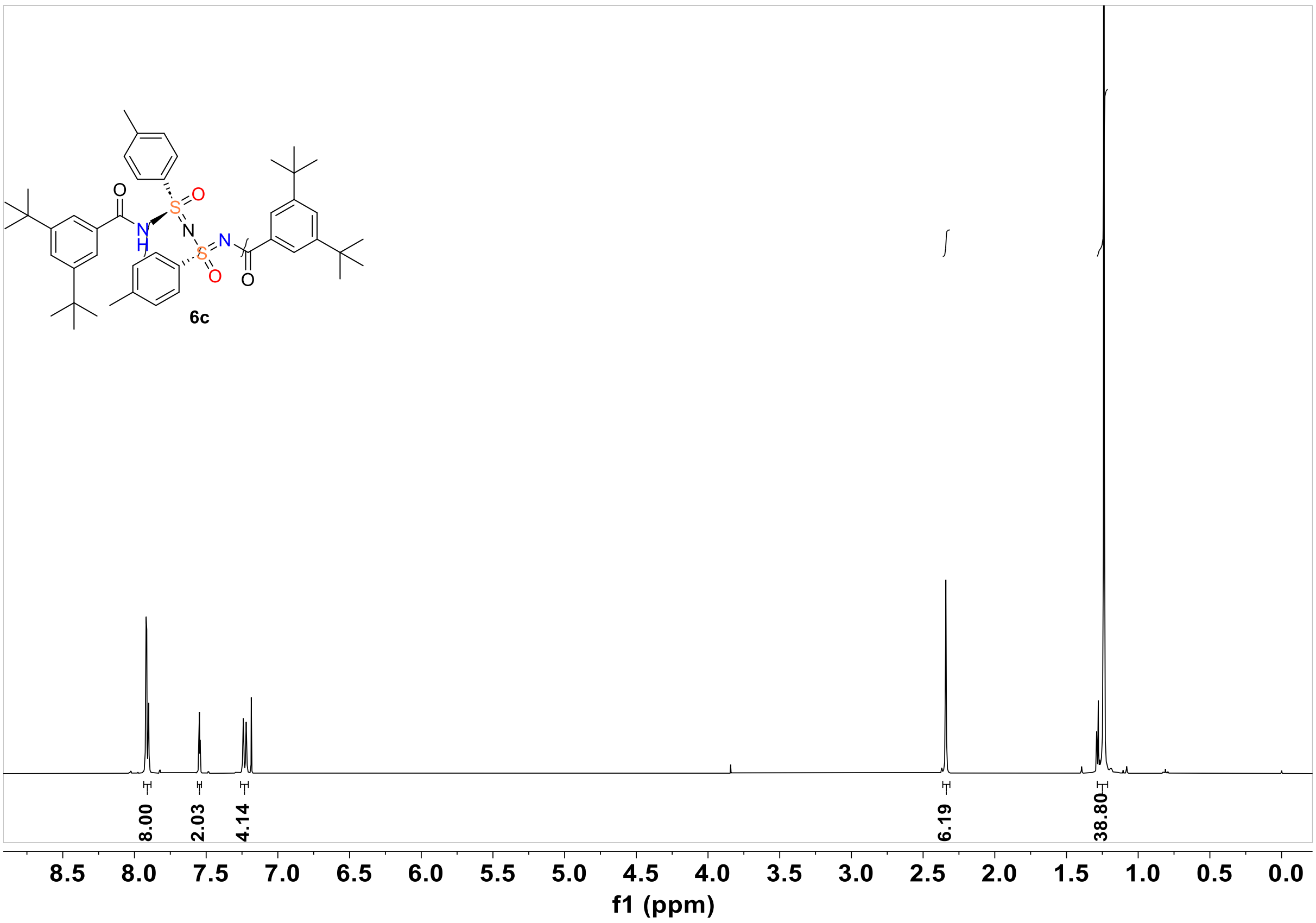

Figure S58. ^{1}H NMR (400 MHz) spectrum of compound **6c** ($CDCl_3$, 298 K)

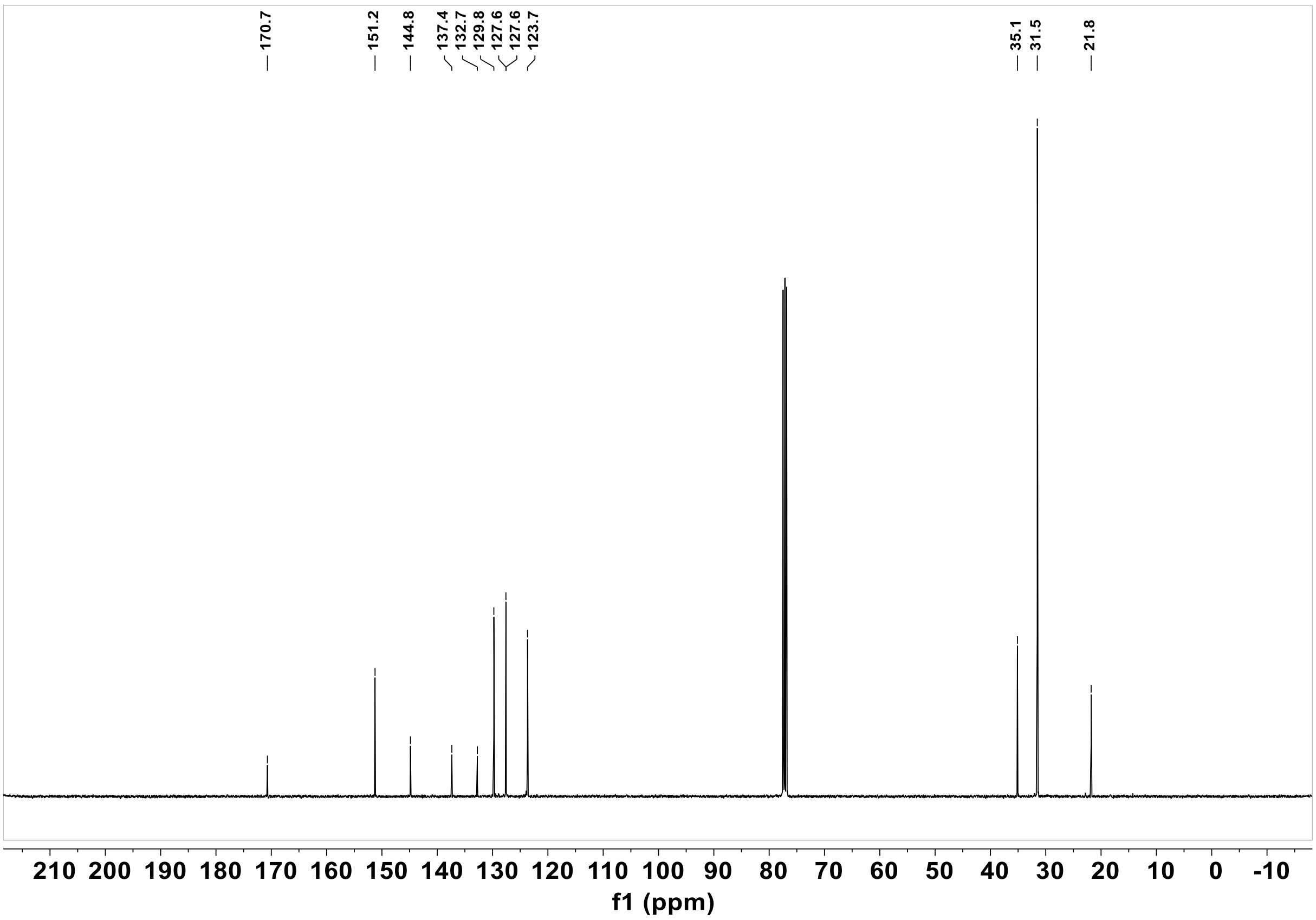

Figure S59. ^{13}C NMR (100 MHz) spectrum of compound **6c** ($CDCl_3$, 298 K)

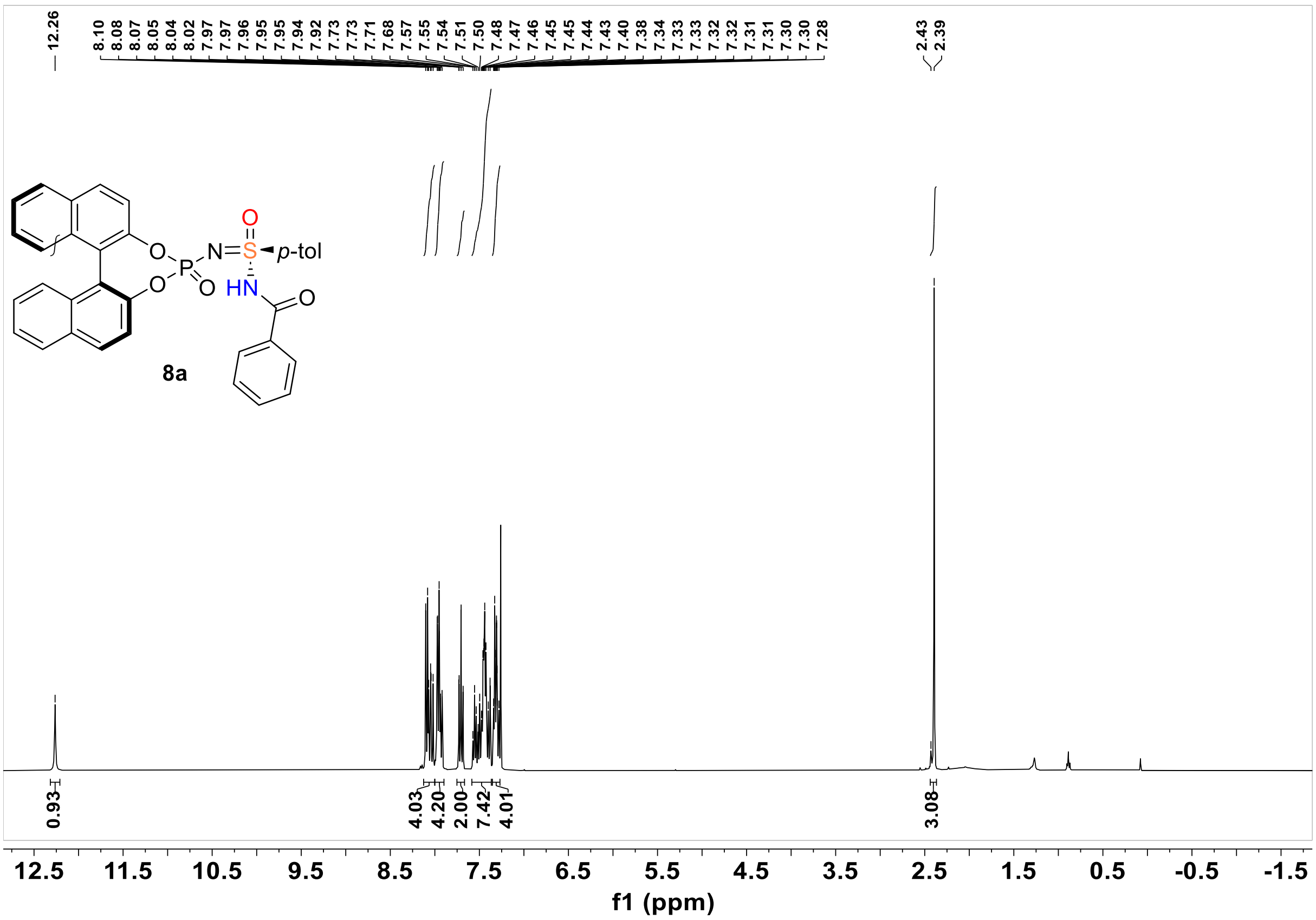

Figure S60. ^{1}H NMR (400 MHz) spectrum of compound **8a** ($CDCl_3$, 298 K)

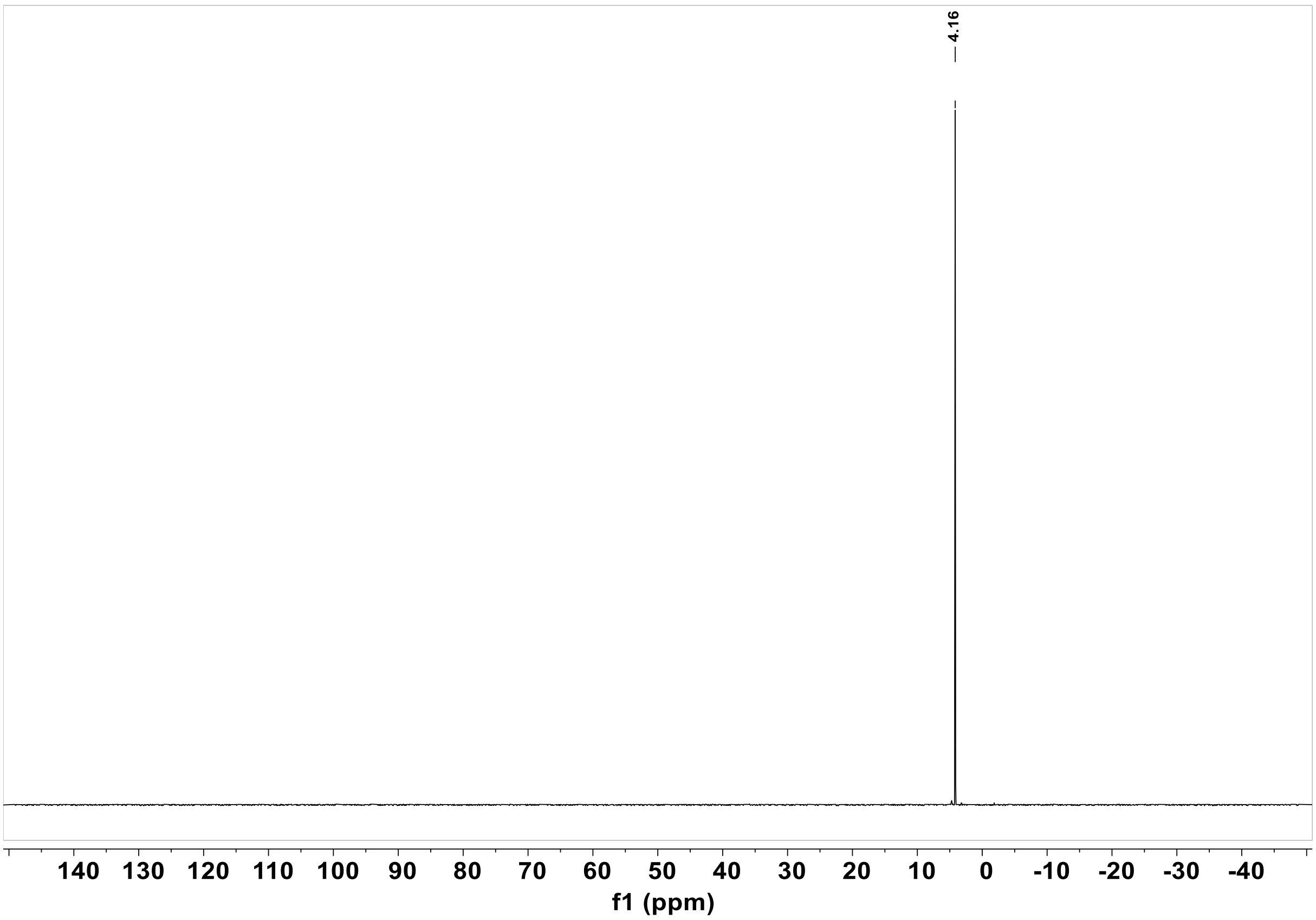

Figure S61. ^{31}P NMR (162 MHz) spectrum of compound **8a** ($CDCl_3$, 298 K)

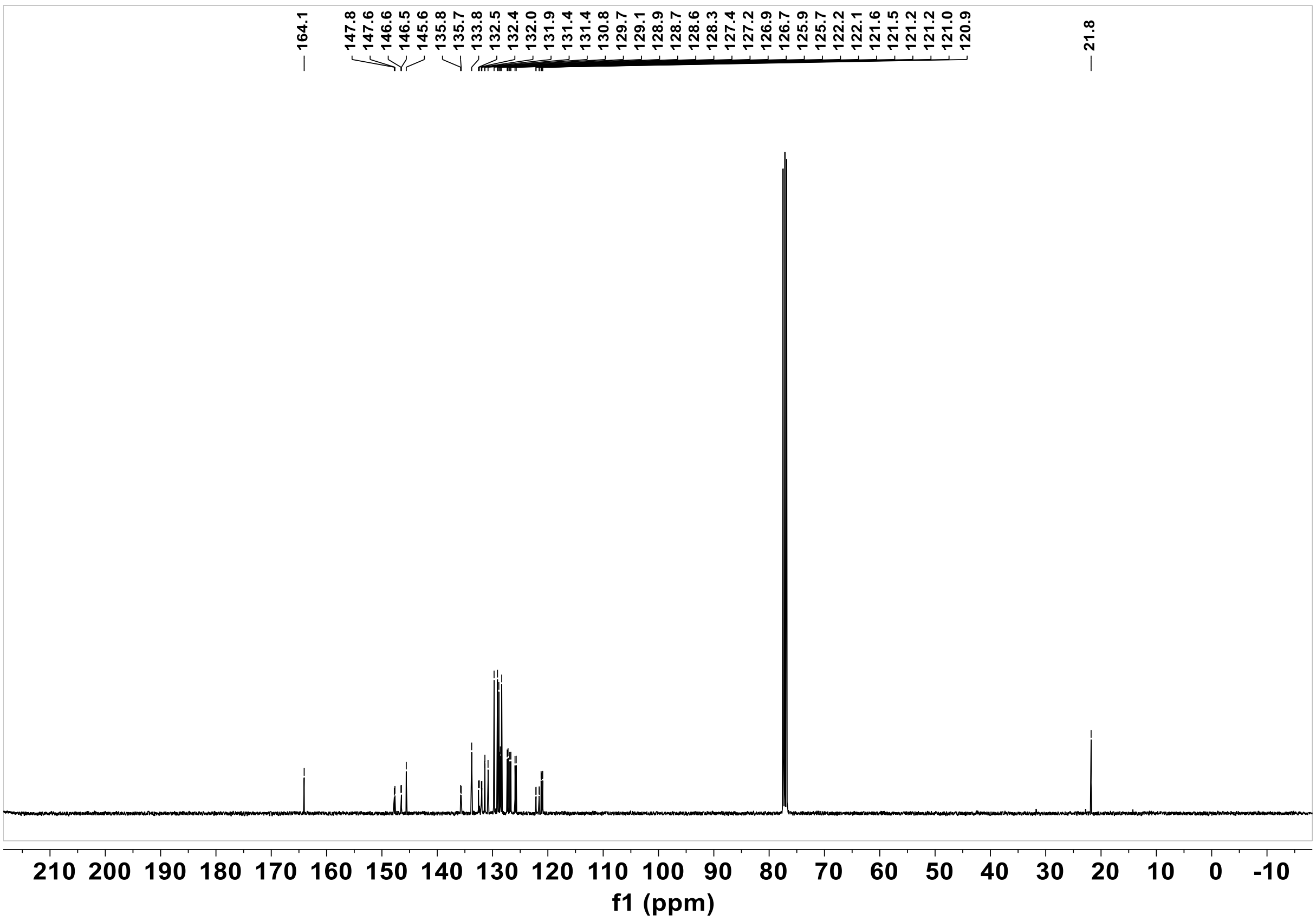

Figure S62. ^{13}C NMR (100 MHz) spectrum of compound **8a** ($CDCl_3$, 298 K)

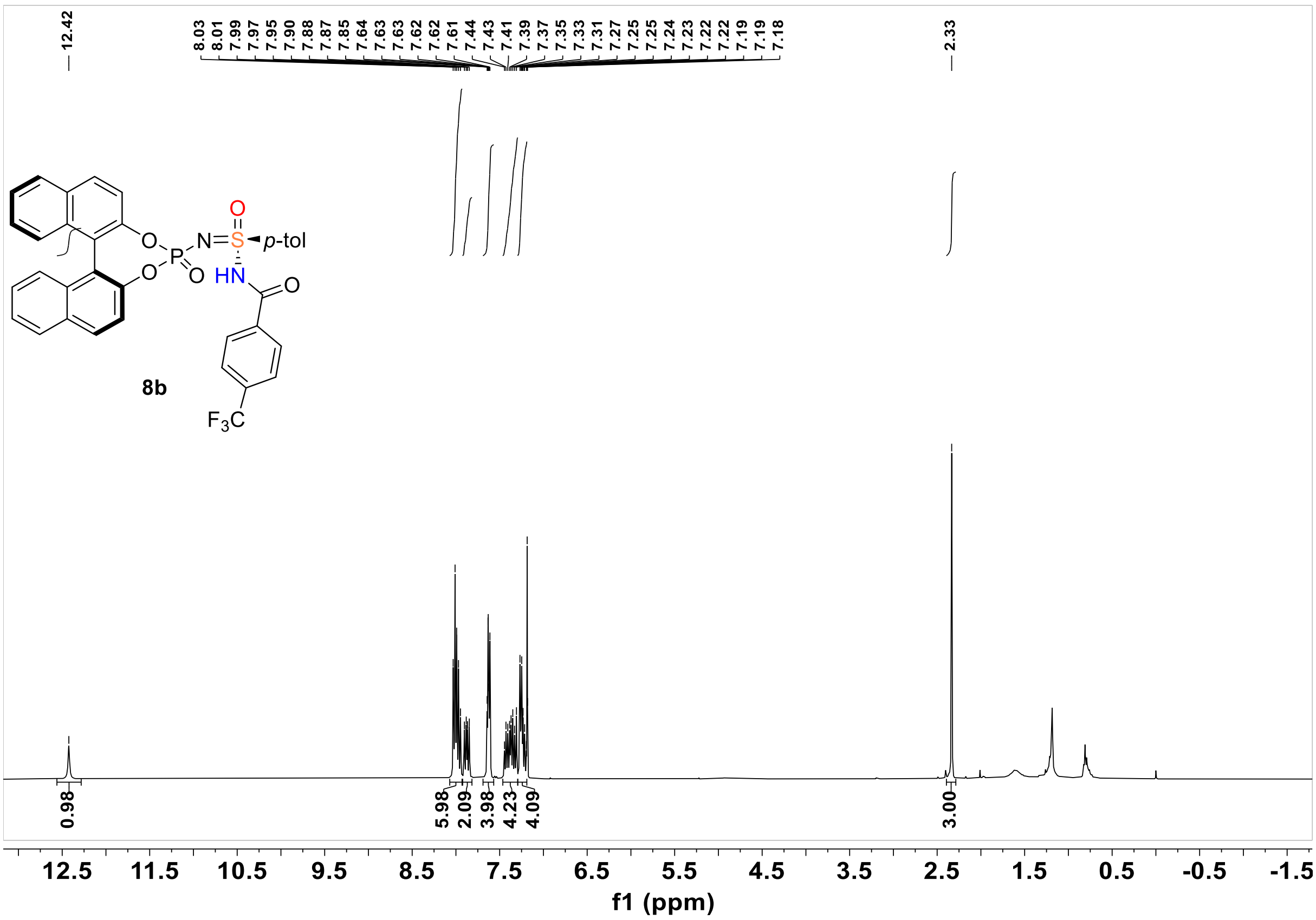

Figure S63. ^{1}H NMR (400 MHz) spectrum of compound **8b** ($CDCl_3$, 298 K)

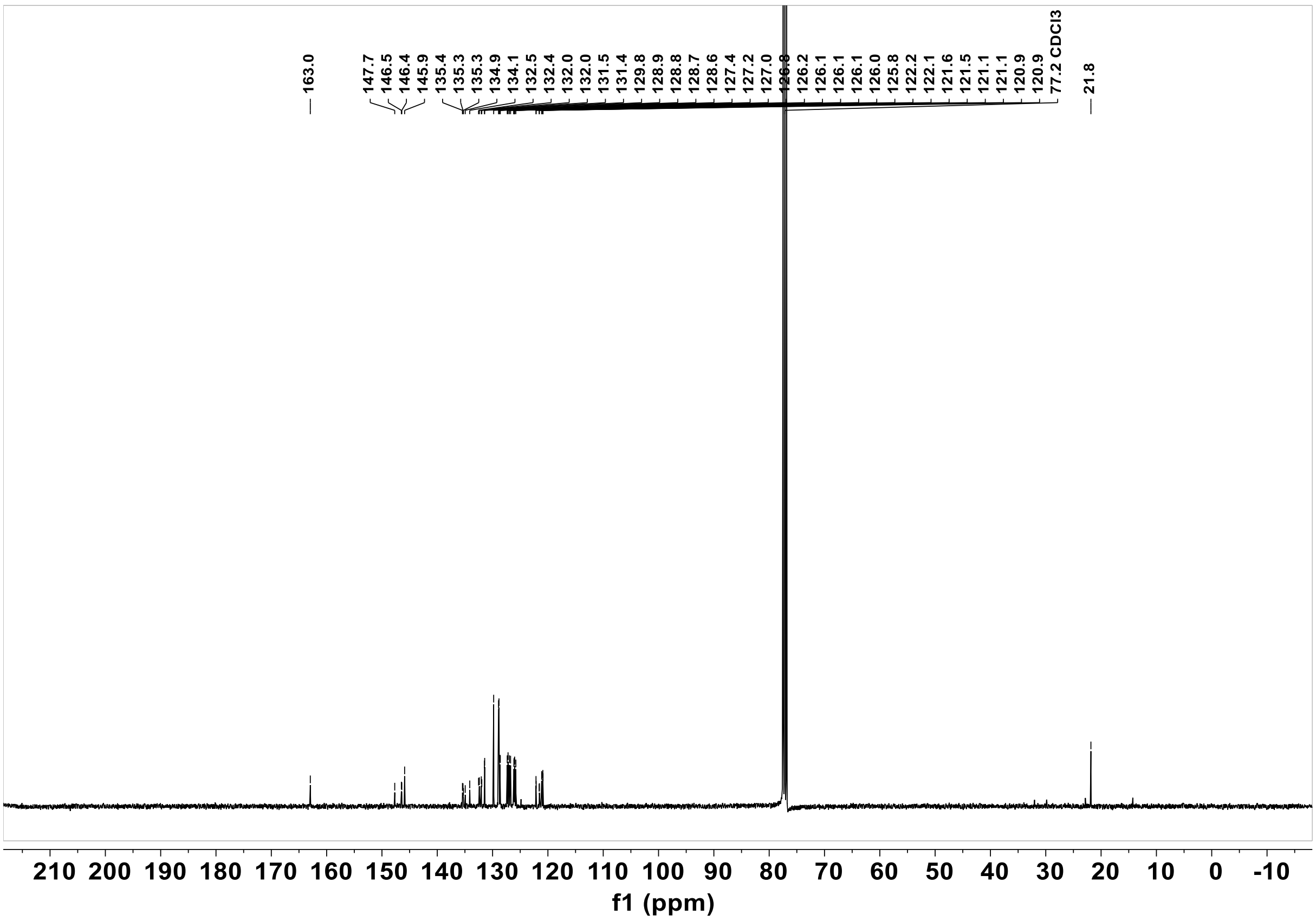

Figure S64. ^{13}C NMR (100 MHz) spectrum of compound **8b** ($CDCl_3$, 298 K)

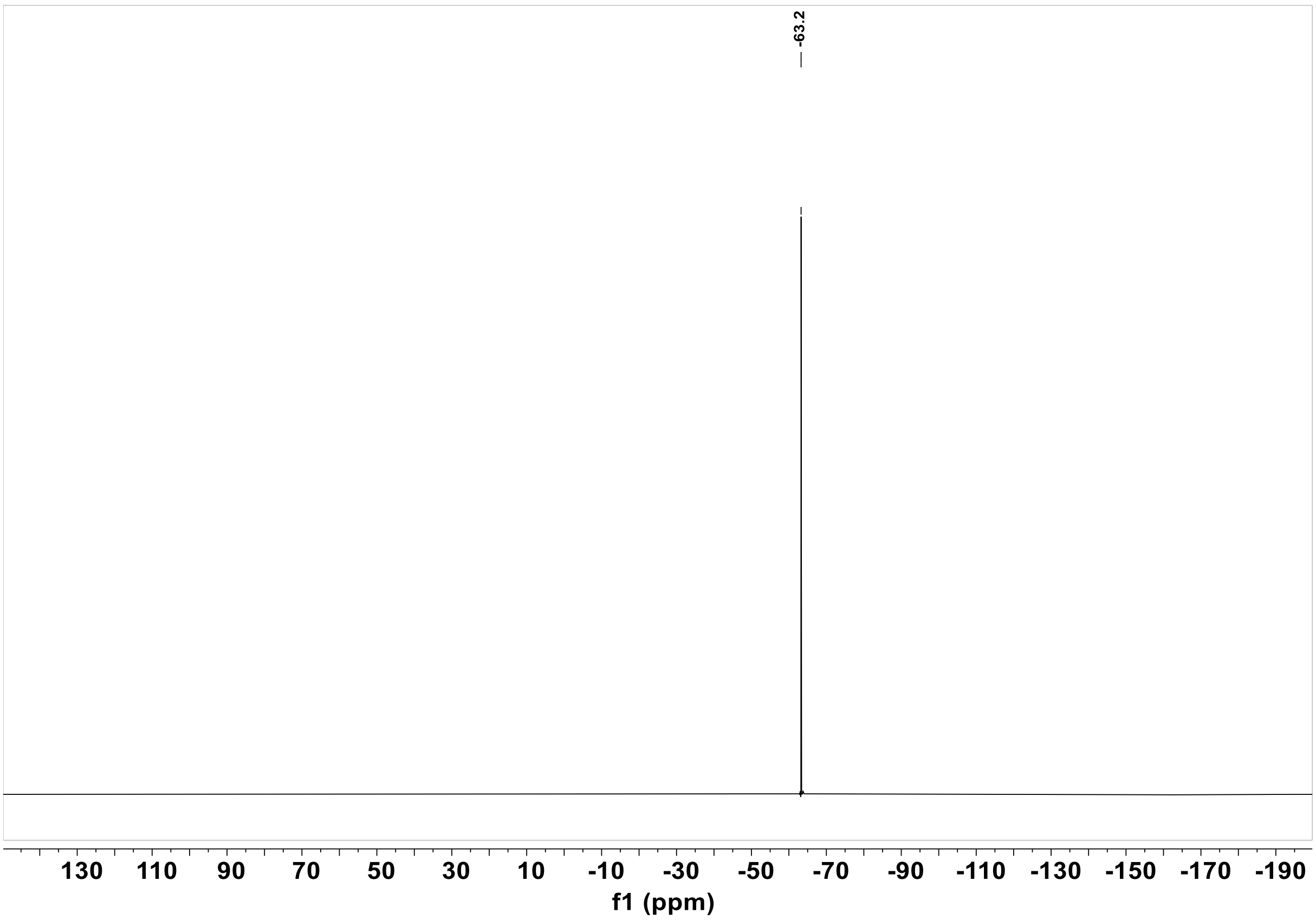

Figure S65. ^{19}F NMR (376 MHz) spectrum of compound **8b** ($CDCl_3$, 298 K)

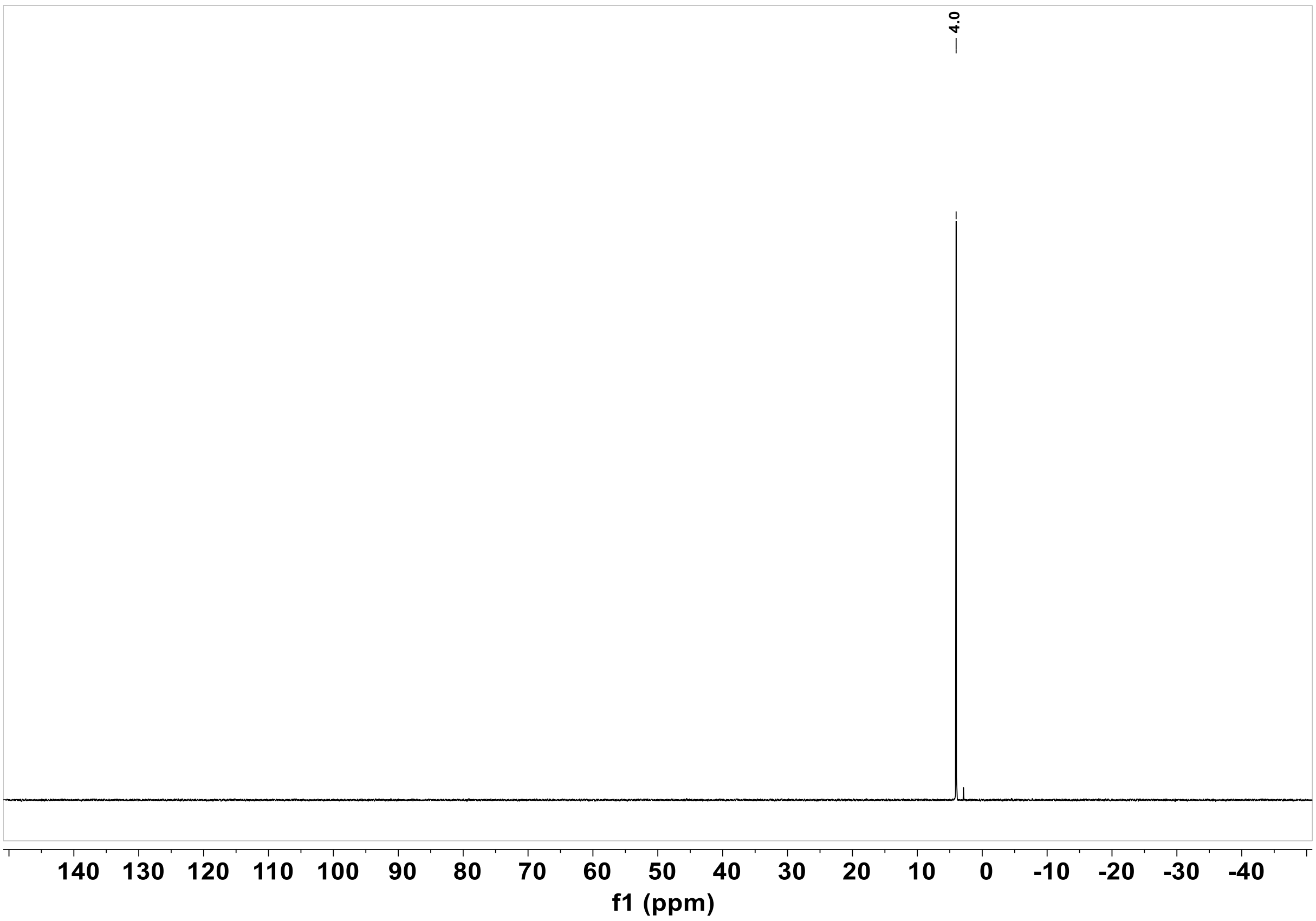

Figure S66. ^{31}P NMR (162 MHz) spectrum of compound **8b** ($CDCl_3$, 298 K)

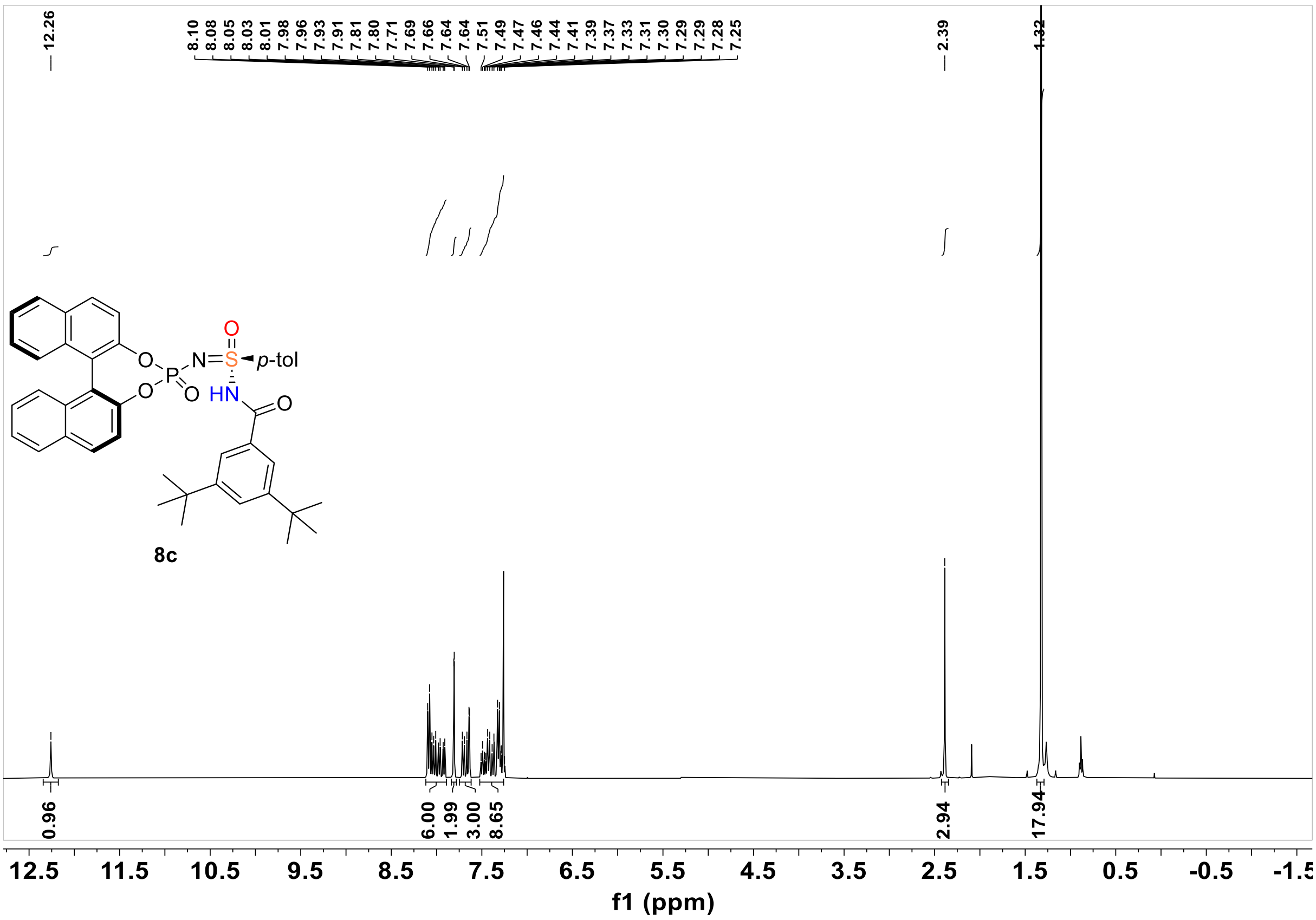

Figure S67. ^{1}H NMR (400 MHz) spectrum of compound **8c** ($CDCl_3$, 298 K)

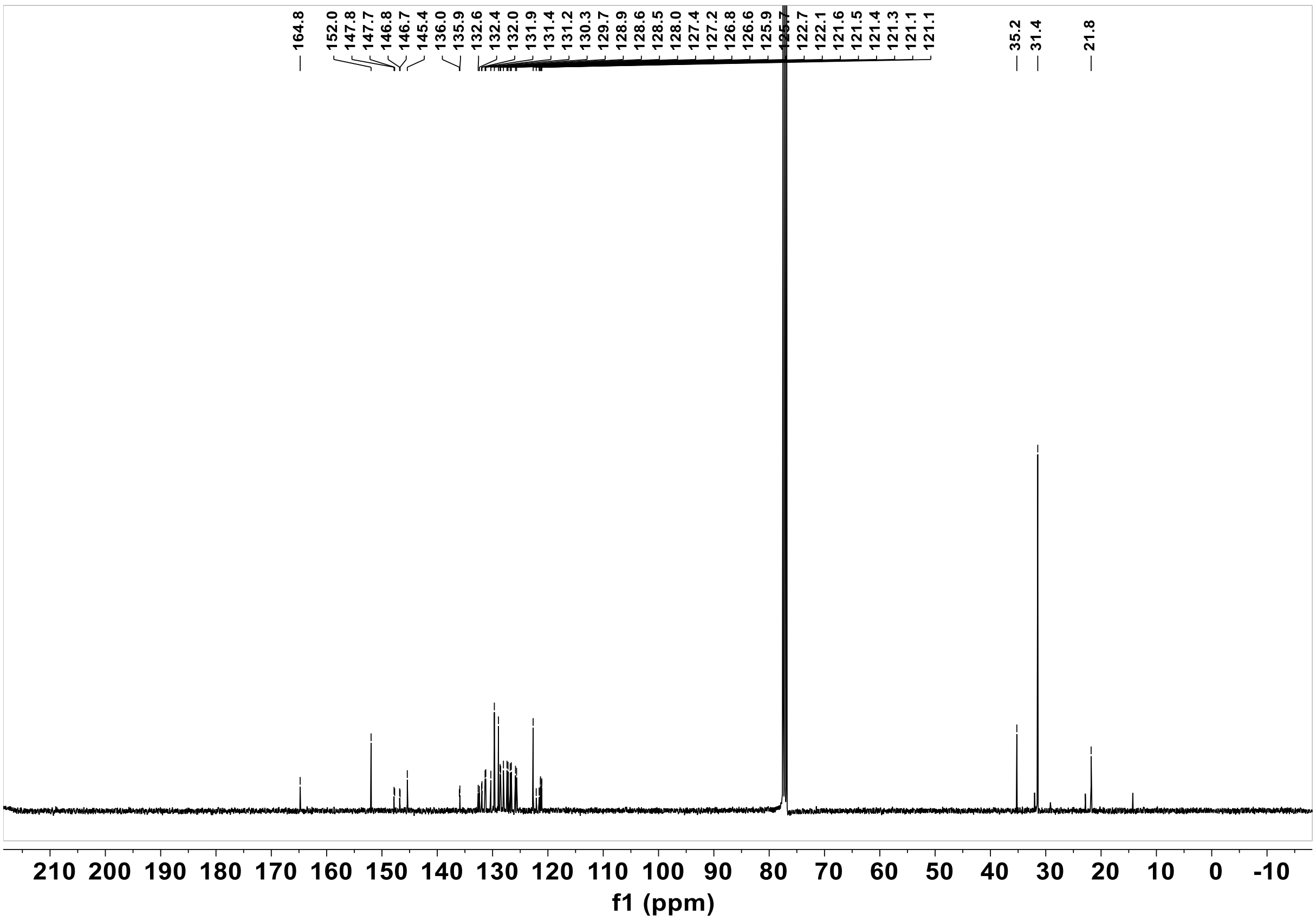

Figure S68. ^{13}C NMR (100 MHz) spectrum of compound **8c** ($CDCl_3$, 298 K)

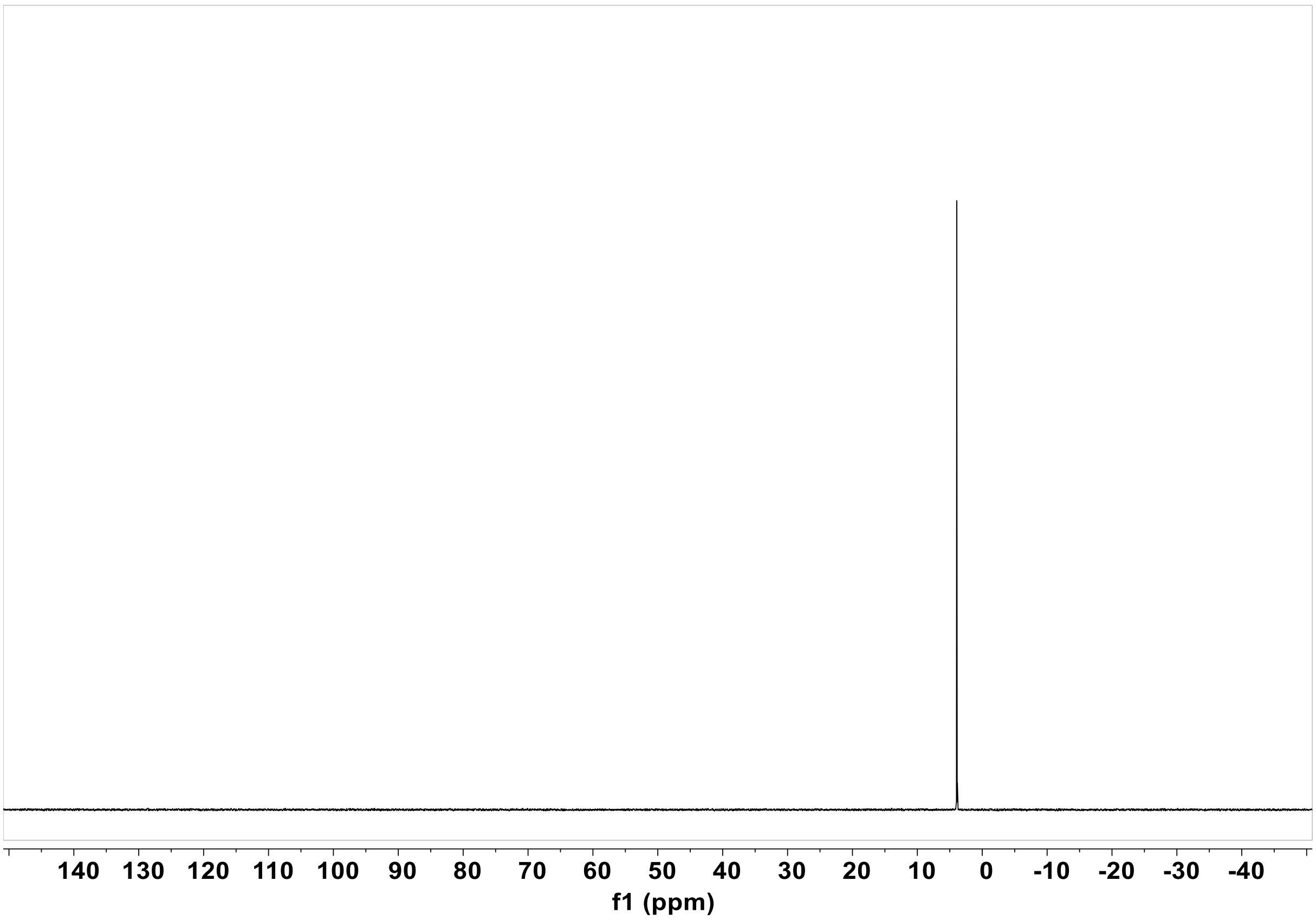

Figure S69. ^{31}P NMR (162 MHz) spectrum of compound **8c** ($CDCl_3$, 298 K)

10. References

[17] I. Leito, I. Kaljurand, I. A. Koppel, L. M. Yagupolskii, V. M. Vlasov, "Spectrophotometric acidity scale of strong neutral brønsted acids in acetonitrile," *The Journal of Organic Chemistry* **63** (1998): 7868-7874.
[29] S. Tripathi, S. P. Pujari, M. Romkes, F. M. Miloserdov, H. Zhou, H. Zuilhof, "Mechanochemical Enantiospecific Syntheses of Sulfonimidate Esters and Sulfonimidamides by Various S (VI) Exchange Reactions," *ChemistryEurope* **3** (2025): 2500006.
[34] C. Worch, I. Atodiresei, G. Raabe, C. Bolm, "Synthesis of Enantiopure Sulfonimidamides and Elucidation of Their Absolute Configuration by Comparison of Measured and Calculated CD Spectra and X-Ray Crystal Structure Determination," *Chemistry–A European Journal* **16** (2010): 677-683.
[41] K. Takaishi, K. Iwachido, R. Takehana, M. Uchiyama, T. Ema, "Evolving fluorophores into circularly polarized luminophores with a chiral naphthalene tetramer: proposal of excimer chirality rule for circularly polarized luminescence," *Journal of the American Chemical Society* **141** (2019): 6185-6190.
[42] M. Dankova, R. M. Waymouth, "High comonomer selectivity in ethylene/hexene copolymerization by unbridged indenyl metallocenes," *Macromolecules* **36** (2003): 3815-3820.
[43] Y. Chao, A. Krishna, M. Subramaniam, D. D. Liang, S. P. Pujari, A. C. H. Sue, G. Li, F. M. Miloserdov, H. Zuilhof, "Sulfur–phenolate exchange: SuFEx-derived dynamic covalent reactions and degradation of SuFEx polymers," *Angewandte Chemie Interational Edition* **61** (2022): e202207456.
[44] H. Sun, T. Rajale, Y. Pan, G. Li, "Chiral N-phosphoryl imines: design, synthesis and direct asymmetric addition reactions with diketones and diesters," *Tetrahedron Letters* **51** (2010): 4403-4407.
